# Zelkovamycin is an OXPHOS Inhibitory Member of the Argyrin Natural Product Family†

**DOI:** 10.1002/chem.202001577

**Published:** 2020-06-17

**Authors:** Daniel Krahn, Geronimo Heilmann, Felix C. E. Vogel, Chrisovalantis Papadopoulos, Susanne Zweerink, Farnusch Kaschani, Hemmo Meyer, Alexander Roesch, Markus Kaiser

**Affiliations:** ^1^ Chemische Biologie Universität Duisburg-Essen ZMB Fakultät für Biologie Universitätsstr. 2 45117 Essen Germany; ^2^ Department of Dermatology University hospital Essen West German Cancer Center University Duisburg-Essen and the German Cancer Consortium (DKTK); ^3^ Current address: Division of Tumor Metabolism and Microenvironment German Cancer Research Center (DKFZ) Im Neuenheimer Feld 280 69120 Heidelberg Germany; ^4^ Molekularbiologie I Universität Duisburg-Essen ZMB Fakultät für Biologie Universitätsstr. 2 45117 Essen Germany; ^5^ Current address: University of Cologne Faculty of Medicine and University Hospital of Cologne Department of Gastroenterology and Hepatology Kerpener Str. 62 50937 Cologne Germany

**Keywords:** argyrin, bioactivity, cyclopeptides, natural products, zelkovamycin

## Abstract

Natural products (NPs) are an important inspirational source for developing drugs and chemical probes. In 1999, the group of Ōmura reported the constitutional elucidation of zelkovamycin. Although largely unrecognized so far, this NP displays structural similarities as well as differences to the argyrin NP family, a class of peptidic NPs with promising anticancer activities and diverse mode‐of‐action at the molecular level. By a combination of structure elucidation experiments, the first total synthesis of zelkovamycin and bioassays, the zelkovamycin configuration was determined and its previously proposed molecular structure was revised. The full structure assignment proves zelkovamycin as an additional member of the argyrins with however unique OXPHOS inhibitory properties. Zelkovamycin may therefore not only serve as a new starting point for chemical inhibitors of the OXPHOS system, but also guide customized argyrin NP isolation and biosynthesis studies.

## Graphical abstract

**Zelkovamycin** is an argyrin natural product. By a combination of structural elucidation methodologies, total synthesis and bioassays, the exact molecular structure of zelkovamycin was assigned and revised and its OXHPOS inhibitory properties revealed.

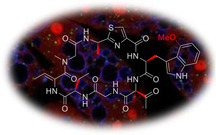

Natural products (NPs) represent an exceptional source of inspirations in drug discovery and frequently serve as valuable starting points in chemical probe synthesis.^1^ To harness the full potential of NPs, a profound characterization of their chemical structure as well as inherent bioactivities is however essential.^2^

In 1999, the group of Ōmura reported the isolation of the NP zelkovamycin as a moderately potent antibacterial cycloheptapeptide from a fermentation broth of *Streptomyces sp*. K96‐0670 (Figure 1 a).^3^ In 2002, the argyrins, a class of currently fifteen cycloheptapeptide NPs, were discovered which besides antibiotic properties show very potent and promising anticancer activities (Figure 1 b).^4^ Although largely unrecognized to date, zelkovamycin obviously displays pronounced structural similarities with the argyrin NP family, suggesting that also zelkovamycin may harbor so far unexplored bioactivities (Figure 1 a,b).

**Figure 1:**
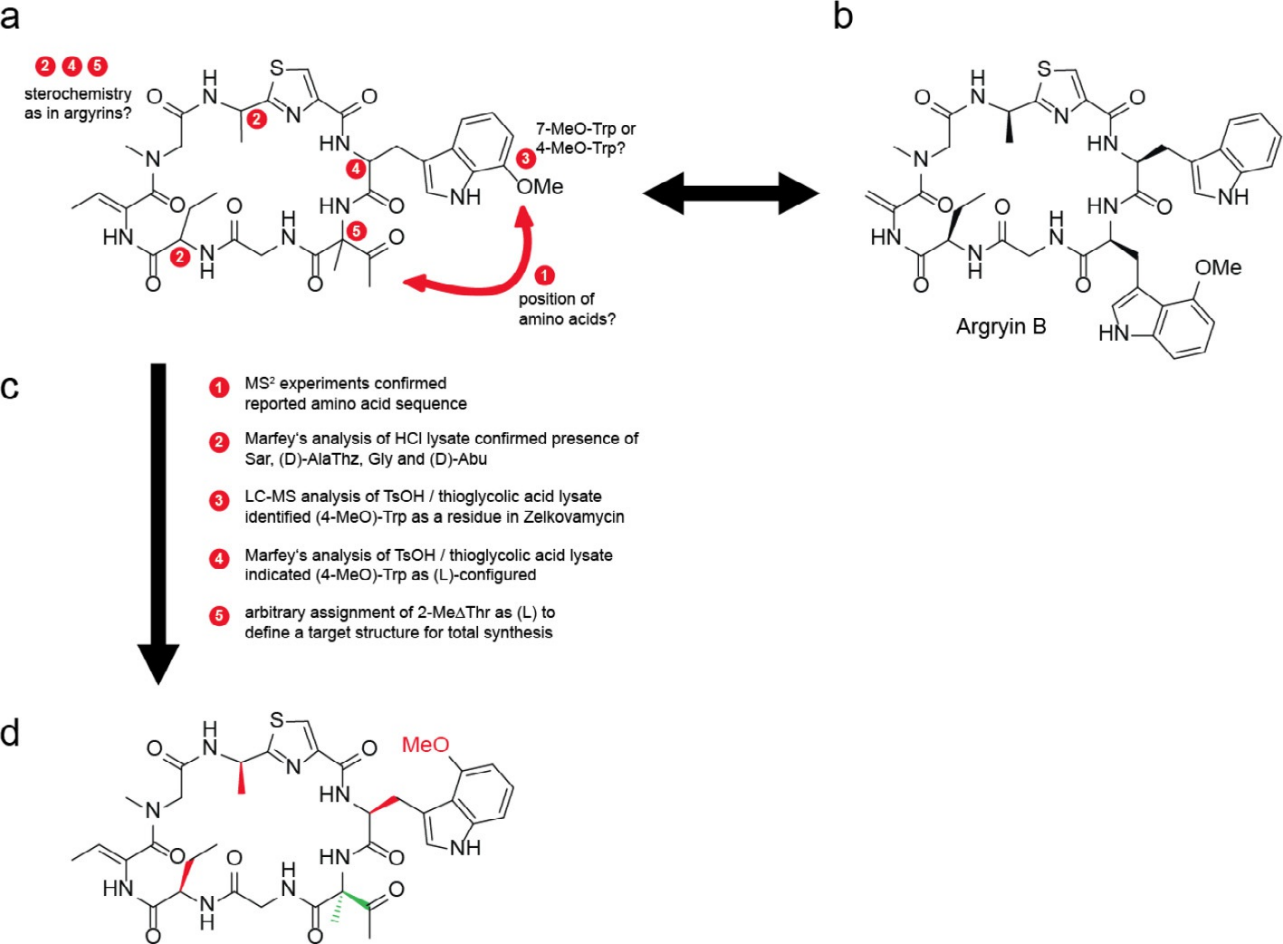
Structural similarities of zelkovamycin and the argyrins and workflow for structure elucidation. (a) Chemical structure of zelkovamycin as published in the original structure elucidation study.^3^ The numbered bullet points indicate positions in the molecule where the connectivity or stereochemistry was uncertain. Comparison of this structure with argyrin B (b) as a representative member of the argyrin natural product family reveal substantial structural similarities but also differences that needed to be clarified for a full assignment and confirmation of the chemical structure of zelkovamycin.4a, 4b, 4c (c) Overview of the different experiments that were carried out to define the structure and stereochemistry of zelkovamycin. (d) Revised structure of zelkovamycin (**1**) with elucidated stereochemistry as deduced from the above experiments. The configuration of the (2‐Me)ΔThr residue (green color) could however not be established and was therefore arbitrarily set to (l) to define a target structure for the subsequent total synthesis. Later chemical synthesis then confirmed that this proposed structure is the correct chemical structure of zelkovamycin.

In addition to the lack of knowledge of its bioactivities, the exact molecular structure of zelkovamycin is however still elusive. The Ōmura group reported zelkovamycin as a cycloheptapeptide built up in the given order from the amino acids glycine (Gly), 2‐amino butyric acid (Abu), (*Z*)‐dehydrobutyric acid ((*Z*)‐Dhb), sarcosine (Sar), an alanine‐thiazole amino acid (AlaThz), 7‐methoxy tryptophan ((7‐MeO)Trp) and 2‐methyl dehydro threonine ((2‐Me)ΔThr), a so far unprecedented amino acid in a peptide NP (Figure 1 a); the stereochemistry of the different amino acids however was not reported. Moreover, despite the striking structural similarities between zelkovamycin and the argyrins, there are also distinct differences: Most obviously, argyrins typically feature a 4‐methoxy tryptophan ((4‐MeO)Trp) or an unsubstituted Trp, but never a (7‐MeO)Trp residue, as reported for zelkovamycin.4a, 4b, 4c Moreover, the position of the (4‐MeO)Trp residue in the argyrins is occupied by the unusual (2‐Me)ΔThr moiety in zelkovamycin, while the (7‐MeO)Trp residue of zelkovamycin is at the site of an unmodified Trp residue in the argyrins. Therefore, more detailed structural studies that enable stereochemical assignment and a confirmation of its overall structure are required.

Chemical synthesis is a well‐established strategy to unambiguously elucidate the molecular structure of a NP, including cyclopeptides.^5^ So far, chemical synthesis of zelkovamycin has not yet been achieved which contrasts with the argyrin NP family for which a few total syntheses have already been established.^6^ In order to unambiguously establish the molecular structure of zelkovamycin and to characterize its biological activities, we describe in this study a set of structure elucidation experiments, a total synthesis and bioassays that 1) resulted in a structural revision and full stereochemical assignment of zelkovamycin, 2) characterized zelkovamycin as a member of the argyrin NP family, and 3) revealed zelkovamycin as an inhibitor of oxidative phosphorylation (OXPHOS).

We started our investigations by re‐analyzing zelkovamycin's overall amino acid sequence. With a small sample of isolated zelkovamycin at hand, we performed an MS^2^ experiment and annotated the resulting spectrum with the software mMass using either the originally reported amino acid sequence (AlaThz|(MeO)Trp|(2‐Me)ΔThr|Gly|Abu|Dhb|Sar; cyclic) or a more argyrin‐like sequence in which the (MeO)Trp group was placed on the position of the (2‐Me)ΔThr moiety (AlaThz|(2‐Me)ΔThr|(MeO)Trp|Gly|Abu|Dhb|Sar; cyclic).^7^ As the published sequence enabled assignment of 66 % vs. 57 % of the total intensity (Figure S1 and Supporting File 1) and most of the unassigned peaks were intense b_2_‐b_4_ ions containing the motif AlaThz|(MeO)Trp and Sar|AlaThz|(MeO)Trp (Δ mass smaller than 0.001 ppm; Table S1), this analysis strongly supported the reported sequence connectivity (Figure 1 c).

Nevertheless, these experiments did not allow to differentiate between (4‐MeO)Trp and (7‐MeO)Trp residues due to their identical *m*/*z* value or to define the stereochemistry of the different amino acids. We thus performed a LC‐MS‐based Marfey's analysis after HCl hydrolysis of which enabled detection and stereochemical assignment of the amino acids Gly, (d)‐Abu, Sar and (d)‐AlaThz (Figure 1 c and Figures S2 and S3); of note, the stereochemical assignments were thereby verified by direct comparison with corresponding chemically synthesized Marfey's modified amino acids (Figures S3 and S4). The other amino acids of zelkovamycin however could not be detected, neither as a Marfey derivative nor in an unmodified form. Detection of the (MeO)Trp and *Z*‐Dhb residue is hampered by their limited chemical stability during hydrolysis conditions.^8^ The failed identification of a Marfey's‐modified (2‐Me)ΔThr residue however might be caused by its low reactivity due to its quaternary sp^3^‐center at the α‐position. For defining the so far missing exact constitution and configuration of the (MeO)Trp residue, we thus resorted to a milder hydrolysis protocol and treated isolated zelkovamycin with 3 N TsOH in the presence of 2 % thioglycolic acid at 110 °C for 18 h.^8^ Subsequent LC‐MS analysis of this hydrolysate in conjunction with co‐injection experiments with chemically synthesized (l)‐(4‐MeO)Trp or (l)‐(7‐MeO)Trp revealed the presence of a (4‐MeO)Trp residue (as found in all other argyrins known so far, Figure S5). A subsequent Marfey's analysis, using also a chemically synthesized Marf‐(l)‐(4‐MeO)Trp as a control, then demonstrated that the (4‐MeO)Trp moiety has the (l)‐configuration (Figure S6).

Altogether, these experiments led to a refined and partially revised proposal for the chemical structure of zelkovamycin in which all amino acids displayed “argyrin‐like” features (constitution and stereochemistry, Figure 1 d). Unfortunately, the stereochemistry of the (2‐Me)ΔThr residue however remained elusive. To obtain a full assignment and proof of the chemical structure of zelkovamycin and to provide further material for subsequent biological assays, we thus arbitrarily assigned a (l)‐configuration to this amino acid, inter alia because the argyrins feature an (l)‐amino acid on this position (Figure 1 d). We then embarked for a total synthesis using a flexible, modular synthesis approach. Accordingly, zelkovamycin was formally divided into three peptidic fragments (fragments A–C) with comparable structural complexity and with a C‐terminal amino acid resistant to racemization during fragment coupling conditions (Figure 2). As we suspected that the carbonyl moiety of the (2‐Me)ΔThr residue might cause problems during the synthesis, we decided to install it at the last stage of the synthesis via oxidation from the corresponding alcohol.

**Figure 2:**
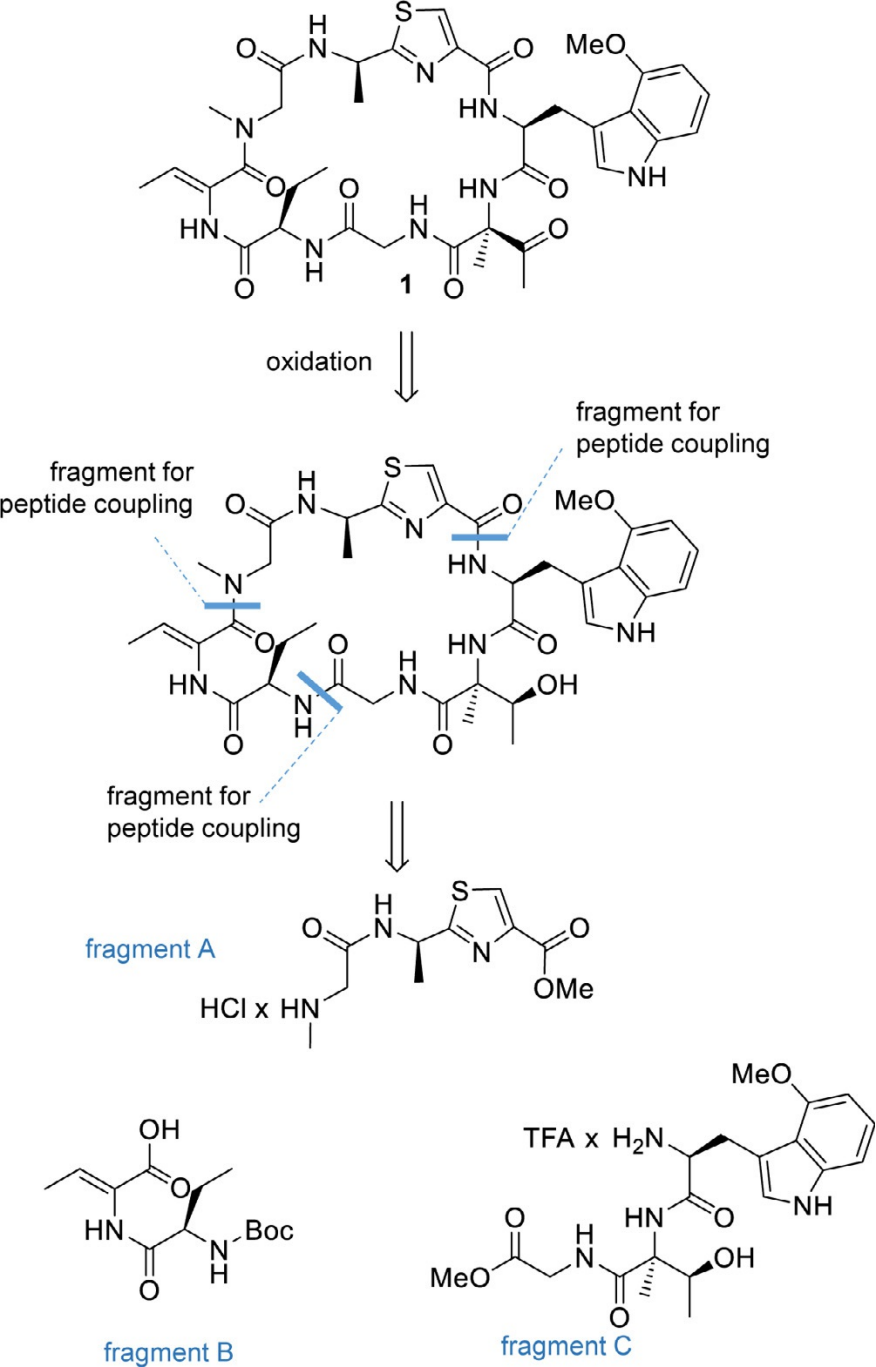
Overview on the envisaged synthesis route to zelkovamycin. Zelkovamycin was retrosynthetically divided into three fragments A–C with comparable structural complexity and a C‐terminal amino acid insusceptible to racemization upon activation during peptide couplings.

For synthesis of fragment A, we first generated Boc‐(d)‐AlaThz‐OMe via the Arndt methodology (Figure S7).^9^ Cleavage of the Boc protecting group with 4 m HCl in dioxane, followed by a coupling of Boc‐Sar‐OH using EDC/HOBt activation and another Boc deprotection step then finalized fragment A (Figure 3 a). Fragment B was obtained from H‐(d)‐Dab‐OH that was Boc‐protected, followed by an EDC/HOBt‐mediated coupling with H‐Thr‐OMe (Figure 3 b). This dipeptide was then converted into Boc‐(d)‐Dab‐(*Z*)‐Dhb‐OH via an EDC/Cu^I^Cl‐mediated water elimination using a modified protocol from the Albericio group,^10^ followed by a methyl ester hydrolysis with LiOH. For the synthesis of fragment C, the generation of the (2‐Me)Thr and (4‐MeO)Trp building block was first required. As the retrosynthetic plan involved a late‐stage oxidation of the hydroxyl residue of the (2‐Me)Thr moiety, the stereochemistry at this site can be freely chosen. We therefore decided to synthesize *allo*‐(l)‐(2‐Me)Thr‐OMe due to an available synthesis protocol from the Goodman group that was slightly adapted to our needs (Figure S8).^11^ The synthesis of the required (l)‐Boc‐(4‐MeO)Trp‐OH building block was carried out according to a Pd‐catalyzed heteroannulation approach from the Zhu group (Figure S9).^12^ With these compounds in hands, we then synthesized fragment C by first coupling Boc‐(l)‐(4‐MeO)Trp‐OH with *allo*‐(l)‐(2‐Me)Thr‐OMe (Figure 3 c) by pre‐treatment of *allo*‐(l)‐H‐(2‐Me)Thr‐OMe with trimethyl silyl chloride, followed by a treatment with HATU/HOAt. Saponification of the methyl ester with LiOH, a subsequent coupling with H‐Gly‐OMe and Boc‐deprotection with TFA in DCM then completed the synthesis of fragment C. Assembly of the different fragments into zelkovamycin started with fragment A (H‐Sar‐(d)‐AlaThz‐OMe) that was coupled to fragment B (Boc‐(d)‐Abu‐(*Z*)‐Dhb‐OH) with EDC/HOBt (Figure 4). The C‐terminal ester of the resulting linear polypeptide was hydrolyzed and this intermediate was coupled to fragment C ((l)‐H‐(4‐MeO)Trp‐*allo*‐(l)‐(2‐Me)Thr‐Gly‐OH) featuring the same coupling conditions. Cyclization was then achieved by a sequence of reactions consisting of methyl ester hydrolysis, acidic cleavage of the N‐terminal Boc‐protecting group and EDC/HOBt‐mediated intramolecular peptide coupling under high dilution conditions. After Dess–Martin periodinane oxidation, the desired final product **1** was obtained. A comparison of the spectral data of synthesized zelkovamycin with those reported for isolated zelkovamycin as well as MS^2^ fragmentation, LC‐MS co‐injection and NMR co‐mixture experiments with isolated zelkovamycin revealed that our structure assignment, including the (l)‐stereochemistry of the (2‐Me)ΔThr residue, was correct (Figures S1, S10 and S11).^3^ Zelkovamycin thus has the chemical structure shown in Figure 1 d.

**Figure 3:**
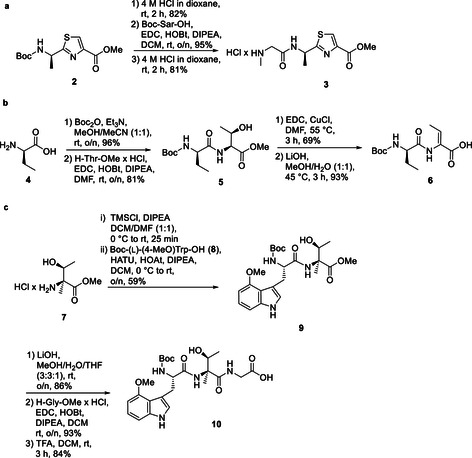
Synthesis of the three fragments A (a), B (b) and C (c) for assembly of the zelkovamycin core ring system.

**Figure 4:**
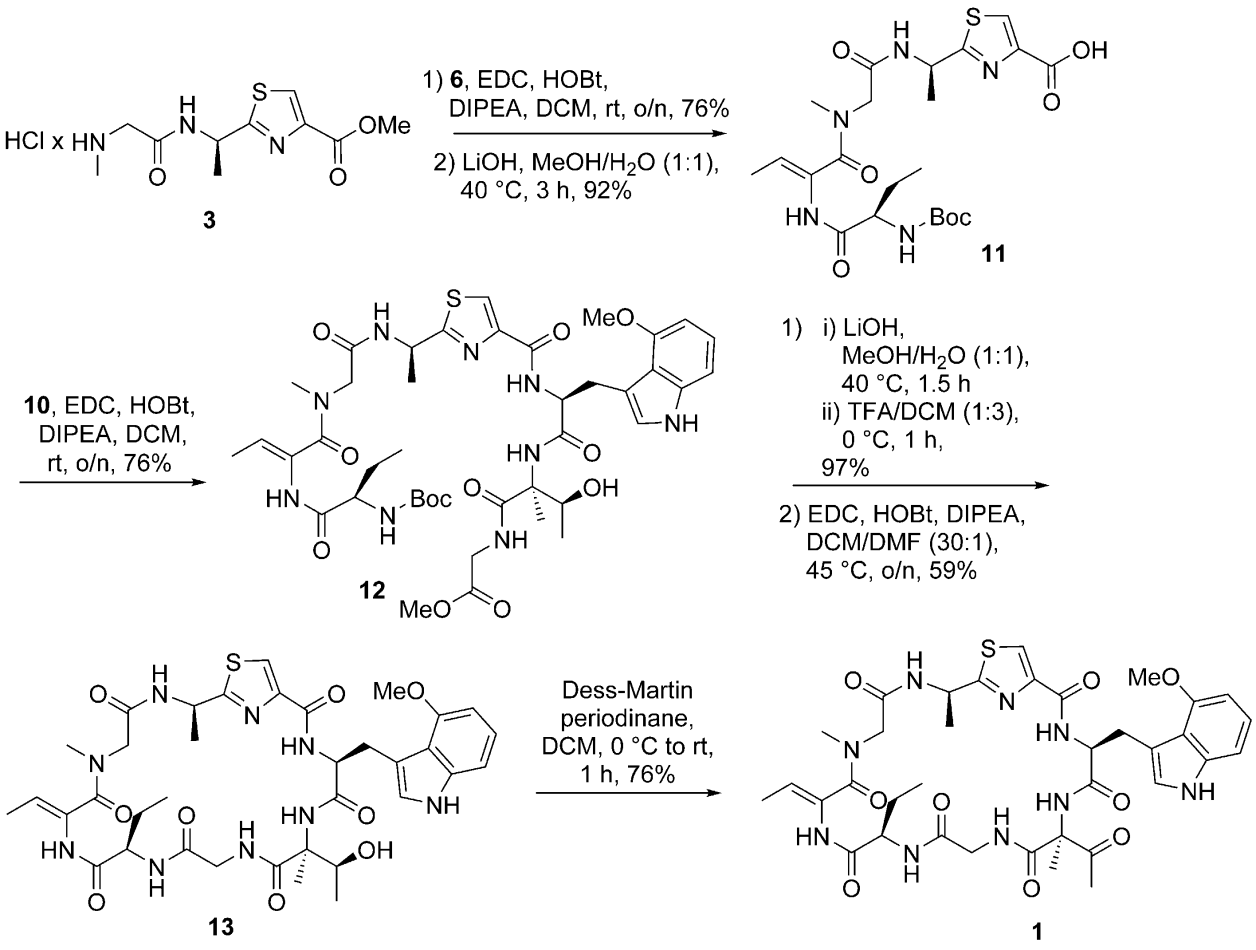
Assembly of the fragments to the final natural product zelkovamycin (**1**).

We next started to evaluate the bioactivity of zelkovamycin in a HeLa cell culture and noted an apparent color change of the medium pH indicator (phenol red) due to an unexpected extracellular acidification after 48 h treatment with 20 μm zelkovamycin (Figure 5 a). We could mimic these effects by an alternative addition of 2.5 μm Carbonyl cyanide *m*‐chlorophenyl hydrazine (CCCP) or 1 μm Antimycin A (AMA), both inhibitors of oxidative phosphorylation (OXPHOS) in mitochondria.^13^ As extracellular acidification may be caused by increased secretion of lactate,^14^we measured the medium lactate concentrations after 48 h treatment with 20 μm zelkovamycin and found increased levels in HeLa and an even stronger rise in SH‐SY5Y cells in a time‐dependent manner (Figure 5 b).

**Figure 5:**
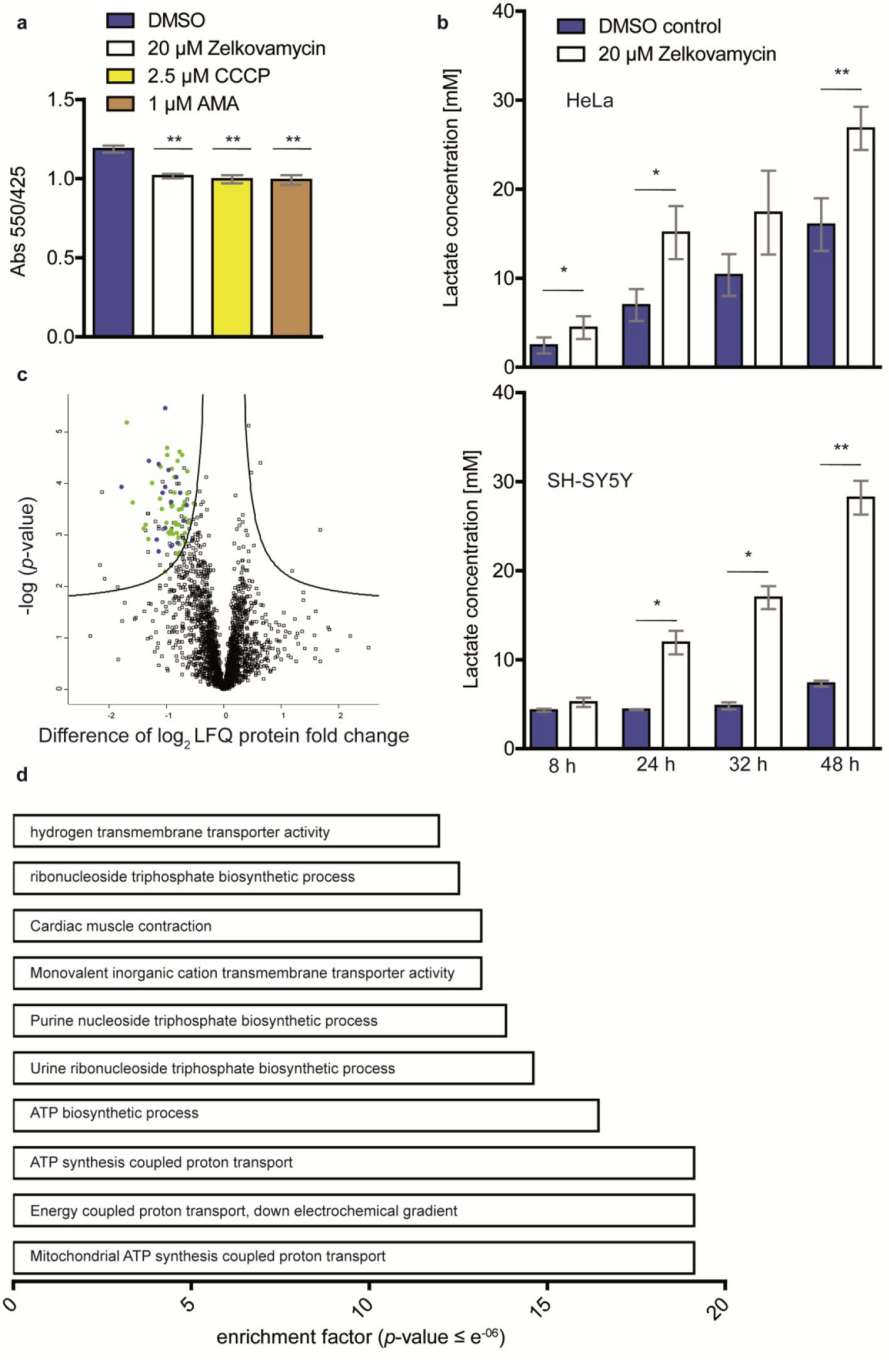
Zelkovamycin induces lactate reflux and lower abundances of mitochondrial proteins. (a) Zelkovamycin leads to extracellular medium acidification, which can be mimicked by the OXPHOS inhibitors CCCP and AMA.^13^ HeLa cells were treated with indicated compounds for 48 h. Acidification was measured by absorbance (Abs) of phenol red (550 nm, 425 nm=reference). Error bars indicate standard deviation from three replicates and significance was tested by Student's *t*‐test. ** indicates a *p* value <0.01. (b) Zelkovamycin increases extracellular medium lactate levels in a time‐dependent manner. Lactate levels of culture medium (DMEM: +10 % FCS and 1 % penicillin/streptomycin) were measured by an (l)‐lactate assay kit after cells (upper panel: HeLa, lower panel: SH‐SY5Y) were grown for 48 h and an aliquot of 100 μL was analyzed at the indicated time points. Error bars indicate standard deviation from three replicates and significance was tested by Student's *t*‐test. * and ** indicates a *p*‐value <0.05 or 0.01, respectively. (c) Zelkovamycin treatment results in lower protein abundance of mitochondrial proteins after label‐free MS‐based proteome analysis. HeLa cells were treated with 20 μm zelkovamycin for 16 h and applied to MS‐based proteome analysis. The black line indicates significantly (permutation‐based FDR=0.01, s0=0.1, *n*=6) regulated proteins (squares). The *y* axis indicates the −log *p*‐value, while the *x* axis indicates the protein abundance difference in log_2_‐fold change (LFQ). Proteins on the upper left of the significance line are down‐regulated, while proteins on the upper right of the significance line are up‐regulated. Proteins colored in green are annotated to be localized in mitochondria or interact with mitochondrial function. Blue colored proteins are related with ATP biosynthesis. (d) Enrichment analysis of all significantly down‐regulated proteins. For enrichment analysis, gene names were annotated by Gene Ontology (GO) and Kyoto Encyclopedia of Genes and Genomes (KEGG) terms. The *p* value was calculated by Benjamini–Hochberg‐FDR. Enrichment factor indicates increased occurrence of GO or KEGG term compared to approximate occurrence. Displayed are top ten enriched GO or KEGG terms.

These experiments thus indicate a zelkovamycin‐induced metabolic switch towards a more glycolytic phenotype. To corroborate this with an unbiased approach, we performed a full proteome analysis of HeLa cells after a 16 h treatment with 20 μm zelkovamycin. The comparison of the resulting label‐free quantification (LFQ) profile with the corresponding DMSO treatment resulted in the identification of 2424 proteins of which 101 were significantly less and five more abundant after zelkovamycin treatment (Table S2). Intriguingly, almost 60 % of the less abundant proteins are annotated to be localized in mitochondria or linked to mitochondrial function (Figure 5 c). A Gene Ontology (GO) and Kyoto Encyclopedia of Genes and Genomes (KEGG) enrichment analysis of these proteins furthermore indicates that most of these proteins are associated with ATP biosynthesis, suggesting that zelkovamycin affects mitochondrial function in eukaryotic cells (Figure 5 d).^14^, ^15^

All performed assays so far thus indicate that long‐term zelkovamycin treatment leads to an impairment of mitochondrial function. To deduce if zelkovamycin however also directly affects mitochondria, we next tested its impact on cellular OXPHOS levels that can be displayed by real‐time cell measurements of the mitochondrial oxygen consumption rate (OCR, “seahorse assay”). Zelkovamycin application to HeLa cells and the more OXPHOS‐dependent SH‐SY5Y and WM3734 melanoma cells resulted in an immediate concentration‐dependent OCR reduction (Figure 6 a and Figure S12).^16^ Zelkovamycin has a unique chemical structure which is distinct from the related argyrins by the presence of the (2‐Me)ΔThr and the position of the (4‐MeO)Trp moiety in the macrocycle. We therefore wondered if the observed OXPHOS effects are zelkovamycin‐specific and depend on these unusual moieties. Accordingly, we repeated the OCR measurements with argryin B as an argryin representative with a potential mitochondrial target (elongation factor G) and two additional zelkovamycin analogues **13** ((2‐Me)ΔThr→(2‐Me)Thr) and **14** ((4‐MeO)Trp→Trp, Figure 6 b).4g, 4h In contrast to zelkovamycin, none of them displayed significant OXPHOS reduction at the tested 10 μm concentration (Figure 6 c) nor led to higher lactate levels in cell medium (Figure S13).

**Figure 6:**
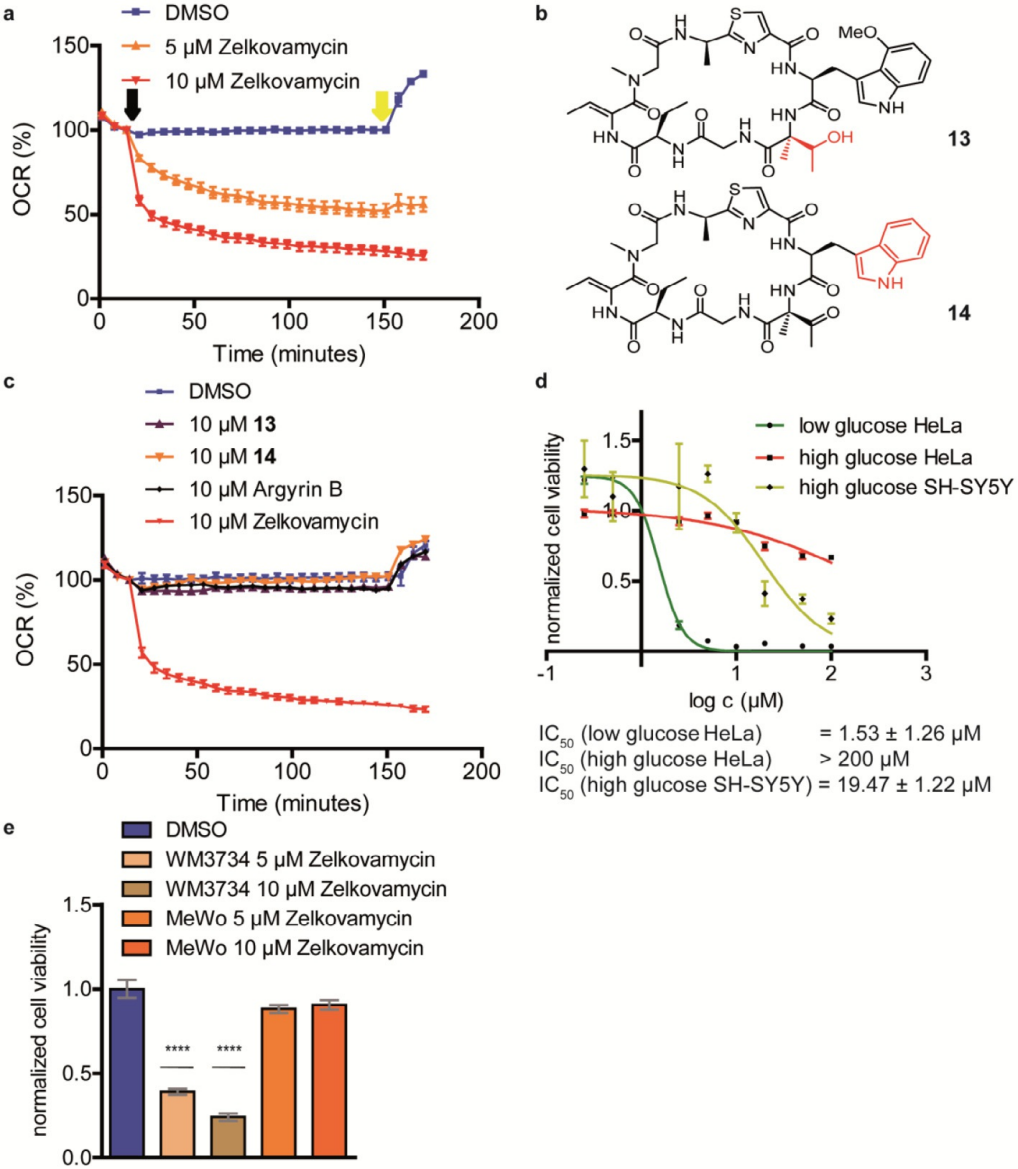
Zelkovamycin inhibits OXPHOS in different cell lines. (a) Zelkovamycin inhibits OCR in a concentration‐dependent manner. OCR of HeLa cells was measured after application of zelkovamycin (black arrow). To depict RRC, FCCP was applied (yellow arrow). Error bars indicate standard deviation from 6–14 replicates. (b) Chemical structure of two zelkovamycin derivatives (**13** and **14**) used for structure–activity relationship studies. Red colored moieties indicate structural differences to zelkovamycin. (c) Argyrin B or zelkovamycin derivatives **13** and **14** do not affect OCR. OCR of HeLa cells was measured after application of the compounds (black arrow). To depict RRC, FCCP was applied (yellow arrow). Error bars indicate standard deviation from 6–14 replicates. (d) Cell viability of SH‐SY5Y and HeLa cells at high or low glucose concentrations determined by a MTT assay in the concentration range of 0.25–100 μm zelkovamycin and normalized to DMSO control. Error bars indicate standard deviation from four replicates. (e) Cell viability of the OXPHOS‐dependent melanoma WM3734 and non‐susceptible MeWo melanoma cell line determined by an MTT assay and normalized to DMSO. Error bars indicate standard deviation from four replicates and significance was tested by Student's *t*‐test. ****indicates a *p* value <0.0001.

We thus showed that zelkovamycin is a direct OXPHOS inhibitor. As impairment of mitochondrial function should also lead to reduced cell viability, we tested the effect of zelkovamycin on 293T, HepG2, HeLa Kyoto, HeLa, HCT116 cells that were only weakly affected after 48 h treatment, and SH‐SY5Y neuroblastoma cells, for which cell viability was reduced by more than 50 % already at 20 μm zelkovamycin (Figure S14). This finding is in accordance with the different metabolic needs of SH‐SY5Y cells that gain their cellular energy mostly by mitochondrial OXPHOS, thereby also explaining the observed higher lactate levels (Figure 5 b), whereas the other five cancer cells rely on cytosol‐located glycolysis.^17^ Accordingly, zelkovamycin treatment of HeLa cells in a glucose‐deprived and thus glycolysis‐restrained cell culture medium (4.5 mm instead of 25 mm glucose) resulted in a>100‐fold reduction of cell viability (from an IC_50_>200 μm in glucose‐rich medium to 0.5 μm in glucose‐deprived medium, Figure 6 d). Of note, SH‐SY5Y cells, in accordance with their stronger dependence on OXPHOS, displayed an IC_50_ of 19.5 μm in glucose‐rich medium while they did not grow at all on glucose‐deprived medium. Finally, cell viability after 72 h of the OXPHOS‐dependent melanoma cell line WM3734 was decreased by 75 % at 10 μm zelkovamycin or 60 % at 5 μm zelkovamycin, while no effect was observed on MeWo melanoma control cells that can metabolize glutamine as a mitochondrial‐alternative energy source (Figure 6 e).^16^

Overall, our studies therefore clarified and revised the chemical structure of zelkovamycin, demonstrating that it is a so far unrecognized member of the argyrin NP family. Moreover, we established its first chemical synthesis, thereby allowing the future chemical synthesis of customized analogues, for example, for structure–activity relationship studies. Finally, we demonstrated that zelkovamycin is an OXPHOS inhibitor which may offer possibilities for developing compounds with potential chemical biology or clinical use.^18^ We anticipate that these findings demonstrate zelkovamycin as a promising argyrin NP for future research, for example, of its direct molecular target or unusual biosynthesis.4c, 19

## Conflict of interest

The authors declare no conflict of interest.

## Supporting information

As a service to our authors and readers, this journal provides supporting information supplied by the authors. Such materials are peer reviewed and may be re‐organized for online delivery, but are not copy‐edited or typeset. Technical support issues arising from supporting information (other than missing files) should be addressed to the authors.

Supplementary
