# Supplementary material for: Zelkovamycin is an OXPHOS Inhibitory Member of the Argyrin Natural Product Family
Source: Chemistry. 2020 Jun 17;26(39):8524–31. doi: 10.1002/chem.202001577 (PMC7383741; doi:10.1002/chem.202001577)

Zelkovamycin is an OXPHOS inhibitory member of the argyrin natural product family

Daniel Krahn[a], Geronimo Heilmann[a], Felix C. E. Vogel[c, †], Chrisovalantis Papadopoulos[b], Susanne Zweerink[a, ‖], Farnusch Kaschani[a], Hemmo Meyer[b], Alexander Roesch[c], Markus Kaiser[a]

[a] Chemische Biologie, Universität Duisburg-Essen, ZMB, Fakultät für Biologie, Universitätsstr. 2, 45117 Essen, Germany

[b] Molekularbiologie I, Universität Duisburg-Essen, ZMB, Fakultät für Biologie, Universitätsstr. 2, 45117 Essen, Germany

[c] Department of Dermatology, University Hospital Essen, West German Cancer Center, University Duisburg-Essen and the German Cancer Consortium (DKTK)

† Current address: Division of Tumor Metabolism and Microenvironment, German Cancer Research Center (DKFZ), Im Neuenheimer Feld 280, 69120 Heidelberg, Germany

‖ Current address: University of Cologne, Faculty of Medicine and University Hospital of Cologne, Department of Gastroenterology and Hepatology, Kerpener Str. 62, 50937 Cologne, Germany

Content

**Supporting Figures**	3 - 19
**Supporting Procedures**	20 - 78
**Supporting References**	79 - 80
**^{1}H and ^{13}C NMR spectra**	81 - 117

Supporting Figures

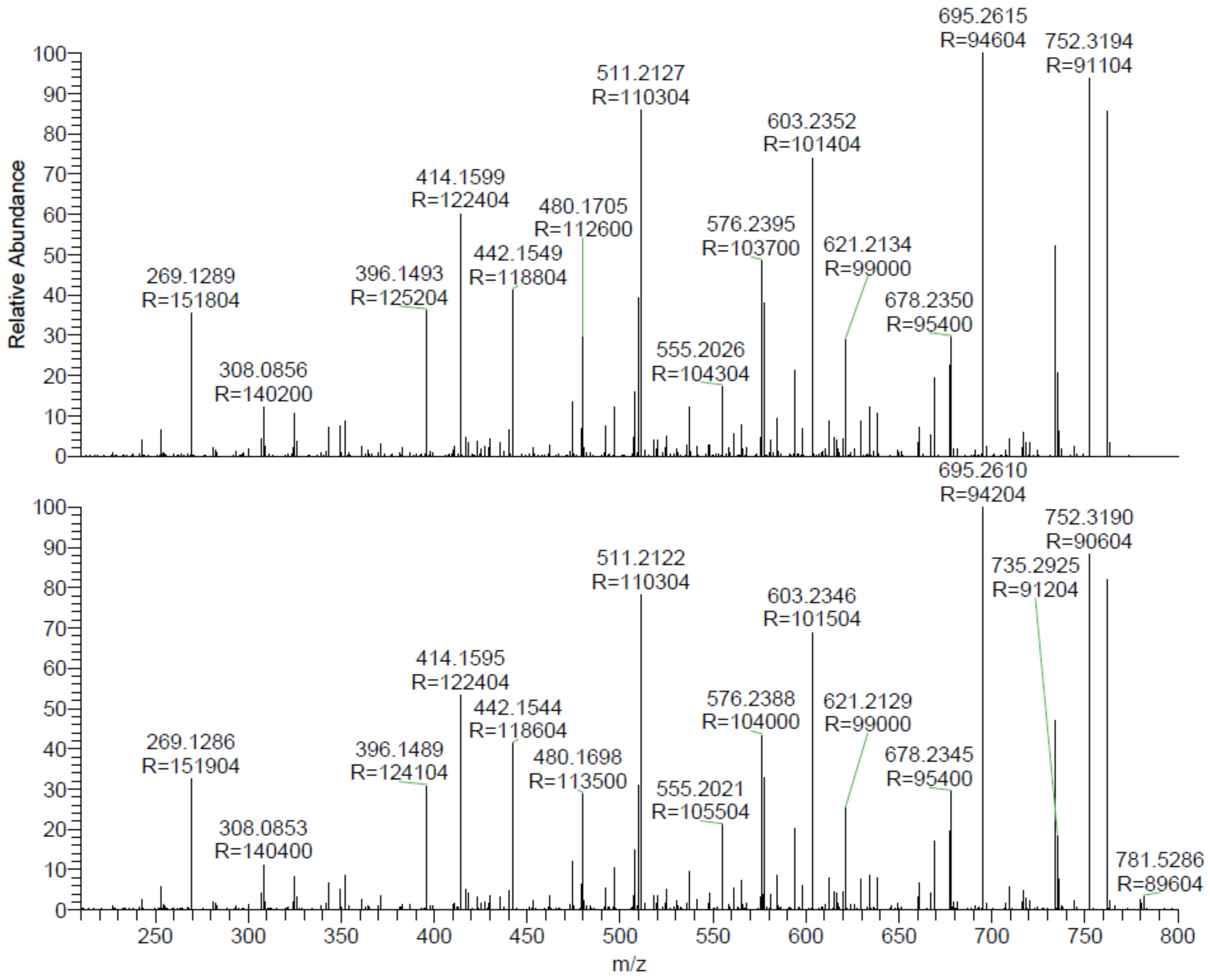

Supporting Figure 1. CID35 spectra of isolated (upper panel) and synthesized (lower panel) Zelkovamycin.

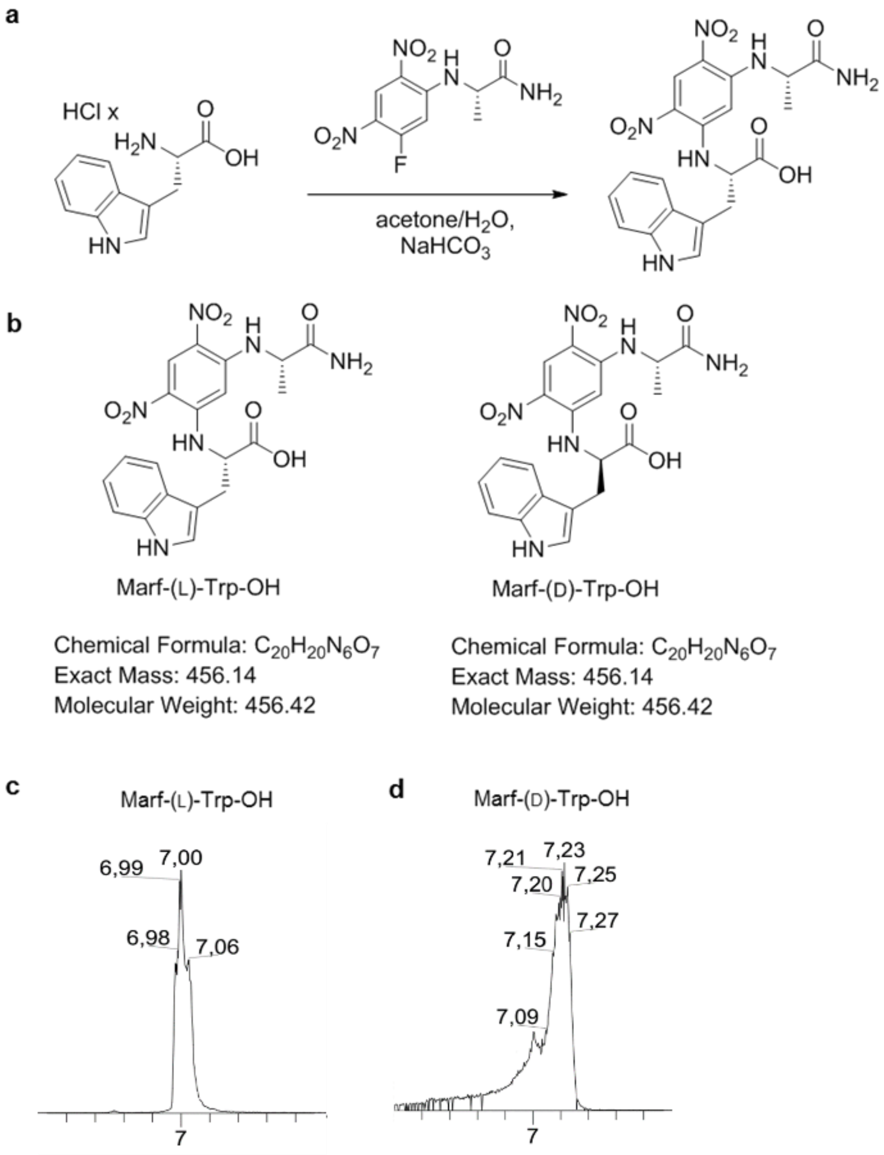

Supporting Figure 2. Establishment and validation of LC-MS settings for reliable separation of Marfey's modified (L) and (D) amino acids.

Depicted in (**a**) is a schematic representation of the reaction leading to Marfey's modified amino acids. (**b**) Chemical structures of Marf-(L)-Trp-OH (left panel) and Marf-(D)-Trp-OH (right panel). In (**c**) are depicted the extracted-ion chromatograms for the analysis of Marfey's modified Marf-(L)-Trp-OH and (**d**) of Marf-(D)-Trp-OH (mass range for both cases *m/z* = 456.00-457.50; corresponding to the $[M+H]^+$ signal of Marf-(L)-Trp-OH or Marf-(D)-Trp-OH).

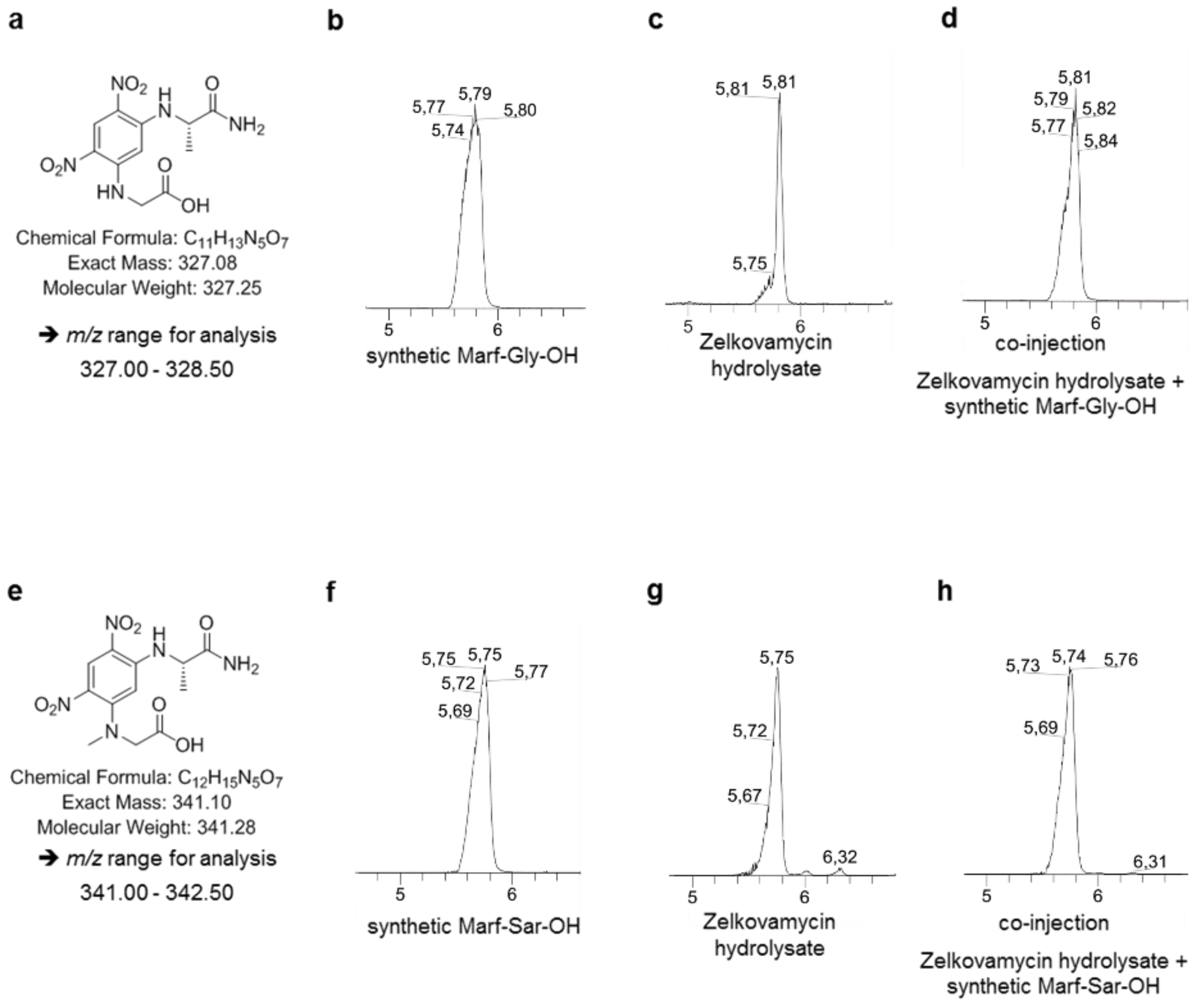

Supporting Figure 3. Marfey's analysis of the 6 N HCl hydrolysate of Zelkovamycin and comparison with the Marf-Gly-OH and Marf-Sar-OH standards.

Depicted in (**a**) is the chemical structure of Marf-Gly-OH and in (**b**) to (**d**) are the relevant parts of the extracted-ion chromatograms for the analysis of Marf-Gly-OH (mass range m/z = 327.00 - 328.50; corresponding to the $[M+H]^+$ signal of Marf-Gly-OH) with synthetic Marf-Gly-OH standard in (**b**), Marfey's modified 6 N HCl hydrolysate of Zelkovamycin in (**c**) and co-injection of synthetic Marf-Gly-OH and Marfey's modified 6 N HCl hydrolysate of Zelkovamycin in (**d**).

Depicted in (**e**) is the chemical structure of Marf-Sar-OH and in (**f**) to (**h**) are the relevant parts of the extracted-ion chromatograms for the analysis of Marf-Sar-OH (mass range m/z = 341.00-342.50; corresponding to the $[M+H]^+$ signal of Marf-Sar-OH) with synthetic Marf-Sar-OH standard in (**f**), Marfey's modified 6 N HCl hydrolysate of Zelkovamycin in (**g**) and co-injection of synthetic Marf-Sar-OH and Marfey's modified 6 N HCl hydrolysate of Zelkovamycin in (**h**).

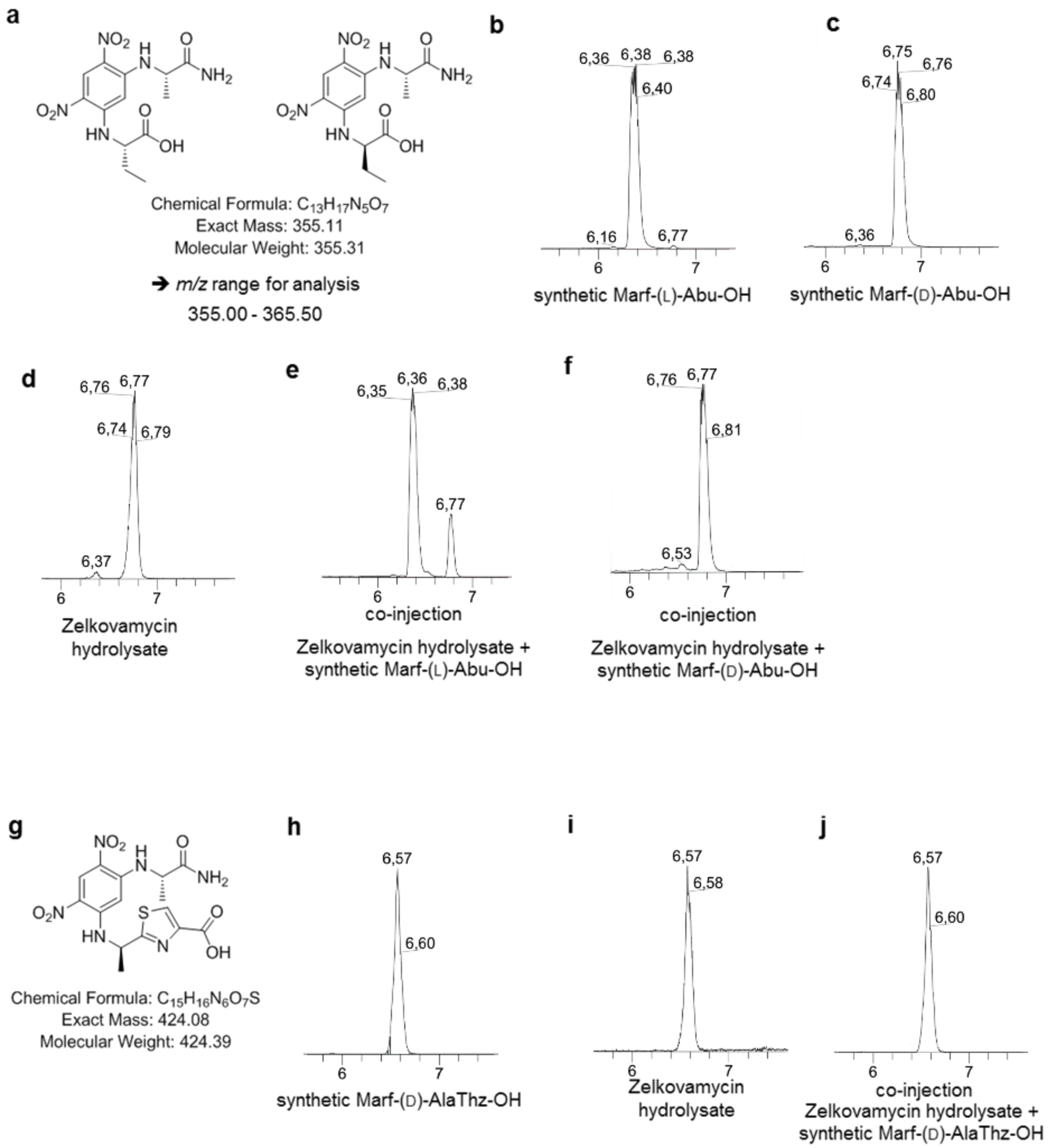

Supporting Figure 4. Marfey's analysis of the 6 N HCl hydrolysate of Zelkovamycin and comparison with the Marf-(L)-Abu-OH and Marf-(D)-Abu-OH as well as Marf-(D)-AlaThz-OH standards.

Depicted in (**a**) is the chemical structure of Marf-(L)-Abu-OH and Marf-(D)-Abu-OH and in (**b**) to (**f**) are the relevant parts of the extracted-ion chromatograms for the analysis of Marf-(L)-Abu-OH and Marf-(D)-Abu-OH (mass range *m/z* = 355.00-356.50; corresponding to the $[M+H]^+$ signal of Marf-(L)-Abu-OH or Marf-(D)-Abu-OH) with synthetic Marf-(L)-Abu-OH standard in (**b**), Marf-(D)-Abu-OH standard in (**c**), Marfey's modified 6 N HCl hydrolysate of Zelkovamycin in (**d**), co-injection of synthetic Marf-(L)-

Abu-OH and Marfey’s modified 6 N HCl hydrolysate of Zelkovamycin in (**e**) and co-injection of synthetic Marf-(D)-Abu-OH and Marfey’s modified 6 N HCl hydrolysate of Zelkovamycin in (**f**).

Depicted in (**g**) is the chemical structure of Marf-(D)-AlaThz-OH and in (**h**) to (**j**) are the relevant parts of the extracted-ion chromatograms for the analysis of Marf-(D)-AlaThz-OH (mass range *m/z* = 424.00-425.50; corresponding to the $[M+H]^+$ signal of Marf-(D)-AlaThz-OH) with synthetic Marf-(D)-AlaThz-OH standard in (**h**), Marfey’s modified 6 N HCl hydrolysate of Zelkovamycin in (**i**) and co-injection of synthetic Marf-(D)-AlaThz-OH and Marfey’s modified 6 N HCl hydrolysate of Zelkovamycin in (**j**).

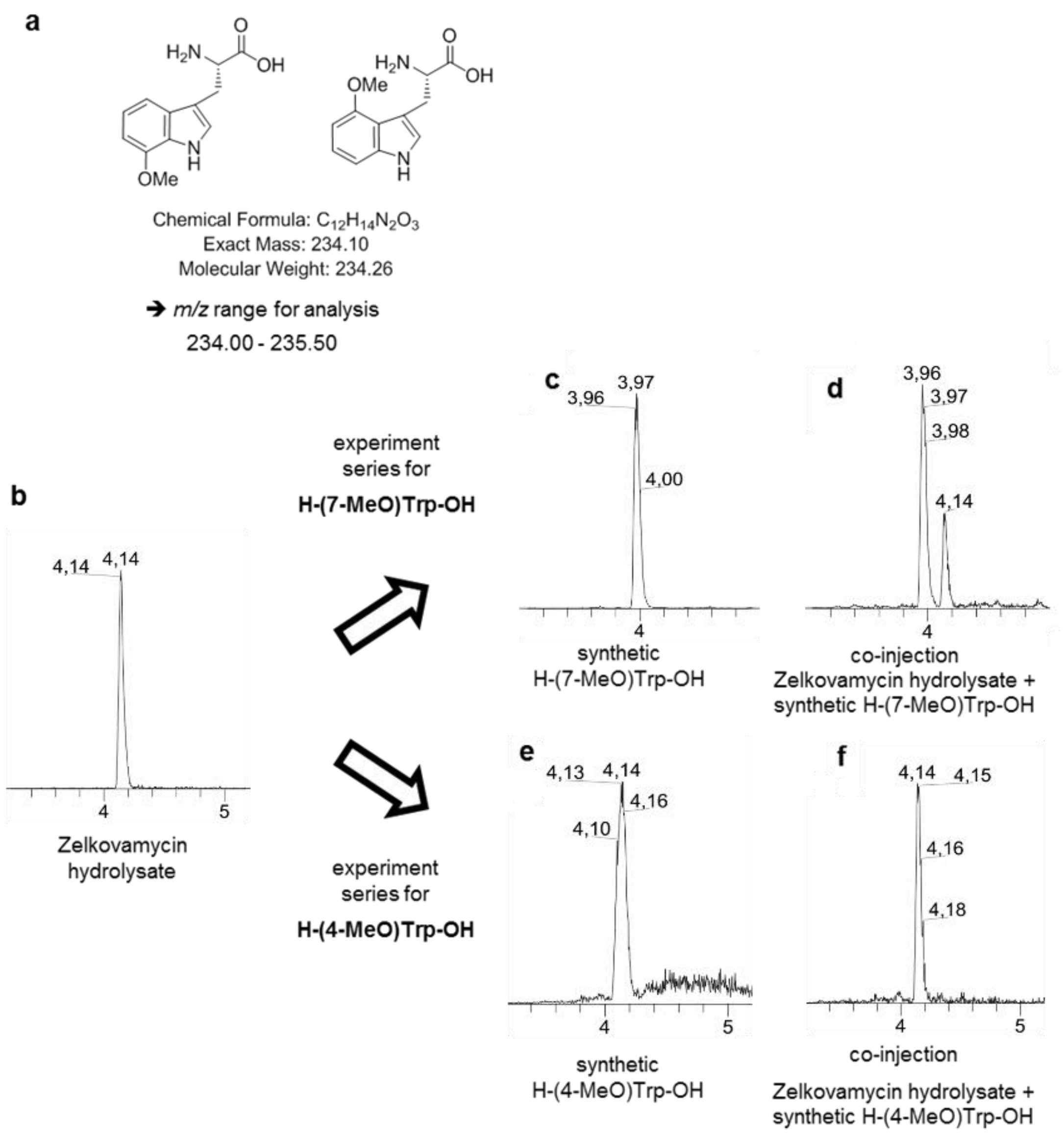

Supporting Figure 5. Identification of H-(4-MeO)Trp-OH as an amino acid building block of Zelkovamycin.

Depicted are in (**a**) the chemical structure of H-(7-MeO)Trp-OH (left panel) and H-(4-MeO)Trp-OH (right panel) and the relevant parts of the extracted-ion chromatograms (mass range *m/z* = 234.00-236.00; corresponding to the $[M+H]^+$ signal of H-(4-MeO)Trp-OH or H-(7-MeO)Trp-OH) of the following samples: (**b**) LC-MS analysis of 3 N TsOH / 2% thioglycolic acid hydrolysate of isolated Zelkovamycin; (**c**) LC-MS analysis of synthetic (L)-H-(7-MeO)Trp-OH after treatment with 3 N TsOH / 2% thioglycolic acid; (**d**) LC-MS analysis of a co-injection of 3 N TsOH / 2% thioglycolic acid hydrolysate of isolated Zelkovamycin and of synthetic (L)-H-(7-MeO)Trp-OH after treatment with 3 N TsOH / 2% thioglycolic acid; (**d**) LC-MS analysis of synthetic (L)-H-(4-MeO)Trp-OH after treatment with 3 N TsOH / 2% thioglycolic acid; (**e**) LC-MS

analysis of a co-injection of 3 N TsOH / 2% thioglycolic acid hydrolysate of isolated Zelkovamycin and of synthetic (L)-H-(7-MeO)Trp-OH after treatment with 3 N TsOH / 2% thioglycolic acid.

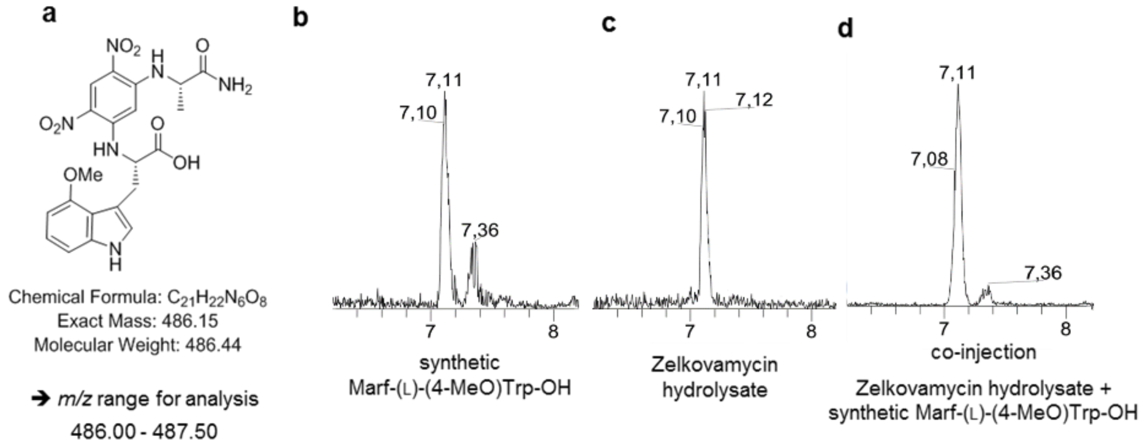

Supporting Figure 6. Marfey's analysis indicates a (L)-stereochemistry for the (4-MeO)Trp residue in Zelkovamycin.

Marfey's analysis of the 3 N TsOH / 2% thioglycolic acid lysate of Zelkovamycin and comparison with a Marf-(L)-(4-MeO)Trp-OH was carried out to confirm the (L)-stereochemistry of the (4-MeO)Trp residue. Depicted in (**a**) is the chemical structure of the Marf-(L)-(4-MeO)Trp-OH standard and in (**b**) to (**c**) the relevant parts of the extracted-ion chromatograms for the analysis of Marf-(L)-(4-MeO)Trp-OH (mass range *m/z* = 486.00-487.50; corresponding to the $[M+H]^+$ signal of Marf-(L)-(4-MeO)Trp-OH) with synthetic Marf-(L)-(4-MeO)Trp-OH standard in (**b**), the Marfey's modified 3 N TsOH / 2% thioglycolic acid hydrolysate of Zelkovamycin in (**c**) and the co-injection of synthetic Marf-(L)-(4-MeO)Trp-OH and Marfey's modified 3 N TsOH / 2% thioglycolic acid hydrolysate of Zelkovamycin in (**d**).

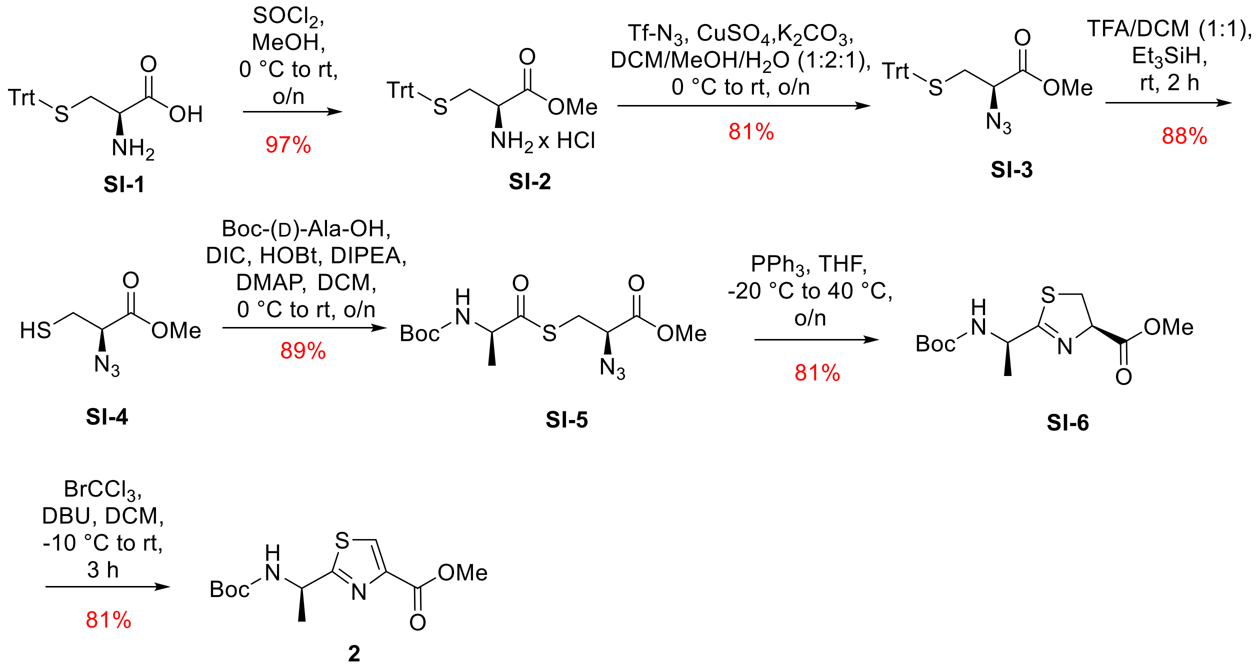

Supporting Figure 7. Overview on the chemical synthesis of the fragment A building block Boc-(D)-AlaThz-OH.

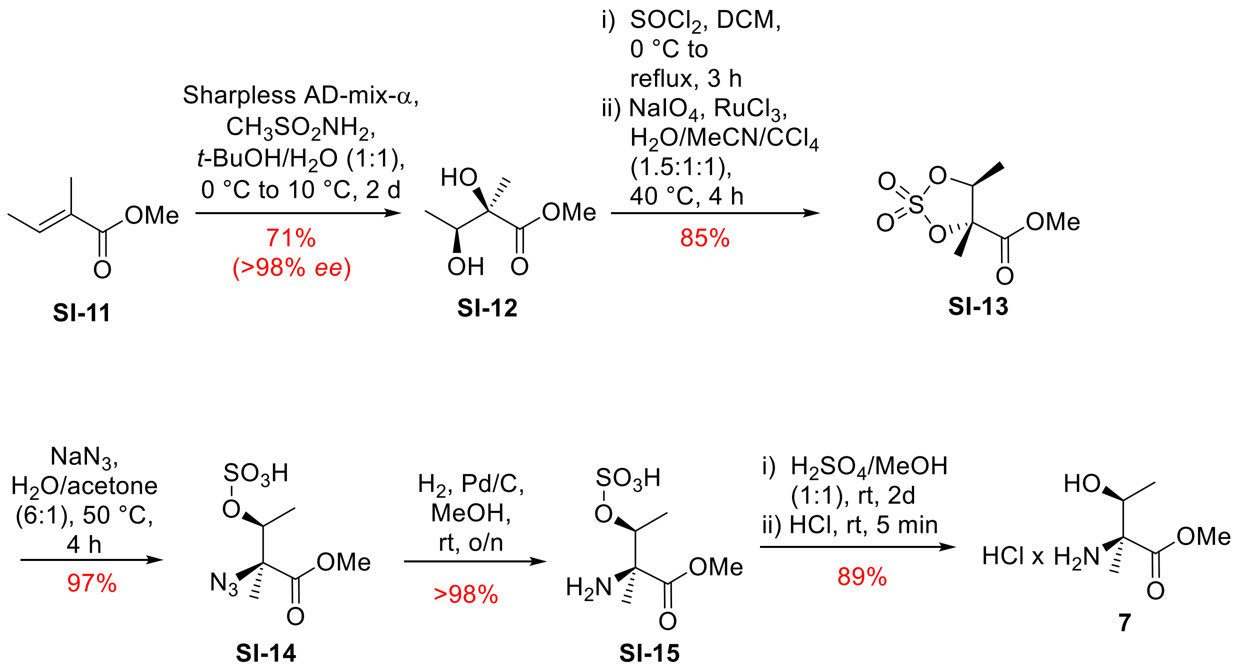

Supporting Figure 8. Chemical synthesis of *allo*-(L)-(2-Me)Thr-OMe (**7**).

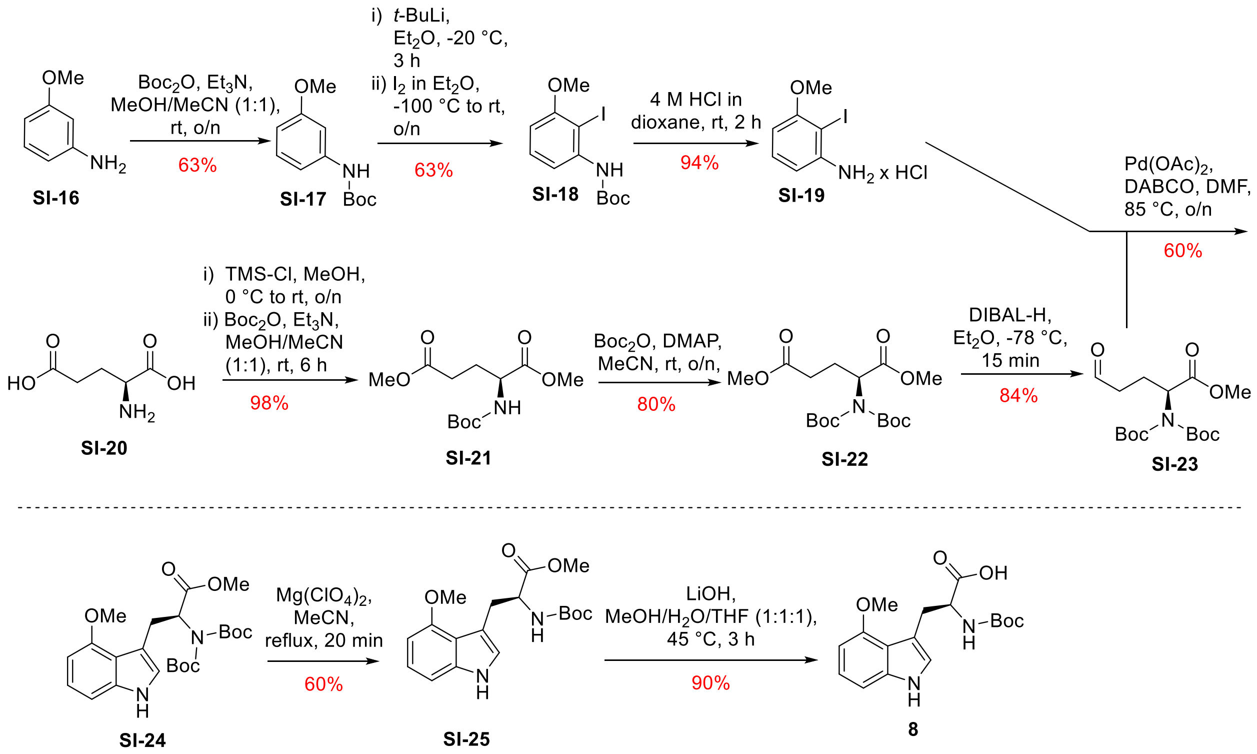

Supporting Figure 9. Chemical synthesis of Boc-(L)-(4-MeO)Trp-OH (**8**).

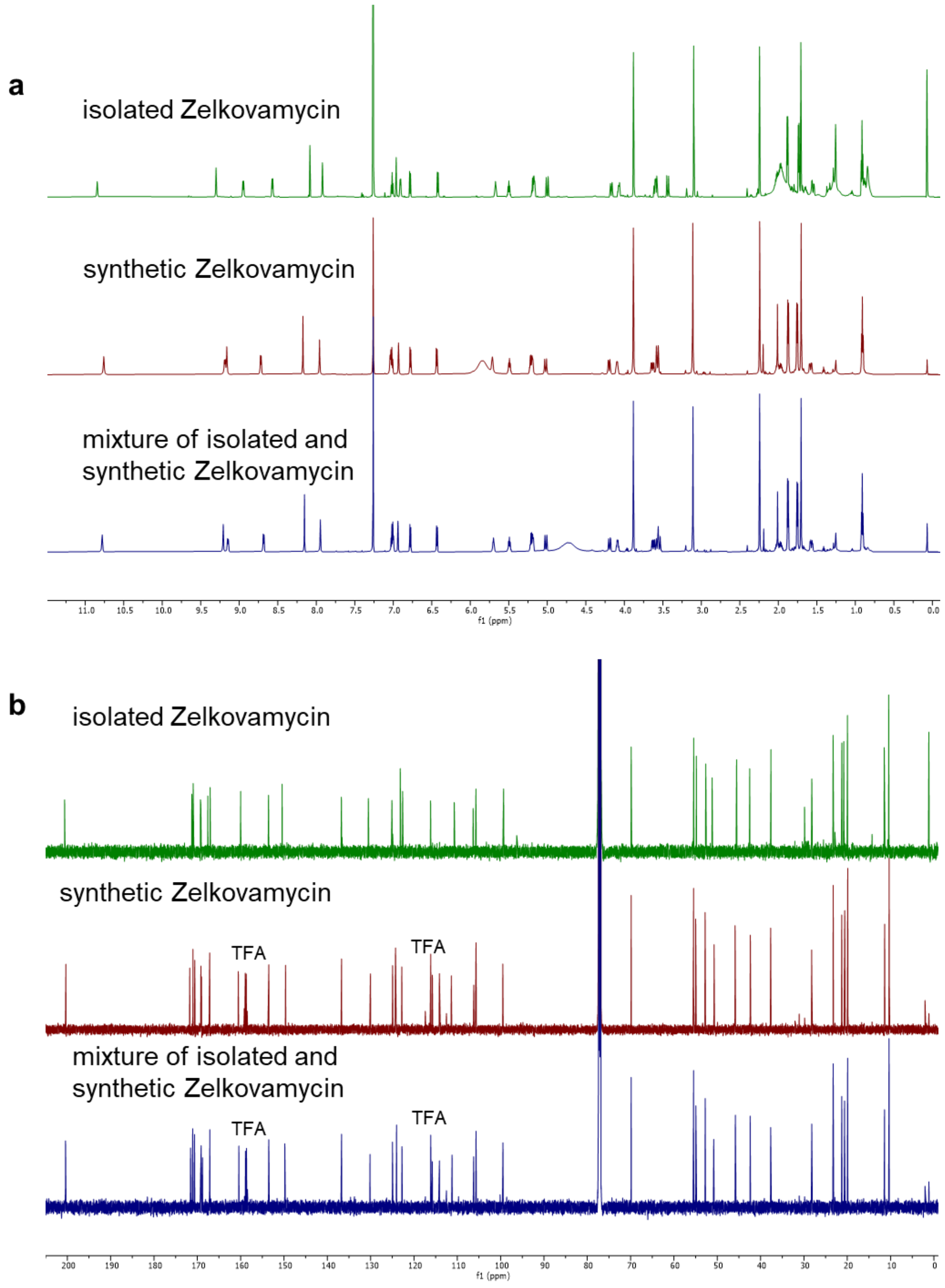

Supporting Figure 10. ^{1}H- and ^{13}C-NMR spectral comparison of synthesized and isolated Zelkovamycin.

Depicted is the ^{1}H-NMR (**a**) and ^{13}C-NMR (**b**) spectrum of isolated Zelkovamycin (blue) synthesized Zelkovamycin (green) and of a roughly (1:1) mixture of synthesized and isolated Zelkvamycin (red) in DMSO-d_6.

Please note that the isolated and synthetic Zelkovamycin samples differ in trifluoroacetate levels due to the purification procedure, resulting in slightly different

chemical shifts for some peaks; the NMR spectra of the (1:1) mixture of isolated and synthetic Zelkovamycin in these cases however does not result in two sets of peaks but only of an ‘intermediate’ peak as typical for an identical compound under different solvent conditions (e.g. as the most extreme case, AlaThz amide proton is in isolated Zelkovamycin at 8.95 ppm, in synthetic Zelkovamycin at 9.19 ppm and in the (1:1) mixture at 9.15 ppm).

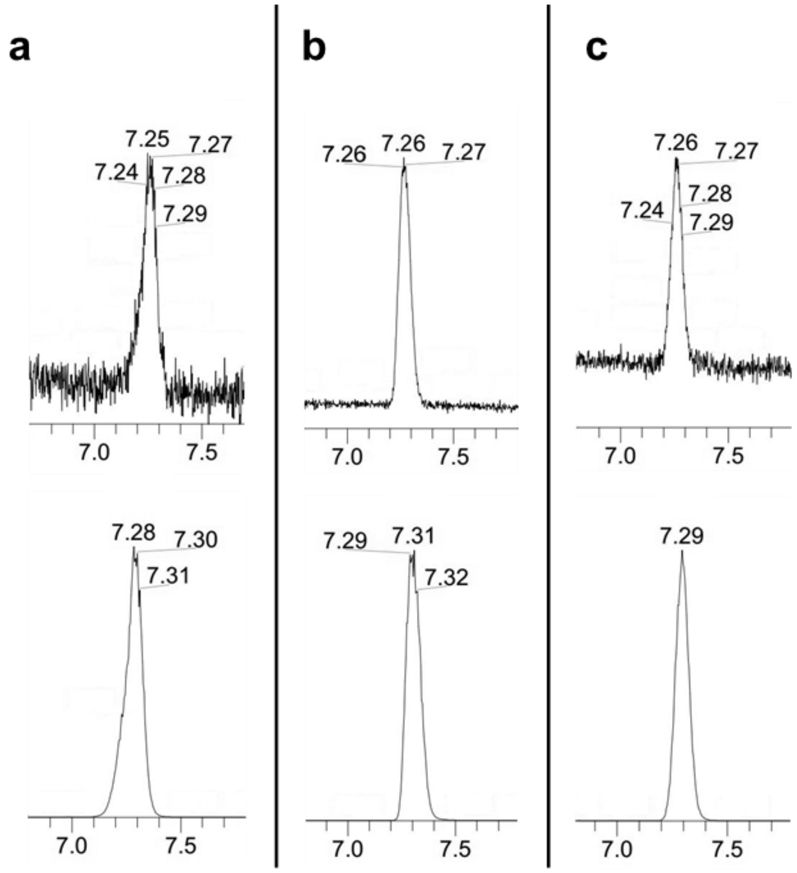

Supporting Figure 11. LC-MS co-injection experiments with isolated and synthesized Zelkovamycin.

The upper panel depicts the corresponding UV trace (214 nm) in the relevant part of the LC-MS spectrum, while the lower panel is the corresponding extracted ion chromatogram. (**a**) LC-MS run of isolated Zelkovamycin. (**b**) LC-MS run of synthesized Zelkovamycin. (**c**) LC-MS run of a coinjection of isolated and synthesized Zelkovamycin.

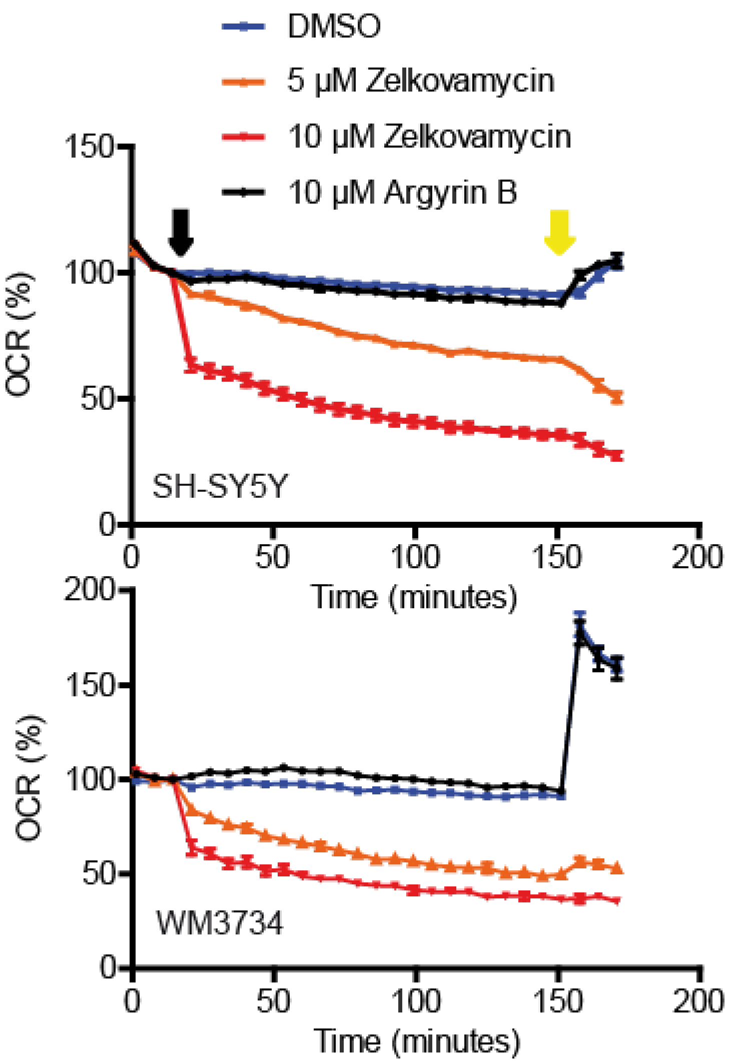

Supporting Figure 12. Zelkovamycin, but not Argyrin B, immediately inhibits OXPHOS in neural and melanoma cell lines.

To determine the effect of Zelkovamycin and Argyrin B on OXPHOS, the OCR of SH-SY5Y or WM3734 cells was measured and normalized after application of Zelkovamycin or Argyrin B (indicated by black arrow). To depict RRC, FCCP was applied (indicated by yellow arrow). OCR was analyzed by SeaHorse XFe96 extracellular flux analyzer (Agilent Technologies). Error bars indicate standard deviation from six – fourteen number of replicates.

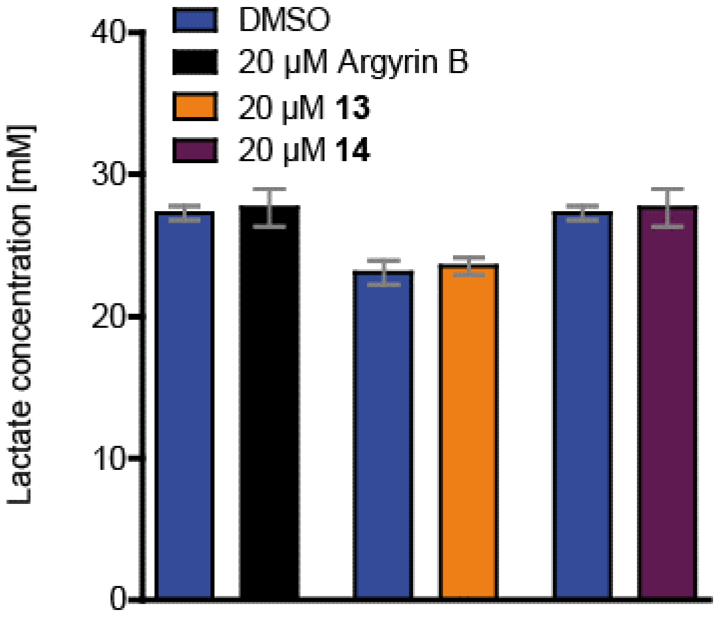

Supporting Figure 13. Argyrin B and Zelkovamycin derivatives **13** (Red-Zelkovamycin) and **14** (ΔMeO-Zelkovamycin) do not increase extracellular lactate concentrations.

HeLa cells were treated with indicated compounds for 48 h. Extracellular lactate concentration is displayed in comparison to corresponding DMSO control. Error bars indicate standard deviation from three number of replicates.

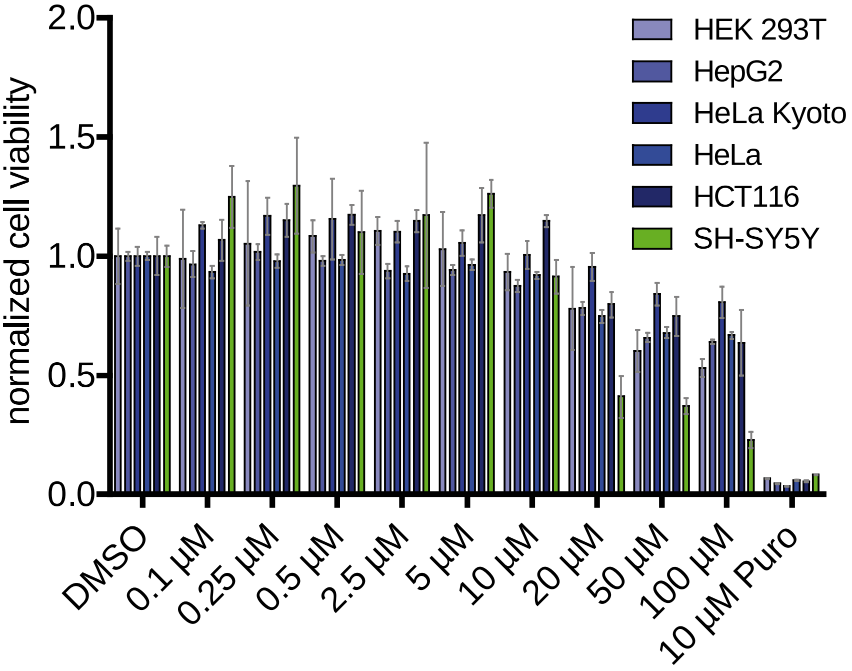

Supporting Figure 14. Cell viability of different cancer cell lines after Zelkovamycin treatment.

Cell viability was determined by MTT assay after treatment with Zelkovamycin at indicated concentrations for 48 h. Cell viability was normalized to corresponding DMSO control. Puromycin (Puro) was used as positive control. Error bars indicate standard deviation from four replicates.

Supporting Procedures

Chemical synthesis of Zelkovamycin (1) and analogues

General

Chemicals and solvents

All chemicals, solvents, reagents and media were purchased from the following companies: Abcam, ABCR, Acros Organics, Alfa Aesar, Bachem, Biomol, Carl Roth, Fluka, TCI Chemicals, Merck, Novabiochem, Iris Biotech, Sigma-Aldrich and VWR and were used without further purification. Anhydrous solvents were purchased from the same suppliers in the highest available quality.

Thin-layer chromatography (TLC)

TLC was carried out on Merck aluminium pre-coated silica gel plats (20 × 20 cm, $60F_{254}$). Spots were detected using UV irradiation at 254 nm or by staining/heating with a developing solution (1.5 g $KMnO_4$, 10 g K_2CO_3 and 1.25 mL of 10% aq. NaOH in 200 mL water).

Flash column chromatography

Normal phase flash column chromatography was performed with silica gel (particle size 35-70 µm, Acros), while reverse flash chromatography was carried out with LiChroprep RP-18 (particle size 40-63 µm, Merck). The corresponding eluent systems are reported in the experimental procedures.

Preparative reversed-phase high performance liquid chromatography (RP-HPLC)

HPLC purifications were performed on a Prominence UFLC HPLC system from Shimadzu, using either a preparative (Phenomenex Luna 5 µm C18, 100 × 21.20 mm RP-C_{18} column and a flow rate of 25 mL min^{-1}) or semi-preparative (Phenomenex Luna 5 µm C18, 100 × 10.00 mm RP-C18 column and a flow rate of 10 mL min^{-1}). As eluents, 0.1% TFA in water and 0.1% TFA in acetonitrile were used. The corresponding gradient programs were individually optimized for each compound purification.

Reversed-phase liquid chromatography-electro spray ionization mass spectrometry (RP-LC-ESI-MS)

LC-MS analyses were performed on a Thermo Scientific LCQ Fleet ESI spectrometer equipped with an Eclipse XDB-C18 (5 µm) column from Agilent (peak detection at 210 and 280 nm). Positive mode measurements were performed with a linear gradient of solvent B (0.1% formic acid in acetonitrile) in solvent A (0.1 formic acid in water) with a flow rate of 1 mL min^{-1} and the following gradient program: 0 min / 10% B → 1 min / 10% B → 10 min / 100% B → 12 min / 100% B → 12.1 min / 10% B → 15 min / 10% B. Negative mode measurements were performed with a linear gradient of solvent C (5 mM ammonium acetate in acetonitrile) in solvent D (5 mM ammonium acetate in water) with a flow rate of 1 mL min^{-1} and the following gradient program: 0 min / 10% C → 1 min / 10% C → 10 min / 100% C → 12 min / 100% C → 12.1 min / 10% C → 15 min / 10% C.

High resolution electro spray ionization mass spectrometry (HR-ESI-MS)

All HRMS analyses were performed on a Bruker maXis 4G Q-TOF ESI spectrometer using the direct flow injection method.

Nuclear magnetic resonance spectroscopy (NMR)

Nuclear magnetic resonance spectra were recorded on a Bruker Avance II 400 system with 400 MHz for ^{1}H and 100 MHz for ^{13}C NMR or a Bruker Avance II 700 MHz system with 700 MHz for ^{1}H and 176 MHz for ^{13}C NMR. The NMR spectra are reported in the

following manner: ^{1}H NMR: chemical shifts (δ) in ppm calculated with reference to the residual signals of the undeuterated solvent, multiplicity (s, singlet, d, doublet, t, triplet, dd, doublet of doublet, dt doublet of triplet, m, multiplet, b, broad signal), coupling constants (*J*) in Hertz (Hz), and number of protons (H). ^{13}C NMR: chemical shifts (δ) in ppm calculated with reference to the residual signals of the undeuterated solvent.

Optical rotation

Optical rotations were determined on a polartronic universal polarimeter from Schmidt & Haensch. Individual concentrations (in g (100 mL)$^{-1}$), temperatures and solvents for each measurement are given in the experimental section. Optical rotations were measured at the wavelength of 589 nm (the sodium D line).

General procedures

Esterification General Procedure ($SOCl_2$)

To a cooled suspension (0 °C, ice bath) of the carboxylic acid (1.0 eq.) in methanol (or the corresponding alcohol of the desired ester) thionyl chloride (2 eq.) was added slowly, using a droplet funnel. The resulting mixture was stirred 15 min at room temperature and then heated up to reflux until TLC analysis indicated complete conversion (usually 4 h for methyl esters). The solvent was removed under reduced pressure and the crude product was dried overnight under high vacuum. If necessary, further purifications were carried out as described in the corresponding synthesis procedure.

Ester Hydrolysis General Procedure (LiOH)

The ester was dissolved in a mixture of methanol and water (1:1). To this solution lithium hydroxide (3.0 eq.) was added and the resulting reaction mixture was stirred at room temperature until TLC analysis indicated complete conversion. If the starting material did not dissolve completely in methanol and water, a few drops of tetrahydrofuran were added until a clear solution was obtained. The organic solvents

were removed under reduced pressure and the remaining aqueous phase was acidified by the addition of potassium bisulfate (sat., aq.). The aqueous layer was diluted with water and extracted with dichloromethane (5x). The combined organic phases were dried over $MgSO_4$ and concentrated under reduced pressure to afford the desired carboxylic acid. If necessary, further purifications were carried out as described in the corresponding synthesis procedure.

Boc-Protection General Procedure

To a solution of di-*tert*-butyl dicarbonate (1.2 eq.) in acetonitrile and methanol (1:1), triethylamine (2.0 eq.) was added and the mixture was stirred for 15 min at room temperature. The compound containing the amine, dissolved in acetonitrile and methanol (1:1), was added dropwise, using a droplet funnel, to the pre activated solution of di-*tert*-butyl dicarbonate over a time period of 2 h. The resulting mixture was stirred for additional 15 min. The solvent was removed under reduced pressure and the crude product was taken up in ethyl acetate and washed with water (2x) and brine (1x). The organic layer was dried over $MgSO_4$ and concentrated under reduced pressure to afford the desired Boc-protected amine. If necessary, further purifications were carried out as described in the corresponding synthesis procedure.

Boc-Deprotection General Procedure

The Boc-protected amine was treated with hydrogen chloride solution (4 M in 1,4-dioxane) and the resulting suspension was stirred for 1 h at room temperature. The solvent was removed under reduced pressure and the remaining precipitate was dried under high vacuum for additional 3 h to yield the unprotected amine as hydrochloride salt.

Peptide-Coupling General Procedure (EDC)

The amine (usually as hydrochloride salt, 1.2 eq.) and 1-(3-dimethylaminopropyl)-3-ethylcarbodiimide hydrochloride (EDC, 3.0 eq.) were suspended in dichloromethane. To this mixture a solution of the carboxylic acid (1.0 eq.), hydroxybenzotriazole (HOBt, 3.0 eq.) and N,N-diisopropylethylamine (DIPEA, 5.0 eq.) in dichloromethane was

added dropwise. The resulting reaction mixture was stirred at room temperature until TLC analysis indicated full conversion. If the starting material did not dissolve completely in dichloromethane, a few drops of dimethylformamide were added until a clear solution was obtained. The reaction mixture was diluted with dichloromethane and washed with sodium bicarbonate (sat., aq., 2x). The combined aqueous layers were re-extracted with dichloromethane (1x). The combined organic layers were washed with potassium bisulfate (sat., aq., 2x) and the combined aqueous layers (washing solution) were again re-extracted with dichloromethane (1x). The combined organic layers were washed with brine (1x), dried over $MgSO_4$ and concentrated under reduced pressure. If necessary, further purification was carried out as described in the corresponding synthesis procedure.

Peptide-Coupling General Procedure (TMS-Activation)

The amine (1.0 eq.) was suspended under an argon atmosphere in anhydrous dichloromethane. Dry *N*,*N*-diisopropylethylamine (5.0 eq.) was added and the resulting mixture was stirred for 10 min until the starting material completely dissolved (if necessary, a few drops of anhydrous dimethylformamide were added to obtain a clear solution). The solution was cooled to 0 °C (ice bath) and trimethylsilyl chloride (TMSCl, 2.0 eq.) was slowly added. The resulting mixture was stirred 10 min at 0 °C and additional 15 min at room temperature. The reaction solution was again cooled down to 0 °C (ice bath) and a prepared solution of the carboxylic acid (1.2 eq.), 1-hydroxy-7-azabenzotriazole (HOAt, 3.0 eq.), 1-[Bis(dimethylamino)methylene] -1H-1,2,3-triazolo[4,5-b] pyridinium 3-oxid hexafluoro phosphate (HATU, 2.0 eq.) and dry N,N-diisopropylethylamine (DIPEA, 3.0 eq.) in anhydrous dichloromethane was added drop wise. The reaction mixture was allowed to warm up to room temperature gradually and was stirred overnight. The mixture was diluted with dichloromethane and washed with sodium bicarbonate (sat., aq., 2x). The combined aqueous layers were re-extracted with dichloromethane (1x) and the combined organic layers were washed with potassium bisulfate (sat., aq., 2x). The combined aqueous layers (washing solution) were again re-extracted with dichloromethane (1x) and the combined organic layers were washed with brine (1x), dried over $MgSO_4$ and concentrated under reduced pressure. If necessary, further purification was carried out as described in the corresponding synthesis procedure.

Dehydrobutyrine Synthesis General Procedure

The threonine derivative (1.0 eq.), 1-ethyl-3-(3-dimethylaminopropyl)carbodiimide (EDC, 5.0 eq.) and copper(I) chloride (2.7 eq.) were dissolved in dimethylformamide (10 mL) and the reaction mixture was heated up to 55 °C for 3 h. The mixture was concentrated to dryness under reduced pressure and re-dissolved in dichloromethane. The solution was washed with sodium bicarbonate (sat., aq., 2x), potassium bisulfate (sat., aq., 2x) and brine (1x). The organic layer was dried over $MgSO_4$ and concentrated to dryness under reduced pressure. If necessary, further purification was carried out as described in the corresponding synthesis procedure.

Chemical synthesis of Boc-AlaThz-OH

Boc-AlaThz-OH was synthesized according to **Supporting Fig. 8.**

Synthesis of H-Cys(Trt)-OMe hydrochloride salt (SI-2)

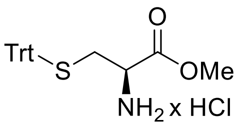

SI-2

H-(L)-Cys(Trt)-OMe hydrochloride salt (**SI-2**) was synthesized following the *Esterification General Procedure ($SOCl_2$)* utilizing H-(L)-Cys(Trt)-OH (**SI-1**, 3.00 g, 8.25 mmol, 1.0 eq.), thionylchloride (1.96 g, 16.51 mmol, 1.2 mL, 2.0 eq.) and methanol (75 mL). H-Cys(Trt)-OMe hydrochloride salt (**SI-2**) was obtained as a colorless solid.

Yield: 3.31 g (8.00 mmol, 97%, colorless solid).

TLC (33% ethyl acetate in cyclohexane): R_f = 0.18.

LC-MS (ESI): t_R = 6.60 min (C_{18}), *m/z* = calcd for $NaC_{23}H_{23}NO_2S^+$ $[M+Na]^+$ 400.13, found 400.11.

HRMS (ESI): *m/z* = calcd for $C_{23}H_{24}NO_2S^+$ $[M+H]^+$ 378.1522, found 378.1522, calcd for $NaC_{23}H_{23}NO_2S^+$ $[M+Na]^+$ 400.1342, found 400.1347.

$[\propto]_{\mathbf{D}}^{\mathbf{23}}$ ([c] = 1 in $CHCl_3$): +82.

^{1}H NMR (400 MHz, $CDCl_3$): δ 8.48 (br, 1H), 7.42-7.39 (m, 6H), 7.30-7.23 (m, 6H), 7.20-7.16 (m, 3H), 3.58 (s, 3H), 3.42 (s, 1H), 2.98-2.87 (m, 2H).

^{13}C NMR (100 MHz, $CDCl_3$): δ 168.0, 143.9, 129.7, 128.4, 127.2, 67.6, 53.6, 52.4, 31.8.

Synthesis of S-Trityl-(L)-azido-cysteine methyl ester (SI-3)

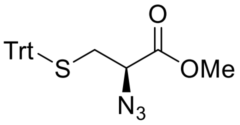

SI-3

Sodium azide (1.72 g, 26.81 mmol, 6.0 eq.) was dissolved in water (5 mL) and cooled down to 0 °C (ice bath). To this solution was slowly added a solution of trifluoromethanesulfonic anhydride (3.78 g, 14.41 mmol, 2.26 mL, 3.0 eq.) in dichloromethane (5 mL). After complete addition the mixture was stirred vigorously for 2 h at 0 °C (ice bath). Potassium carbonate (sat., aq., 15 mL) was added and the mixture was transferred to a separatory funnel and extracted with dichloromethane (3x, 10 mL). The organic phases were combined and used without further purification.

H-Cys(Trt)-OMe hydrochloride salt (**SI-2**, 1.85 g, 4.47 mmol, 1.0 eq.) and copper(II) sulfate pentahydrate (112 mg, 0.45 mmol, 0.1 eq.) were dissolved in water (30 mL). Potassium carbonate (1.85 g, 13.41 mmol, 3.0 eq.) was added and the mixture was cooled down to 0 °C (ice bath). The previously prepared triflyl azide solution was added, followed by addition of methanol (~50 mL) until a homogenous mixture was obtained. The ice bath was removed and the reaction mixture was stirred overnight at room temperature. The organic solvents were removed under reduced pressure and the remaining aqueous layer was extracted with dichloromethane (5x 15 mL). The combined organic layers were dried over $MgSO_4$, concentrated under reduced pressure and purified by flash column chromatography (5% ethyl acetate in cyclohexane) to afford S-Trityl-(L)-azido-cysteine methyl ester (**SI-3**) as a pale yellow solid.

Yield: 1.46 g (3.62 mmol, 81%, pale yellow solid).

TLC (33% ethyl acetate in cyclohexane): R_f = 0.79;

(15% ethyl acetate in cyclohexane): R_f = 0.66.

LC-MS (ESI): t_R=11.39 min (C_{18}); *m/z* = calcd for $NaC_{23}H_{21}N_3O_2S^+$ $[M+Na]^+$ 426.12, found 426.08.

HRMS (ESI): *m/z* = calcd for $NaC_{23}H_{21}N_3O_2S^+$ $[M+Na]^+$ 426.1247, found 426.1247.

$[\propto]_D^{24}$ ([c] = 1 in $CHCl_3$): -9.

^{1}H NMR (400 MHz, $CDCl_3$): δ 7.46-7.43 (m, 6H), 7.33-7.28 (m, 6H), 7.26-7.21 (m, 3H), 3.71 (s, 3H), 3.20 (dd, *J* = 5.9, 8.1, 1H), 2.68 (dd, *J* = 5.9, 13.4, 1H), 2.54 (dd, *J* = 8.1, 13.4, 1H).

^{13}C NMR (100 MHz, $CDCl_3$): δ 169.5, 144.4, 129.7, 128.3, 127.1, 67.5, 61.5, 53.0, 33.3.

Synthesis of Azido-(L)-cysteine methyl ester (SI-4)

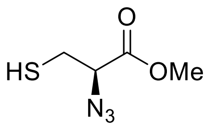

SI-4

S-Trityl-(L)-azido-cysteine methyl ester (**SI-3**, 365 mg, 0.90 mmol, 1 eq.) and triethylsilane (210 mg, 1.81 mmol, 289 µL, 2 eq.) were dissolved in dichloromethane (1.5 mL) and cooled down to 0 °C (ice bath). To this solution was slowly added trifluoroacetic acid (1.5 mL). After complete addition the mixture was warmed up to room temperature and stirred for 2 h. The solvent was removed under reduced pressure and the remaining crude product was coevaporated with toluene (3x 7 mL) to remove trifluoroacetic acid residues. Due to the high volatility of the product, evaporation and drying under high vacuum was carried out carefully. The crude azido-cysteine methyl ester (**SI-4**) was obtained as a colorless oil and was used without further purification.

Yield: 128 mg (0.79 mmol, 88%, colorless oil).

TLC (5% ethyl acetate in cyclohexane): R_f = 0.29.

$[\propto]_D^{23}$ ([c] = 2 in $CHCl_3$): -11.5.

^{1}H NMR (400 MHz, $CDCl_3$): δ 3.83 (s, 3H), 2.96-2.81 (m, 2H), 1.73 (t, *J* = 8.8, 1H).

^{13}C NMR (100 MHz, $CDCl_3$): δ 169.2, 64.3, 53.1, 26.4.

Synthesis methyl (*R*)- 2-azido- 3 - (((*tert*-butoxycarbonyl)- (D) -alanyl) thio) propanoate (SI-5)

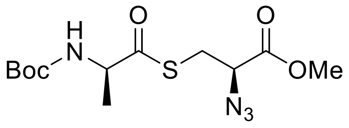

SI-5

Boc-(D)-Ala-OH (39 mg, 0.20 mmol, 1.1 eq.), HOBt (38 mg, 0.28 mmol, 1.5 eq.) and DMAP (2 mg, 0.02 mmol, 0.1 eq.) were suspended in dichloromethane and cooled down to 0 °C (ice bath). To this mixture, DIC (35 mg, 0.28 mmol, 43 µL, 1.5 eq.) and DIPEA (72 mg, 0.56 mmol, 97 µL, 3.0 eq.) were added and the mixture was stirred for 30 min at 0 °C. Azido-(L)-cysteine methyl ester (**SI-4**, 30 mg, 0.19 mmol, 1.0 eq.) was added. The resulting reaction mixture was stirred overnight and allowed to warm up to room temperature gradually. The solvent was removed under reduced pressure and the crude product was purified by flash column chromatography (5% ethyl acetate in cyclohexane) to yield the title compound **SI-5** as a colorless oil.

Yield: 55 mg (0.17 mmol, 89%, colorless oil).

TLC (33% ethyl acetate in cyclohexane): R_f = 0.31

(5% ethyl acetate in cyclohexane): R_f = 0.14.

LC-MS (ESI): t_R = 8.58 min (C_{18}); *m/z* = calcd for $C_7H_{12}N_4O_3S^+$ [M-Boc+H]$^+$ 232.06, found 232.75, calcd for $C_{19}H_{33}N_8O_8S_2^+$ [2M-Boc+H]$^+$ 565.19, found 564.58, calcd for $NaC_{12}H_{20}N_4O_5S^+$ [M+Na]$^+$ 355.10, found 355.09.

HRMS (ESI): *m/z* = calcd for $NaC_{12}H_{20}N_4O_5S^+$ [M+Na]$^+$ 355.1047, found 355.1077.

$[\propto]_D^{24}$ ([c] = 1 in $CHCl_3$): -18.

^{1}H NMR (400 MHz, $CDCl_3$): δ 4.97 (d, *J* = 7.0, 1H), 4.37 (t, *J* = 7.2, 1H), 4.09-4.04 (m, 1H), 3.80 (s, 3H), 3.38-3.31 (m, 1H), 3.21-3.11 (m, 1H), 1.44 (s, 9H), 1.37 (d, *J* = 7.3, 3H).

^{13}C NMR (100 MHz, $CDCl_3$): δ 201.2, 169.1, 155.0, 80.6, 61.4, 56.5, 53.1, 30.0, 28.4, 18.5.

Synthesis methyl (*R*)-2-((*R*)-1-((*tert*-butoxycarbonyl)amino)ethyl)-4,5-dihydrothiazole-4-carboxylate (SI-6)

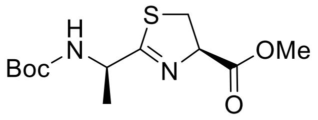

SI-6

Methyl (*R*)-2-azido-3-(((*tert*-butoxycarbonyl)-(D)-alanyl)thio)propanoate (**SI-5**, 24 mg, 0.07 mmol, 1.0 eq.) was dissolved in tetrahydrofuran (3 mL) and cooled down to -20 °C (NaCl, ice bath). To this solution triphenylphosphine (28 mg, 0.11 mmol, 1.5 eq.) dissolved in tetrahydrofuran (2 mL) was added. After complete addition the mixture was stirred for 15 min at -20 °C, warmed up to 40 °C and stirred overnight. The solvent was removed under reduced pressure and the crude product, obtained as a colorless solid, was used directly without further purification.

Yield: 17 mg (0.06 mmol, 81%, colorless solid).

TLC (50% ethyl acetate in cyclohexane): R_f = 0.39.

LC-MS (ESI): t_R = 7.14 min (C_{18}); *m/z* = calcd for $C_7H_{13}N_2O_2S^+$ $[M\text{-}Boc+H]^+$ 189.07, found 188.99, calcd for $C_{12}H_{21}N_2O_4S^+$ $[M+H]^+$ 289.12, found 289.08, calcd for $NaC_{12}H_{20}N_2O_4S^+$ $[M+Na]^+$ 311.10, found 311.06.

HRMS (ESI): *m/z* = calcd for $NaC_{12}H_{20}N_2O_4S^+$ $[M+Na]^+$ 311.1036, found 311.1031.

$[\propto]_{\mathbf{D}}^{\mathbf{24}}$ ([c] = 1 in $CHCl_3$): +17.

^{1}H NMR (400 MHz, $CDCl_3$): δ 5.18 (br, 1H), 5.20-5.10 (m, 1H), 4.60 (br, 1H), 3.80 (s, 3H), 3.64-3.51 (m, 2H), 1.45 (s, 9H), 1.43 (br, 3H).

^{13}C NMR (100 MHz, $CDCl_3$): δ 180.3, 171.2, 155.3, 80.1, 79.3, 53.2, 48.4, 35.4, 28.6, 20.5.

Synthesis methyl (*R*)-2-(1-((*tert*-butoxycarbonyl)amino)ethyl)thiazole-4-carboxylate (2)

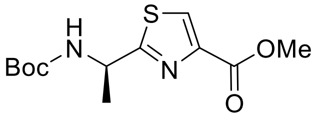

2

Thiazoline (**SI-6**, (24 mg, 0.08 mmol, 1.0 eq.) was dissolved in dichloromethane (3 mL), cooled down to -10 °C (acetone, ice bath), DBU (27 mg, 0.17 mmol, 13 µL, 2.1 eq.)

was added and the resulting mixture was stirred for 10 min at -10 °C. Bromotrichloromethane (17 mg, 0.09 mmol, 9 µL, 1.1 eq.) was added and stirring was continued at -10 °C for 10 minutes, followed by warming up to room temperature and continuous stirring for further 3 h. The solvent was removed under reduced pressure and the crude product was purified by flash column chromatography (33% ethyl acetate in cyclohexane) to yield methyl (*R*)-2-(1-((*tert*-butoxycarbonyl)amino)ethyl)thiazole-4-carboxylate (**2**) as a colorless solid.

Yield: 19 mg (0.07 mmol, 81%, colorless solid).
TLC (33% ethyl acetate in cyclohexane): R_f = 0.27.
LC-MS (ESI): t_R = 7.34 min (C_{18}); *m/z* = calcd for $C_7H_{10}N_2O_2S^+$ [M-Boc+H]$^+$ 186.05 found 186.88, calcd for $C_{12}H_{19}N_2O_4S^+$ [M+H]$^+$ 287.11, found 287.07, calcd for $NaC_{12}H_{18}N_2O_4S^+$ [M+Na]$^+$ 309.09, found 309.05.
HRMS (ESI): *m/z* = calcd for $C_{12}H_{19}N_2O_4S^+$ [M+H]$^+$ 287.1060, found 287.1061, calcd for $NaC_{12}H_{18}N_2O_4S^+$ [M+Na]$^+$ 309.0879, found 309.0896.
$[\alpha]_D^{22}$ ([c] = 1 in $CHCl_3$): +5.
^{1}H NMR (400 MHz, $CDCl_3$): δ 8.10 (s, 1H), 5.18 (br, 1H), 5.11 (br, 1H), 3.94 (s, 3H), 1.62 (d, *J* = 6.8, 3H), 1.44 (s, 9H).
^{13}C NMR (100 MHz, $CDCl_3$): δ 175.3, 161.9, 155.0, 147.0, 127.5, 80.4, 52.6, 49.0, 28.4, 21.8.

Chemical synthesis of Fragment A

The synthesis of fragment A was carried out as described in **Fig. 2a** *via* the following intermediates:

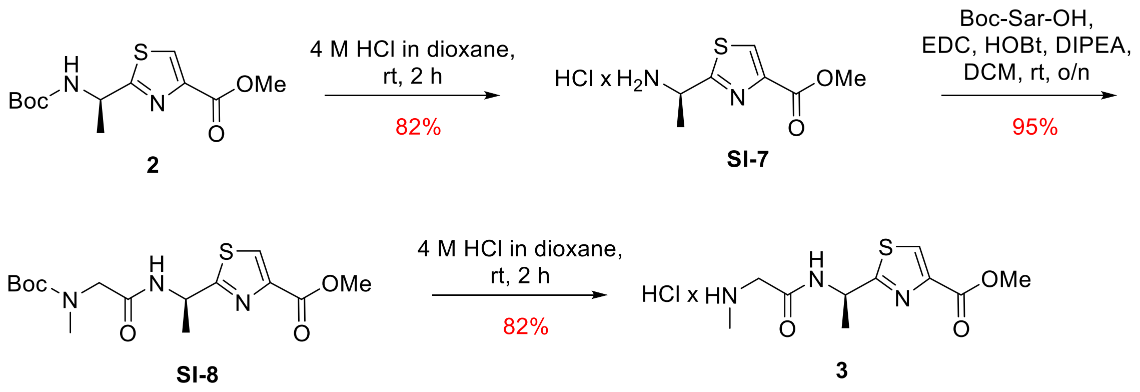

Synthesis methyl (*R*)-2-(1-aminoethyl)thiazole-4-carboxylate hydrochloride salt (SI-7)

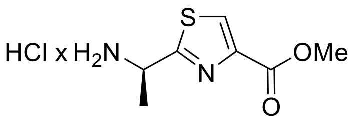

SI-7

Methyl (*R*)-2-(1-aminoethyl)thiazole-4-carboxylate hydrochloride salt (**SI-7**) was synthesized following the *Boc-Deprotection General Procedure* utilizing methyl (*R*)-2-(1-((*tert*-butoxycarbonyl)amino)ethyl)thiazole-4-carboxylate (**2**, 30 mg, 0.1 mmol, 1.0 eq.) and hydrogen chloride solution (1 mL, 4.0 M in 1,4-dioxane) to yield methyl (*R*)-2-(1-aminoethyl)thiazole-4-carboxylate hydrochloride salt (**SI-7**) as a colorless solid.

Yield: 19 mg (0.07 mmol, 82%, colorless solid).

TLC (10% methanol in dichloromethane) R_f = 0.70.

LC-MS (ESI): t_R = 1.64 min (C_{18}); *m/z* = calcd for $C_7H_{11}N_2O_2S^+$ $[M+H]^+$ 187.05, found 186.98, calcd for $NaC_7H_{10}N_2O_2S^+$ $[M+Na]^+$ 209.04, found 209.03.

HRMS (ESI): *m/z* = calcd for $C_7H_{11}N_2O_2S^+$ $[M+H]^+$ 187.0536, found 187.0525, calcd for $NaC_7H_{10}N_2O_2S^+$ $[M+Na]^+$ 209.0355, found 209.0351.

$[\propto]_{\mathbf{D}}^{\mathbf{24}}$ ([c] = 1 in $CHCl_3$): -1.

^{1}H NMR (400 MHz, MeOD): δ 8.50 (s, 1H), 7.96-7.76 (m, 2H), 3.93 (s, 3H), 3.69-3.66 (m, 1H), 1.76 (d, *J* = 6.8, 3H).

^{13}C NMR (100 MHz, MeOD): δ 169.0, 162.9, 147.6, 131.0, 53.0, 49.5 20.3.

Synthesis methyl (*R*)-2-(1-(2-((*tert*-butoxycarbonyl) (methyl)amino) acetamido) ethyl) thiazole-4-carboxylate (SI-8)

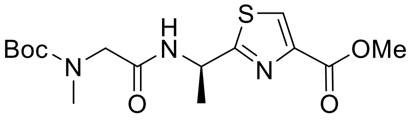

SI-8

Methyl (*R*)-2-(1-(2-((*tert*-butoxycarbonyl)(methyl)amino)acetamido)ethyl)thiazole-4-carboxylate (**SI-8**) was synthesized following the *Peptide-Coupling General Procedure (EDC)* utilizing methyl (*R*)-2-(1-aminoethyl)thiazole-4-carboxylate hydrochloride salt (**SI-7**, 25 mg, 11 mmol, 1.0 eq.), Boc-Sar-OH (25 mg, 13 mmol, 1.2 eq.), EDC (65 mg,

0.34 mmol, 3.0 eq.), HOBt (46 mg, 34 mmol, 3.0 eq.), DIPEA (73 mg, 56 mmol, 98 µL, 5.0 eq.) and dichloromethane (5 mL). The crude product was purified by reversed phase flash column chromatography (40% acetonitrile in water) to afford methyl (*R*)-2-(1-(2-((*tert*-butoxycarbonyl)(methyl)amino) acetamido) ethyl) thiazole-4-carboxylate (**SI-8**) as a colorless solid.

Yield: 38 mg (0.07 mmol, 95%, colorless solid).
TLC (50% ethyl acetate in cyclohexane): R_f = 0.13;
(66% ethyl acetate in cyclohexane): R_f = 0.21.
LC-MS (ESI): t_R = 6.45 min (C_{18}); *m/z* = calcd for $C_{10}H_{16}N_3O_3S^+$ [M-Boc+H]$^+$ 258.08, found 258.01, calcd for $C_{15}H_{24}N_3O_5S^+$ [M+H]$^+$ 358.14, found 358.10, calcd for $NaC_{15}H_{23}N_3O_5S^+$ [M+Na]$^+$ 380.13, found 380.06, calcd for $Na(C_{15}H_{23}N_3O_5S)^{2+}$ [2M+Na]$^+$ 737.26, found 736.94.
HRMS (ESI): *m/z* = calcd for $C_{15}H_{24}N_3O_5S^+$ [M+H]$^+$ 358.1431, found 358.1428, calcd for $NaC_{15}H_{23}N_3O_5S$+ [M+Na]$^+$ 380.1251, found 380.1269.
$[\propto]_D^{24}$ ([c] = 1 in $CHCl_3$): -1.
^{1}H NMR (400 MHz, $CDCl_3$): δ 8.06 (s, 1H), 7.00 (br, 1H), 5.39 (br, 1H), 3.88 (s, 3H), 3.83 (d, *J* = 16.3, 3H), 2.91 (s, 3H), 1.61 (d, *J* = 6.96, 3H), 1.41 (s, 9H).
^{13}C NMR (100 MHz, $CDCl_3$): δ 173.9, 173.7, 169.1, 161.7, 146.8, 127.6, 80.9, 53.1, 52.4, 47.2, 36.0, 28.3, 21.3.

Synthesis methyl (*R*)-2-(1-(2- (methylamino) acetamido)ethyl)thiazole-4-carboxylate hydrochloride salt (3)

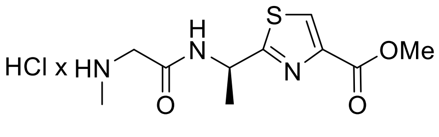

3

Methyl (*R*)-2-(1-(2-(methylamino) acetamido) ethyl)thiazole-4-carboxylate hydrochloride salt (**3**) was synthesized following the *Boc-Deprotection General Procedure* utilizing methyl (*R*)-2-(1-(2-((tert-butoxycarbonyl) (methyl)amino) acetamido)ethyl)thiazole-4-carboxylate (**SI-8**, 48 mg, 0.13 mmol, 1.0 eq.) and hydrogen chloride solution (1 mL, 4 M in 1,4-dioxane). The crude product was purified by phase flash column chromatography (20% methanol in dichloromethane) to afford

methyl (*R*)-2-(1-(2-(methylamino)acetamido)ethyl)thiazole-4-carboxylate hydrochloride salt (**3**) as a colorless solid.

Yield: 32 mg (0.11 mmol, 81%, colorless solid).

TLC (10% methanol in dichloromethane): R_f = 0.15;

(20% methanol in dichloromethane): R_f = 0.33.

LC-MS (ESI): t_R = 1.76 min (C_{18}); *m/z* = calcd for $C_{10}H_{16}N_3O_3S^+$ $[M+H]^+$ 258.09, found 258.04, calcd for $NaC_{10}H_{15}N_3O_3S^+$ $[M+Na]^+$ 280.07, found 280.01, calcd for $H(C_{10}H_{15}N_3O_3S)_2$ $[2M+H]^+$ 515.17, found 515.09, calcd for $Na(C_{10}H_{15}N_3O_3S)_2$ $[2M+Na]^+$ 537.16, found 537.07.

HRMS (ESI): *m/z* = calcd for $C_{10}H_{16}N_3O_3S$ $[M+H]^+$ 258.0907, found 258.0911, calcd for $NaC_{10}H_{15}N_3O_3S^+$ $[M+Na]^+$ 280.0726, found 280.0730.

$[\alpha]_D^{23}$ ([c] = 1 in MeOH): +2.

^{1}H NMR (400 MHz, MeOD): δ 8.33 (s, 1H), 5.44 (q, *J* = 7.0, 1H), 3.92 (s, 3H), 3.56 (s, 2H), 2.55 (s, 3H), 1.63 (d, *J* = 7.0, 3H).

^{13}C NMR (100 MHz, MeOD): δ 176.4, 170.3, 163.2, 147.4, 129.3, 52.8, 52.8, 52.7, 35.0, 21.0.

Chemical synthesis of Fragment B

The synthesis of fragment B was carried out as described in **Fig. 2b** via the following intermediates:

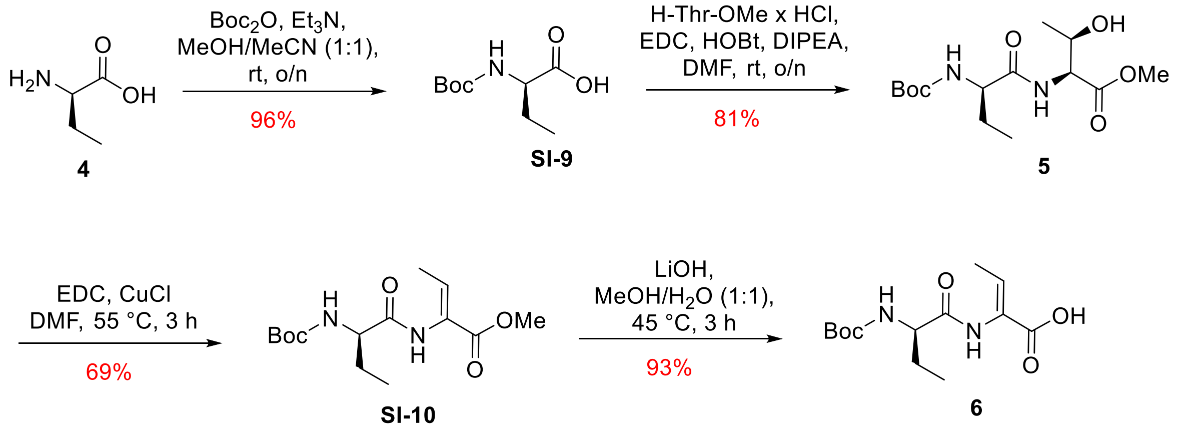

Synthesis of Boc-(D)-Abu-OH (SI-9)

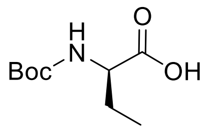

SI-9

Boc-(D)-Abu-OH (**SI-9**) was synthesized by following the *Boc-Protection General Procedure* utilizing H-(D)-Abu-OH (1.00 g, 9.70 mmol, 1.0 eq.), di-*tert*-butyl dicarbonate (2.77 g, 11.64 mmol, 2.64 mL, 1.2 eq.), triethylamine (2.51 g, 19.39 mmol, 3.45 mL, 2.0 eq.), acetonitrile (30 mL) and methanol (30 mL). Boc-(D)-Abu-OH (**SI-9**) was obtained as a colorless oil.

Yield: 1.90 g (9.35 mmol, 96%, colorless oil).
TLC (33% ethyl acetate in cyclohexane): R_f = 0.28.
LC-MS (ESI): t_R = 6.90 (C_{18}); *m/z* = calcd for $C_9H_{16}NO_4$ [M-H]$^-$ 202.11, found 202.14.
HRMS (ESI): *m/z* = calcd for $C_9H_{16}NO_4$ [M-H]$^-$ 202.1074, found 202.1091.
$[\propto]_D^{23}$ ([c] = 2 in $CHCl_3$): -14.
^{1}H NMR (400 MHz, $CDCl_3$): δ 4.26 (m, 1H), 1.97-1.81 (m, 1H), 1.80-1.66 (m, 1H), 1.44 (s, 9H), 0.96 (t, *J* = 7.4, 3H).
^{13}C NMR (100 MHz, $CDCl_3$): δ 175.5, 163.2, 79.8, 53.5, 28.4, 25.8, 9.6.

Synthesis of Boc-(D)-Abu-(L)-Thr-OMe (5)

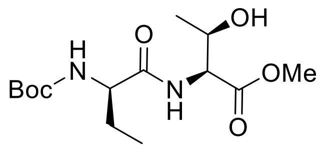

5

Boc-(D)-Abu-(L)-Thr-OMe (**5**) was synthesized following the *Peptide-Coupling General Procedure (EDC)* utilizing Boc-(D)-Abu-OH (**SI-9**, 1.00 g, 4.92 mmol, 1.0 eq.), H-(L)-Thr-OMe hydrochloride salt (998 mg, 5.90 mmol, 1.2 eq.), EDC (2.83 g, 14.76 mmol, 3.0 eq.), HOBt (2.00 g, 14.76 mmol, 3.0 eq.), DIPEA (3.18 g, 24.60 mmol, 4.29 mL, 5.0 eq.) and dichloromethane (30 mL). The crude product was purified by flash column chromatography (50% ethyl acetate in cyclohexane) to afford Boc-(D)-Abu-(L)-Thr-OMe (**5**) as a colorless solid.

Yield: 954 mg (3.00 mmol, 60%, colorless solid).

TLC (33% ethyl acetate in cyclohexane): R_f = 0.09,
(66% ethyl acetate in cyclohexane): R_f = 0.42.

LC-MS (ESI): t_R = 5.99 (C_{18}); *m/z* = calcd for $C_9H_{18}N_2O_4$ [M-Boc+H]$^+$ 219.34, found 219.06, calcd for $H(C_{14}H_{26}N_2O_6)_2$ [2M+H]$^+$ 637.37, found 637.07, calcd for $NaC_{14}H_{27}N_2O_6$ [M+Na]$^+$ 341.17, found 341.13, calcd for $Na(C_{14}H_{26}N_2O_6)_2$ [2M+Na]$^+$ 659.35, found 659.27.

HRMS (ESI): *m/z* = calcd for $C_{14}H_{27}N_2O_6$ [M+H]$^+$ 319.1864, found 319.1870, calcd for $NaC_{14}H_{27}N_2O_6$ [M+Na]$^+$ 341.1683, found 341.1700, calcd for $Na(C_{14}H_{26}N_2O_6)_2$ [2M+Na]$^+$ 659.3474, found 658.3480.

$[\propto]_D^{24}$ ([c] = 1 in $CHCl_3$): +16.

^{1}H NMR (400 MHz, $CDCl_3$): δ 6.98 (d, *J* = 8.9, 1H), 5.16 (br, 1H), 4.57 (dd, *J* = 2.6, 8.9, 1H), 4.37-4.32 (m, 1H), 4.12 (br, 1H), 3.75 (s, 3H), 2.54 (br, 1H), 1.95-1.84 (m, 1H), 1.75-1.60 (m, 1H), 1.44 (s, 9H), 1.21 (d, *J* = 6.4, 3H), 0.97 (t, *J* = 7.43, 3H).

^{13}C NMR (100 MHz, $CDCl_3$): δ 172.9, 171.7, 156.0, 80.4, 68.1, 57.4, 56.1, 52.8, 28.5, 25.9, 20.2, 10.1.

Synthesis of Boc-(D)-Abu-(*Z*)-Dhb-OMe (SI-10)

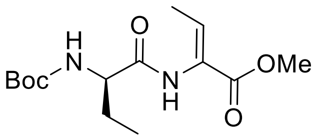

SI-10

Boc-(D)-Abu-(Z)-Dhb-Ome (**SI-10**) was synthesized by following the *Dehydrobutyrine Synthesis General Procedure* utilizing Boc-(D)-Abu-(L)-Thr-Ome (**5**, 287 mg, 0.90 mmol, 1.0 eq.), EDC (864 mg, 4.51 mmol, 5.0 eq.), copper(I) chloride (241 mg, 2.43 mmol, 2.7 eq.) and dimethylformamide (10 mL). The obtained crude product was purified by flash column chromatography (20% ethyl acetate in cyclohexane) to afford Boc-(D)-Abu-(*Z*)-Dhb-Ome (**SI-10**) as a colorless oil.

Yield: 188 mg (0.63 mmol, 69%, colorless oil).

TLC (50% ethyl acetate in cyclohexane): R_f = 0.51.

LC-MS (ESI): t_R = 6.82 (C_{18}); *m/z* = calcd for $C_9H_{16}N_2O_3$ [M-Boc+H]$^+$ 200.12, found 200.97, calcd for $C_{14}H_{25}N_2O_5$ [M+H]$^+$ 301.18, found 301.03, calcd for $H(C_{14}H_{24}N_2O_5)_2$ [2M+H]$^+$ 601.34, found 601.19, calcd for $NaC_{14}H_{24}N_2O_5$

[M+Na]+ 323.16, found 323.12, calcd for $Na(C_{14}H_{24}N_2O_5)_2$ [2M+Na]+ 623.33, found 623.20.

HRMS (ESI): *m/z* = calcd for $C_{14}H_{25}N_2O_5$ [M+H]+ 301.1758, found 301.1763, calcd for $NaC_{14}H_{24}N_2O_5$ [M+Na]+ 323.1577, found 323.1596, $Na(C_{14}H_{24}N_2O_5)_2$ [2M+Na]+ 623.3263, found 623.3263.

$[\alpha]_D^{21}$ ([c] = 1 in $CHCl_3$): +37.

^{1}H NMR (400 MHz, $CDCl_3$): δ 7.62 (br, 1H), 6.79 (q, *J* = 7.2, 1H), 5.17 (d, *J* = 7.3, 1H), 4.17 (br, 1H), 3.73 (s, 3H), 1.96-1.86 (m, 1H), 1.73 (d, *J* = 7.2, 3H), 1.71-1.66 (m, 1H), 1.42 (s, 9H), 0.98 (t, *J* = 7.4, 3H).

^{13}C NMR (100 MHz, $CDCl_3$): δ 170.7, 165.0, 156.0, 134.7, 126.1, 80.3, 56.1, 52.4, 28.5, 25.8, 14.7, 10.0.

Synthesis of Boc-(D)-Abu-(*Z*)-Dhb-OH (6)

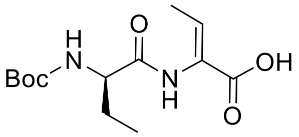

6

Boc-(D)-Abu-(*Z*)-Dhb-OH (**6**) was synthesized following the *Ester Hydrolysis General Procedure (LiOH)* utilizing Boc-(D)-Abu-(*Z*)-Dhb-OMe (**SI-10**, 128 mg, 0.43 mmol, 1.0 eq.), lithium hydroxide (20 mg, 0.85 mmol, 2.0 eq.), methanol (3.0 mL) and water (3.0 mL). Boc-(D)-Abu-(*Z*)-Dhb-OH (**6**) was obtained as a colorless oil.

Yield: 113 mg (0.40 mmol, 93%, colorless oil).

TLC (10% methanol in dichloromethane): R_f = 0.48.

LC-MS (ESI): t_R = 5.83 (C_{18}); *m/z* = calcd for $C_8H_{14}N_2O_3$ [M-Boc+H]+ 186.10, found 186.97, calcd for $C_{13}H_{23}N_2O_5$ [M+H]+ 287.16, found 287.18, calcd for $NaC_{13}H_{22}N_2O_5$ [M+Na]+ 309.14, found 309.06, calcd for $H(C_{13}H_{22}N_2O_5)_2$ [2M+H]+ 573.31, found 573.27, calcd for $Na(C_{13}H_{22}N_2O_5)_2$ [2M+Na]+ 595.30, found 595.26.

HRMS (ESI): *m/z* = calcd for $C_{13}H_{23}N_2O_5$ [M+H]+ 287.1601, found 287.1602, calcd for $NaC_{13}H_{22}N_2O_5$ [M+Na]+ 309.1421, found 309.1428, calcd for $H(C_{13}H_{22}N_2O_5)_2$ [2M+H]+ 573.3130, found 573.3177, calcd for $Na(C_{13}H_{22}N_2O_5)_2$ [2M+Na]+ 595.3950, found 595.2946.

$[\alpha]_D^{23}$ ([c] = 1 in MeOH): +19.

^{1}H NMR (400 MHz, $CDCl_3$): δ 6.84 (d, *J* = 7.1, 1H), 4.07-4.03 (m, 1H), 1.91-1.81 (m, 1H), 1.75 (d, *J* = 7.1, 3H), 1.72-1.64 (m, 1H), 1.45 (s, 9H), 1.02 (t, *J* = 7.5, 3H).

^{13}C NMR (100 MHz, MeOD): δ 174.4, 167.2, 157.9, 136.5, 128.6, 80.6, 57.7, 28.7, 26.6, 14.1, 10.6.

Chemical synthesis of (2*S*,3*S*) 2-methylthreonine methyl ester hydrochloride (7)

(2*S*,3*S*) 2-methylthreonine methyl ester hydrochloride was synthesized according to **Supporting Fig. 9**.

Synthesis of (2*R*,3*S*)-methyl-2,3-dihydroxy-2-methylbutanoate (SI-12)

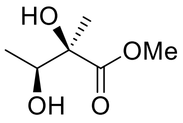

SI-12

To a cooled solution (0 °C, ice bath) of AD-mix α (23.97 g, 17.13 mmol, 1.0 eq.) and methanesulfonamide (326 mg, 3.43 mmol, 0.2 eq.) in *tert*-butyl alcohol (90 mL) and water (90 mL) was added methyl tiglate (**SI-11**, 1.96 g, 17,13 mmol, 1.0 eq.). The resulting reaction mixture was slowly warmed up to 10 °C (cold room) and stirred for two days. Solid sodium sulfite (27 g) was added and the mixture was stirred for 20 min at room temperature. Diethyl ether (350 mL) was added, the phases were separated, and the aqueous layer was extracted with diethyl ether (1x, 50 mL). The combined organic phases were washed with water (2x, 40 mL) and brine (1x, 40 mL). The organic layer was dried over $MgSO_4$ and concentrated under reduced pressure. The crude product was purified by flash column chromatography (33% ethyl acetate in cyclohexane) to afford pure (2*R*,3*S*)-methyl-2,3-dihydroxy-2-methylbutanoate (**SI-12**) as a colorless oil.

Yield: 1.79 g (12.1 mmol, 71%, colorless oil).

TLC (33% ethyl acetate in cyclohexane): R_f = 0.47.

LC-MS (ESI): t_R = 2.88 min (C_{18}); *m/z* = calcd for $C_6H_{13}O_4^+$ $[M+H]^+$ 149.08, found 148.86, calcd for $H(C_6H_{12}O_4)_2^+$ $[2M+H]^+$ 297.15, found 296.74, calcd for $NaC_6H_{12}O_4^+$ $[M+Na]^+$ 171.06, found 171.03.

HRMS (ESI): *m/z* = calcd for $NaC_6H_{12}O_4^+$ $[M+Na]^+$ 171.0633, found 171.0663.

$[\alpha]_D^{25}$ ([c] = 1 in $CHCl_3$): -1.

^{1}H NMR (400 MHz, $CDCl_3$): δ 3.89 (q, *J* = 6.5, 1H), 3.74 (s, 3H), 1.25 (s, 3H), 1.16 (d, *J* = 6.5, 3H).

^{13}C NMR (100 MHz, $CDCl_3$): δ 176.8, 77.5, 71.7, 52.9, 21.6, 16.5.

Synthesis of (4*R*,5*S*)-methyl-4,5-dimethyl-1,3,2-dioxathiolane-4-carboxylate-2,2-dioxide (SI-13)

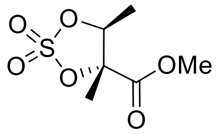

SI-13

To a cooled solution (0 °C, ice bath) of (2*R*,3*S*)-methyl-2,3-dihydroxy-2-methylbutanoate (**SI-12**, 1.1 g, 7.32 mmol, 1.0 eq.) in dichloromethane (10 mL), thionyl chloride (1.74 g, 1.06 mL, 18.29 mmol, 2.5 eq.) was added and the reaction mixture was heated to reflux for 3 h. The mixture was concentrated to dryness under reduced pressure. To remove the remaining thionyl and hydrogen chloride, the crude mixture was dried under high vacuum overnight. (4*R*, 5*S*)-methyl-4,5-dimethyl-1,3,2-dioxathiolane-4-carboxylate-2-oxide was obtained in a quantitative yield and was used without further purification.

TLC (33% ethyl acetate in cyclohexane): R_f = 0.5.

LC-MS (ESI): t_R = 6.70 (C_{18}); *m/z* = calcd for $C_6H_{11}O_5S^+$ $[M+H]^+$ 195.03, found 195.08.

The cyclic sulfite was dissolved in a (1.5:1:1) mixture of water (15 mL), acetonitrile (10 mL) and carbon tetrachloride (10 mL). Sodium periodate (3.13 g, 14.63 mmol, 2.0 eq.) and ruthenium(III) chloride hydrate (25 mg, 0.12 mmol, 0.02 eq.) were added, the resulting reaction mixture was warmed up to 40 °C and stirred for 4 h. The mixture was cooled to room temperature and extracted with diethyl ether (3x). A small amount of activated carbon was added to the combined organic phases to remove the occurring brown color. The organic layer was dried over $MgSO_4$ and concentrated under reduced pressure. The crude product was purified by flash column

chromatography (33% ethyl acetate in cyclohexane) to afford pure (4*R*,5*S*)-methyl-4,5-dimethyl-1,3,2-dioxathiolane-4-carboxylate-2,2-dioxide (**SI-13**) as a colorless oil. The oil turned into a colorless solid after storing at -80 °C overnight.

Yield: 1.3 g (6.19 mmol, 85%, colorless solid).
TLC (33% ethyl acetate in cyclohexane): R_f = 0.34.
LC-MS (ESI): t_R = 7.05 (C18); *m/z* = calcd for $C_6H_9O_6S^-$ [M-H]$^-$ 209.01, found 209.01.
HRMS (ESI): *m/z* = calcd for $C_6H_9O_6S^-$ [M-H]$^-$ 209.0119, found 209.0147.
$[\alpha]_D^{25}$ ([c] = 1 in $CHCl_3$)**:** -3.1.
^{1}H NMR (400 MHz, $CDCl_3$): δ 5.22 (q, *J* = 6.5, 1H), 3.87 (s, 3H), 1.70 (s, 3H), 1.60 (d, *J* = 6.49, 3H).
^{13}C NMR (100 MHz, $CDCl_3$): δ 168.1, 89.4, 82.8, 53.9, 18.2, 14.6.

Synthesis of (2*S*,3*S*)-methyl-2-azido-2-methyl-3-(sulfooxy)butanoate (SI-14)

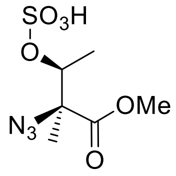

SI-14

To a solution of (4*R*,5*S*)-methyl-4,5-dimethyl-1,3,2-dioxathiolane-4-carboxylate-2,2-dioxide (**SI-13**, 518 mg, 2.47 mmol, 1.0 eq.) in a (6:1) mixture of acetone (6 mL) and water (1 mL) was added sodium azide (401 mg, 6.17 mmol, 2.5 eq.). The reaction mixture was heated up to 50 °C for 4 h, cooled again to room temperature and the solvent was removed under reduced pressure. The residue was redissolved in a small amount of water and lyophilized to obtain (2*S*,3*S*)-methyl-2-azido-2-methyl-3-(sulfooxy)butanoate (**SI-14**) as a colorless powder.

Yield: 609 mg (2.4 mmol, 97%, colorless solid).
TLC (66% ethyl acetate in cyclohexane): R_f = 0.39.
LC-MS (ESI): t_R = 4.13; *m/z* = calcd for $C_6H_{10}N_3O_6S^-$ [M-H]$^-$ 252.03, found 251.99.
HRMS (ESI): *m/z* = calcd for $C_6H_{10}N_3O_6S^-$ [M-H]$^-$ 252.0285, found 252.0289.
$[\alpha]_D^{27}$ ([c] = 1 in MeOH): -1.0.
^{1}H NMR (400 MHz, MeOD): δ 4.70 (q, *J* = 6.3, 1H), 3.79 (s, 3H), 1.60 (s, 3H), 1,31 (d, *J* = 6.4, 3H).
^{13}C NMR (100 MHz, MeOD): δ 172.9, 78.1, 70.6, 53.5, 19.7, 16.3.

Synthesis of (2*S*,3*S*)-methyl 2-amino-2-methyl-3-(sulfooxy)butanoate (SI-15)

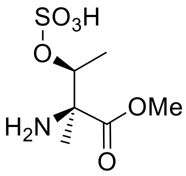

SI-15

To a solution of (2*S*,3*S*)-methyl-2-azido-2-methyl-3-(sulfooxy)butanoate (**SI-14**, 1.95 g, 7.72 mmol, 1.0 eq.) in dry methanol (20 mL) under a hydrogen atmosphere was added a suspension of a small amount of Pd/C in dry methanol (3 mL). The reaction mixture was stirred overnight at room temperature. The Pd/C was removed by filtering over Celite® 545. And the organic solvent was removed under reduced pressure. (2*S*,3*S*)-methyl 2-amino-2-methyl-3-(sulfooxy)butanoate (**SI-15**) was obtained as a pale yellow oil.

Yield: 1.74 g (7.64 mmol, 99%, pale yellow oil).
TLC (10% methanol in dichloromethane): R_f = 0.33.
LC-MS (ESI): t_R = 1.60 (C_{18}); *m/z* = calcd for $C_6H_{12}NO_6S^-$ $[M-H]^-$ 226.04, found 226.03.
HRMS (ESI): *m/z* = calcd for $C_6H_{14}NO_6S^+$ $[M+H]^+$ 228.0536, found 228.0523, calcd for $NaC_6H_{13}NO_6S^+$ $[M+Na]^+$ 250.0356, found 250.0347.
$[\propto]_D^{26}$ ([c] = 1 in MeOH): +2.1.
^{1}H NMR (400 MHz, MeOD): δ 4.66 (q, *J* = 6.6, 1H), 3.88 (s, 3H), 1.62 (s, 3H), 1,42 (d, *J* = 6.6, 3H).
^{13}C NMR (100 MHz, MeOD): δ 170.5, 77.0, 64.7, 54.1, 20.4, 16.2.

Synthesis of (2*S*,3*S*) 2-methylthreonine methyl ester hydrochloride (7)

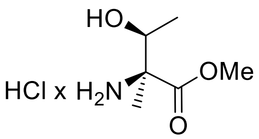

7

(2*S*,3*S*)-methyl 2-amino-2-methyl-3-(sulfooxy)butanoate (**SI-15**, 550 mg, 2.17 mmol, 1.0 eq.) was dissolved in a (1:1) mixture of methanol (3 mL) and concentrated sulfuric acid (3 mL). The mixture was stirred vigorously at room temperature for two days. The reaction mixture was diluted with methanol (5 mL) and adjusted to pH 8-9 by addition of solid sodium hydrogencarbonate. Water (15 mL) was added and methanol was

removed under reduced pressure. The remaining aqueous phase was extracted with ethyl acetate (3x 15 mL). The combined organic layers were dried over $MgSO_4$. 1 N hydrochloric acid (15 mL) was added and the mixture was shaken vigorously. The organic phase was removed under reduced pressure and the remaining aqueous phase was lyophilized to yield (2*S*,3*S*) 2-methylthreonine methyl ester hydrochloride (**7**) as a colorless powder.

Yield: 356 mg (1.94 mmol, 89%, colorless solid).
TLC (10% methanol in dichloromethane): R_f = 0.62.
LC-MS (ESI): t_R = 1.46; *m/z* = calcd for $C_6H_{14}NO_3^+$ $[M+H]^+$ 148.10, found 148.04, calcd for $NaC_6H_{13}NO_3^+$ $[M+Na]^+$ 170.08, found 170.04.
HRMS (ESI): *m/z* = calcd for $C_6H_{14}NO_3^+$ $[M+H]^+$ 148.0968, found 148.0942, calcd for $NaC_6H_{13}NO_3^+$ $[M+Na]^+$ 170.0788, found 170.0773.
$[\alpha]_D^{25}$ ([c] = 1 in $CHCl_3$): +7.
^{1}H NMR (400 MHz, $CDCl_3$): δ 3.98 (q, *J* = 6.5, 1H), 3.85 (s, 3H), 1.56 (s, 3H), 1.23 (d, *J* = 6.5, 3H).
^{13}C NMR (100 MHz, MeOD): δ 171.4, 70.6, 65.3, 53.8, 20.2, 18.2.

Chemical synthesis of Boc-(L)-(4-OMe)Trp-OH (8)

Boc-(L)-(4-MeO)Trp-OH was synthesized according to **Supporting Fig. 10**.

Boc-*m*-anisidine (SI-17)

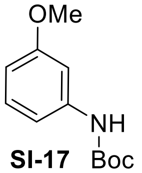

SI-17

Boc-*m*-anisidine (**SI-17**) was synthesized following the *Boc-Protection General Procedure* utilizing *m*-anisidine (**SI-16**, 10 g, 81.2 mmol, 9.17 mL, 1.0 eq.), di-*tert*-butyl dicarbonate (23.2 g, 97.44 mmol, 24.42 mL, 1.2 eq.) triethylamine (20.99 g, 162.4 mmol, 28.91 mL, 2 eq.), acetonitrile (95 mL) and methanol (95 mL). The crude

product was purified by flash column chromatography (2% ethyl acetate in cyclohexane). Boc-*m*-anisidine (**SI-17**) was obtained as a colorless oil.

Yield: 11.36 g (50.88 mmol, 64%, colorless oil).

TLC (5% ethyl acetate in cyclohexane): R_f = 0.41;

(33% ethyl acetate in cyclohexane): R_f = 0.72.

LC-MS (ESI): t_R = 9.81min (C_{18}); *m/z* = calcd for $Na_2C_7H_8NO^+$ $[2Na+M]^+$ 168.04, found 168.57.

HRMS (ESI): *m/z* = calcd for $NaC_{12}H_{17}NO_3^+$ $[Na+M]^+$ 246.1101, found 246.1098.

^{1}H NMR (400 MHz, $CDCl_3$): δ 7.17 (t, *J* = 8.2, 1H), 7.10 (br, 1H), 6.84 (dd, *J* = 1.3, 8.0, 1H), 6.59 (dd, *J* = 2.5, 8.3, 1H), 6.46 (br, 1H), 3.80 (s, 3H), 1.52 (s, 9H).

^{13}C NMR (100 MHz, $CDCl_3$): δ 160.5, 152.8, 139.9, 129.9, 110.8, 109.1, 104.3, 80.7, 55.4, 28.5.

***tert*-Butyl (2-iodo-3-methoxyphenyl)carbamate (SI-18)**

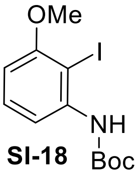

SI-18

Boc-*m*-anisidine (**SI-17**, 3.00 g, 13.44 mmol, 1.0 eq.) was dissolved under an argon atmosphere in anhydrous diethyl ether (80 mL) and cooled down to -20 °C (NaCl, ice bath). *tert*-Butyllithium (1.81 g, 28.22 mmol, 2.1 eq., 1.7 M in pentane, 16.60 mL) was added and the resulting mixture was stirred for 3 h at -20 °C. The mixture was cooled down to -100 °C (pentane, dry ice, liquid nitrogen) and iodine (4.10 g, 16.12 mmol, 1.2 eq.) dissolved in dry diethyl ether (35 mL) was slowly added. After complete addition, the mixture was stirred and gradually warmed up to room temperature overnight. Sodium thiosulfate (sat., aq., 100 mL) was added and stirring was continued for 15 min. The phases were separated and the aqueous phase was extracted with diethyl ether (50 mL, 3x). The combined organic phases were washed with brine, dried over $MgSO_4$ and concentrated under reduced pressure. The obtained crude product was purified by flash column chromatography (15% ethyl acetate in cyclohexane) to afford *tert*-butyl (2-iodo-3-methoxyphenyl)carbamate (**SI-18**) as a colorless solid.

Yield: 2.95 g, (8.46 mmol, 63%, colorless solid).

TLC (33% ethyl acetate in cyclohexane): R_f = 0.65.

LC-MS (ESI): t_R = 10.37 min (C_{18}); *m/z* = $C_7H_9INO^+$ $[M\text{-}Boc+H]^+$ 249.97, found 250.32, calcd for $NaC_{12}H_{16}INO_3^+$ $[M+Na]^+$ 372.01, found 371.96, calcd for $Na(C_{12}H_{16}INO_3)_2^+$ $[2M+Na]^+$ 721.02, found 720.93.

HRMS (ESI): *m/z* = calcd for $NaC_{12}H_{16}INO_3^+$ $[M+Na]^+$ 372.0067, found 372.0093, calcd for $Na(C_{12}H_{16}INO_3)_2^+$ $[2M+Na]^+$ 721.0242, found 721.0253.

^{1}H NMR (400 MHz, $CDCl_3$): δ 7.74 (d, *J* = 8.3, 1H), 7.26 (t, *J* = 5.8, 1H), 7.04 (br, 1H), 6.53 (d, *J* = 8.2, 1H), 3.88 (s, 3H), 1.54 (s, 9H).

^{13}C NMR (100 MHz, $CDCl_3$): δ 158.4, 152.8, 140.4, 129.8, 112.7, 105.6, 81.2, 81.1, 56.7, 28.4.

2-Iodo-3-methoxyphenylamine hydrochloride salt (SI-19)

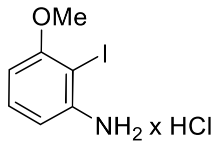

SI-19

2-Iodo-3-methoxyphenylamine hydrochloride salt (**SI-19**) was synthesized following the *Boc-Deprotection General Procedure* utilizing *tert*-butyl (2-iodo-3-methoxyphenyl)carbamate (**SI-18**, 325 mg, 0.93 mmol, 1.0 eq.) and hydrogen chloride solution (3 mL, 4 M in 1,4-dioxane) to afford 2-Iodo-6-methoxyphenylamine hydrochloride salt (**SI-19**) as a colorless solid.

Yield: 251 mg (0.88 mmol, 94%, colorless solid).

TLC (50% ethyl acetate in cyclohexane): R_f = 0.83.

LC-MS (ESI): t_R = 7.73 min (C_{18}); *m/z* = calcd for $C_7H_9INO^+$ $[M+H]^+$ 249.97, found 249.91.

HRMS (ESI): *m/z* = calcd for $C_7H_9INO^+$ $[M+H]^+$ 249.9723, found 249.9735.

^{1}H NMR (400 MHz, MeOD): δ 7.49 (t, *J* = 8.1, 1H), 7.09 (dd, *J* = 8.0, 1.0, 1H), 7.05 (dd, *J* = 7.6, 1, 1H), 3.94 (s, 3H).

^{13}C NMR (100 MHz, MeOD): δ 161.4, 136.2, 132.0, 116.9, 112.1, 84.5, 57.5.

Boc-(L)-Glu(Me)-OMe (SI-21)

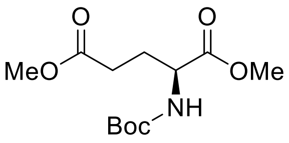

SI-21

H-(L)-Glu-OH (**SI-20**, 10.00 g, 68.00 mmol, 1.0 eq.) was suspended under an argon atmosphere in anhydrous methanol (100 mL) and cooled down to 0 °C (ice bath). To this suspension TMSCl (32.51 g, 299.21 mmol, 37.98 mL, 4.4 eq.) was added slowly. The mixture was warmed up to room temperature slowly and stirred overnight. Dry DIPEA (35.16 g, 272.01 mmol, 47.38, 4.0 eq.) and Boc_2O (17.81 g, 81.60 mmol, 18.75 mL 1.2 eq.) were added slowly and stirring was continued for 6 h. The organic solvent was removed under reduced pressure and the residue was taken up in potassium sulfate (sat., aq., 50 mL) and extracted with diethyl ether (3x 35 mL). The combined organic layers were dried over $MgSO_4$, concentrated under reduced pressure and the obtained crude product was purified flash column chromatography (15% ethyl acetate in cyclohexane) to yield Boc-(L)-Glu(Me)-OMe (**SI-21**) as a colorless oil.

Yield: 18.27 (66.42 mmol, 98%, colorless oil).

TLC (33% ethyl acetate in cyclohexane): R_f = 0.64.

LC-MS (ESI): t_R = 7.30 min (C_{18}); *m/z* = calcd for $C_{19}H_{34}N_2O_{10}^+$ [2M-Boc+H]$^+$ 450.22, found 450.89, $NaC_{12}H_{21}NO_6^+$ [M+Na]$^+$ 298.13, found 298.12, $Na(C_{12}H_{21}NO_6)_2^+$ [2M+Na]$^+$ 573.26, found 573.17.

HRMS (ESI): *m/z* = calcd for $NaC_{12}H_{21}NO_6^+$ [M+Na]$^+$ 298.1261, found 298.1270, $Na(C_{12}H_{21}NO_6)_2^+$ [2M+Na]$^+$ 573.2635, found 573.2636.

$[\propto]_D^{23}$ ([c] = 1 in $CHCl_3$): +10.

^{1}H NMR (400 MHz, $CDCl_3$): δ 5.15 (br, 1H), 4.29 (m, 1H), 3.70 (s, 3H), 3.64 (s, 3H), 2.44-2.30 (m, 2H), 2.19-2.10 (m, 1H), 1.96-1.86 (m, 1H), 1.39 (s, 9H).

^{13}C NMR (100 MHz, $CDCl_3$): δ 173.2, 172.7, 155.4, 80.0, 52.9, 52.4, 51.8, 30.1, 28.3, 27.8.

(Boc)$_2$-(L)-Glu(Me)-OMe (SI-22)

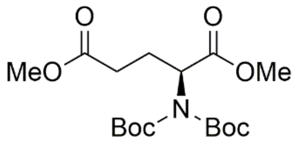

SI-22

Boc-(L)-Glu(Me)-OMe (**SI-21**, 18.00 g, 65.38 mmol, 1.0 eq.) and DMAP (2.00 g, 16.35 mmol, 0.25 eq.) were dissolved in acetonitrile (215 mL). To this solution, di-*tert*-butyl dicarbonate (17.11 g, 78.46 mmol, 18.92 mL, 1.2 eq.) was slowly added. The resulting mixture was stirred overnight at room temperature. The organic solvent was removed under reduced pressure and the obtained crude product was purified by flash column chromatography (15% ethyl acetate in cyclohexane) to obtain (Boc)$_2$-(L)-Glu(Me)-OMe (**SI-22**) as colorless solid.

Yield: 19.60 (52.20 mmol, 80%, colorless solid).
TLC (33% ethyl acetate in cyclohexane): R_f = 0.53.
LC-MS (ESI): t_R = 9.39 min (C_{18}); *m/z* = calcd for $NaC_{17}H_{29}NO_8^+$ [M+Na]$^+$ 398.12, found 398.12, calcd for $Na(C_{17}H_{29}NO_8)_2^+$ [2M+Na]$^+$ 773.37, found 772.83.
HRMS (ESI): *m/z* = calcd for $C_{17}H_{30}NO_8^+$ [M+H]$^+$ 376.1966, found 376.1966, calcd for $NaC_{17}H_{29}NO_8^+$ [M+Na]$^+$ 398.1785, found 398.1788, calcd for $Na(C_{17}H_{29}NO_8)_2^+$ [2M+Na]$^+$ 773.3684, found 773.3699.
$[\alpha]_D^{23}$ ([c] = 1 in $CHCl_3$): -34.
^{1}H NMR (400 MHz, $CDCl_3$): δ 4.93-4.90 (m, 1H), 3.70 (s, 3H), 3.65 (s, 3H), 2.51-2.45 (m, 1H), 2.42-2.32 (m, 2H), 2.21-2.12 (m, 1H), 1.47 (s, 18H).
^{13}C NMR (100 MHz, $CDCl_3$): δ 173.2, 170.9, 152.0, 83.4, 57.4, 52.3, 51.8, 30.7, 28.1, 25.3.

Methyl (*S*)-2-di-*tert*-butoxycarbonylamino-5-oxopentanoate (SI-23)

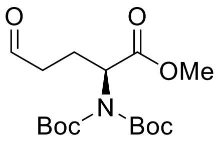

SI-23

$(Boc)_2$-(L)-Glu(Me)-OMe (**SI-22**, 1.00 g, 2.66 mmol, 1.0 eq.) was dissolved in dry diethyl ether (27 mL) under argon atmosphere. The solution was cooled down to -78 °C (dry ice, acetone bath) and DIBAL-H (2.66 mmol, 1 eq., 2.66 mL, 1 M in toluene) was slowly added. The mixture was stirred 15 min at this temperature, the reaction was stopped by the addition of water (400 µL) and stirring was continued for further 30 min. $MgSO_4$ was added to remove the water and the mixture was filtered. The remaining organic layer was concentrated under reduced pressure and the obtained cure product was purified by flash column chromatography (15% ethyl acetate in cyclohexane) to yield methyl (*S*)-2-di-*tert*-butoxycarbonylamino-5-oxopentanoate (**SI-23**) as colorless oil.

Yield: 772 mg (2.24 mmol, 84%, colorless oil).

TLC (33% ethyl acetate in cyclohexane): R_f = 0.61.

LC-MS (ESI): t_R = 8.96 min (C_{18}); *m/z* = calcd for $NaC_{11}H_{19}NO_5^+$ $[M\text{-}Boc+Na]^+$ 268.12, found 268.88, calcd for $NaC_{16}H_{27}NO_7^+$ $[M+Na]^+$ 368.17, found 386.06.

HRMS (ESI): *m/z* = calcd for $C_{16}H_{28}NO_7^+$ $[M+H]^+$ 346.1860, found 346.1836, $NaC_{16}H_{27}NO_7^+$ $[M+Na]^+$ 368.1680, found 368.1655.

$[\propto]_D^{23}$ ([c] = 1 in $CHCl_3$): -28.

^{1}H NMR (400 MHz, $CDCl_3$): δ 9.76 (t, *J* = 1.0, 1H), 4.89-4.85 (m, 1H), 3.71 (s, 3H), 2.63-2.44 (m, 3H), 2.20-2.10 (m, 1H), 1.48 (S, 18H).

^{13}C NMR (100 MHz, $CDCl_3$): δ 201.0, 170.8, 152.1, 83.6, 57.4, 52.4, 40.6, 28.1, 22.7.

(Boc)$_2$-(L)-(4-MeO)Trp-OMe (SI-24)

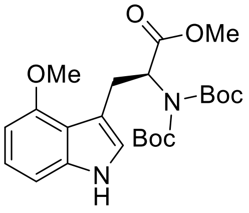

SI-24

Methyl (*S*)-2-di-*tert*-butoxycarbonylamino-5-oxopentanoate (**SI-23**, 242 mg, 0.7 mmol, 1.0 eq.), 2-iodo-3-methoxyphenylamine hydrochloride salt (**SI-19**, 200 mg, 0.7 mmol, 1.0 eq.) and DABCO (236 mg, 2.1 mmol, 3.0 eq.) were dissolved under an argon atmosphere in dry DMF (25 mL). The mixture was degassed in a sonication bath for 20 min. During that time argon was bubbled through the solution. Palladium(II) acetate (16 mg, 0.07 mmol, 0.1 eq.) dissolved in dry, degassed DMF (5 mL) was added and the reaction mixture was heated to 85 °C and stirred overnight. The mixture was cooled down to room temperature, diluted with water (30 mL) and extracted with ethyl acetate (3x 50 mL). The combined organic phases were washed with brine, dried over $MgSO_4$ and concentrated under reduced pressure. The obtained crude product was purified by flash column chromatography (15% ethyl acetate in cyclohexane) to yield (Boc)$_2$-(L)-(4-MeO)Trp-OMe (**SI-24**) as a yellow solid.

Yield: 172 mg (0.38 mmol, 55%, yellow solid).

TLC (15% ethyl acetate in cyclohexane): R_f = 0.25;
(33% ethyl acetate in cyclohexane): R_f = 0.38.

LC-MS (ESI): t_R = 9.82 min (C_{18}); *m/z* = calcd for $C_{18}H_{25}N_2O_5^+$ $[M\text{-}Boc+H]^+$ 349.18, found 348.79, calcd for $NaC_{23}H_{32}N_2O_7^+$ $[M+Na]^+$ 471.21, found 471.18, calcd for $Na(C_{23}H_{32}N_2O_7)_2^+$ $[2M+Na]^+$ 919.43, found 918.83.

HRMS (ESI): *m/z* = calcd for $NaC_{23}H_{32}N_2O_7^+$ $[M+Na]^+$ 471.2107, found 471.2102, calcd for $Na(C_{23}H_{32}N_2O_7)_2^+$ $[2M+Na]^+$ 919.4317, found 919.4300.

$[\propto]_D^{23}$ ([c] = 0.5 in $CHCl_3$): -79.

^{1}H NMR (400 MHz, $CDCl_3$): δ 8.16 (br, 1H), 7.03 (t, *J* = 8.0, 1H), 6.93 (d, *J* = 8.0, 1H), 6.79 (d, *J* = 1.9, 1H), 6.45 (d, *J* = 7.7, 1H), 5.39 (dd, *J* = 4.0, 10.8, 1H), 3.89 (s, 3H), 3.88-3.83 (m, 1H), 3.77 (s, 3H), 3.28 (dd, *J* = 10.8, 14.3, 1H), 1.23 (s, 18H).

^{13}C NMR (100 MHz, $CDCl_3$): 171.6, 154.9, 151.6, 138.3, 122.8, 122.2, 117.6, 112.0, 104.7, 99.5, 82.6, 59.8, 55.3, 52.2, 28.1, 27.7.

Boc-(L)-(4-MeO)Trp-OMe (SI-25)

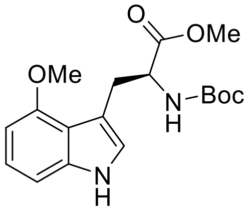

SI-25

$(Boc)_2$-(L)-(4-MeO)Trp-OMe (**SI-24**, 400 mg, 0.89 mmol, 1.0 eq.) was dissolved in acetonitrile (50 mL). Magnesium perchlorate (10 mg, 0.04 mmol, 0.05 eq.) was added and the resulting mixture was heated to 90 °C for 20 min. The organic solvent was removed under reduced pressure and the obtained crude product was purified by flash column chromatography (20% ethyl acetate in cyclohexane) to yield Boc-(L)-(4-MeO)Trp-OMe (**SI-25**) as a pale yellow solid.

Yield: 183 mg (0.89 mmol, 60%, pale yellow solid).

TLC (33% ethyl acetate in cyclohexane): R_f = 0.31;

(20% ethyl acetate in cyclohexane): R_f = 0.14.

LC-MS (ESI): t_R = 8.53 min (C_{18}); *m/z* = calcd for $C_{13}H_{16}N_2O_3^+$ $[M\text{-}Boc+H]^+$ 249.01, found 248.90, calcd for $C_{18}H_{24}N_2O_5^+$ $[M+H]^+$ 349.18 found 348.71, calcd for $NaC_{18}H_{24}N_2O_5^+$ $[M+Na]^+$ 371.16, found 371.18, calcd for $H(C_{18}H_{23}N_2O_5)_2^+$ $[2M+H]^+$ 697.35, found 696.63, calcd for $Na(C_{18}H_{23}N_2O_5)_2^+$ $[2M+Na]^+$ 719.33, found 718.57.

HRMS (ESI): *m/z* = calcd for $C_{18}H_{25}N_2O_5^+$ $[M+H]^+$ 349.1758 found 349.1757, calcd for $NaC_{18}H_{24}N_2O_5^+$ $[M+Na]^+$ 371.1577, found 371.1580.

$[\alpha]_D^{22}$ ([c] = 1 in $CHCl_3$): -139.

^{1}H NMR (400 MHz, $CDCl_3$): δ 8.11 (s, 1H), 7.09 (t, *J* = 8.0, 1H), 6.97 (s, 1H), 6.90 (d, *J* = 8.1, 1H), 6.51 (d, *J* = 7.8, 1H), 5.73 (br, 1H), 4.49 (m, 1H), 3.97 (s, 3H), 3.70 (s, 3H), 3.39 (m, 1H), 3.26 (m, 1H), 1.33 (s, 9H).

^{13}C NMR (100 MHz, CDCl3): δ 173.5, 155.7, 154.2, 138.1, 123.0, 122.0, 117.6, 111.1, 104.9, 99.7, 79.4, 55.8, 55.2, 52.1, 28.4, 27.1.

Boc-(L)-(4-MeO)Trp-OH (8)

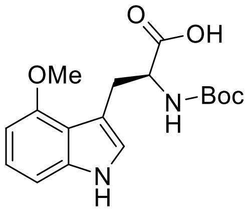

8

Boc-(L)-(4-MeO)Trp-OH (**8**) was synthesized following the *Ester Hydrolysis General Procedure (LiOH)* utilizing Boc-(L)-(4-MeO)Trp-OMe (**SI-25**, 83 mg, 0.24 mmol, 1.0 eq.), lithium hydroxide (17 mg, 0.71 mmol, 3.0 eq.), methanol (8.00 mL), water (8.00 mL) and tetrahydrofuran (2 mL). Boc-(L)-(4-MeO)Trp-OH (**8**) was obtained as a pale yellow solid.

Yield: 72 mg (0.22 mmol, 90%, pale yellow solid).
TLC (50% ethyl acetate in cyclohexane): R_f = 0.12;
(10% methanol in dichloromethane): R_f = 0.40.
LC-MS (ESI): t_R = 7.36 min (C_{18}); *m/z* = calcd for $C_{12}H_{14}N_2O_3^+$ $[M\text{-}Boc+H]^+$ 234.10 found 234.96, calcd for $C_{17}H_{23}N_2O_5^+$ $[M+H]^+$ 335.16 found 334.79, calcd for $NaC_{17}H_{22}N_2O_5^+$ $[M+Na]^+$ 357.14 found 357.11, calcd for $H(C_{17}H_{22}N_2O_5)_2^+$ $[2M+H]^+$ 669.31 found 668.46, calcd for $Na(C_{17}H_{22}N_2O_5)_2^+$ $[2M+Na]^+$ 690.30 found 690.67, calcd for $C_{29}H_{36}N_4O_8^+$ $[2M\text{-}Boc+H]^+$ 568.25 found 568.62.
HRMS (ESI): *m/z* = calcd for $C_{17}H_{21}N_2O_5^-$ $[M\text{-}H]^-$ 333.1445, found 333.1302.
$[\alpha]_D^{23}$ ([c] = 1 in $CHCl_3$): -44.
^{1}H NMR (400 MHz, $CDCl_3$): δ 8.18 (br, 1H), 7.10 (t, *J* = 7.8, 1H), 7.00-6.96 (m, 1H), 6.96 (d, *J* = 6.9, 1H), 6.52 (d, *J* = 7.7, 1H), 6.09 (br, 1H), 4.45 (br, 1H), 3.97 (s, 3H), 3.89-3.83 (m, 1H), 3.75-3.72 (m, 1H), 1.36 (s, 9H).
^{13}C NMR (100 MHz, $CDCl_3$): δ 176.9, 156.5, 153.8, 138.1, 122.9, 117.4, 110.3, 105.2, 99.7, 80.2, 57.2, 55.1, 53.6, 29.8, 28.4.

Chemical synthesis of Fragment C

The synthesis of fragment C was performed as described in **Fig. 2c** *via* the following intermediates:

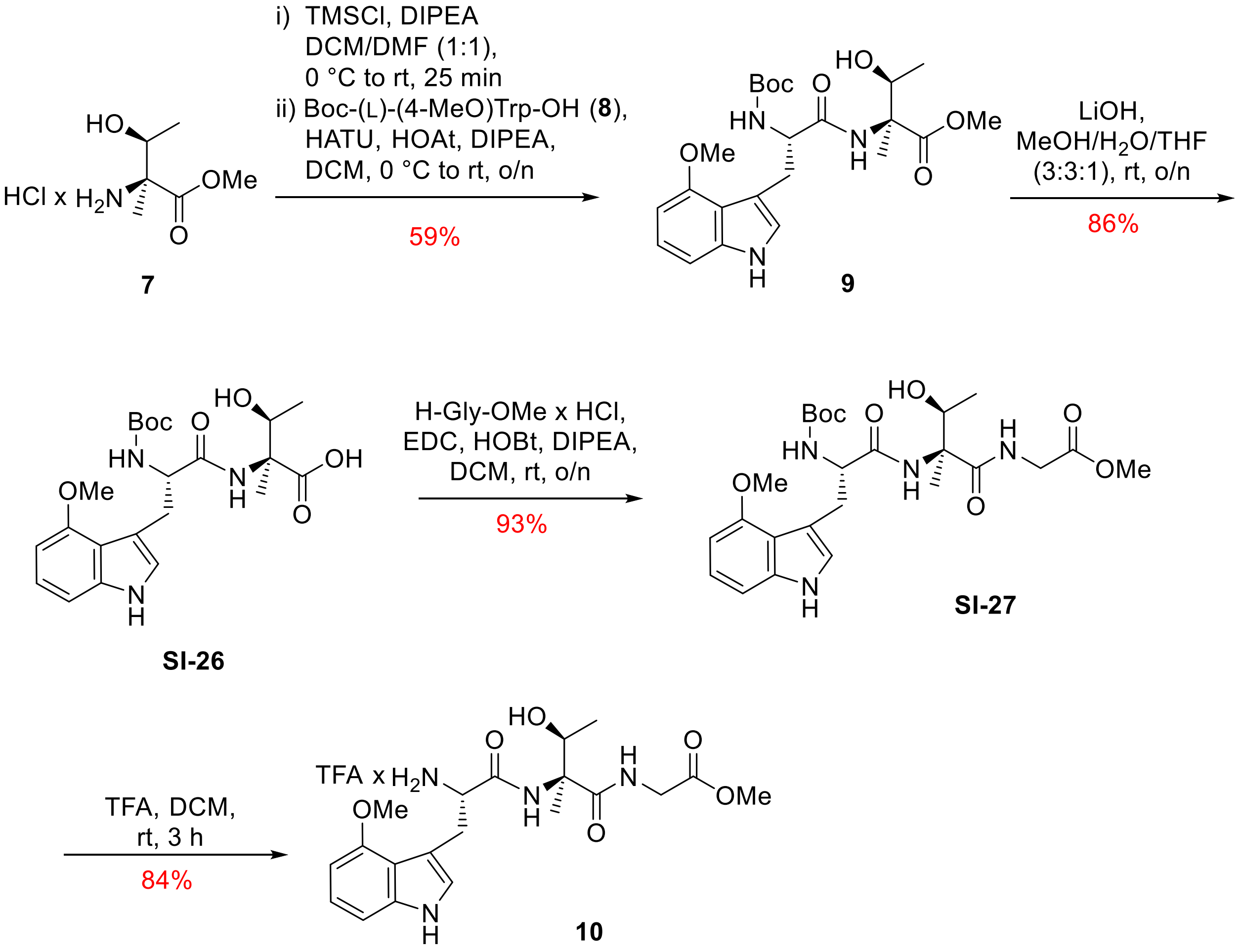

Synthesis of Boc-(L)-(4-MeO)Trp-(2*S*,3*S*-α-Me)Thr-OMe (9)

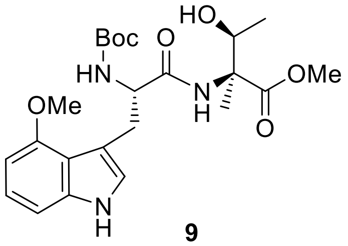

9

Boc-(L)-(4-MeO)Trp-(2*S*,3*S*-α-Me)Thr-OMe (**9**) was synthesized following the *Peptide-Coupling General Procedure (TMS-Activation)* utilizing (2*S*,3*S*) 2-methylthreonine methyl ester hydrochloride (**7**, 95 mg, 0.52 mmol, 1.0 eq.), TMSCl (197 mg, 1.81 mmol, 229 µL, 3.5 eq.), DIPEA (334 mg, 2.59 mmol, 451 µL, 5.0 eq.) and dichloromethane

(15 mL) in addition to Boc-(L)-(4-MeO)Trp-OH (**8**, 138 mg, 0.41 mmol, 0.8 eq.), HOAt (211 mg, 1.55 mmol, 3 eq.), HATU (590 mg, 1.55 mmol, 3.0 eq.), DIPEA (451 mg, 2.59 mmol, 451 µL, 5.0 eq.) and dichloromethane (15 mL). The crude product was purified by flash column chromatography (50% ethyl acetate in cyclohexane) to afford Boc-(L)-(4-MeO)Trp-(2*S*,3*S*-α-Me)-Thr-OMe (**9**) as a colorless solid. Although the compound turned red upon storage in $CDCl_3$, no change in the analytical data occurred.

Yield: 142 mg (0.13 mmol, 59%, colorless solid).

TLC (50% ethyl acetate in cyclohexane): R_f = 0.22,
(66% ethyl acetate in cyclohexane): R_f = 0.40.

LC-MS (ESI): t_R = 7.74 (C_{18}); *m/z* = calcd for $C_{18}H_{25}N_3O_5^+$ [M-Boc+H]$^+$ 363.18, found 364.20, calcd for $C_{23}H_{34}N_3O_7^+$ [M+H]$^+$ 464.24, found 464.20, calcd for $NaC_{23}H_{33}N_3O_7^+$ [M+Na]$^+$ 486.22, found 486.20, calcd for $Na(C_{23}H_{33}N_3O_7)_2^+$ [2M+Na]$^+$ 949.45, found 949.40.

HRMS (ESI): *m/z* = calcd for $C_{23}H_{34}N_3O_7^+$ [M+H]$^+$ 464.2391, found 464.2394, calcd for $NaC_{23}H_{33}N_3O_7^+$ [M+Na]$^+$ 486.2211, found 486.2217, calcd for $Na(C_{23}H_{33}N_3O_7)_2^+$ [2M+Na]$^+$ 949.4535, found 949.4545.

^{1}H NMR (400 MHz, $CDCl_3$): δ 8.21 (s, 1H), 7.39 (s, 1H), 7.10 (t, *J* = 7.9, 1H), 6.99 (d, *J* = 8.1, 1H), 6.96 (s, 1H), 6.55 (d, *J* = 7.7, 1H), 6.15 (s, 1H), 5.53 (s, 1H), 4.45 (q, *J* = 6.5, 1H), 4.37 (q, *J* = 6.2, 1H), 4.02 (s, 3H), 3.73 (s, 3H), 3.39-3.27 (m, 2H), 1.36 (s, 9H), 1.34 (s, 3H), 0.89 (d, *J* = 6.5, 3H).

^{13}C NMR (100 MHz, $CDCl_3$): δ 173.8, 173.7, 153.7, 138.2, 123.2, 122.9, 117.3, 110.4, 107.3, 105.3, 99.7, 83.2, 71.1, 65.7, 60.5, 55.2, 53.3, 30.2, 28.3, 25.8, 23.8.

Synthesis Boc-(L)-(4-MeO)Trp-(2*S*,3*S*-α-Me)Thr-OH (SI-26)

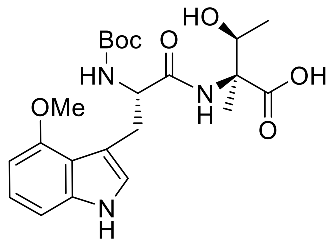

SI-26

Boc-(L)-(4-MeO)Trp-(2*S*,3*S*-α-Me)Thr-OH (**SI-26**) was synthesized following the *Ester Hydrolysis General Procedure (LiOH)* utilizing Boc-(L)-(4-MeO)Trp-(2*S*,3*R*-α-Me)Thr-OMe (**9**, 45 mg, 0.10 mmol, 1.0 eq.), lithium hydroxide (7 mg, 0.29 mmol, 3.0 eq.), methanol (1.50 mL), water (1.50 mL) and tetrahydrofuran (0.5 mL). The crude product was purified by HPLC to obtain pure Boc-(L)-(4-MeO)Trp-(2*S*,3*S*-α-Me)Thr-OH (**SI-26**) as a colorless solid.

Yield: 38 mg (0.08 mmol, 86%, pale yellow solid).

TLC (10% methanol in dichloromethane): R_f = 0.18.

LC-MS (ESI): t_R = 7.02 (C_{18}); *m/z* = calcd for $C_{17}H_{23}N_3O_5^+$ [M-Boc+H]$^+$ 349.16, found 350.23, calcd for $C_{22}H_{32}N_3O_7^+$ [M+H]$^+$ 450.22, found 450.2, calcd for $NaC_{22}H_{31}N_3O_7^+$ [M+Na]$^+$ 472.21, found 472.23, calcd for $H(C_{22}H_{31}N_3O_7)_2^+$ [2M+H]$^+$ 899.44, found 898.65, calcd for $Na(C_{22}H_{31}N_3O_7)_2^+$ [2M+Na]$^+$ 921.42, found 920.83.

HRMS (ESI): *m/z* = calcd for $C_{22}H_{32}N_3O_7^+$ [M+H]$^+$ 450.2235, found 450.2229, calcd for $NaC_{22}H_{31}N_3O_7^+$ [M+Na]$^+$ 472.2054, found 472.2134.

$[\alpha]_D^{22}$ ([c] = 1 in $CHCl_3$): -4.

^{1}H NMR (400 MHz, $CDCl_3$): δ 9.48 (s, 1H), 7.02 (t, *J* = 7.9, 1H), 6.97 (d, *J* = 8.2, 1H), 6.92 (s, 1H), 6.68 (s, 1H), 6.47 (d, *J* = 7.8, 1H), 4.22 (s, 1H), 3.92 (s, 3H), 3.82 (s, 1H), 3.26 (s, 3H), 1.34 (s, 9H), 1.17 (s, 3H), 0.96 (d, *J* = 6.4, 3H).

^{13}C NMR (100 MHz, $CDCl_3$): δ 178.2, 172.9, 156.8, 153.4, 138.3, 122.3, 117.2, 109.0, 105.8, 99.3, 80.4, 71.0, 64.3, 57.7, 55.0, 28.9, 28.3, 19.6, 18.1.

Synthesis of Boc-(L)-(4-MeO)Trp-(2*S*,3*S*-α-Me)Thr-Gly-OMe (SI-27)

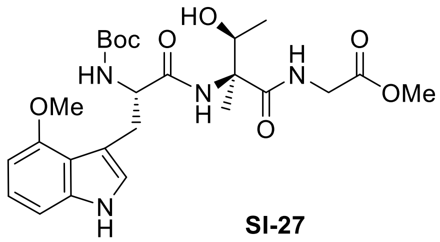

SI-27

Boc-(L)-(4-MeO)Trp-(2*S*,3*S*-α-Me)Thr-Gly-OMe (**SI-27**) was synthesized following the *Peptide-Coupling General Procedure (EDC)* utilizing Boc-(L)-(4-MeO)Trp-(2*S*,3*S*-α-Me) Thr-OH (**SI-26**, 63 mg, 0.14 mmol, 1.0 eq.), H-Gly-OMe hydrochloride salt (26 mg, 0.21 mmol, 1.5 eq.), EDC (81 mg, 0.42 mmol, 3.0 eq.), HOBt (57 mg, 0.42 mmol, 3.0 eq.), DIPEA (91 mg, 0.70 mmol, 122 µL, 5.0 eq.) and dichloromethane (3 mL). The crude product was purified by flash column chromatography (5% methanol in dichloromethane) to afford Boc-(L)-(4-MeO)Trp-(2*S*,3*S*-α-Me)Thr-Gly-OMe (**SI-27**) as a colorless solid.

Yield: 68 mg (0.13 mmol, 93%, colorless solid).

TLC (5% methanol in dichloromethane): Rf = 0.43.

LC-MS (ESI): t_R = 7.45 (C_{18}); *m/z* = calcd for $C_{20}H_{28}N_4O_6^+$ [M-Boc+H]$^+$ 421.21, found 421.13, calcd for $C_{25}H_{37}N_4O_8^+$ [M+H]$^+$ 521.26, found 521.30, calcd for $NaC_{25}H_{36}N_4O_8^+$ [M+Na]$^+$ 543.24, found 521.30, calcd for $H(C_{25}H_{36}N_4O_8)_2^+$ [2M+H]$^+$ 1041.51, found 1040.66, calcd for $Na(C_{25}H_{36}N_4O_8)_2^+$ [2M+Na]$^+$ 1063.50, found 1062.97.

HRMS (ESI): *m/z* = calcd for $C_{25}H_{37}N_4O_8^+$ [M+H]$^+$ 521.2606, found 521.2606, calcd for $NaC_{25}H_{36}N_4O_8^+$ [M+Na]$^+$ 543.2425, found 543.2434, calcd for $Na(C_{25}H_{36}N_4O_8)_2^+$ [2M+Na]$^+$ 1063.4964, found 1063.4955.

$[\propto]_{\mathbf{D}}^{\mathbf{23}}$ ([c] = 1 in $CHCl_3$): -9.

^{1}H NMR (400 MHz, $CDCl_3$): δ 8.31 (s, 1H), 8.02 (s, 1H), 7.80 (br, 1H), 7.13 (t, *J* = 8.0, 1H), 7.04 (d, *J* = 8.0, 1H), 6.94 (s, 2H), 6.58 (d, *J* = 7.7, 1H), 4.49-4.36 (m, 1H), 4.12-4.06 (m, 1H), 4.05-4.01 (m, 1H), 4.02 (s, 3H), 3.70 (s, 3H), 3.68-3.60 (m, 1H), 3.41-3.36 (m, 1H), 3.32-3.19 (m, 1H), 1.51 (s, 3H), 1.36 (s, 8H), 1.12 (d, *J* = 6.3, 3H).

^{13}C NMR (100 MHz, $CDCl_3$): δ 172.8, 171.8, 171.7, 162.7, 157.6, 153.3, 138.2, 123.6, 123.0, 117.2, 109.9, 105.6, 99.9, 80.5, 73.0, 64.0, 58.7, 55.1, 52.7, 41.3, 28.3, 28.0, 18.5, 17.2.

H-(L)-(4-MeO)Trp-(2*S*,3*S*-α-Me)Thr-Gly-OMe trifluoroacetate salt (10)

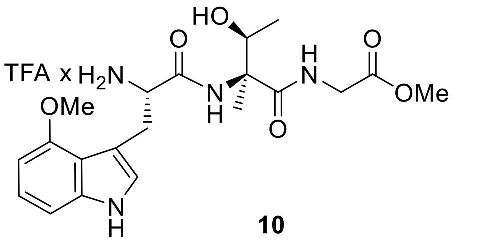

10

H-(L)-(4-MeO)Trp-(2*S*,3*S*-α-Me)Thr-Gly-OMe trifluoroacetate salt (**10**) was synthesized following the *Boc-Deprotection General Procedure (TFA)* utilizing Boc-(L)-(4-MeO)Trp-(2*S*,3*S*-α-Me)Thr-Gly-OMe (**SI-27**, 63 mg, 0.12 mmol, 1.0 eq.), dichloromethane (2 mL) and trifluoroacetic acid (1 mL) to afford H-(L)-(4-MeO)Trp-(2*S*,3*S*-α-Me)Thr-Gly-OMe trifluoroacetate salt (**10**) as a pale green solid.

Yield: 54 mg (0.1 mmol, 84%, pale green solid).

TLC (5% methanol in dichloromethane): R_f = 0.34.

LC-MS (ESI): t_R = 4.33 (C_{18}); *m/z* = calcd for $C_{20}H_{29}N_4O_6^+$ $[M+H]^+$ 421.21, found 421.20, calcd for $NaC_{20}H_{28}N_4O_6^+$ $[M+Na]^+$ 443.19, found 443.20.

HRMS (ESI): *m/z* = calcd for $C_{20}H_{29}N_4O_6^+$ $[M+H]^+$ 421.2082, found 421.2082, calcd for $NaC_{20}H_{28}N_4O_6^+$ $[M+Na]^+$ 443.1901, found 443.1896.

$[\propto]_D^{23}$ ([c] = 1 in MeOH): -11.

^{1}H NMR (700 MHz, MeOD): δ 7.06 (s, 1H), 7.05 (t, *J* = 4.0, 1H), 6.99 (d, *J* = 8.1, 1H), 6.57 (d, *J* = 7.7, 1H), 4.32 (t, *J* = 7.4, 1H), 4.16-4.09 (m, 1H), 4.01 (s, 3H), 3.97-3.92 (m, 1H), 3.89-3.84 (m, 1H), 3.72 (s, 3H), 3.51-3.45 (m, 1H), 3.24-3.18 (m, 1H), 1.29 (s, 3H), 0.98 (d, *J* = 6.4, 3H).

^{13}C NMR (176 MHz, MeOD): δ 174.8, 171.7, 169.9, 155.3, 140.2, 124.4, 124.0, 117.8, 108.5, 106.4, 100.5, 70.7, 65.0, 56.2, 55.8, 52.6, 42.2, 30.6, 18.8, 17.9.

Assembly of the three fragments to Zelkovamcin (1)

The synthesis of Zelkovamycin (**1**) was carried out as described in **Fig. 3** *via* the following intermediates:

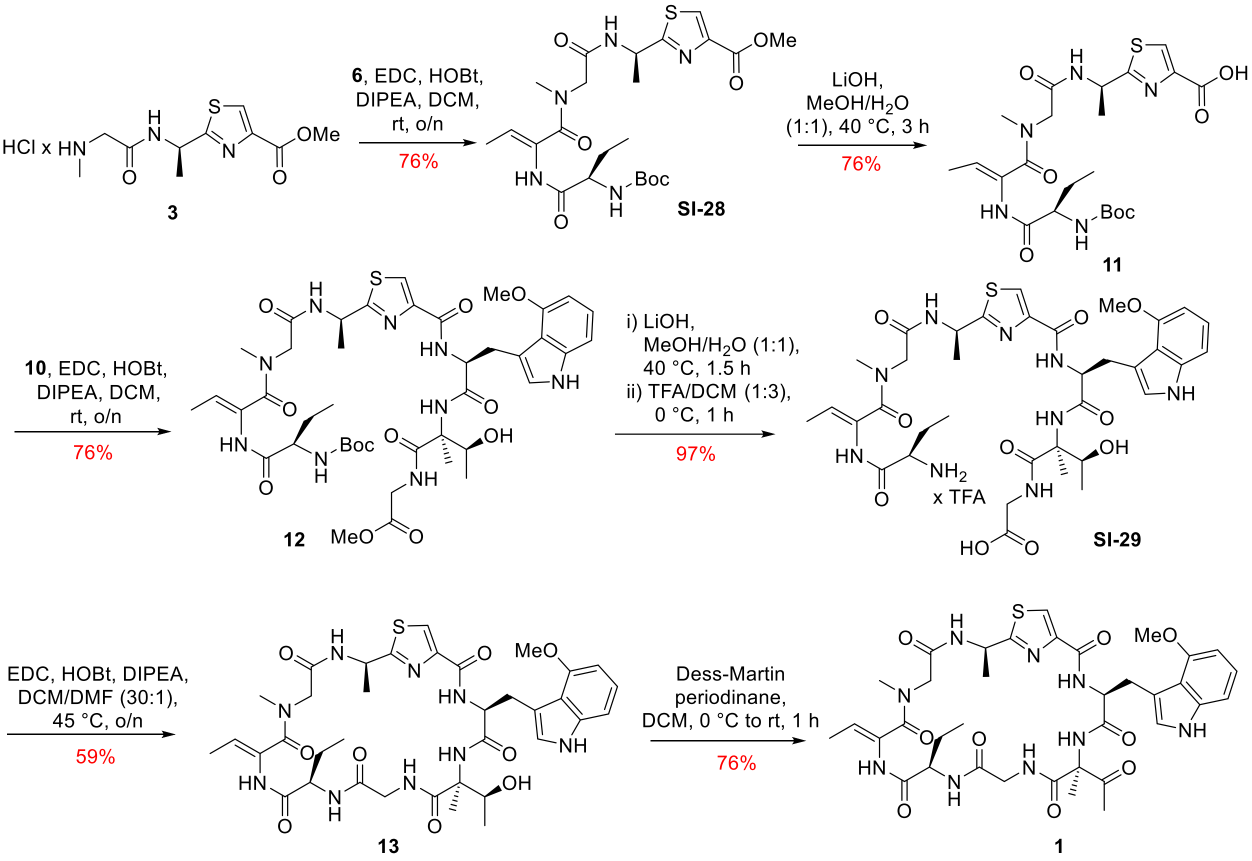

Methyl 2-((6*R*,15*R*,*E*)-6-ethyl-9-ethylidene-2,2,11-trimethyl-4,7,10,13-tetraoxo-3-oxa-5,8,11,14-tetraazahexadecan-15-yl)thiazole-4-carboxylate (SI-28)

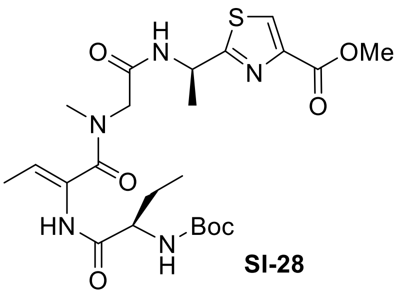

SI-28

Methyl 2-((6*R*,15*R*,*E*)-6-ethyl-9-ethylidene-2,2,11-trimethyl-4,7,10,13-tetraoxo-3-oxa-5,8,11,14-tetraazahexadecan-15-yl)thiazole-4-carboxylate (**SI-28**) was synthesized following the *Peptide-Coupling General Procedure (EDC)* utilizing methyl (*R*)-2-(1-(2-

(methylamino)acetamido)ethyl)thiazole-4-carboxylate hydrochloride salt (**3**, 92 mg, 0.31 mmol, 1.2 eq.), Boc-(D)-Abu-(*Z*)-Dhb-OH (**6**, 75 mg, 0.26 mmol, 1.0 eq.), EDC (151 mg, 0.79 mmol, 3.0 eq.), HOBt (106 mg, 0.79 mmol, 3.0 eq.), DIPEA (169 mg, 1.31 mmol, 228 µL, 5.0 eq.) and dichloromethane (10 mL). The crude product was purified by flash column chromatography (5% MeOH in DCM) to afford methyl 2-((6*R*,15*R*,*E*)-6-ethyl-9-ethylidene-2,2,11-trimethyl-4,7,10,13-tetraoxo-3-oxa-5,8,11,14-tetraazahexadecan-15-yl)thiazole-4-carboxylate (**SI-28**) as a colorless solid.

Yield: 105 mg (0.2 mmol, 76%, colorless solid).

TLC (5% methanol in dichloromethane): R_f = 0.27,

(10% methanol in dichloromethane): R_f = 0.62.

LC-MS (ESI): t_R = 6.68 min (C_{18}); *m/z* = calcd for $NaC_{23}H_{35}N_5O_7S^+$ $[M+Na]^+$ 548.21, found 548.19, calcd for $C_{23}H_{36}N_5O_7S^+$ $[M+H]^+$ 526.23, found 526.19, calcd for $H(C_{23}H_{35}N_5O_7S)_2^+$ $[2M+H]^+$ 1051.46, found 1050.1.

HRMS (ESI): *m/z* = calcd for $C_{23}H_{35}N_5O_7S^+$ $[M+H]^+$ 526.2330, found 526.2327, calcd for $NaC_{23}H_{35}N_5O_7S^+$ $[M+Na]^+$ 548.2149, found 548.2158.

$[\alpha]_D^{25}$ ([c] = 1 in $CDCl_3$): +47.

^{1}H NMR (400 MHz, $CDCl_3$): δ 9.05 (br, 1H), 8.10 (s, 1H), 7.96 (s, 1H), 7.89 (s, 1H), 5.36-5.29 (m, 1H), 5.25-5.18 (m, 1H), 4.04-3.98 (m, 1H), 3.80 (s, 3H), 3.05 (s, 2H), 2.84 (s, 2H), 2.75 (s, 2H), 1.61 (d, *J* = 7.1, 3H), 1.57 (d, *J* = 7.0, 3H), 1.29 (s, 9H), 0.77 (t, *J* = 7.4, 3H).

^{13}C NMR (100 MHz, $CDCl_3$): δ 175.5, 168.3, 168.2, 162.4, 161.7, 155.8, 146.4, 129.2, 127.4, 116.5, 80.0, 52.1, 51.2, 47.7, 38.2, 36.4, 31.3, 28.1, 20.4, 11.5, 9.7.

2-((6*R*,15*R*,*E*)-6-ethyl-9-ethylidene-2,2,11-trimethyl-4,7,10,13-tetraoxo-3-oxa-5,8,11,14-tetraazahexadecan-15-yl)thiazole-4-carboxylic acid (11)

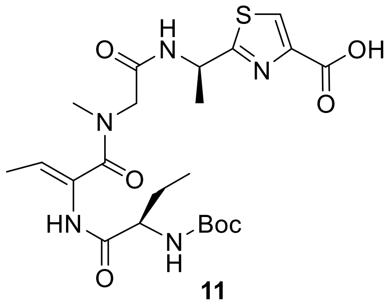

11

2- ((6*R*,15*R*,*E*)- 6 -ethyl- 9-ethylidene-2,2,11-trimethyl- 4,7,10,13 -tetraoxo-3-oxa-5,8,11,14-tetraazahexadecan-15-yl)thiazole-4-carboxylic acid (**11**) was synthesized following the *Ester Hydrolysis General Procedure (LiOH)* utilizing methyl 2-((6*R*,15*R*,*E*)-6-ethyl-9-ethylidene-2,2,11-trimethyl-4,7,10,13-tetraoxo-3-oxa-5,8,11,14-tetraazahexadecan-15-yl)thiazole-4-carboxylate (**SI-28**, 80 mg, 0.15 mmol, 1.0 eq.), lithium hydroxide (11 mg, 0.46 mmol, 1.5 eq.), methanol (3.20 mL) and water (3.20 mL). 2-((6*R*,15*R*,*E*)-6-ethyl-9-ethylidene-2,2,11-trimethyl-4,7,10,13-tetraoxo-3-oxa-5,8,11,14-tetraazahexadecan-15-yl)thiazole-4-carboxylic acid (**11**) was obtained as a colorless solid.

Yield: 71 mg (0.14 mmol, 92%, colorless solid).

TLC (10% methanol in dichloromethane): R_f = 0.31.

LC-MS (ESI): t_R = 5.86 min (C_{18}); *m/z* = calcd for $C_{22}H_{34}N_5O_7S^+$ [M+H]$^+$ 512.22, found 511.75, calcd for $H(C_{22}H_{33}N_5O7S)_2^+$ [2M+H]$^+$ 1023.43, found 1022.44, calcd for $NaC_{22}H_{33}N_5O_7S^+$ [M+Na]$^+$ 534.20, found 534.18.

HRMS (ESI): *m/z* = calcd for $C_{22}H_{34}N_5O_7S^+$ [M+H]$^+$ 512.2173, found 512.2159, calcd for $NaC_{22}H_{33}N_5O_7S^+$ [M+Na]$^+$ 534.1993, found 534.1982, calcd for $Na(C_{22}H_{33}N_5O_7S)_2^+$ [2M+Na]$^+$ 1045.4094, found 1045.4100.

$[\propto]_{\mathbf{D}}^{\mathbf{25}}$ ([c] = 1 in MeOH): -53.

^{1}H NMR (400 MHz, MeOD): δ 8.24 (s, 1H), 5.49-5.48 (m, 1H), 5.36-5.34 (m, 1H), 4.39-4.18 (m, 1H), 4.13-3.93 (m, 2H), 3.18 (s, 3H), 1.80 (d, *J* = 7.0, 3H), 1.74-1.69 (m, 1H), 1.66 (d, *J* = 6.9, 3H), 1.64-1.57 (m, 1H), 1.42 (s, 9H), 0.96 (t, *J* = 6.7, 3H).

^{13}C NMR (100 MHz, MeOD): δ 176.9, 173.8, 170.8, 170.6, 164.0, 157.9, 148.4, 130.7, 128.9, 119.5, 80.6, 61.5, 57.0, 52.2, 39.1, 28.7, 26.4, 20.9, 12.0, 10.7.

Methyl ((2*S*,3*S*)-2- ((*S*)- 2- (2- ((*R*) -1-(2- ((*Z*)-2- ((*R*) -2-((*tert*-butoxycarbonyl) amino)butanamido)-N-methylbut-2-enamido) acetamido) ethyl) thiazole-4-carbox amido)-3-(4-methoxy-1H-indol-3-yl) propanamido)-3- hydroxy-2- methyl butanoyl) glycinate (12)

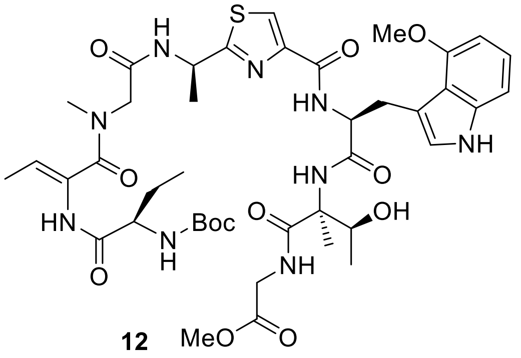

12

Methyl ((2*S*,3*S*)-2-((*S*)-2-(2-((*R*)-1-(2-((*Z*)-2-((*R*)-2-((*tert*-butoxycarbonyl) amino) butanamido)-N-methylbut-2-enamido)acetamido) ethyl) thiazole-4-carboxamido)-3-(4-methoxy-1H-indol-3-yl) propanamido)-3-hydroxy-2-methylbutanoyl)glycinate (**12**) was synthesized following the modified *Peptide-Coupling General Procedure (EDC)* utilizing H-(L)-(4-MeO)Trp-(2*S*,3*S*-α-Me)-Thr-Gly-OMe trifluoroacetate salt (**10**, 9 mg, 0.02 mmol, 1.0 eq.), 2-((6*R*,15*R*,*E*) -6-ethyl-9-ethylidene-2,2,11-trimethyl-4,7,10,13-tetraoxo-3-oxa-5,8,11,14-tetraazahexadecan-15-yl)thiazole-4-carboxylic acid (**11**, 9 mg, 0.02 mmol, 1.0 eq.), EDC (10 mg, 0.05 mmol, 3.0 eq.), HOBt (7 mg, 0.05 mmol, 3.0 eq.), DIPEA (11 mg, 0.09 mmol, 15 µL, 5.0 eq.) and dichloromethane (5 mL). The crude product was purified by flash column chromatography (6% methanol in dichloromethane) to afford methyl ((2*S*,3*S*)-2- ((*S*)- 2- (2- ((*R*) -1-(2- ((*Z*)-2- ((*R*) -2-((*tert*-butoxycarbonyl) amino)butanamido)-N-methylbut-2-enamido) acetamido) ethyl) thiazole-4-carbox amido)-3-(4-methoxy-1H-indol-3-yl) propanamido)-3- hydroxy-2-methyl butanoyl) glycinate (**12**) as a colorless solid.

Yield: 11.1 mg (0.02 mmol, 69%, colorless solid).

TLC (10% methanol in dichloromethane): R_f = 0.62.

LC-MS (ESI): t_R = 7.08 (C_{18}); *m/z* = calcd for $C_{42}H_{60}N_9O_{12}S^+$ $[M+H]^+$ 914.41, found 914.13, calcd for $NaC_{42}H_{60}N_9O_{12}S^+$ $[M+Na]^+$ 936.39, found 936.32, calcd for $Na(C_{42}H_{59}N_9O_{12}S)_2^+$ $[2M+Na]^+$ 1849.79, found 1849.89.

HRMS (ESI): *m/z* = calcd for $C_{42}H_{60}N_9O_{12}S^+$ $[M+H]^+$ 914.4077, found 914.4076, calcd for $NaC_{42}H_{60}N_9O_{12}S^+$ $[M+Na]^+$ 936.3896, found 936.3893.

$[\propto]_{\mathbf{D}}^{\mathbf{23}}$ ([c] = 1 in MeOH): -2.

^{1}H NMR (700 MHz, MeOD): δ 8.04 (s, 1H), 7.05 (s, 1H), 7.01 (t, *J* = 8.0, 1H), 6.95 (d, *J* = 8.0, 1H), 6.55 (d, *J* = 7.7, 1H), 5.52-5.44 (m, 1H), 5.38-5.32 (m, 1H), 4.70-4.64 (m, 1H), 4.57 (s, 2H), 4.24-4.13 (m, 1H), 4.08-4.04 (m, 1H), 4.01 (s, 3H), 3.94 (d, *J* = 17.5, 1H), 3.85 (d, *J* = 17.4, 1H), 3.70 (s, 3H), 3.50-3.45 (m, 1H), 3.44-3.38 (m, 1H), 3.15 (s, 3H), 1.79 (d, *J* = 7.1, 3H), 1.71-1.63 (m, 2H), 1.60 (d, *J* = 7.1, 3H), 1.47 (s, 3H), 1.36 (s, 9H), 1.03 (d, *J* = 6.4, 3H), 0.85 (t, *J* = 7.6, 3H).

^{13}C NMR (176 MHz, MeOD): δ 176.6, 176.2, 175.4, 174.1, 173.7, 173.3, 171.9, 170.8, 170.6, 163.9, 157.9, 155.4, 150.0, 139.8, 130.7, 125.3, 123.8, 123.5, 118.5, 110.7, 106.1, 100.2, 80.7, 71.4, 64.4, 58.3, 55.7, 54.8, 52.6, 52.3, 49.0, 46.1, 42.1, 29.5, 28.7, 26.3, 21.7, 21.1, 19.1, 17.6, 12.0, 10.6.

((2*S*,3*S*) -2-((*S*)-2-(2-((*R*) -1-(2-((*Z*)-2-((*R*)-2- aminobutanamido) -N-methylbut-2-enamido) acetamido) ethyl) thiazole-4-carboxamido) -3-(4-methoxy-1H-indol-3-yl)propanamido) -3-hydroxy-2-methylbutanoyl) glycine trifluoroacetate (SI-29)

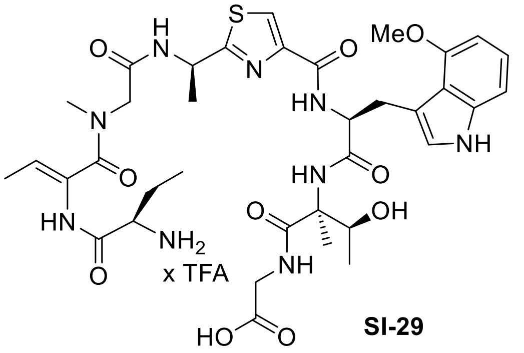

SI-29

((2*S*,3*S*) -2-((*S*)-2-(2-((*R*) -1-(2-((*Z*)-2-((*R*)-2- aminobutanamido) -N-methylbut-2-enamido) acetamido) ethyl) thiazole-4-carboxamido) -3-(4-methoxy-1H-indol-3-yl)propanamido) -3-hydroxy-2-methylbutanoyl) glycine trifluoroacetate (**SI-29**) was synthesized over two steps:

((2*S*,3*S*)-2-((*S*)-2-(2-((*R*)-1-(2-((*Z*)-2-((*R*)-2-((*tert*-butoxycarbonyl) amino) butanamido)-N-methylbut-2-enamido)acetamido)ethyl) thiazole-4-carboxamido) -3-(4-methoxy-1H-indol-3-yl)propanamido) -3-hydroxy-2-methylbutanoyl) glycine was synthesized following the *Ester Hydrolysis General Procedure (LiOH)* utilizing methyl ((2*S*,3*S*)-2-((*S*)-2-(2-((*R*)-1-(2-((*Z*) -2-((*R*) -2-((*tert*-butoxycarbonyl) amino)

butanamido) -N-methylbut-2-enamido) acetamido) ethyl) thiazole-4-carboxamido)-3-(4-methoxy-1H-indol-3-yl) propanamido)-3-hydroxy-2-methylbutanoyl)glycinate (**12**, 7 mg, 0.01 mmol, 1.0 eq.), lithium hydroxide (1 mg, 0.02 mmol, 3.0 eq.), methanol (2.00 mL) and water (2.00 mL). The mixture was acidified by the addition of potassium bisulfate (sat., aq., 1 mL), the organic solvent was removed under reduced pressure, the remaining aqueous phase was extracted with dichloromethane (5x) and the combined organic phases were dried over $MgSO_4$ and concentrated under reduced pressure to afford ((2*S*,3*S*)-2-((*S*)-2-(2-((*R*)-1-(2-((*Z*)-2- ((*R*)-2- ((*tert*-butoxycarbonyl) amino) butanamido) -N-methylbut-2-enamido) acetamido) ethyl) thiazole-4-carboxamido) -3-(4-methoxy-1H-indol-3-yl)propanamido) -3-hydroxy-2-methylbutanoyl) glycine as a colorless solid. The obtained product was used without further purification.

TLC (10% methanol in dichloromethane): R_f = 0.09.

LC-MS (ESI): t_R = 6.75 (C_{18}); *m/z* = calcd for $C_{41}H_{58}N_9O_{12}S^+$ $[M+H]^+$ 900.39, found 900.11, calcd for $NaC_{41}H_{57}N_9O_{12}S^+$ $[M+Na]^+$ 922.37, found 922.18, calcd for $Na(C_{41}H_{57}N_9O_{12}S)_2^+$ $[2M+Na]^+$ 1821.76, found 1821.26.

HRMS (ESI): *m/z* = calcd for $C_{41}H_{58}N_9O_{12}S^+$ $[M+H]^+$ 900.3920, found 900.3911, calcd for $NaC_{41}H_{57}N_9O_{12}S^+$ $[M+Na]^+$ 922.3740, found 922.3744.

((2*S*,3*S*) -2-((*S*)-2-(2-((*R*) -1-(2-((*Z*)-2-((*R*)-2- aminobutanamido) -N-methylbut-2-enamido) acetamido) ethyl) thiazole-4-carboxamido) -3-(4-methoxy-1H-indol-3-yl)propanamido) -3-hydroxy-2-methylbutanoyl) glycine trifluoroacetate (**SI-29**) was synthesized following the *Boc-Deprotection General Procedure (TFA)* utilizing ((2*S*,3*S*)-2-((*S*)-2-(2-((*R*)-1-(2-((*Z*)-2- ((*R*)-2- ((*tert*-butoxycarbonyl) amino) butanamido) -N-methylbut-2-enamido) acetamido) ethyl) thiazole-4-carboxamido) -3-(4-methoxy-1H-indol-3-yl)propanamido) -3-hydroxy-2-methylbutanoyl) glycine as crude product of the previous step (7 mg, 0.01 mmol, 1 eq.) and TFA (10% in DCM). ((2*S*,3*S*) -2-((*S*)-2-(2-((*R*) -1-(2-((*Z*)-2-((*R*)-2- aminobutanamido) -N-methylbut-2-enamido) acetamido) ethyl) thiazole-4-carboxamido) -3-(4-methoxy-1H-indol-3-yl)propanamido) -3-hydroxy-2-methylbutanoyl) glycine trifluoroacetate (**SI-29**) was obtained as a colorless solid and was used in the next step without further purification.

Yield: 7 mg (0.01 mmol, 97%, colorless solid).

TLC (25% methanol in dichloromethane): R_f = 0.04.

LC-MS (ESI): t_R = 4.85 (C_{18}); *m/z* = calcd for $C_{36}H_{50}N_9O_{10}S^+$ $[M+H]^+$ 800.34, found 800.26, calcd for $NaC_{35}H_{50}N_9O_{10}S^+$ $[M+Na]^+$ 822.32, found 822.30.

HRMS (ESI): *m/z* = calcd for $C_{36}H_{50}N_9O_{10}S^+$ $[M+H]^+$ 800.3396, found 800.3399, calcd for $NaC_{35}H_{50}N_9O_{10}S^+$ $[M+Na]^+$ 822.3215, found 822.3369.

(12*Z*,4*S*,7*S*,13*R*,16*Z*,22*R*) -13-ethyl-16-ethylidene-7-((*S*) -1-hydroxyethyl) -4-((4-methoxy-1H-indol-3-yl)methyl) -7,18,22-trimethyl-3,6,9,12,15,18,21-heptaaza-1(2,4)-thiazolacyclodocosaphane-2,5,8,11,14,17,20-heptaone (13)

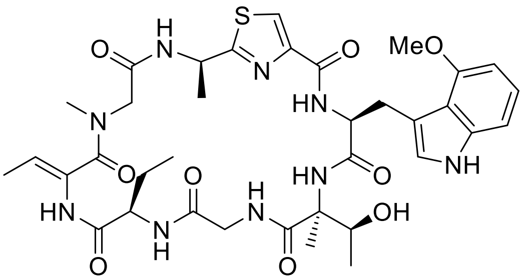

13

(12*Z*,4*S*,7*S*,13*R*,16*Z*,22*R*) -13-ethyl-16-ethylidene-7-((*S*) -1-hydroxyethyl) -4-((4-methoxy-1H-indol-3-yl)methyl) -7,18,22-trimethyl-3,6,9,12,15,18,21-heptaaza-1(2,4)-thiazolacyclodocosaphane-2,5,8,11,14,17,20-heptaone (**13**) was synthesized following the *Peptide-Coupling General Procedure (EDC)* utilizing ((2*S*,3*S*) -2-((*S*)-2-(2-((*R*) -1-(2-((*Z*)-2-((*R*)-2- aminobutanamido) -N-methylbut-2-enamido) acetamido) ethyl) thiazole-4-carboxamido) -3-(4-methoxy-1H-indol-3-yl)propanamido) -3-hydroxy-2-methylbutanoyl) glycine trifluoroacetate (**SI-29**, 48 mg, 0.06 mmol, 1.0 eq.), EDC (33 mg, 0.17 mmol, 3.0 eq.), HOBt (23 mg, 0.17 mmol, 3.0 eq.), DIPEA (37 mg, 0.29 mmol, 50 µL, 5.0 eq.) and dichloromethane (300 mL) and DMF (10 mL), 45 °C (oil bath temperature), overnight. The complete organic solvent was removed under reduced pressure and the crude product was dried under high vacuum for 1 h before the standard workup procedure was applied. The crude product was purified by HPLC to afford (12*Z*,4*S*,7*S*,13*R*,16*Z*,22*R*) -13-ethyl-16-ethylidene-7-((*S*) -1-hydroxyethyl) -4-((4-methoxy-1H-indol-3-yl)methyl) -7,18,22-trimethyl-3,6,9,12,15,18,21- heptaaza-1(2,4)- thiazolacyclodocosaphane-2,5,8,11,14, 17,20-heptaone (**13**) as a colorless solid.

Yield: 27 mg (0.03 mmol, 59%, colorless solid).

TLC (10% methanol in dichloromethane): R_f = 0.56,

(6% methanol in dichloromethane): R_f = 0.38.

LC-MS (ESI): t_R = 6.48 (C_{18}); *m/z* = calcd for $C_{36}H_{48}N_9O_9S^+$ $[M+H]^+$ 782.3, found 782.4, calcd for $NaC_{36}H_{47}N_9O_9S^+$ $[M+Na]^+$ 804.3, found 804.3.

HRMS (ESI): *m/z* = calcd for $C_{36}H_{48}N_9O_9S^+$ $[M+H]^+$ 782.3290, found 782.3306, calcd for $NaC_{36}H_{47}N_9O_9S^+$ $[M+Na]^+$ 804.3110, found 804.3124.

$[\alpha]_D^{26}$ ([c] = 1 in $CHCl_3$): +8.

^{1}H NMR (700 MHz, $CDCl_3$): δ 10.71 (s, 1H), 9.30 (s, 1H), 9.14 (d, *J* = 7.5, 1H), 9.10 (d, *J* = 8.3, 1H), 8.13 (s, 1H), 7.98 (s, 1H), 7.43 (d, *J* = 6.6, 1H), 7.07-7.02 (m, 1H), 6.82 (d, *J* = 8.1, 1H), 6.50 (d, *J* = 7.8, 1H), 5.70 (t, *J* = 6.2, 1H), 5.49 (t, *J* = 7.5, 1H), 5.24 (t, *J* = 6.8, 2H), 5.00 (d, *J* = 17.3, 1H), 4.52 (d, *J* = 14.7, 1H), 4.13 (m, 1H), 3.87 (s, 3H), 3.75-3.70 (m, 1H), 3.68 (m, 1H), 3.54 (d, *J* = 17.3, 1H), 3.15 (s, 3H), 2.02 (m, 2H), 1.91 (d, *J* = 7.1, 3H), 1.79 (d, *J* = 7.1, 3H), 1.60 (d, *J* = 14.2, 1H), 1.49 (s, 3H), 1.24 (d, *J* = 6.3, 3H), 0.92 (t, *J* = 7.4, 3H).

^{13}C NMR (176 MHz, $CDCl_3$): δ 172.3, 171.6, 171.5, 170.5, 169.5, 169.4, 168.8, 161.0, 153.8, 149.5, 136.7, 129.9, 124.3, 123.7, 123.0, 116.5, 111.7, 106.3, 105.7, 100.3, 71.8, 65.5, 55.8, 54.9, 53.3, 51.0, 45.9, 42.0, 37.9, 29.4, 21.3, 20.5, 20.0, 17.4, 11.5, 10.4.

Zelkovamycin (1)

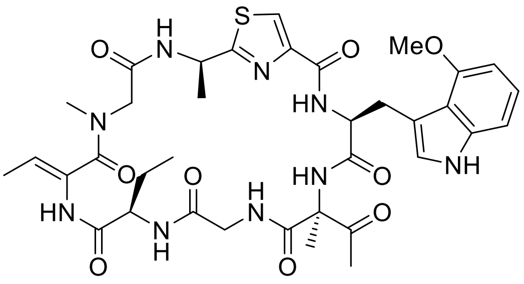

1

(12*Z*,4*S*,7*S*,13*R*,16*Z*,22*R*) -13-ethyl-16-ethylidene-7-((*S*) -1-hydroxyethyl) -4-((4-methoxy-1H-indol-3-yl)methyl) -7,18,22-trimethyl-3,6,9,12,15,18,21-heptaaza-1(2,4)-thiazolacyclodocosaphane-2,5,8,11,14,17,20-heptaone (**13**, 1 mg, 12.8 µmol, 1.0 eq.)

was dissolved under argon atmosphere in dry DCM (3 mL). The solution was cooled down to 0 °C (ice bath) and Dess-Martin periodinane was added (freshly prepared solution, 0.3 M in dry DCM, 43 µL, 12.8 µmol, 1.0 eq.). The mixture was stirred for 15 min at 0 °C (ice bath). The reaction mixture was warmed up to room temperature and the progress of the reaction was monitored by LC-MS. Complete consumption of the starting material was usually achieved after additional 45 min at room temperature. The reaction mixture was filtered through a syringe filter, the filter was washed with DCM (2x) and the organic solvent was removed under reduced pressure at room temperature. The obtained crude product was purified by HPLC to afford Zelkovamycin (**1**) as a pale yellow solid. This procedure was repeated five times and the pooled crude products were purified together in one HPLC run.

Yield: 3.8 mg (4.86 µmol, 76%, pale yellow solid).

TLC (10% methanol in dichloromethane): R_f = 0.52,
(5% methanol in dichloromethane): Rf = 0.19.

LC-MS (ESI): t_R = 7.29 (C_{18}); *m/z* = calcd for $C_{36}H_{46}N_9O_9S^+$ [M+H]$^+$ 780.3, found 780.3, calcd for $NaC_{36}H_{45}N_9O_9S^+$ [M+Na]$^+$ 802.3, found 802.3, calcd for $H(C_{36}H_{45}N_9O_9S)_2^+$ [2M+H]$^+$ 1559.6, found 1558.3, calcd for $Na(C_{36}H_{45}N_9O_9S)_2^+$ [M+Na]$^+$ 1581.6, found 1580.3.

HRMS (ESI): *m/z* = calcd for $C_{36}H_{46}N_9O_9S^+$ [M+H]$^+$ 780.3134, found 780.3137, calcd for $NaC_{36}H_{45}N_9O_9S^+$ [M+Na]$^+$ 802.2953, found 802.2956.

$[\propto]_D^{23}$ ([c] = 1 in $CHCl_3$): +10.

^{1}H NMR (700 MHz, $CDCl_3$): δ 10.76 (s, 1H), 9.19 (d, *J* = 8.5, 1H), 9.16 (s, 1H), 8.72 (d, *J* = 7.5, 1H), 8.17 (s, 1H), 7.96 (s, 1H), 7.04 (d, *J* = 6.3, 1H), 7.02 (t, *J* = 8.0, 1H), 6.93 (s, 1H), 6.78 (d, *J* = 8.1, 1H), 6.44 (d, *J* = 7.7, 1H), 5.74-5.69 (m, 1H), 5.53-5.46 (m, 1H), 5.25-5.20 (m, 1H), 5.22-5.17 (m, 1H), 5.02 (d, *J* = 17.2, 1H), 4.20 (dd, *J* = 15.7, 3.6, 1H), 4.12-4.06 (m, 1H), 3.88 (s, 3H), 3.63 (dd, *J* = 17.4, 7.4, 1H), 3.61-3.58 (m, 1H), 3.57 (d, *J* = 16.8, 1H), 3.11 (s, 3H), 2.24 (s, 3H), 2.05-1.99 (m, 1H), 1.99-1.93 (m, 1H), 1.87 (d, *J* = 7.1, 3H), 1.76 (d, *J* = 7.1, 3H), 1.70 (s, 3H), 1.58 (d, *J* = 5.2, 1H) , 0.91 (t, *J* = 7.4, 3H).

^{13}C NMR (176 MHz, $CDCl_3$): δ 200.3, 171.7, 171.0, 170.6, 169.2, 169.1, 169.0, 167.1, 160.5, 153.5, 149.6, 136.7, 130.0, 124.9, 124.3, 122.8,

116.1, 111.4, 106.2, 105.7, 99.5, 77.2, 69.9, 55.5, 55.0, 52.8, 50.7, 45.9, 42.4, 37.7, 28.2, 23.2, 21.3, 20.6, 19.9, 11.4, 10.3.

For comparison, we also determined the corresponding data for commercially available, isolated Zelkovamycin and found matching in all criteria:

TLC (10% methanol in dichloromethane): R_f = 0.51,
(5% methanol in dichloromethane): R_f = 0.18.

LC-MS (ESI): t_R = 7.29 (C18); *m/z* = calcd for $C_{36}H_{46}N_9O_9S^+$ $[M+H]^+$ 780.87, found 780.39, calcd for $NaC_{36}H_{45}N_9O_9S^+$ $[M+Na]^+$ 802.30, found 802.40, calcd for $H(C_{36}H_{45}N_9O_9S)_2^+$ $[2M+H]^+$ 1559.62, found 1558.49.

^{1}H NMR (700 MHz, $CDCl_3$): δ 10.84 (s, 1H), 9.30 (s, 1H), 8.95 (d, *J* = 8.6, 1H), 8.57 (d, *J* = 7.5, 1H), 8.08 (s, 1H), 7.92 (s, 1H), 7.01 (t, *J* = 7.9, 1H), 6.96 (s, 1H), 6.91 (d, *J* = 6.5, 1H), 6.78 (d, *J* = 8.3, 1H), 6.42 (d, *J* = 7.8, 1H), 5.70-5.65 (m, 1H), 5.53-5.46 (m, 1H), 5.22-5.17 (m, 1H), 5.19-5.15 (m, 1H), 5.00 (d, *J* = 17.0, 1H), 4.17 (dd, *J* = 15.5, 3.5, 1H), 4.10-4.04 (m, 1H), 3.88 (s, 3H), 3.63-3.58 (m, 1H), 3.58-3.54 (m, 1H), 3.43 (d, *J* = 17.0, 1H), 3.10 (s, 3H), 2.24 (s, 3H), 2.06-2.00 (m, 1H), 2.00-1.93 (m, 1H), 1.88 (d, *J* = 7.1, 3H), 1.73 (d, *J* = 7.1, 3H), 1.71 (s, 3H), 1.56 (d, *J* = 5.3, 1H), 0.91 (t, *J* = 7.4, 3H).

^{13}C NMR (176 MHz, $CDCl_3$): δ 200.6, 171.3, 171.0, 170.9, 169.3, 169.2, 167.6, 167.0, 160.0, 153.6, 150.4, 136.8, 130.5, 125.1, 123.2, 122.6, 116.2, 110.7, 106.3, 105.7, 99.3, 69.9, 55.4, 54.8, 52.7, 51.2, 45.5, 42.5, 37.6, 28.2, 23.3, 21.3, 20.8, 19.9, 11.5, 10.4.

NMR data of a (1:1) mixture of commercially available, isolated Zelkovamycin and synthesized Zelkovamycin:

^{1}H NMR (700 MHz, $CDCl_3$): δ 10.78 (s, 1H), 9.21 (s, 1H), 9.15 (d, *J* = 8.4 Hz, 1H), 8.68 (d, *J* = 7.5, 1H), 8.15 (s, 1H), 7.95 (s, 1H), 7.02 (t,

J = 7.9 Hz, 1H), 7.00 (d, J = 7.0, 1H), 6.94 (s, 1H), 6.78 (d, J = 8.1, 1H), 6.43 (d, J = 7.7, 1H), 5.73-5.68 (m, 1H), 5.52-5.46 (m, 1H), 5.24-5.18 (m, 1H), 5.18-5.14 (m, 1H), 5.02 (d, J = 17.1, 1H), 4.19 (dd, J = 15.7, 3.6, 1H), 4.11-4.06 (m, 1H), 3.88 (s, 3H), 3.62 (dd, J = 17.3, 7.4, 1H), 3.59-3.55 (m, 1H), 3.55 (d, J = 17.5, 1H), 3.11 (s, 3H), 2.24 (s, 3H), 2.04-2.00 (m, 1H), 1.99-1.94 (m, 1H), 1.88 (d, J = 7.0, 3H), 1.75 (d, J = 7.1, 3H), 1.70 (s, 3H), 1.58 (d, J = 5.4, 1H), 0.91 (t, J = 7.4, 3H).

^{13}C NMR (176 MHz, $CDCl_3$): δ 200.3, 171.7, 171.0, 170.6, 169.2, 169.1, 169.0, 167.1, 160.5, 153.5, 149.6, 136.7, 130.0, 124.9, 124.3, 122.8, 116.1, 111.4, 106.2, 105.7, 99.5, 69.9, 55.5, 55.0, 52.8, 50.7, 45.9, 42.4, 37.7, 28.2, 23.2, 21.3, 20.6, 19.9, 11.4, 10.3.

Zelkovamycin analogue

The Zelkovamycin analogue ΔMeO-Zelkovamycin (**14**) was synthesized *via* an analogues synthetic route.

ΔMeO-Zelkovamycin (14)

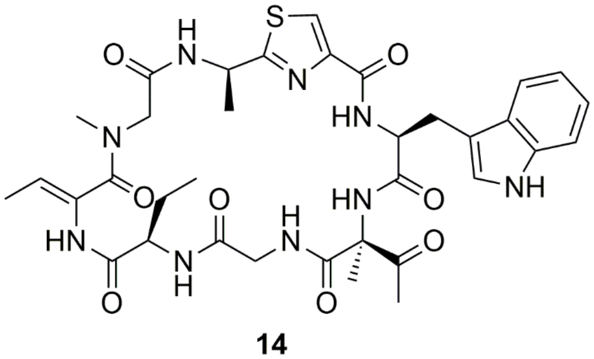

14

Yield: 4.1 mg

TLC (10% methanol in dichloromethane): R_f = 0.8,

(5% methanol in dichloromethane): R_f = 0.5.

LC-MS (ESI): *m/z* calcd for $C_{35}H_{44}N_9O_8S^+$ $[M+H]^+$ 750.3, found 750.3, calcd for $NaC_{35}H_{43}N_9O_8S^+$ $[M+Na]^+$ 772.3, found 772.3.

HRMS (ESI): *m/z* calcd for $C_{35}H_{44}N_9O_8S^+$ $[M+H]^+$ 750.3028, found 750.3034, calcd for $NaC_{35}H_{43}N_9O_8S^+$ $[M+Na]^+$ 772.2848, found 772.2846, calcd for $H(C_{35}H_{43}N_9O_8S)_2^+$ $[2M+H]^+$ 1499.5989, found 1499.5984.

$[\propto]_{\mathbf{D}}^{\mathbf{23}}$ ([c] = 1 in $CHCl_3$): +13.

^{1}H NMR (700 MHz, $CDCl_3$): δ 10.76 (s, 1H), 9.34 (s, 1H), 8.97 (d, *J* = 8.5, 1H), 8.54 (d, *J* = 7.5, 1H), 8.10 (s, 1H), 7.72 (s, 1H), 7.56 (d, *J* = 8.3, 1H), 7.20 (d, *J* = 7.8, 1H), 7.14 (t, *J* = 7.5, 1H), 7.12 (s, 1H), 7.12 (s, 1H), 7.10 (t, *J* = 7.1, 1H), 6.82 (d, *J* = 6.4, 1H), 5.53-5.46 (m, 1H), 5.26-5.21 (m, 1H), 5.23-5.18 (m, 1H), 5.16-5.11 (m, 1H), 5.02 (d, *J* = 17.1, 1H), 4.11-4.05 (m, 1H), 3.67-3.62 (m, 1H), 3.61-3.55 (m, 1H), 3.45 (d, *J* = 17.5, 2H), 3.12 (s, 3H), 2.23 (s, 3H), 2.05-1.99 (m, 1H), 1.90 (d, *J* = 7.0, 3H), 1.74 (d, *J* = 7.1, 3H), 1.67 (s, 3H), 1.62 (d, *J* = 5.3, 1H), 0.90 (t, *J* = 7.4, 3H).

^{13}C NMR (176 MHz, $CDCl_3$): δ 201.3, 171.1, 171.1, 171.0, 169.4, 167.9, 166.4, 163.5, 160.2, 158.9, 150.2, 135.2, 130.4, 126.0, 122.0, 120.3, 116.6, 112.2, 111.1, 106.2, 100.1, 69.8, 54.9, 52.5, 51.1, 45.7, 42.7, 37.7, 27.6, 23.3, 21.3, 20.8, 19.9, 11.5, 10.4.

Preparation of the standards for the Marfey's analysis

Marfey's Derivatization General Procedure

The synthesis of the different Marfey's derivatives was carried out according to a modified version of the instruction sheet 48895 by Thermo Scientific.

The corresponding amino acid (2.5 µmol) was placed in an Eppendorf tube and dissolved in H_2O (50 µl). Marfey's reagent (FDAA, 3.5 µmol) dissolved in acetone (100 µl) was added. The mixture was vortexed, $NaHCO_3$ (aq., 1 M, 20 µl) was added and the resulting mixture was vortexed again. The Eppendorf tube was placed in a pre-heated heat block and gently shaken for 1 h at 40 °C. The mixture was cooled down to room temperature, HCl (aq., 1M, 80 µl) was added and the resulting mixture was vortexed. The solvent was removed under high vacuum and the residue was taken up in a mixture of MeCN/H_2O (the ratio was adapted to ensure solubility) and filtered through a micro filter. The obtained sample was used without further purification for LC-MS analysis. If required, the solution was further diluted with MeCN/H2O (1:1) prior to LC-MS analysis.

Marf-(L)-Phe-OH

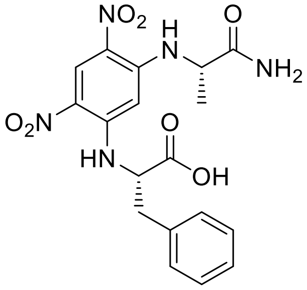

Marf-(L)-Phe-OH was synthesized following the *Marfey's Derivatization General Procedure*. The obtained product was analyzed by LC-MS and used for LC-MS Marfey's analysis without further purification.

LC-MS (ESI (G1)): t_R = 7.14 min (C_{18}); *m/z* = calcd for $C_{18}H_{20}N_5O_7^+$ $[M+H]^+$ 418.14, found 417.97, calcd for $H(C_{18}H_{19}N_5O_7)_2^+$ $[2M+H]^+$ 835.26, found 834.76, calcd for $Na(C_{18}H_{19}N_5O_7)_2^+$ $[2M+Na]^+$ 857.25, found 856.39.

Marf-(D)-Phe-OH

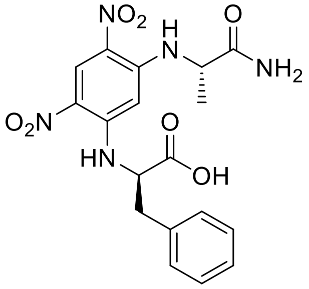

Marf-(D)-Phe-OH was synthesized following the *Marfey's Derivatization General Procedure*. The obtained product was analyzed by LC-MS and used for LC-MS Marfey's analysis without further purification.

LC-MS (ESI (G1)): t_R = 7.53 min (C_{18}); *m/z* = calcd for $C_{18}H_{20}N_5O_7^+$ $[M+H]^+$ 418.14, found 417.91, calcd for $H(C_{18}H_{19}N_5O_7)_2^+$ $[2M+H]^+$ 835.26, found 834.53, calcd for $Na(C_{18}H_{19}N_5O_7)_2^+$ $[2M+Na]^+$ 857.25, found 856.49.

Marf-Gly-OH

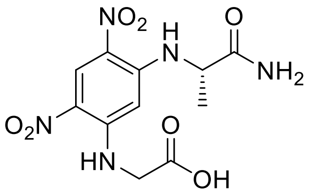

Marf-Gly-OH was synthesized following the *Marfey's Derivatization General Procedure*. The obtained product was analyzed by LC-MS and used for LC-MS Marfey's analysis without further purification.

LC-MS (ESI (G1)): t_R = 5.80 min (C18); *m/z* = calcd for $C_{11}H_{14}N_5O_7^+$ $[M+H]^+$ 328.09, found 327.98, calcd for $H(C_{11}H_{13}N_5O_7)_2^+$ $[2M+H]^+$ 655.17, found 654.57.

LC-MS (ESI (G2)): t_R = 31.51 min (C_{18}); *m/z* = calcd for $C_{11}H_{14}N_5O_7^+$ $[M+H]^+$ 328.09, found 328.86.

Marf-Sar-OH

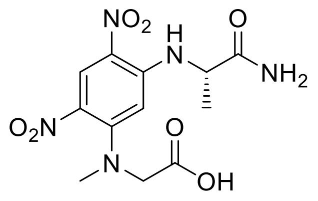

Marf-Sar-OH was synthesized following the *Marfey's Derivatization General Procedure*. The obtained product was analyzed by LC-MS and used for LC-MS Marfey’s analysis without further purification.

LC-MS (ESI (G1)): t_R = 5.72 min (C_{18}); *m/z* = calcd for $C_{12}H_{16}N_5O_7^+$ $[M+H]^+$ 342.10, found 341.96, calcd for $Na(C_{12}H_{15}N_5O_7)_2^+$ $[2M+Na]^+$ 705.18, found 704.53.

LC-MS (ESI (G2)): t_R = 31.39 min (C_{18}); *m/z* = calcd for $C_{12}H_{16}N_5O_7^+$ $[M+H]^+$ 342.10, found 342.98.

Marf-AlaThz-OH

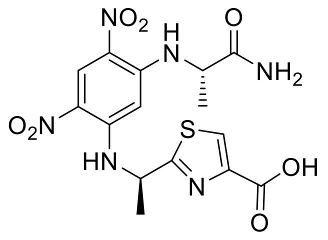

Marf-AlaThz-OH was synthesized following the *Marfey's Derivatization General Procedure*. The obtained product was analyzed by LC-MS and used for LC-MS Marfey’s analysis without further purification.

LC-MS (ESI (G1)): t_R = 6.57 min (C_{18}); *m/z* = calcd for $C_{15}H_{17}N_6O_7S^+$ $[M+H]^+$ 425.09, found 424.97, calcd for $H(C_{15}H_{16}N_6O_7S)_2^+$ $[2M+H]^+$ 849.17, found 848.38, calcd for $Na(C_{15}H_{16}N_6O_7S)_2^+$ $[2M+Na]^+$ 871.15, found 870.33.

Marf-(L)-Abu-OH

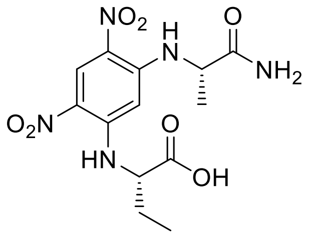

Marf-(L)-Abu-OH was synthesized following the *Marfey's Derivatization General Procedure*. The obtained product was analyzed by LC-MS and used for LC-MS Marfey's analysis without further purification.

LC-MS (ESI (G1)): t_R = 6.34 min (C_{18}); *m/z* = calcd for $C_{13}H_{18}N_5O_7^+$ $[M+H]^+$ 356.12, found 355.99, calcd for $H(C_{13}H_{17}N_5O_7)_2^+$ $[2M+H]^+$ 711.23, found 710.76, calcd for $Na(C_{13}H_{17}N_5O_7)_2^+$ $[2M+Na]^+$ 733.22, found 732.51.

LC-MS (ESI (G2)): t_R = 22.32 min (C_{18}); *m/z* = calcd for $C_{13}H_{18}N_5O_7^+$ $[M+H]^+$ 356.12, found 356.03, calcd for $H(C_{13}H_{17}N_5O_7)_2^+$ $[2M+H]^+$ 711.23, found 710.71, calcd for $Na(C_{13}H_{17}N_5O_7)_2^+$ $[2M+Na]^+$ 733.22, found 732.46.

Marf-(D)-Abu-OH

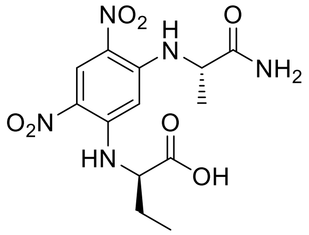

Marf-(D)-Abu-OH was synthesized following the *Marfey's Derivatization General Procedure*. The obtained product was analyzed by LC-MS and used for LC-MS Marfey's analysis without further purification.

LC-MS (ESI (G1)): t_R = 6.34 min (C_{18}); *m/z* = calcd for $C_{13}H_{18}N_5O_7^+$ $[M+H]^+$ 356.12, found 355.99, calcd for $H(C_{13}H_{17}N_5O_7)_2^+$ $[2M+H]^+$ 711.23, found 710.76, calcd for $Na(C_{13}H_{17}N_5O_7)_2^+$ $[2M+Na]^+$ 733.22, found 732.51.

LC-MS (ESI (G2)): t_R = 22.32 min (C_{18}); *m/z* = calcd for $C_{13}H_{18}N_5O_7^+$ $[M+H]^+$ 356.12, found 356.03, calcd for $H(C_{13}H_{17}N_5O_7)_2^+$ $[2M+H]^+$ 711.23, found 710.71, calcd for $Na(C_{13}H_{17}N_5O_7)_2^+$ $[2M+Na]^+$ 733.22, found 732.46.

Marf-(L)-(4-MeO)Trp-OH

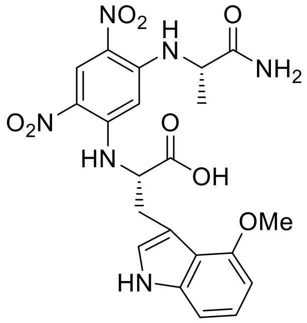

Marf-(L)-(4-MeO)Trp-OH was synthesized following the *Marfey's Derivatization General Procedure*. The obtained product was analyzed by LC-MS and used for LC-MS Marfey's analysis without further purification.

LC-MS (ESI (G1)): t_R = 7.08 min (C_{18}); *m/z* = calcd for $C_{21}H_{23}N_6O_8^+$ $[M+H]^+$ 487.16, found 486.95, calcd for $H(C_{21}H_{22}N_6O_8)_2^+$ $[2M+H]^+$ 973.31, found 972.49, calcd for $Na(C_{21}H_{22}N_6O_8)_2^+$ $[2M+Na]^+$ 995.29, found 994.34.

LC-MS (ESI (G2)): t_R = 28.42 min (C_{18}); *m/z* = calcd for $C_{21}H_{23}N_6O_8^+$ $[M+H]^+$ 487.16, found 486.91, calcd for $H(C_{21}H_{22}N_6O_8)_2^+$ $[2M+H]^+$ 973.31, found 972.40, calcd for $Na(C_{21}H_{22}N_6O_8)_2^+$ $[2M+Na]^+$ 995.29, found 994.33.

Marfey's analysis methodologies

HCl hydrolyzation of Zelkovamycin

The hydrolyzation of Zelkovamycin was carried out following a procedure by Eveleigh and Winter.[1]

To this end, isolated Zelkovamycin (1 mg) was treated with 6 N HCl (sequencing grade) in a sealed tube at 130 °C for 5 h. The obtained hydrolysate was analyzed as

unmodified lysate by LC-MS as well as subsequently converted into the corresponding Marfey's derivatives for further analytical investigations.

TsOH hydrolyzation of Zelkovamycin

A mild hydrolysis procedure utilizing TsOH was carried out to preserve the hydrolysis labile tryptophan residue of Zelkovamycin. To this end, an aliquot of Zelkovamycin was incubated with 3 N TsOH in the presence of 2% thioglycolic acid at 110 °C for 18 h according to the procedure developed by Liu and Chang to determine the presence of tryptophanes in proteins.[2] The obtained hydrolysate was analyzed as unmodified lysate by LC-MS as well as subsequently converted into the corresponding Marfey's derivatives for further analytical investigations.

LC-MS Setup for Marfey's Analysis

Due to the different intermolecular interactions of Marfey's derivatives containing a (D) - or a (L) -amino acid, it is possible to separate them on a non-chiral, reverse phase analytical HPLC column. Stronger intramolecular interactions between the (D)-amino acid 'residue' and the Marfey's reagent 'residue' reduce their polarity relative to the (L)-derivatives. Therefore, (D)-derivatives display longer retention times than the corresponding (L)-derivatives.

For analysis of the Marfey's amino acid derivatives, two different elution gradients were used. First, a short gradient program (denoted as 'G1') that was generaly used to measure the retention times of the different Marfey's derivatives in single- and coinjection experiments. Second, a longer control gradient program (denoted as 'G2') was used to verify the analysis for those cases in which the difference in the retention time between both isomers was only small. Accordingly, the following settings and gradients were used:

Solvent A: 0.1% formic acid in H_2O; Solvent B: 0.1% formic acid in MeCN, flow rate of 1 mL/min; ESI peak detection in the positive mode.

Gradient 1 (G1): 0 min / 10% B → 1 min / 10% B → 10 min / 100% B → 12 min 100% B → 12.1 min 10% B → 15 min 10% B.

Gradient 2 (G2): 0 min / 5% B → 35 min / 50% B → 36 min / 100% B → 40 min 100% B.

Biochemical and biological materials and methods

Cell culture

HeLa cells were cultured (37 °C, 5% CO_2) using Dulbecco's Modified Eagle's Medium (DMEM, Gibco®, 31966-047) supplemented with 10% fetal bovine serum (Gibco®, 16140-071) and 1% Penicillin/Streptomycin (Gibco®, 15140-122) to a maximum of 25 passages. SH-SY5Y cells were cultured in DMEM/F12 (Gibco®, 31330-038) supplemented with 10% fetal bovine serum and 1% Penicillin/Streptomycin to a maximum of 15 passages. WM3734 cells were cultured in Tu2% (400 mL MCDB153 basal medium, 100 mL L15 Leibowitz, substituted with 250 µL human insulin, 560 µL [1.5 M] $CaCl_2$, 10 mL FBS, and 5 mL [200 mM] L-glutamine) at 37 °C in 5% CO_2 as described.[3] MeWo cells were cultured in 10% FBS- and 1% L-glutamine [200 mM]-substituted RPMI medium. For all further assays, cells were seeded in indicated well plates at least 16 h before treatment and grown to approximately 80% confluency. Prior to compound treatment cells were washed with Phosphate-buffered saline (PBS, Gibco®, 10010023).

Cell viability assays

HeLa (1.4 x 10^4 cells/well), SH-SY5Y (1.6 x 10^4 cells/well), WM3734 and MeWo cells (both 2.0 X 10^4 cells/well) were cultured in 96 well microtiter plates (Sarstedt 83.3924) as described above. Subsequently, cells were treated with indicated compounds. After 48 h cell viability was determined by an MTT assay. To this end, 25 µL of the MTT reagent mixture (5 mg/ml in PBS, Thiazolyl Blue Tetrazolium Bromide, SigmaAldrich, M2128) was added directly into the medium. After incubation (3 h, 37 °C and 5% CO_2), the resultant formazan crystals were dissolved in 100 µL extraction buffer (20% SDS in 1:1 Dimethylformamide/Water, pH = 4.7, 4 h at 37 °C). For quantification of cell viability, absorbance at 570 nm was measured by using a microplate reader (Spark 10M, Tecan). All experiments were performed at least in triplicates and the relative cell viability was expressed as percentage relative to the DMSO-treated control cells. For IC_{50} determination normalized cell viability was fitted using GraphPad®. Nonlin fit: log(inhibitor) vs. response -- Variable slope (four parameters).

Quantification of extracellular acidification

HeLa cells (1.4 x 10^4 cells/well) were cultured in 96 well microtiter plates as described above. The cells were then treated with indicated compounds for 48 h. Next, absorbance of phenol red was measured by a microplate reader at 550 nm (blank reference 425 nm) and the corresponding ratio of signal intensities 550/425 nm to determine medium acidification was calculated. Statistical significance was determined using GraphPad® student's t-test of at least three replicates.

Lactate assay

(L)-Lactate concentrations in the culture medium were determined using an (L)-Lactate assay kit (KA3776 Version 02, Abnova). Culturing and compound treatment of HeLa and SH-SY5Y cells (0.1 x 106 cells/well) in 12-well microplates was performed as described above. Cells were then treated with indicated compounds. After 8, 24, 32 and 48 h of incubation, 100 µL of the culture media were sampled and diluted 100× with water to avoid metabolite interference. The subsequent measurements were performed according to the manufacturer's instructions. The resulting fluorescence (Ex530/Em585) was determined with a microplate reader. Statistical significance was evaluated using GraphPad® student's t-test of at least three replicates.

Pyruvate dehydrogenase activity assay

Activity of Pyruvate Dehydrogenase (PDH) was determined using a PDH activity assay kit (MK183, SIGMA-ALDRICH). To this end, SH-SY5Y cells (0.3 x 10^6 cells/well) were cultured in 6-well microplates as described above. After 4, 16, and 24 h, cells were collected by a cell scraper in PBS (+ 1× Protease-Inhibitor, Sigma-Aldrich, MSSAFE-1VL) and disrupted by sonication (Diagenode, conditions: 1 min pulse/30 sec pause, 5-7 cycles with high power). The subsequent steps and PDH activity calculation were then performed according to the manufacturer's instructions. Absorbance at 450 nm reflecting PDH activity was measured over a time period of 40 min using a microplate reader. The initial time point ($T_{initial}$) was set to 5 min whereas the final time point (T_{final}) was set to 40 min. Statistical significance of the results was evaluated using GraphPad® student's t-test of at least three replicates.

MS-based proteomics

Culture conditions

HeLa cells were cultured in 12-well microplates (0.1×10^6 cells/well) as described above. Subsequently, cells were treated with 20 µM Zelkovamycin or DMSO for 16 h. The cells were then collected by a cell scraper in PBS (+ 1× Protease-Inhibitor, Sigma-Aldrich, MSSAFE-1VL) and disrupted by sonication (Diagenode, conditions: 1 min pulse/30 sec pause, 5-7 cycles with high power). The resulting protein concentration was measured by a Bradford assay (Roth®, Roti®-Quant). An equivalent of 15 µg protein was precipitated by 4× volumes of acetone and submitted to LC MS/MS sample preparation.

LC-MS/MS settings

Experiments were performed on an Orbitrap Elite instrument (Thermo)[4] that was coupled to an EASY-nLC 1000 liquid chromatography (LC) system (Thermo). The LC was operated in the one-column mode. The analytical column was a fused silica capillary (75 µm × 37 cm) with an integrated PicoFrit emitter (New Objective) packed in-house with Reprosil-Pur 120 C18-AQ 1.9 µm resin (Dr. Maisch). The analytical column was encased by a column oven (Sonation) and attached to a nanospray flex ion source (Thermo). The column oven temperature was adjusted to 45 °C during data acquisition. The LC was equipped with two mobile phases: solvent A (0.1% formic acid, FA, in water) and solvent B (0.1% FA in acetonitrile, ACN). All solvents were of UPLC grade (Sigma). Peptides were directly loaded onto the analytical column with a maximum flow rate that would not exceed the set pressure limit of 980 bar (usually around 0.6 – 1.0 µL min^{-1}). Peptides were subsequently separated on the analytical column by running a 140 min gradient of solvent A and solvent B (start with 7% B; gradient 7% to 35% B for 120 min; gradient 35% to 80% B for 10 min and 80% B for 10 min) at a flow rate of 300 nL min^{-1}. The mass spectrometer was operated using Xcalibur software (version 2.2 SP1.48). The mass spectrometer was set in the positive ion mode. Precursor ion scanning was performed in the Orbitrap analyzer (FTMS; Fourier Transform Mass Spectrometry) in the scan range of *m/z* 300-1800 and at a resolution of 60000 with the internal lock mass option turned on (lock mass was 445.120025 *m/z*, polysiloxane; ref. [5]). Product ion spectra were recorded in a data

dependent fashion in the ion trap (ITMS) in a variable scan range and at a rapid scan rate. The ionization potential (spray voltage) was set to 1.8 kV. Peptides were analyzed using a repeating cycle consisting of a full precursor ion scan (3.0×10^6 ions or 50 ms) followed by 15 product ion scans (1.0×10^4 ions or 50 ms) where peptides are isolated based on their intensity in the full survey scan (threshold of 500 counts) for tandem mass spectrum (MS^2) generation that permits peptide sequencing and identification. Collision induced dissociation (CID) energy was set to 35% for the generation of MS^2 spectra. During MS^2 data acquisition dynamic ion exclusion was set to 120 seconds with a maximum list of excluded ions consisting of 500 members and a repeat count of one. Ion injection time prediction, preview mode for the FTMS, monoisotopic precursor selection and charge state screening were enabled. Only charge states higher than 1 were considered for fragmentation.

Peptide and Protein Identification using MaxQuant

RAW spectra were submitted to an Andromeda[6] search in MaxQuant (1.5.3.30) using the default settings.[7] Label-free quantification and match-between-runs was activated.[8] The MS/MS spectra data were searched against the Uniprot human reference database (UP000005640_9606.fasta, 70244 entries, downloaded 3/1/2016). All searches included a contaminants database search (as implemented in MaxQuant, 245 entries). The contaminants database contains known MS contaminants and was included to estimate the level of contamination. Andromeda searches allowed oxidation of methionine residues (16 Da) and acetylation of the protein N-terminus (42 Da) as dynamic modifications and the static modification of cysteine (57 Da, alkylation with iodoacetamide). Enzyme specificity was set to “Trypsin/P” with two missed cleavages allowed. The instrument type in Andromeda searches was set to Orbitrap and the precursor mass tolerance was set to ±20 ppm (first search) and ±4.5 ppm (main search). The MS/MS match tolerance was set to ±0.5 Da. The peptide spectrum match FDR and the protein FDR were set to 0.01 (based on target-decoy approach). Minimum peptide length was 7 amino acids. For protein quantification unique and razor peptides were allowed. Modified peptides were allowed for quantification. The minimum score for modified peptides was 40. Label-free protein quantification was switched on, and unique and razor peptides were considered for quantification with a minimum ratio count of 2. Retention times were recalibrated based

on the built-in nonlinear time-rescaling algorithm. MS/MS identifications were transferred between LC-MS/MS runs with the “match between runs” option in which the maximal match time window was set to 0.7 min and the alignment time window set to 20 min. The quantification is based on the “value at maximum” of the extracted ion current. At least two quantitation events were required for a quantifiable protein. Further analysis and filtering of the results was done in Perseus v1.5.2.6.[9] For quantification we combined related biological replicates to categorical groups and investigated only those proteins that were found in at least one categorical group in a minimum of 5 out of 6 biological replicates. Missing values were imputed (width 0.3, down shift 1.8). Comparison of protein group quantities (relative quantification) between different MS runs is based solely on the LFQ’s as calculated by MaxQuant (MaxLFQ algorithm; ref [8]).

Data availability

The mass spectrometry proteomics data have been deposited to the ProteomeXchange Consortium via the PRIDE[10] partner repository (https://www.ebi.ac.uk/pride/archive/) with the dataset identifier PXD014554. During the review process the data can be accessed via a reviewer account (Username: reviewer36060@ebi.ac.uk; Password: KaNC4Zbe)

Functional real-time metabolic analyses

To functionally and physiologically analyze the effect of the drug on mitochondrial metabolism, real time metabolic analysis of the oxygen consumption rate (OCR) and extracellular acidification rate (ECAR) was performed on a SeaHorse XFe96 extracellular flux analyzer (Agilent Technologies). In brief, cell number optimization experiments were done to determine appropriate cell densities as required for our experimental conditions. Based on this, 15,000 WM3734 cells, 15,000 HeLa cells or 50,000 SH-SY5Y cells were seeded per well of a XFe96 V3 PS tissue culture microplate one day prior to the experiment and incubated overnight at 37 °C in 5% CO_2. For metabolic analysis, cell culture medium was replaced by Agilent Seahorse XF Base Medium supplemented with iso-osmolar concentrations of glucose, sodium pyruvate, and glutamine 1 h before assay read-out. At least 4-6 replicates (wells) were used per concentration or experimental condition. During the real time analysis of our

specifically design assay, 3 baseline measurements were followed by the application of the drugs Zelkovamycin (**1**), Argyrin B, ΔMeO-Zelkovamycin (**14**) or reduced-Zelkovamycin (**13**) and direct effect on mitochondrial metabolism was documented over 18 measurements (≈108 min.). Finally, application of FCCP was performed to determine the maximal respiratory capacity of the cells (RRC) after incubation with the mentioned drugs and their effect on mitochondrial metabolism was analyzed by 3 measurements. For data normalization to the actual cell numbers per well, cells were fixed overnight with 4% PFA in PBS and stained with DAPI. Fluorescence was measured on a Tecan Spark microplate reader. Data analysis and normalization to DAPI fluorescence was performed using the Seahorse XF Mito Fuel Flex Test Report Generator in the Wave Software (Agilent Technolgies, Version 2.4.0.60).

Phosphofructokinase activity assay

HeLa cells (0.3 x 10^6 cells/well) were cultured in 6-well microplates as described above. Cells were then treated with indicated compounds for 24 h. The cells were collected by a cell scraper in PBS (+ 1× Protease-Inhibitor, Sigma-Aldrich, MSSAFE-1VL) and disrupted by sonication (Diagenode, conditions: 1 min pulse/30 sec pause, 5-7 cycles with high power). The activity of Phosphofructokinase (PFK) was measured by an Activity Colorimetric Assay Kit (Sigma-Aldrich, MAK093) following the manufacturer’s instructions. The resulting PFK activity was normalized to DMSO. Statistical significance was evaluated using GraphPad® student’s t-test of at least three replicates.

Supporting References

[1] J. W. Eveleigh, G. D. Winter, in *Protein sequence determination: A sourcebook of methods and techniques* (Ed.: S. B. Needleman), Springer Berlin Heidelberg, Berlin, Heidelberg, 1970, pp. 91-123.

[2] T.-Y. Liu, Y. H. Chang, Hydrolysis of proteins with p-toluenesulfonic acid. *J. Biol. Chem.* **1971**, *246*, 2842-2848.

[3] K. Satyamoorthy, E. DeJesus, A.J. Linnenbach, B. Krai, D.L. Kornreich, S. Rendle, D.E. Elder, M. Herlyn, Melanoma cell lines from different stages of progression and their biological and molecular analyses. *Melanoma Res.* **1997**, *7*, S35-42.

[4] A. Michalski, E. Damoc, O. Lange, E. Denisov, D. Nolting, M. Müller, R. Viner, J. Schwartz, P. Remes, M. Belford, J.-J. Dunyach, J. Cox, S. Horning, M. Mann, A. Makarov, Ultra high resolution linear ion trap Orbitrap mass spectrometer (Orbitrap Elite) facilitates top down LC MS/MS and versatile peptide fragmentation modes. *Mol. Cell. Proteomics* **2012**, *11*, O111.013698.

[5] J. V. Olsen, L. M. de Godoy, G. Li, B. Macek, P. Mortensen, R. Pesch, A. Makarov, O. Lange, S. Horning, M. Mann, Parts per million mass accuracy on an Orbitrap mass spectrometer via lock mass injection into a C-trap. *Mol. Cell. Proteomics* **2005**, *4*, 2010-2021.

[6] J. Cox, N. Neuhauser. A. Michalski, R. A. Scheltema, J. V. Olsen, M. Mann, Andromeda: a peptide search engine integrated into the MaxQuant environment. *J. Proteome Res.* **2011**, *10*, 1794-1805.

[7] J. Cox, M. Mann M, MaxQuant enables high peptide identification rates, individualized p.p.b.-range mass accuracies and proteome-wide protein quantification. *Nat. Biotechnol.* **2008**, *26*, 1367-1372.

[8] J. Cox, M. Y. Hein, C. A. Luber, I. Paron, N. Nagaraj, M. Mann, Accurate Proteome-wide Label-free Quantification by Delayed Normalization and Maximal Peptide Ratio Extraction, Termed MaxLFQ. *Mol. Cell. Proteomics* **2014**, *13*, 2513-2526.

[9] S. Tyanova, T. Temu, P. Sinitcyn, A. Carlson, M. Y. Hein, T. Geiger, M. Mann, J. Cox, The Perseus computational platform for comprehensive analysis of (prote)omics data. *Nat. Methods* **2016**, *13*, 731-740.

[10] J. A. Vizcaíno, A. Csordas, N. del-Toro, J. A. Dianes, J. Griss, I. Lavidas, G. Mayer, Y. Perez-Riverol, F. Reisinger, T. Ternent, Q. W. Xu, R. Wang, H. Hermjakob, 2016 update of the PRIDE database and related tools. *Nucleic Acids Res.* **2016**, *44*, D447-D456.

Spectra

^{1}H NMR of **SI-2** in $CDCl_3$

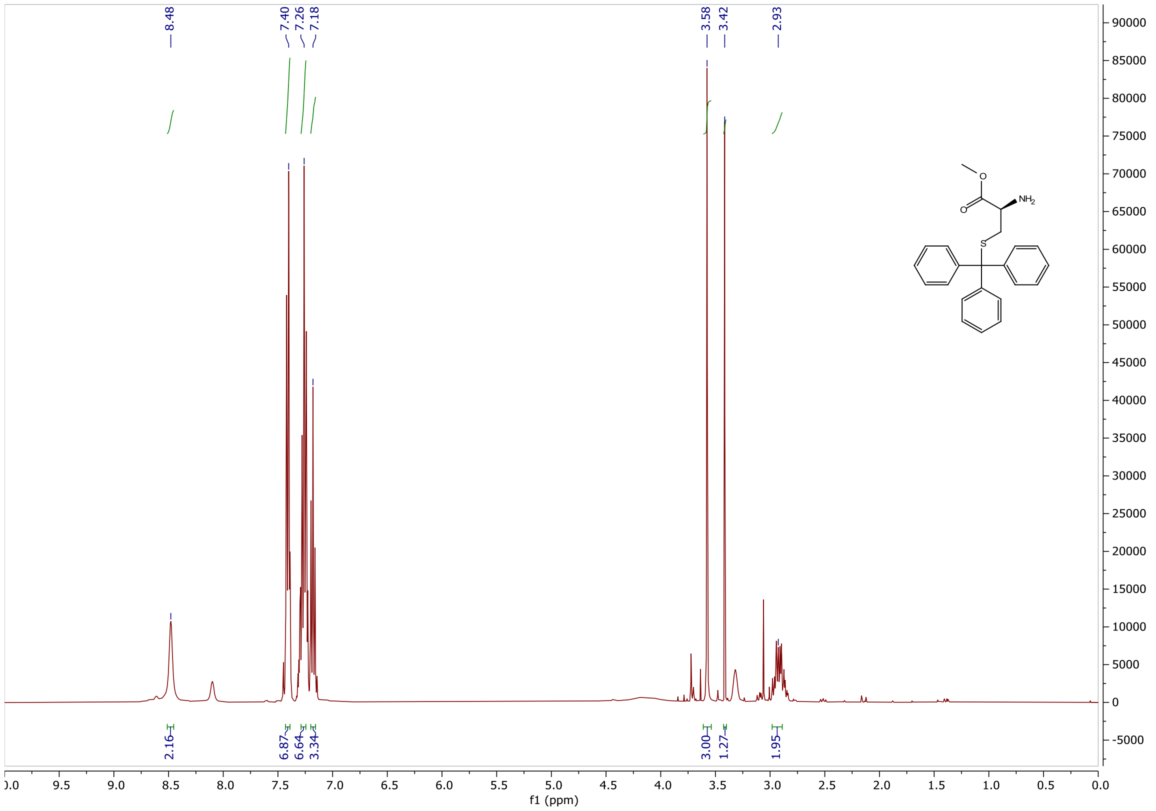

^{13}C NMR of **SI-2** in $CDCl_3$

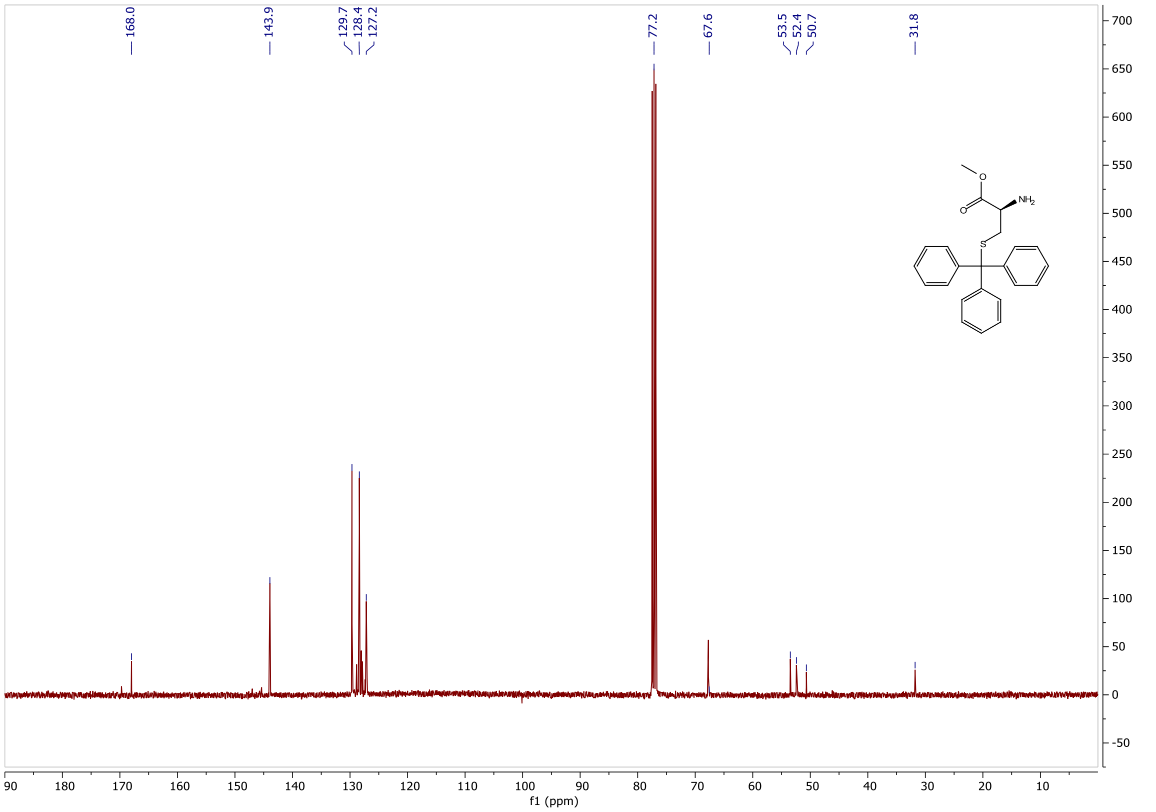

^{1}H NMR of **SI-3** in $CDCl_3$

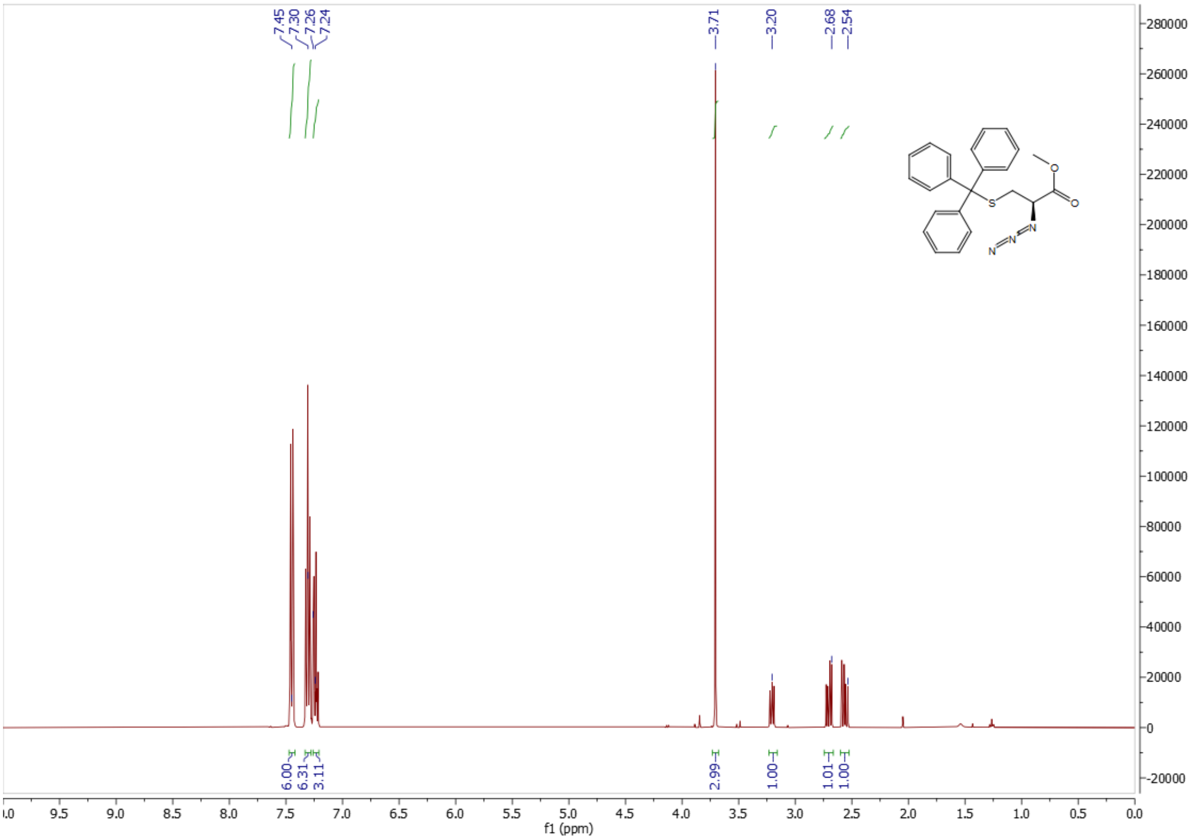

^{13}C NMR of **SI-3** in $CDCl_3$

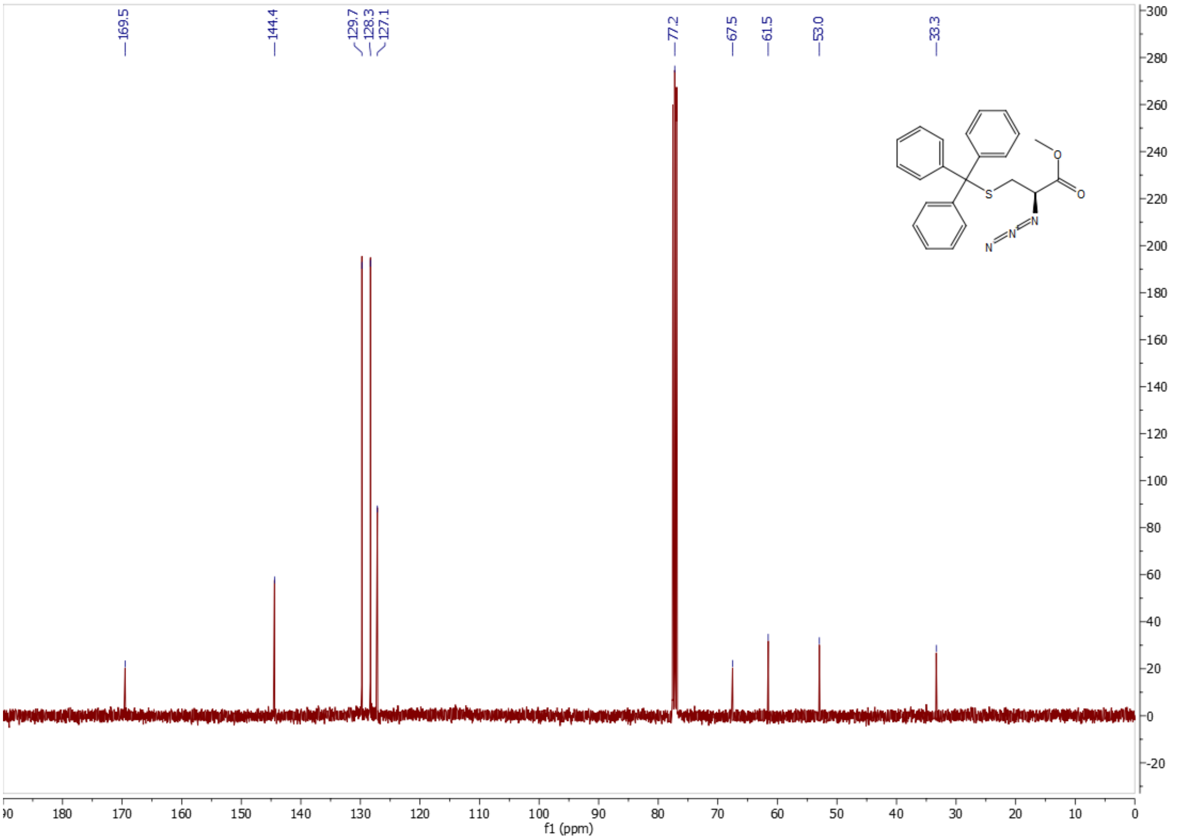

^{1}H NMR of **SI-4** in $CDCl_3$

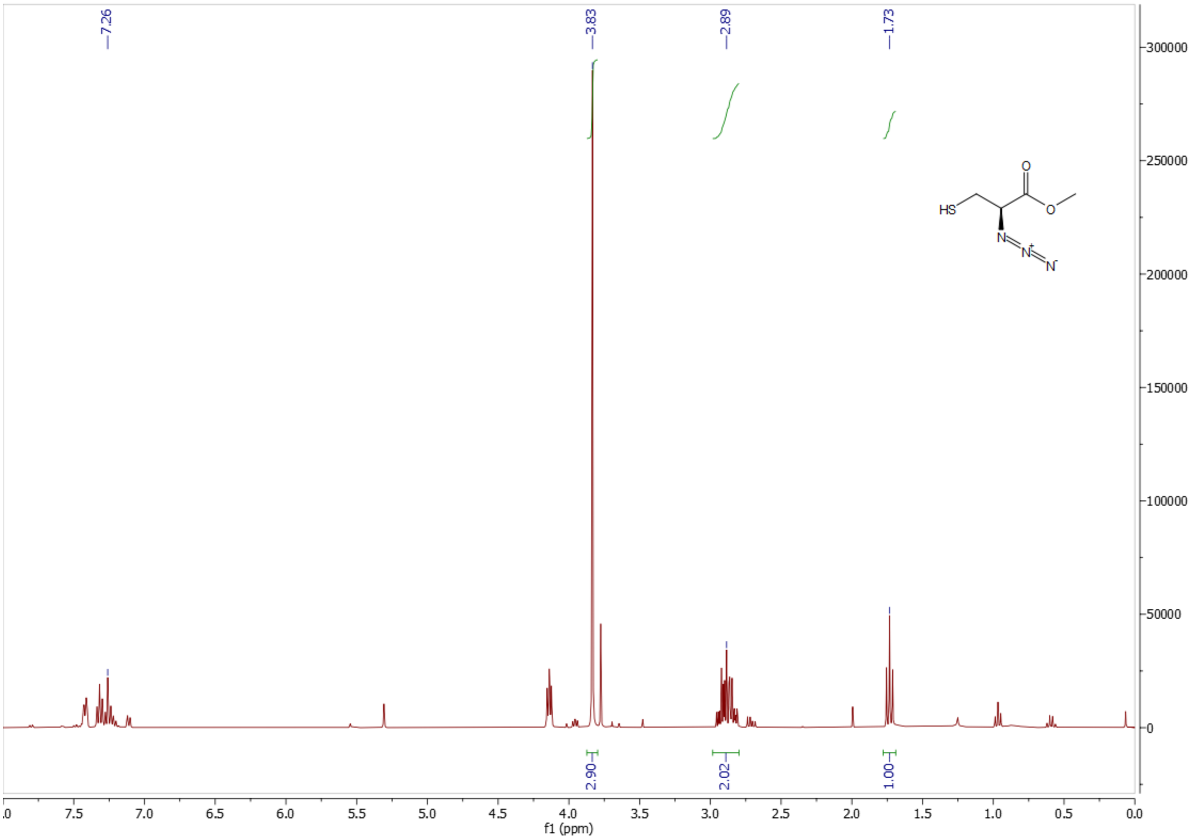

^{13}C NMR of **SI-4** in $CDCl_3$

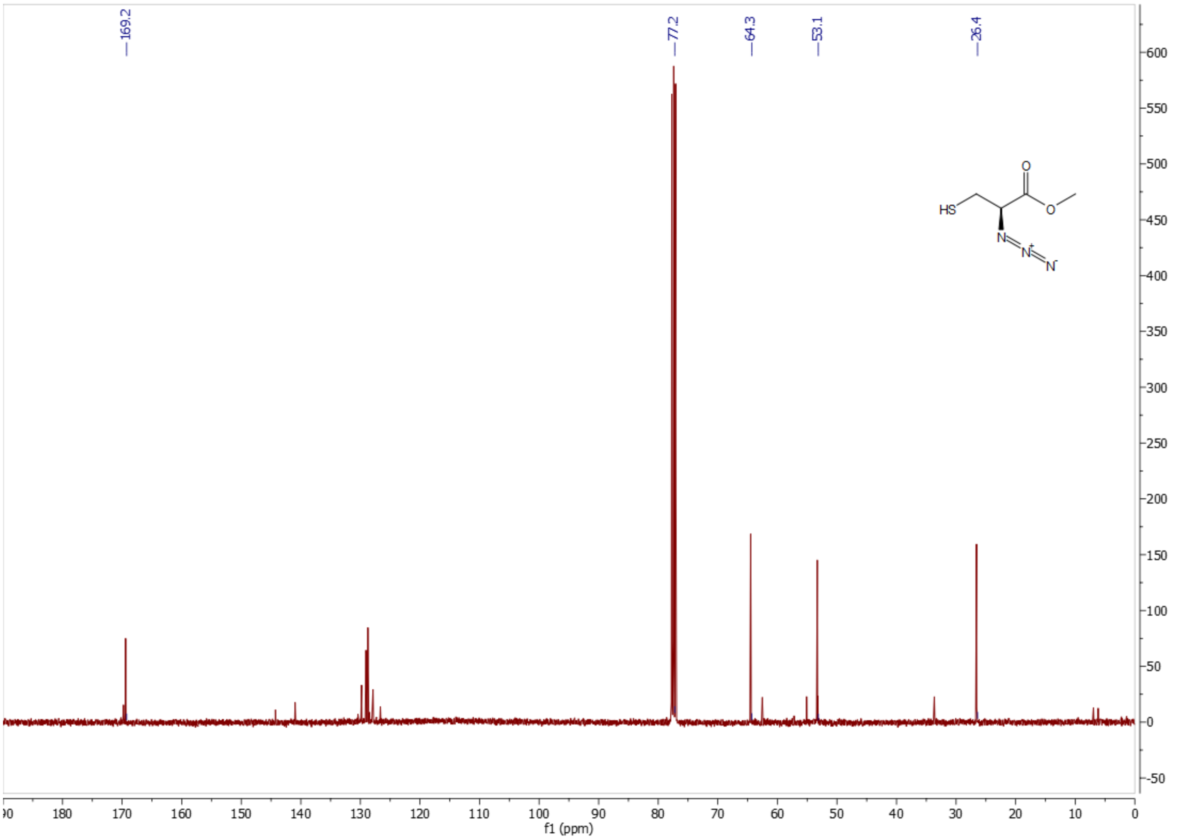

^{1}H NMR of **SI-5** in $CDCl_3$

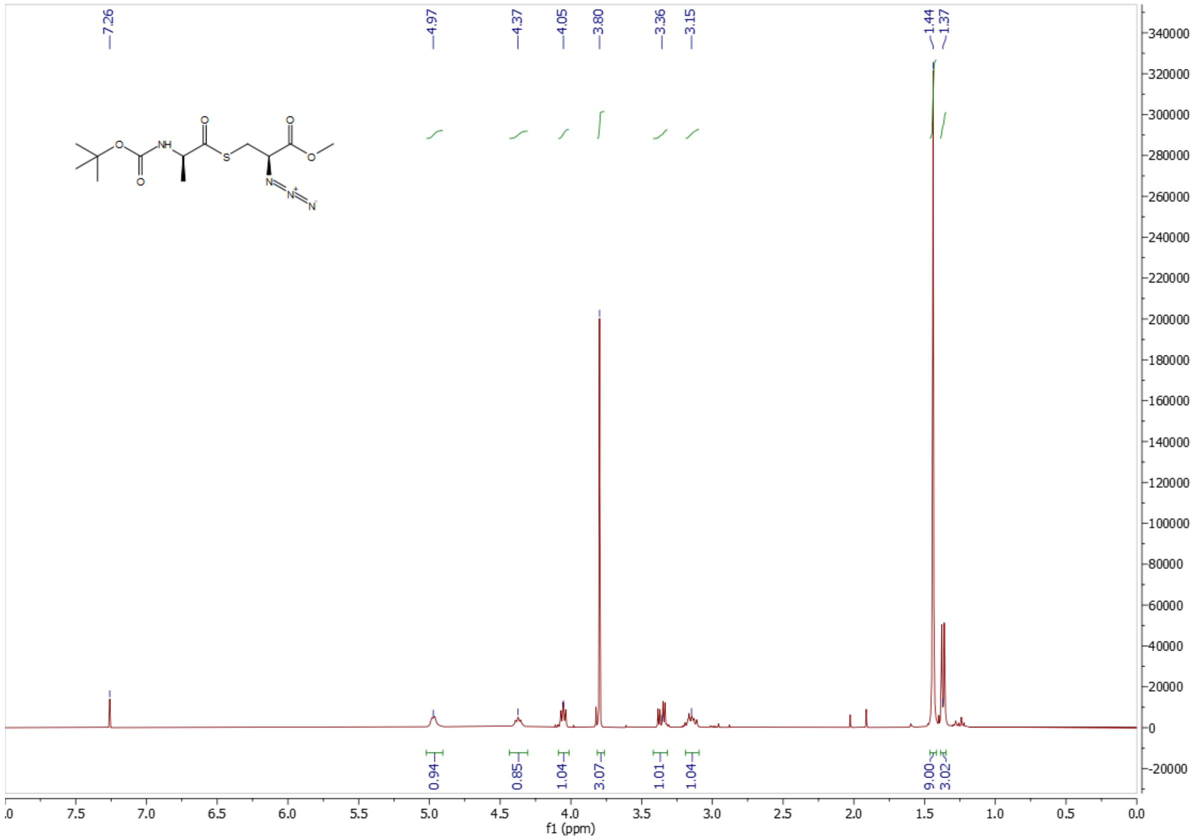

^{13}C NMR of **SI-5** in $CDCl_3$

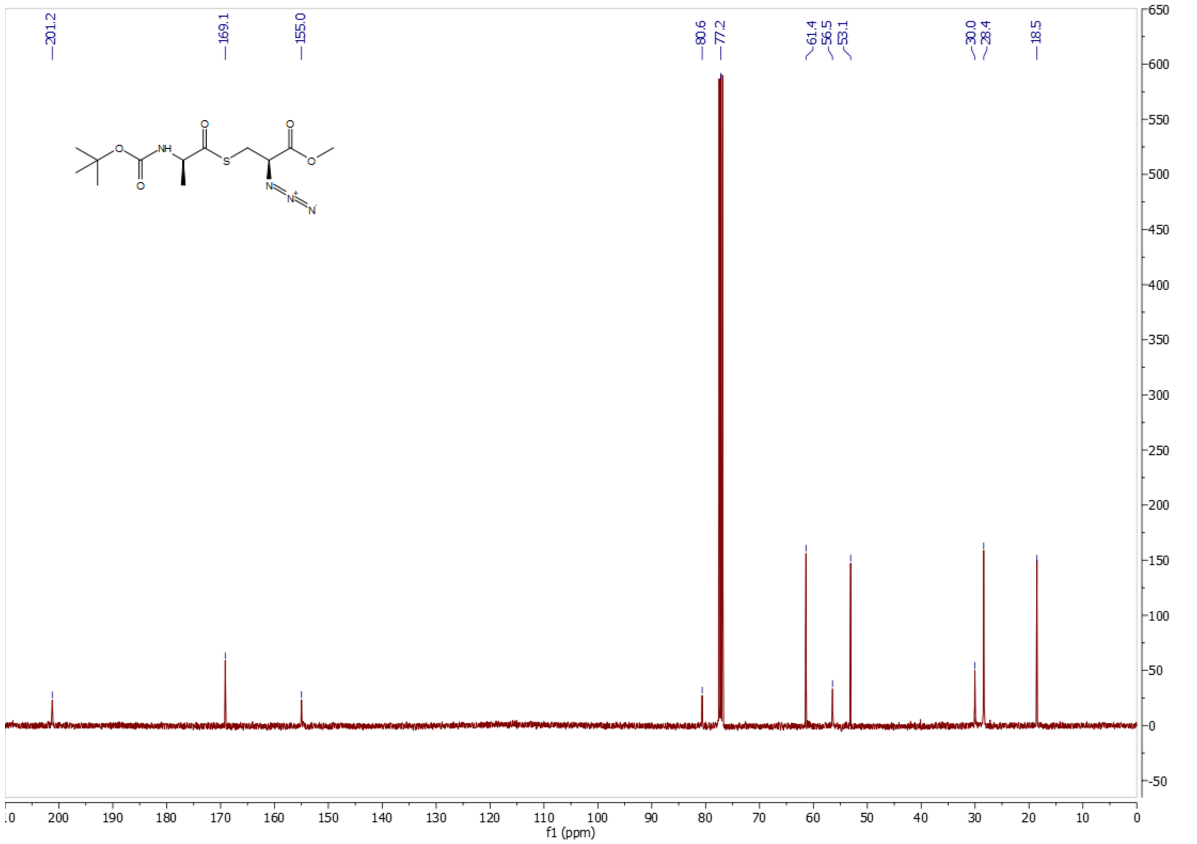

^{1}H NMR of **SI-6** in $CDCl_3$

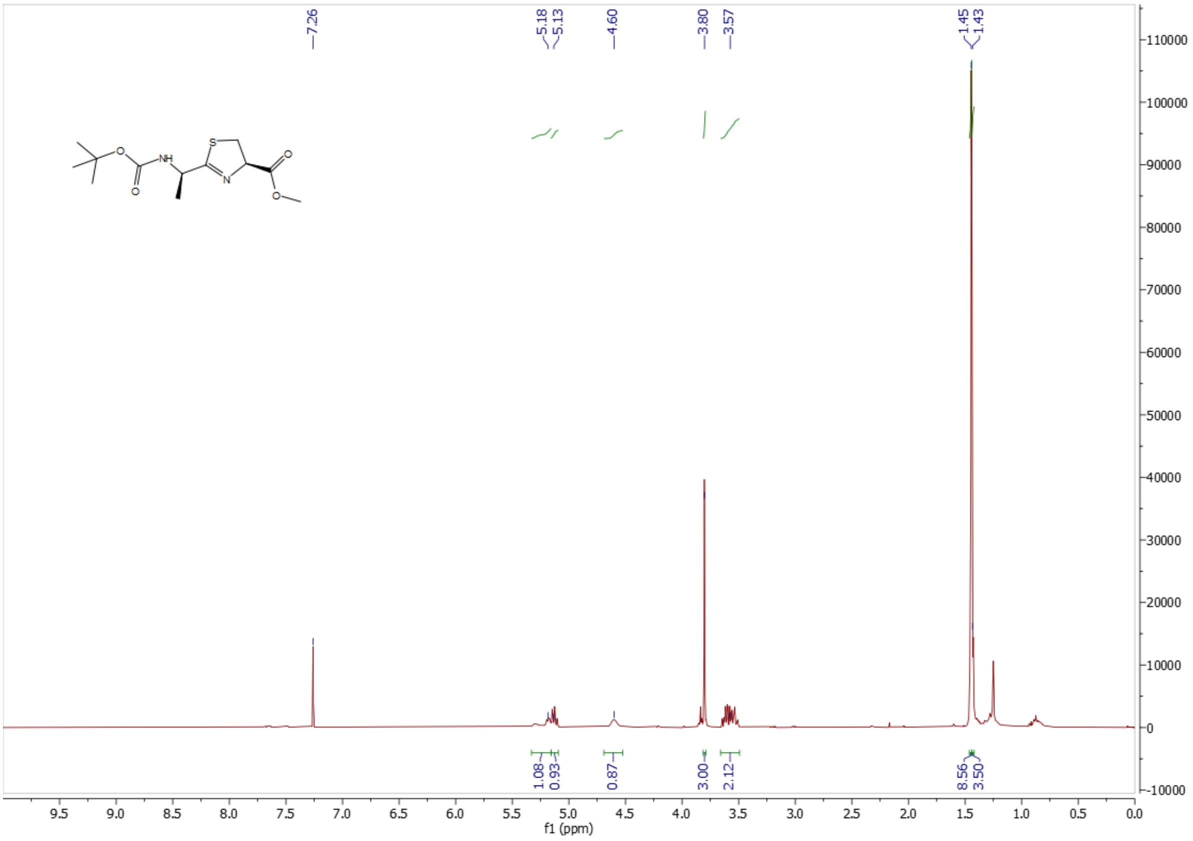

^{13}C NMR of **SI-6** in $CDCl_3$

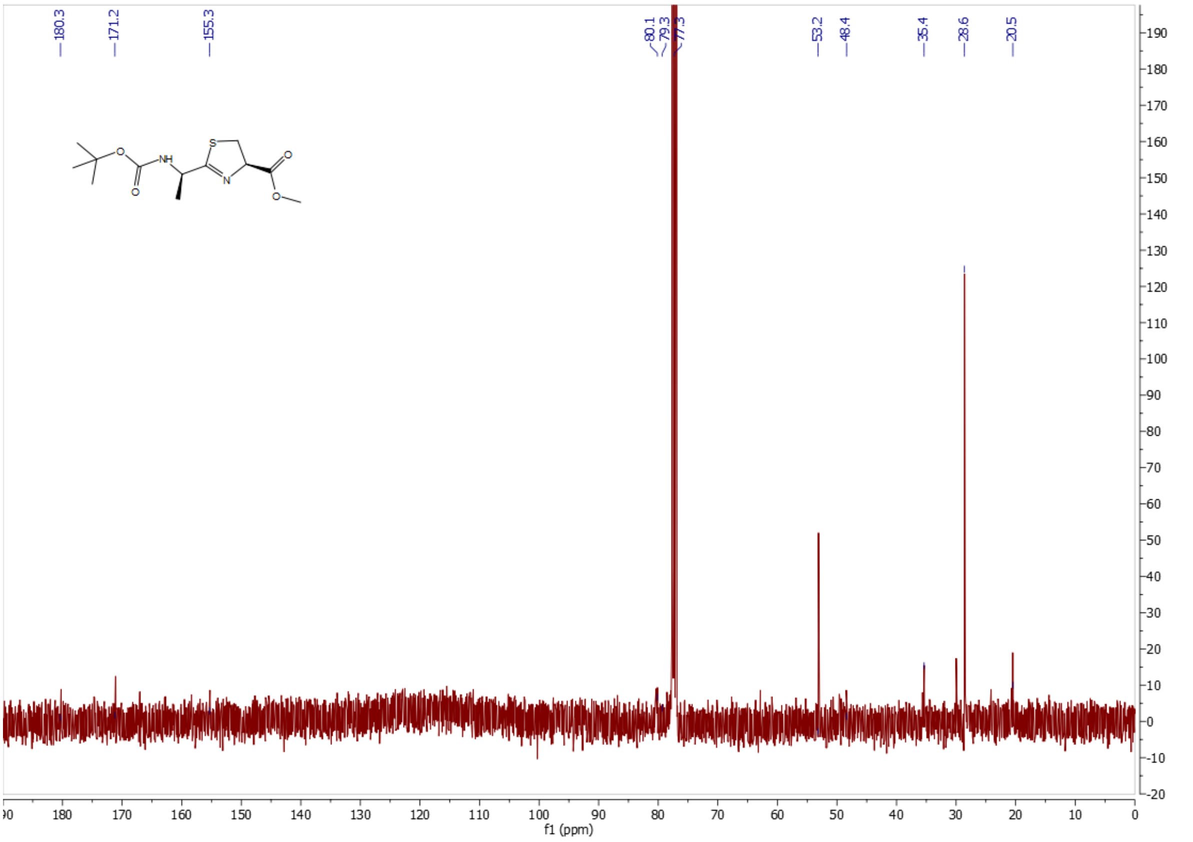

^{1}H NMR of **2** in $CDCl_3$

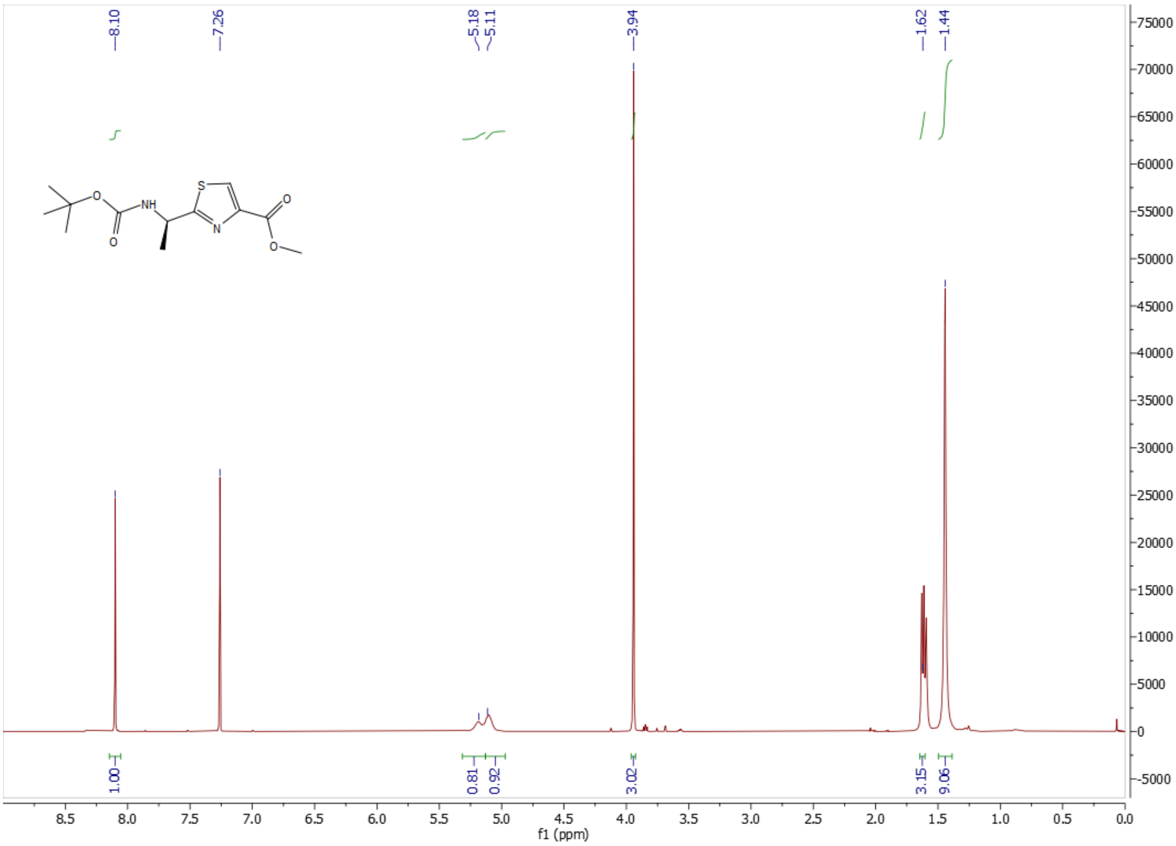

^{13}C NMR of **2** in $CDCl_3$

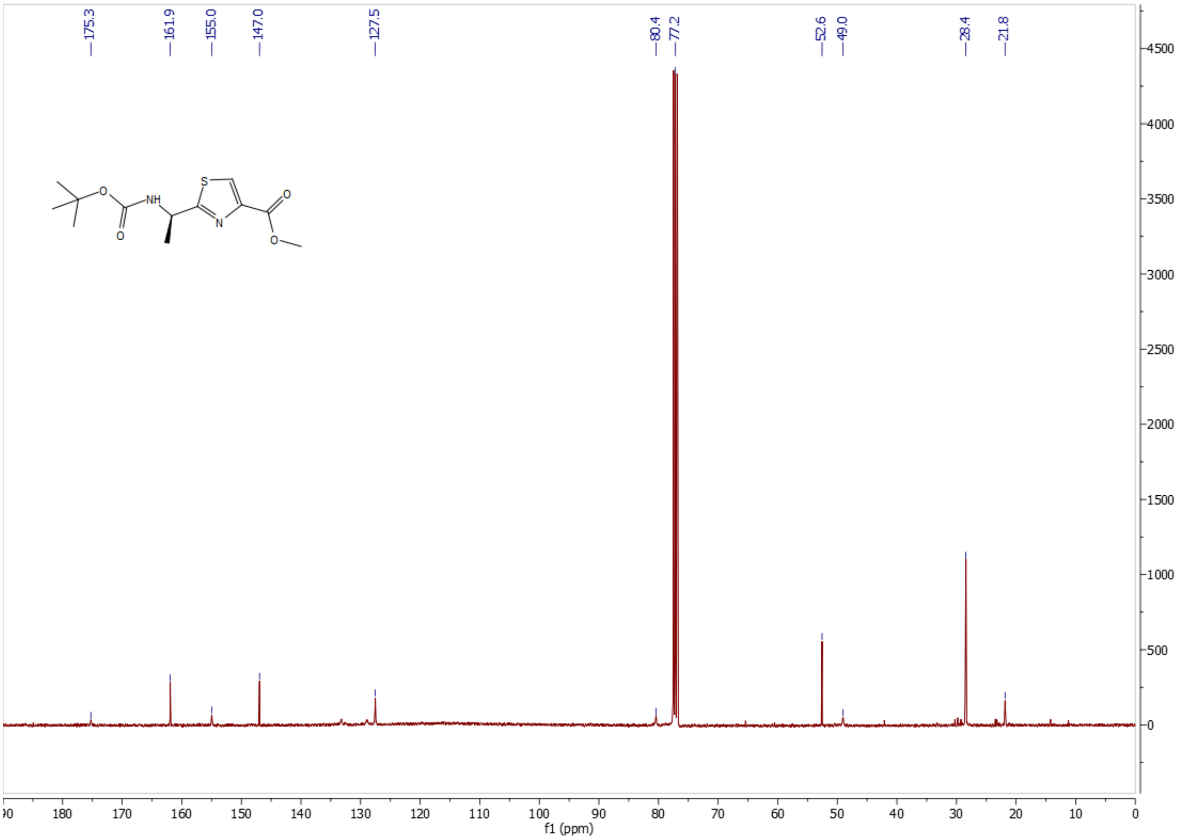

^{1}H NMR of **SI-7** in MeOD

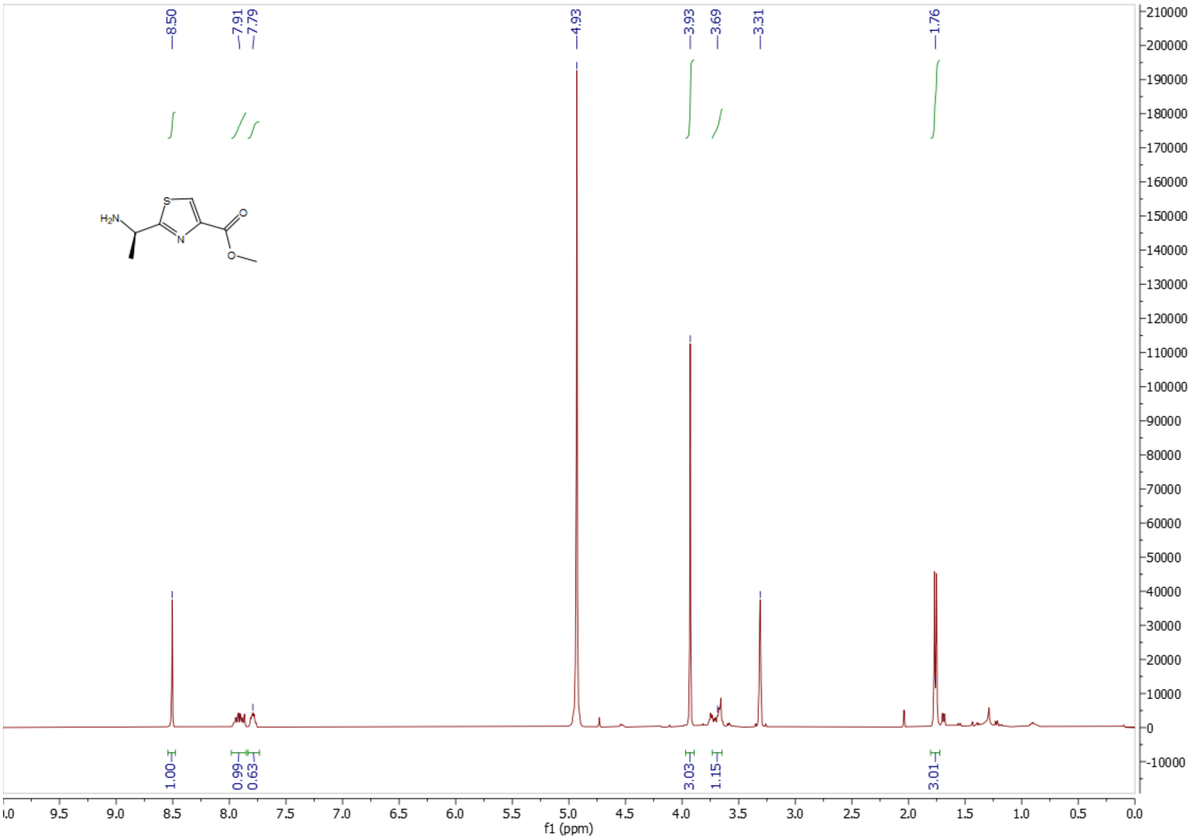

^{13}C NMR of **SI-7** in MeOD

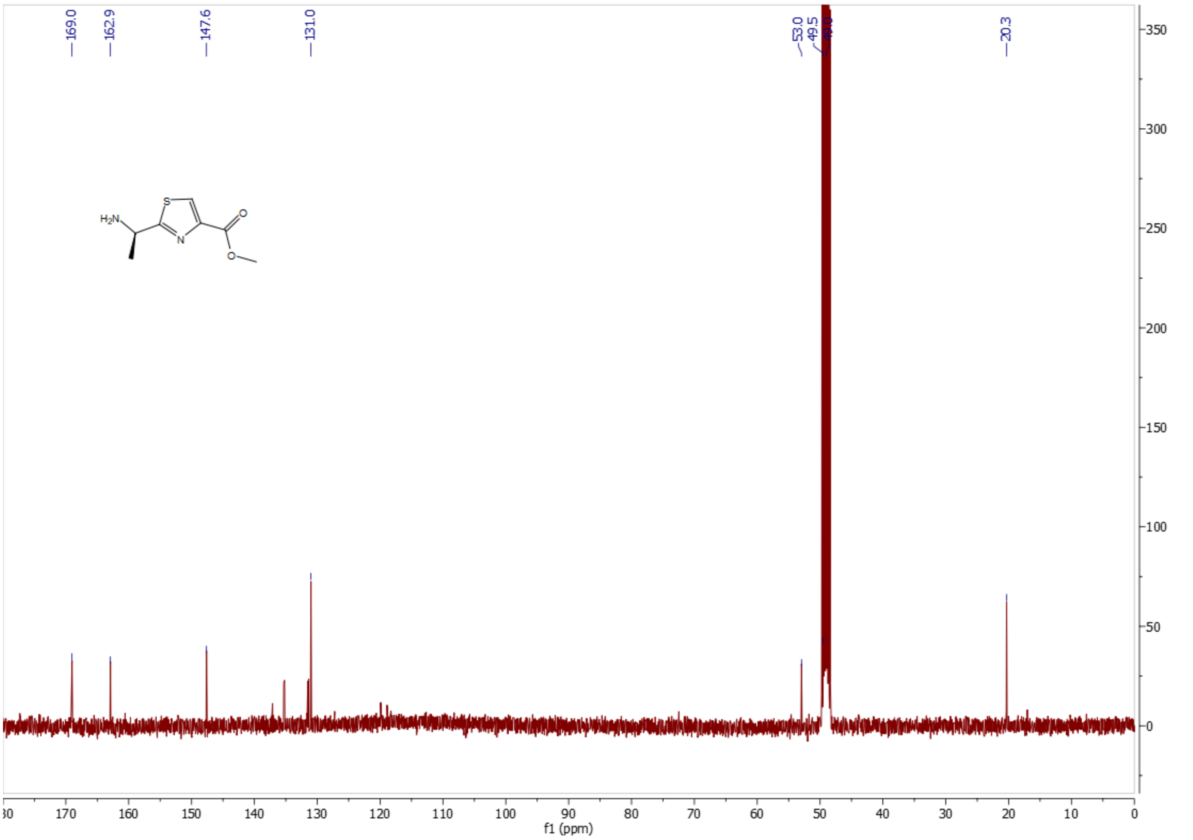

^{1}H NMR of **SI-8** in $CDCl_3$

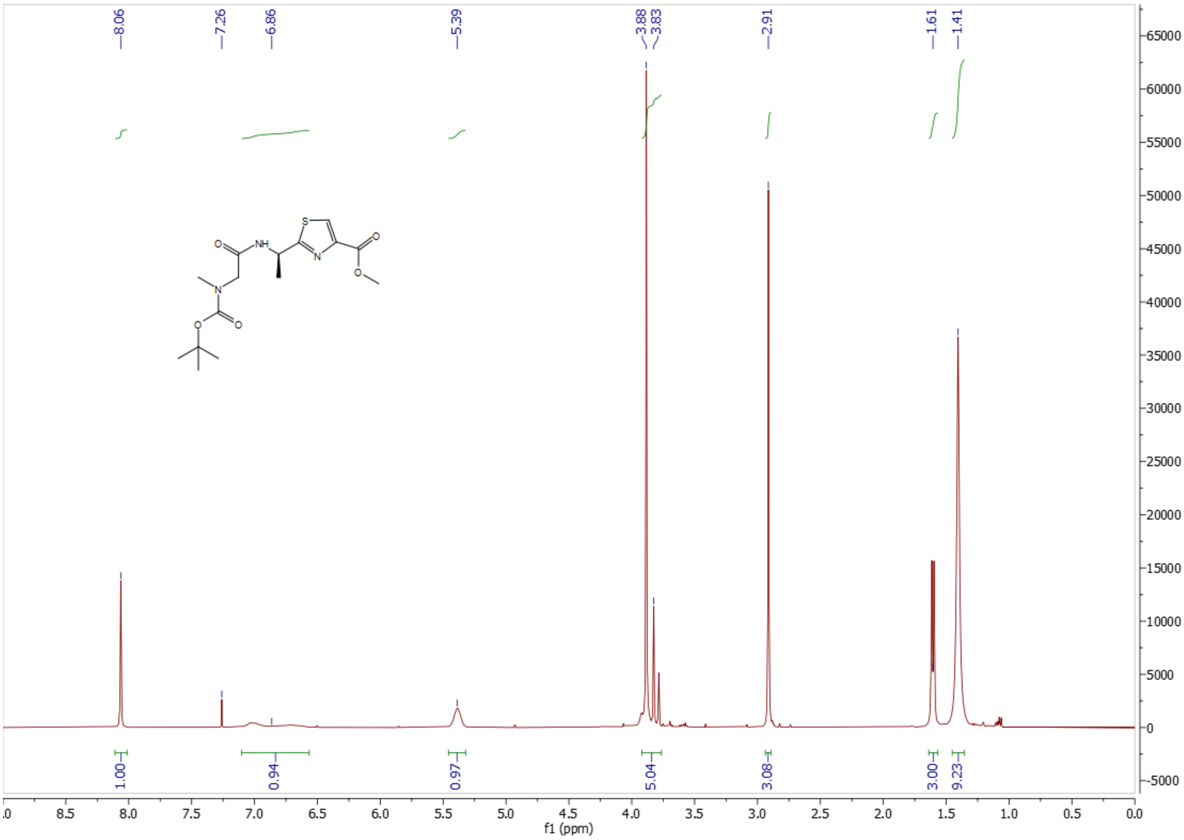

^{13}C NMR of **SI-8** in $CDCl_3$

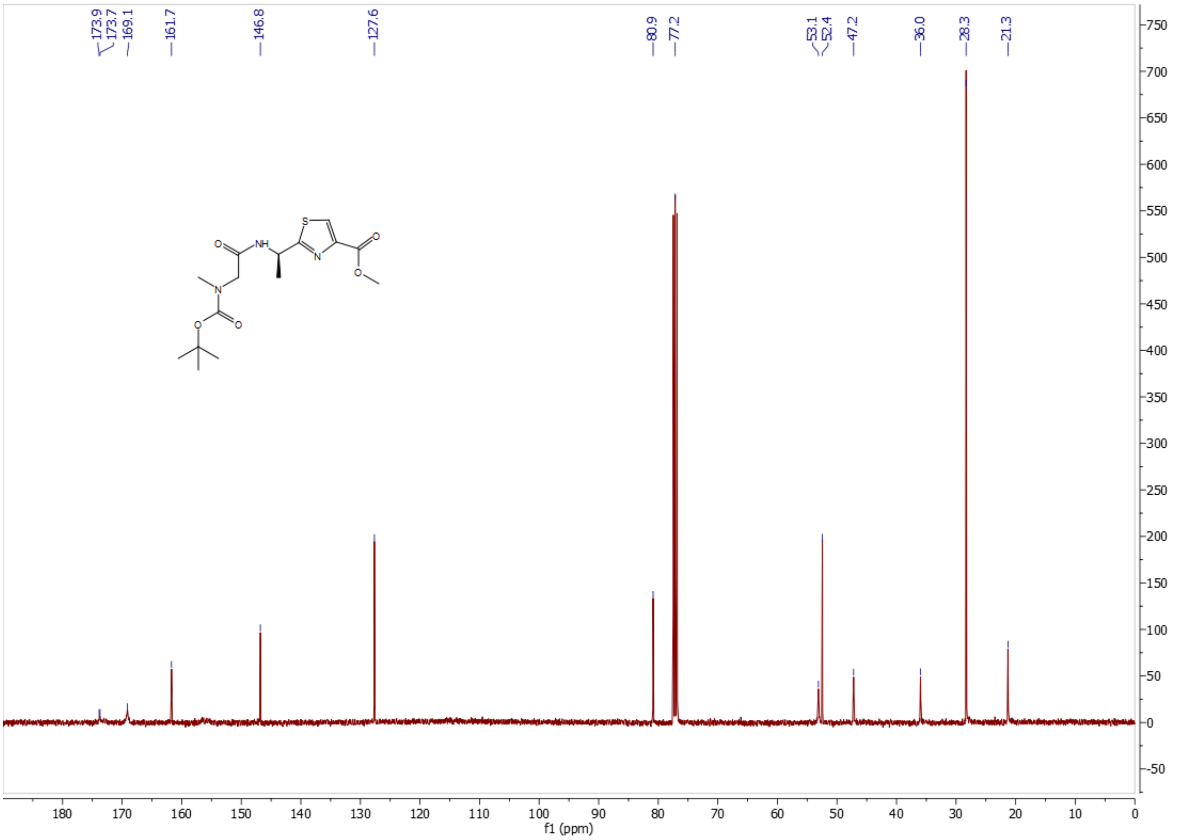

^{1}H NMR of **3** in MeOD

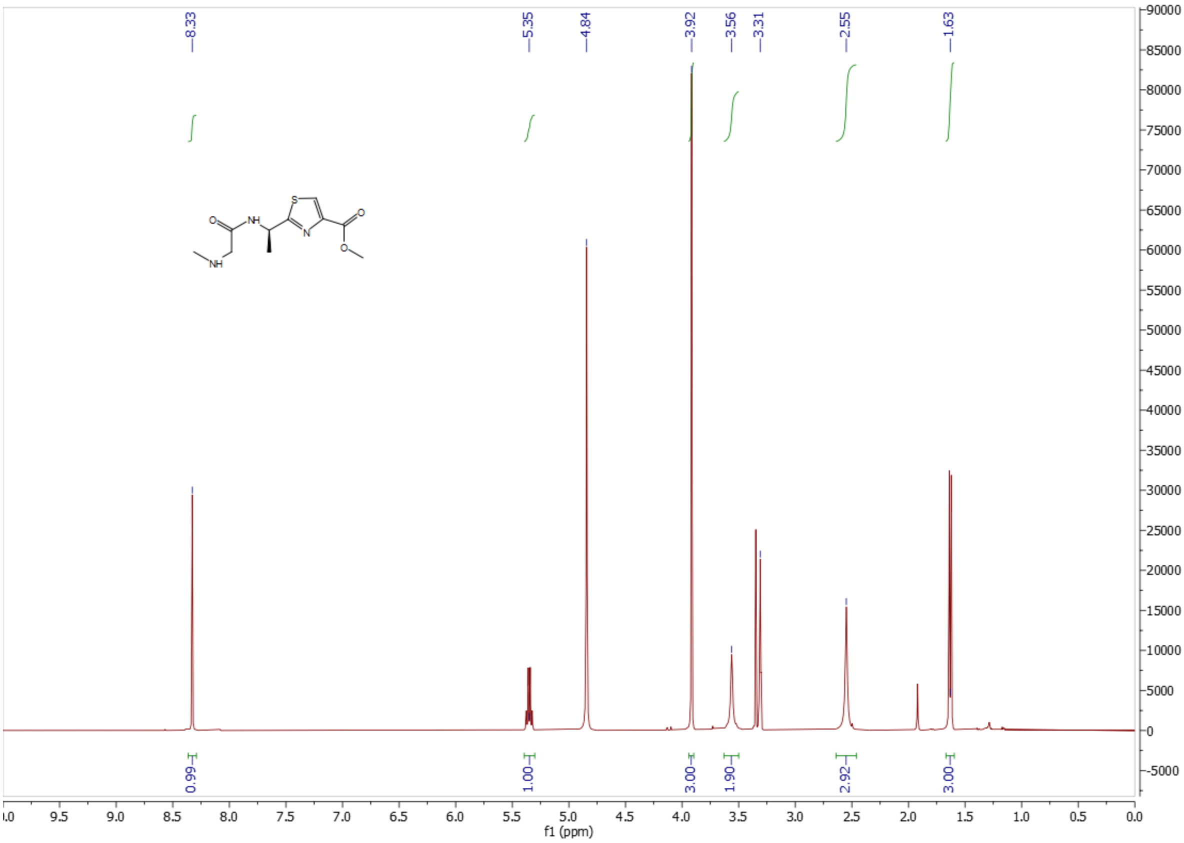

^{13}C NMR of **3** in MeOD

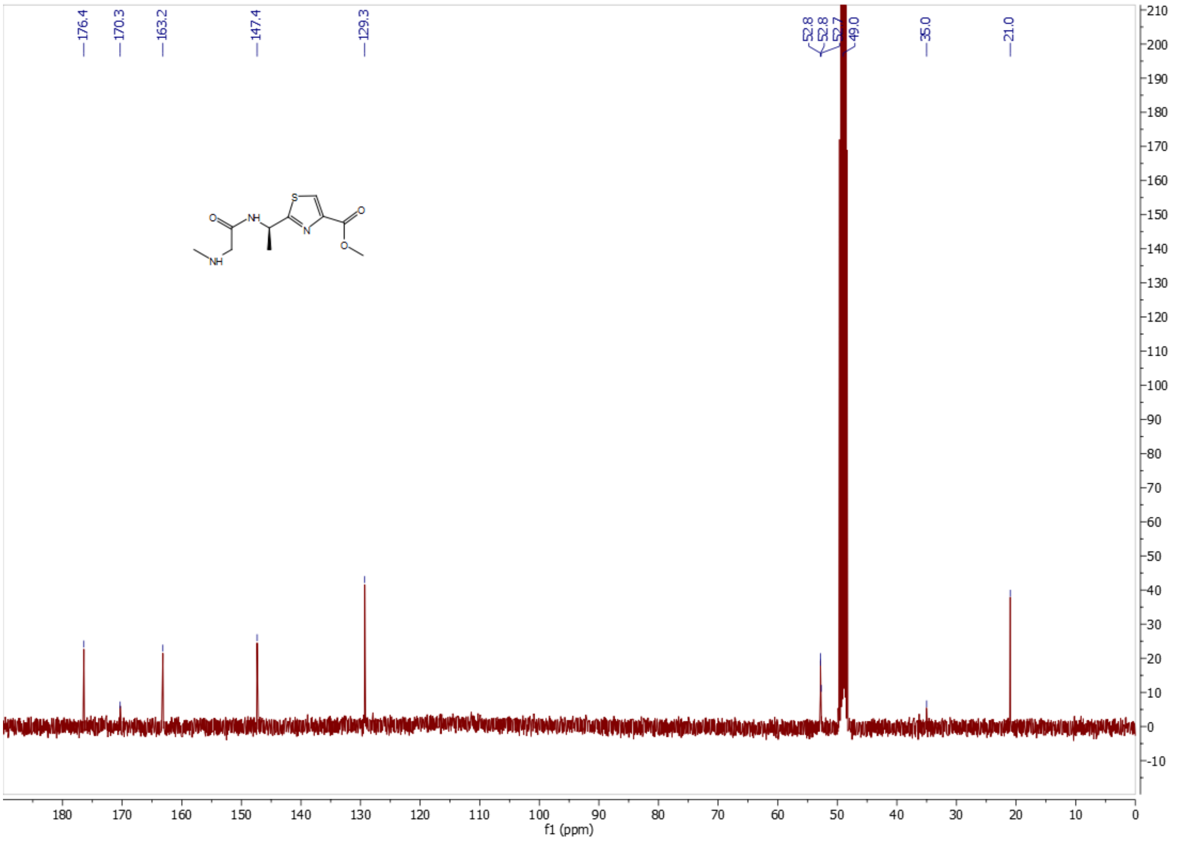

^{1}H NMR of **SI-9** in $CDCl_3$

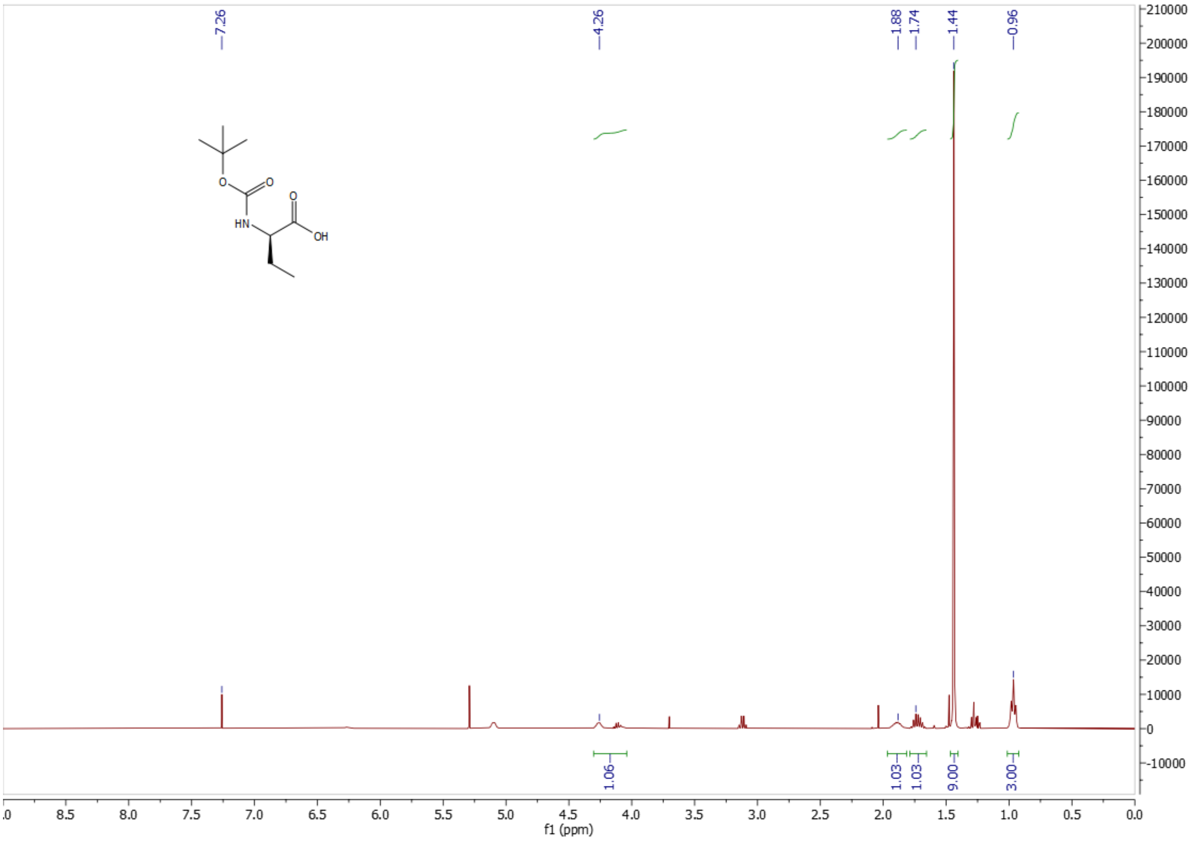

^{13}C NMR of **SI-9** in $CDCl_3$

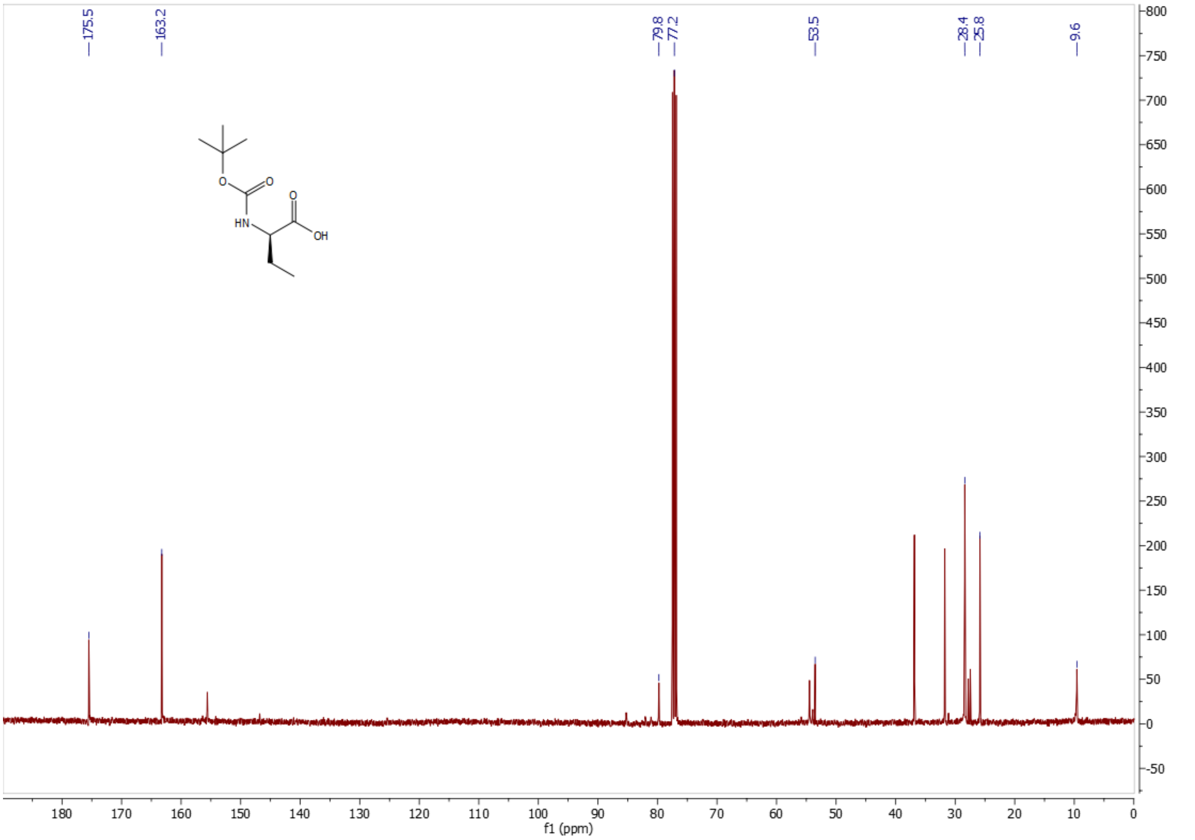

^{1}H NMR of **5** in $CDCl_3$

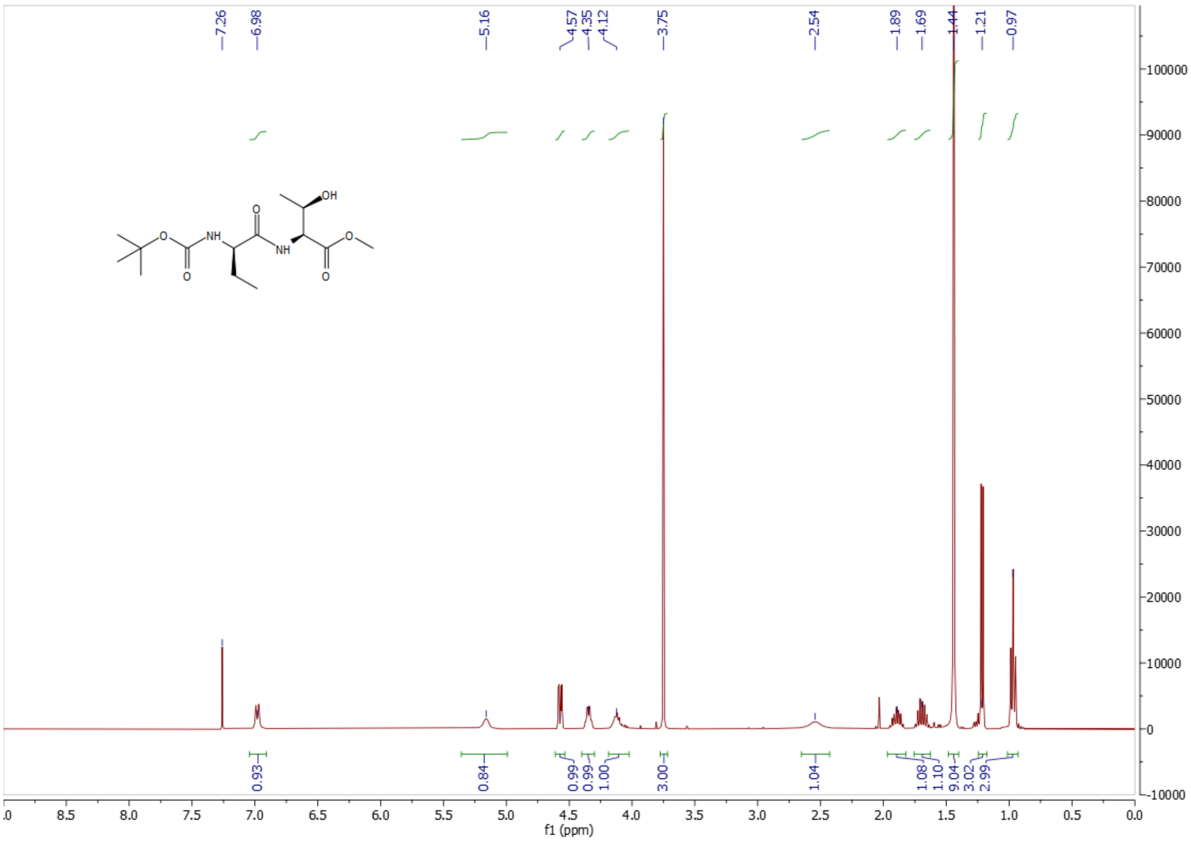

^{13}C NMR of **5** in $CDCl_3$

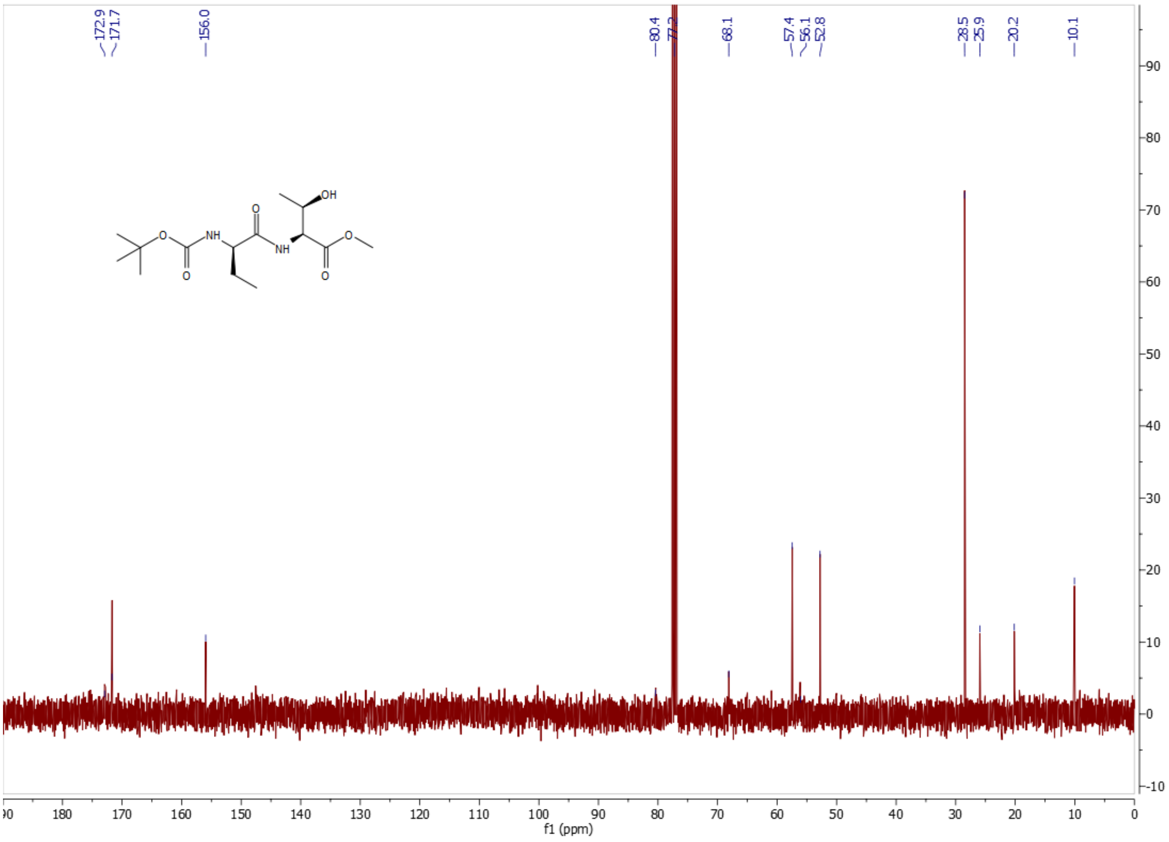

^{1}H NMR of **SI-10** in $CDCl_3$

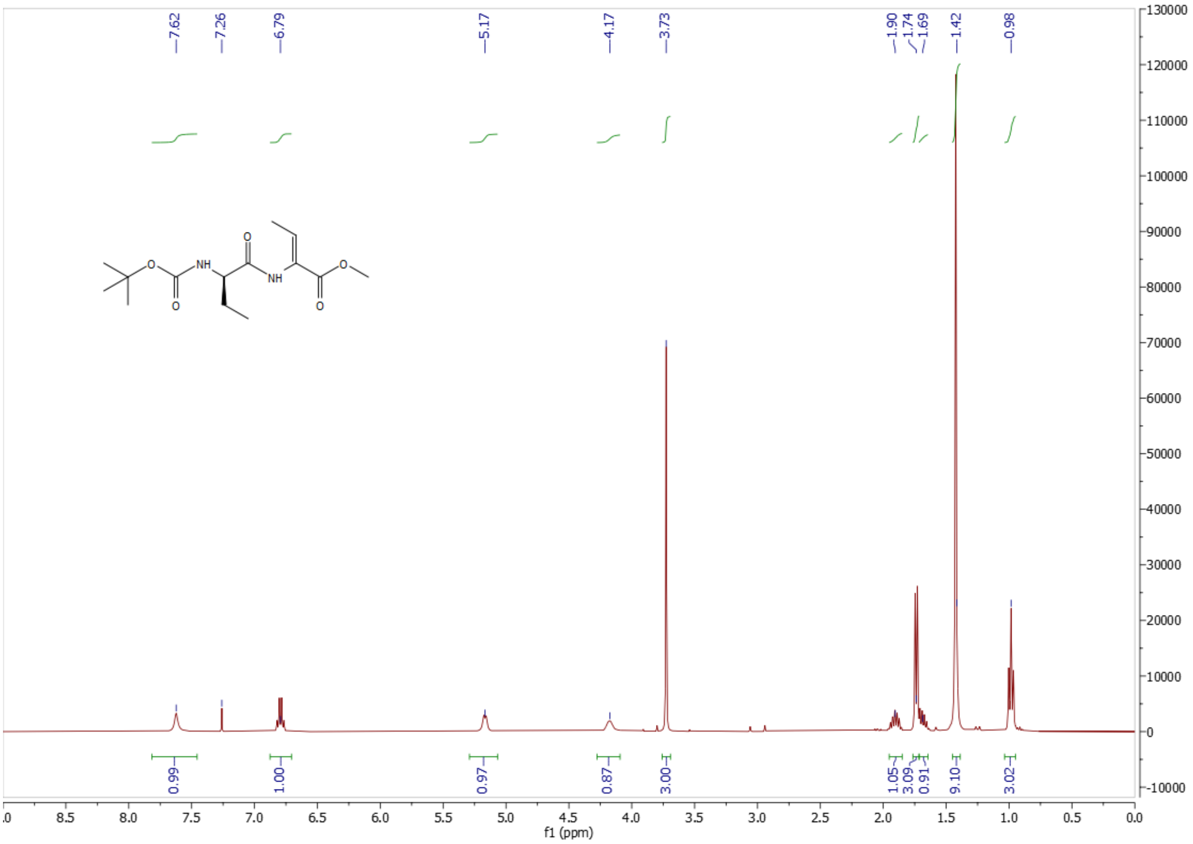

^{13}C NMR of **SI-10** in $CDCl_3$

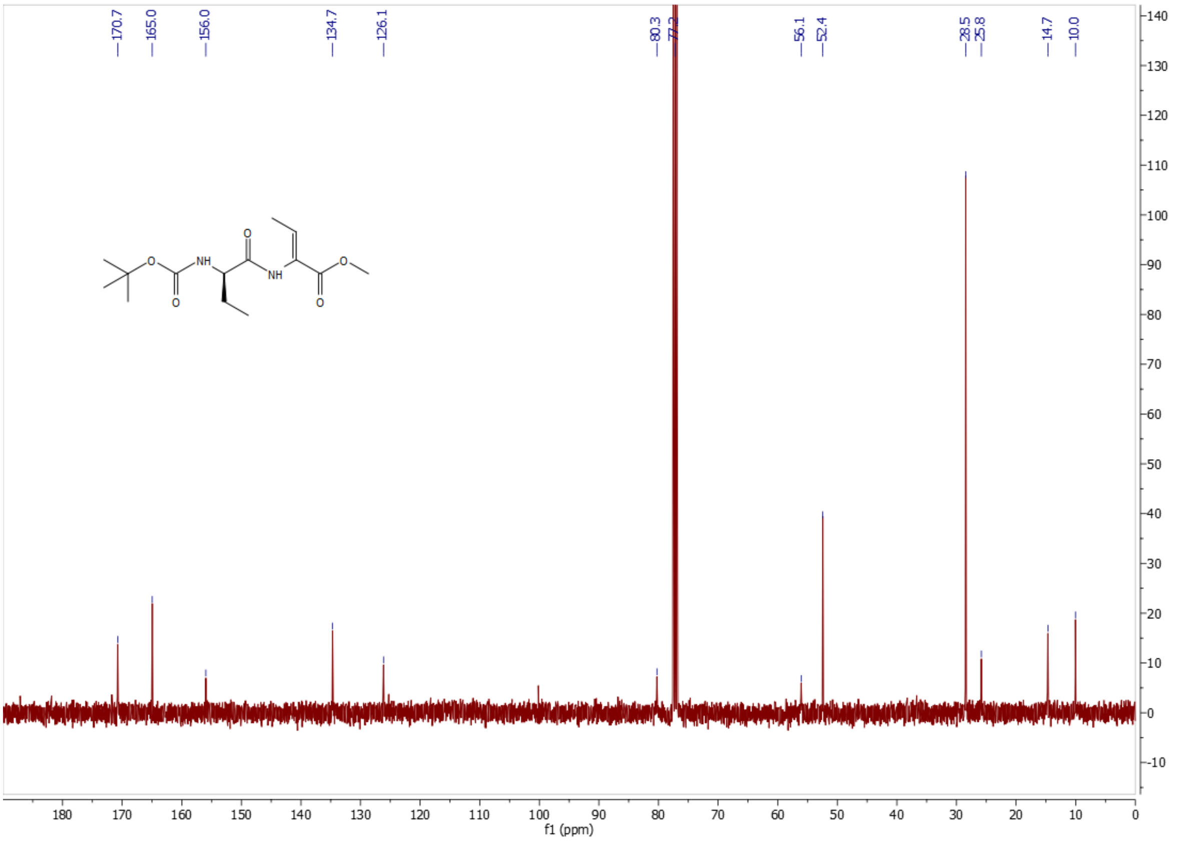

^1H NMR of **6** in MeOD

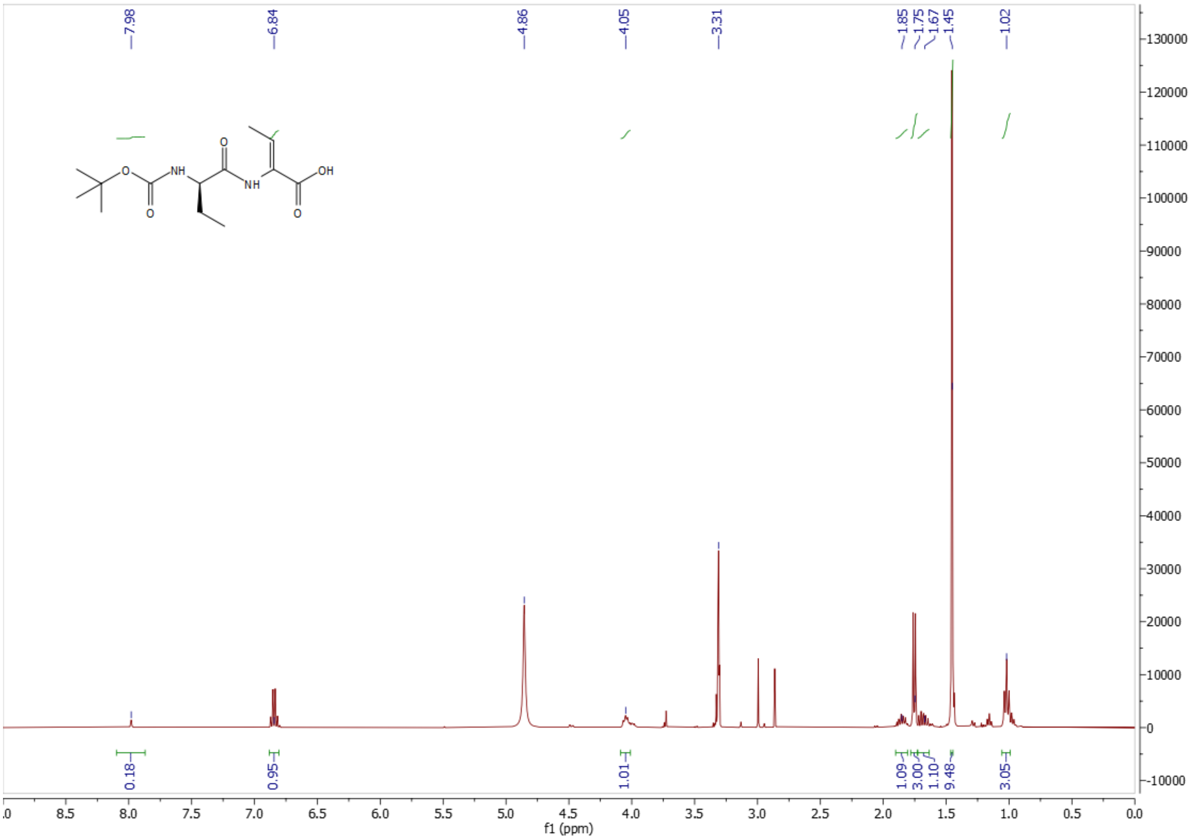

^{13}C NMR of **6** in MeOD

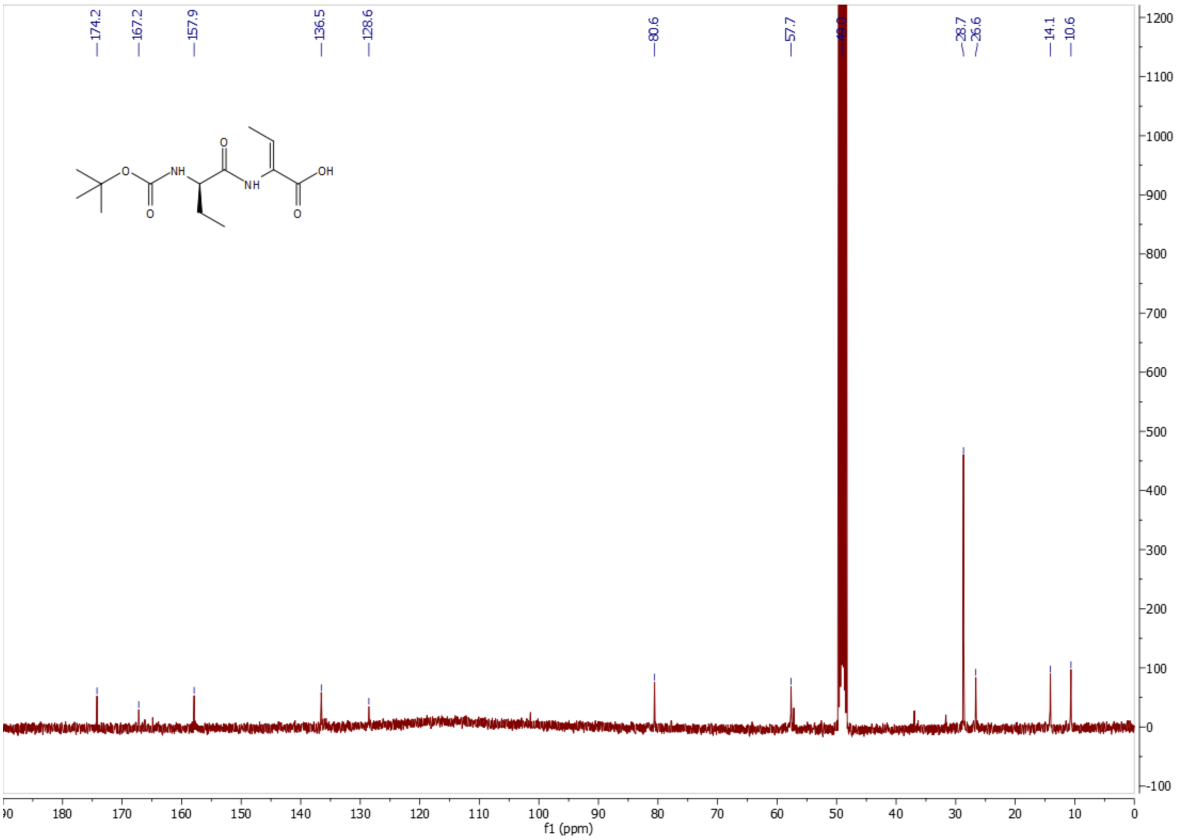

^{1}H NMR of **SI-12** in $CDCl_3$

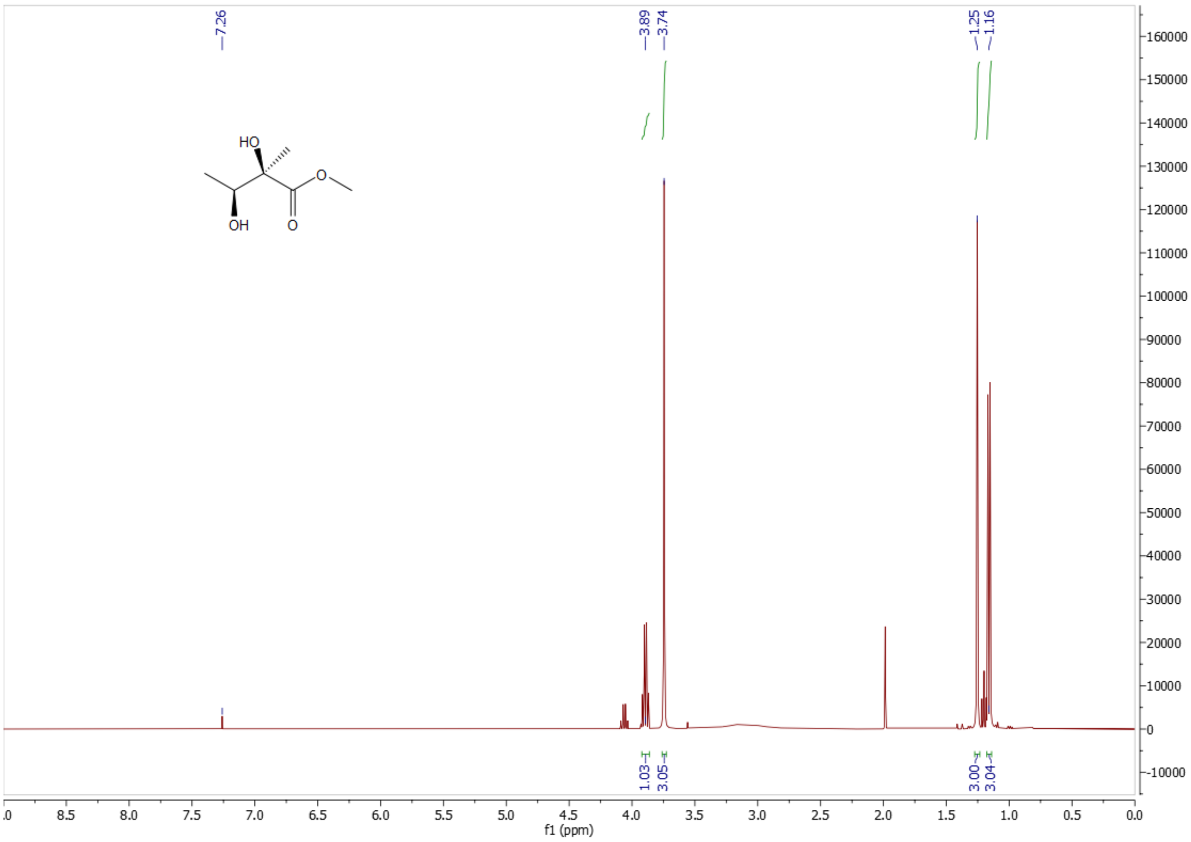

^{13}C NMR of **SI-12** in $CDCl_3$

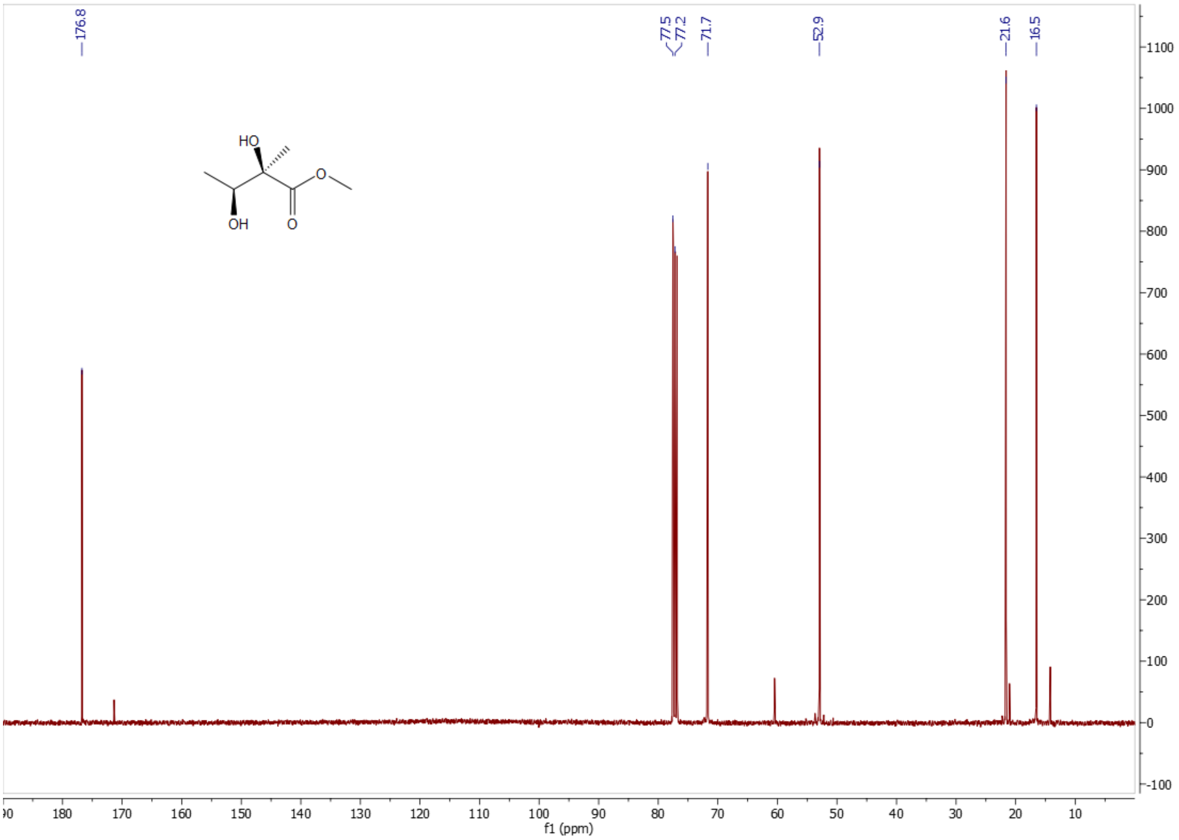

^{1}H NMR of **SI-13** in $CDCl_3$

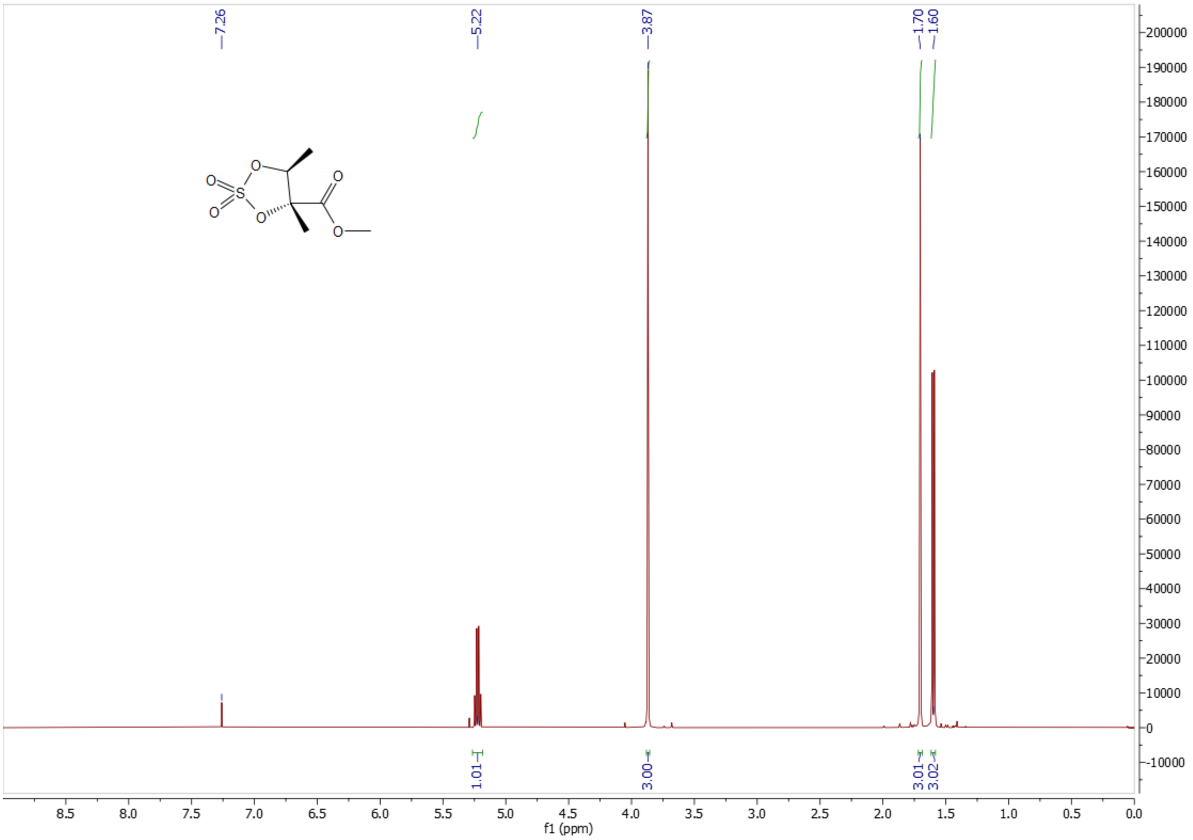

^{13}C NMR of **SI-13** in $CDCl_3$

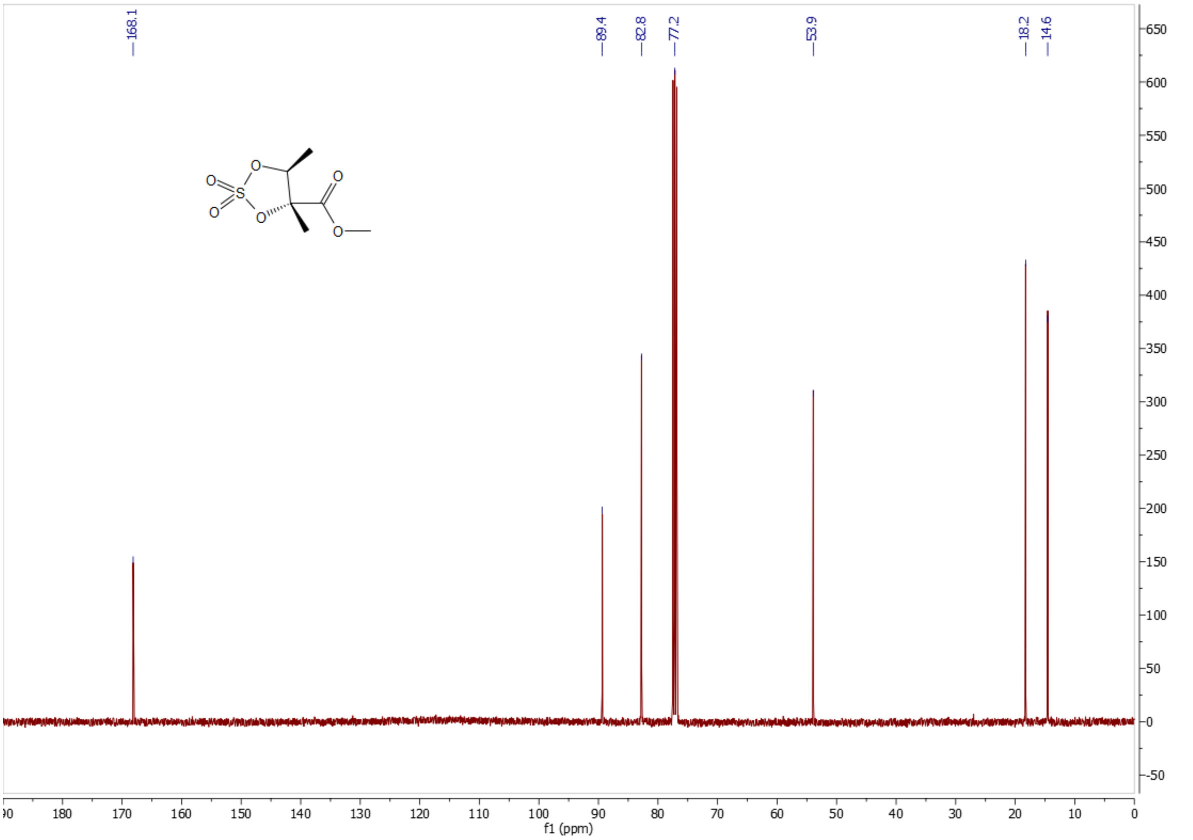

^{1}H NMR of **SI-14** in MeOD

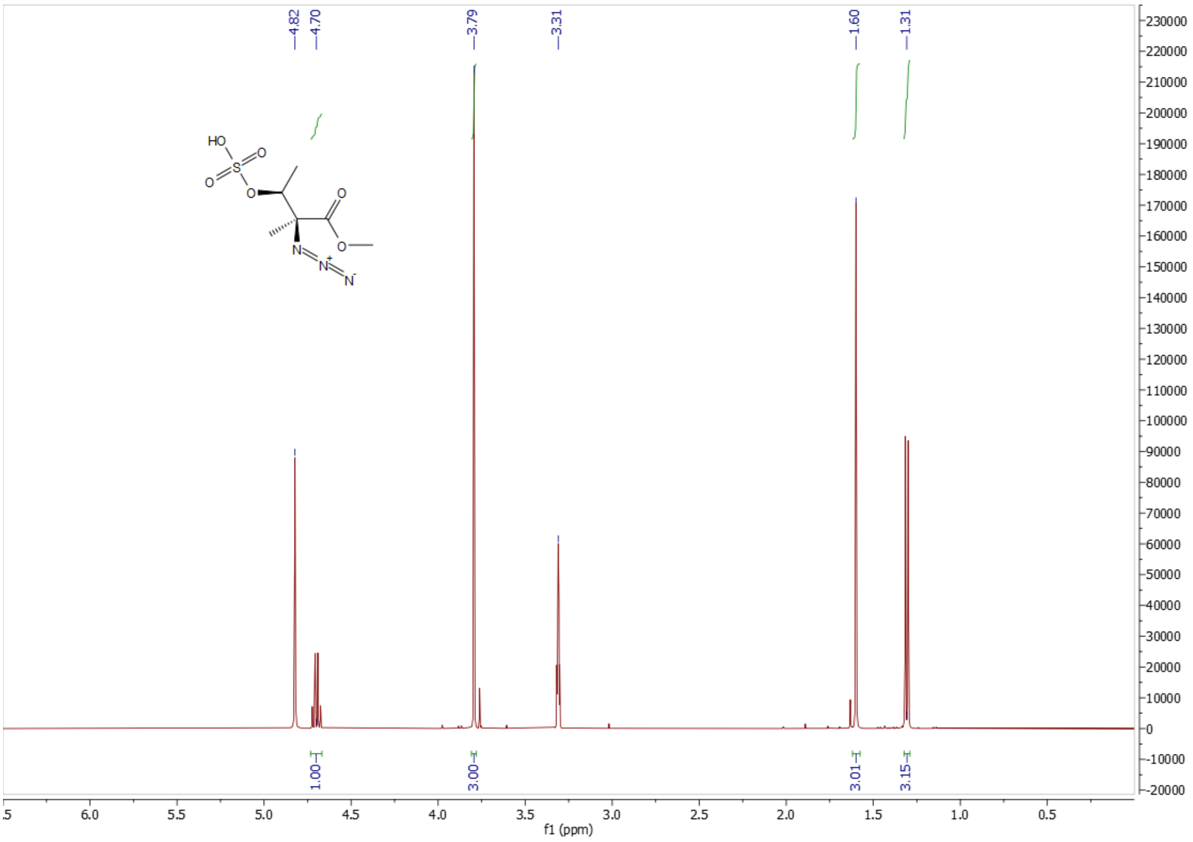

^{13}C NMR of **SI-14** in MeOD

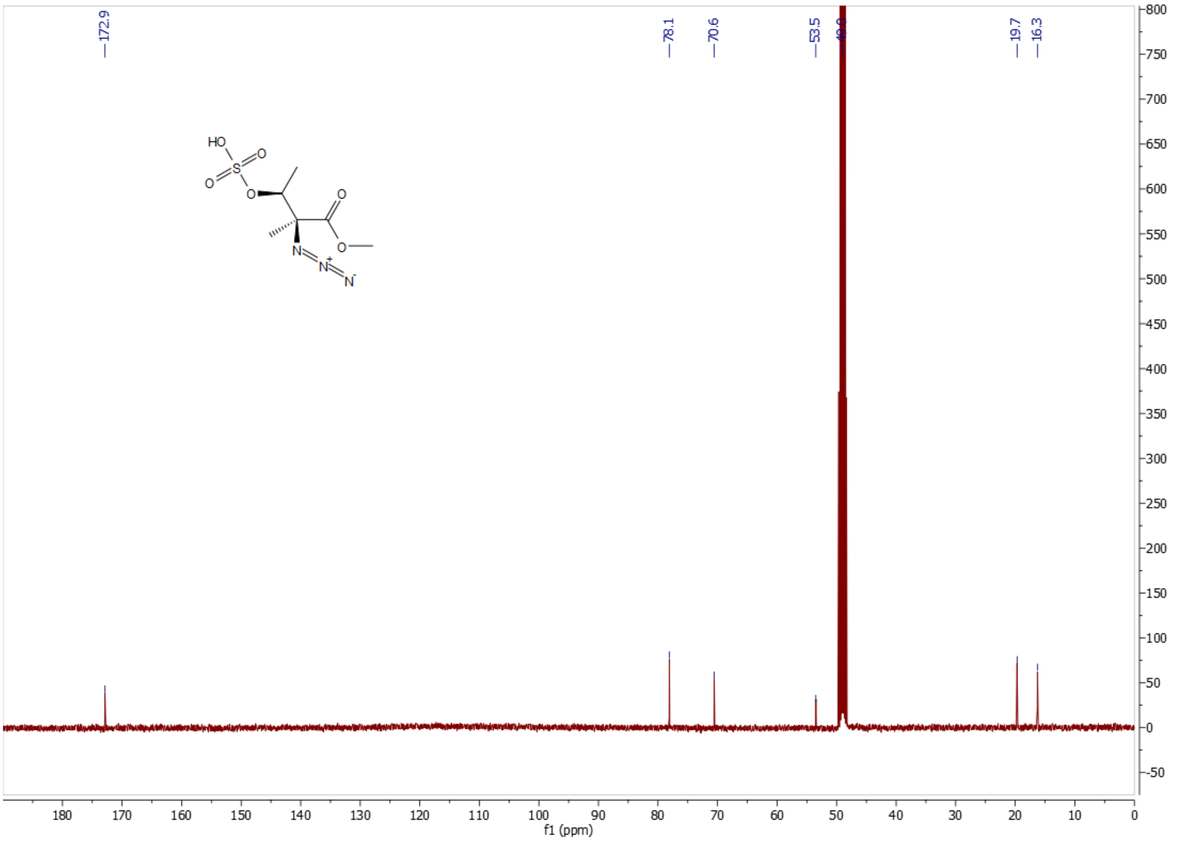

^{1}H NMR of **SI-15** in MeOD

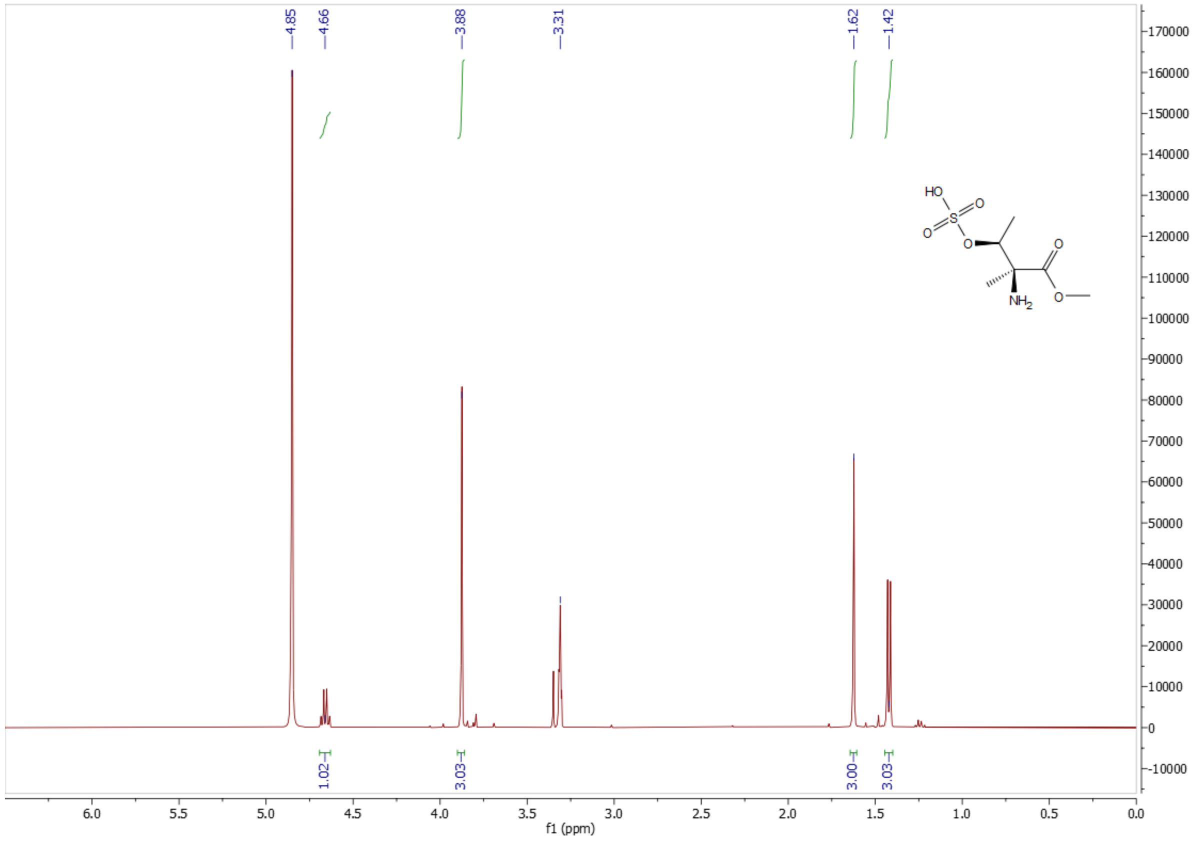

^{13}C NMR of **SI-15** in MeOD

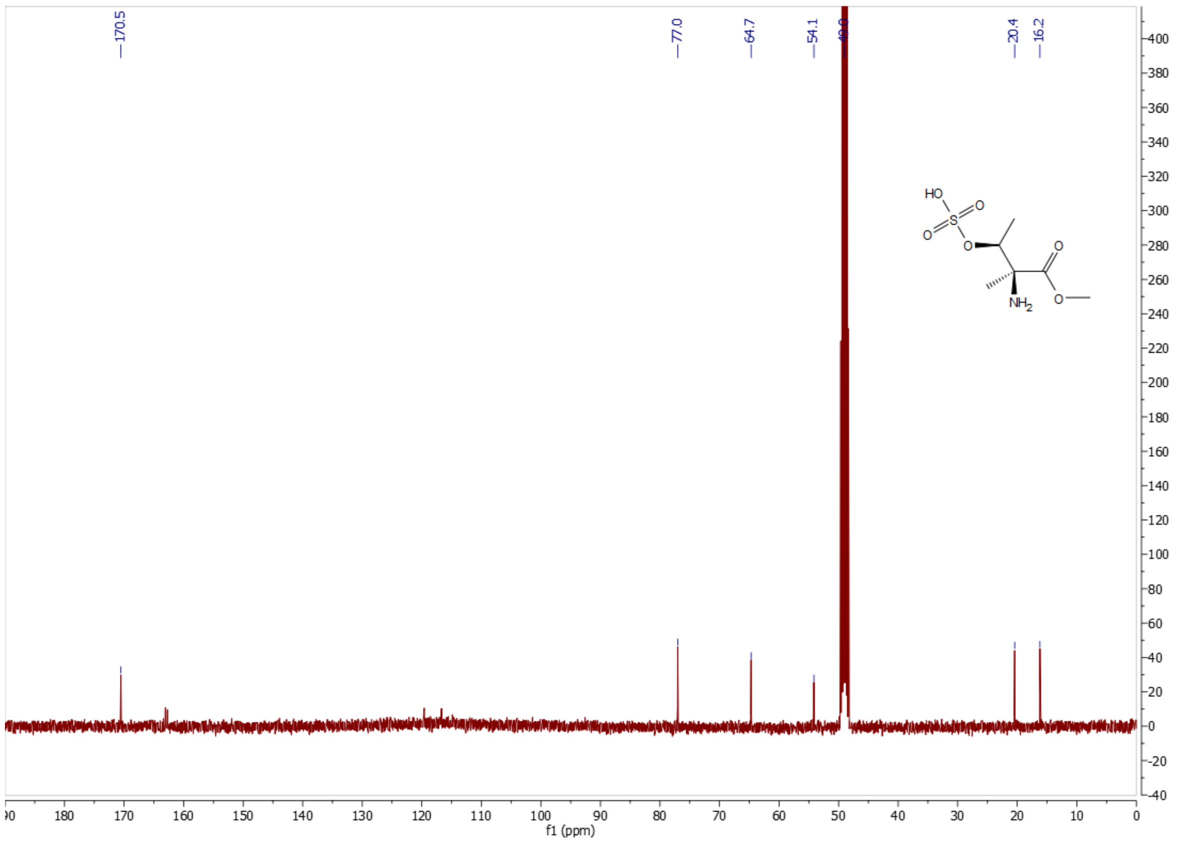

^{1}H NMR of **7** in MeOD

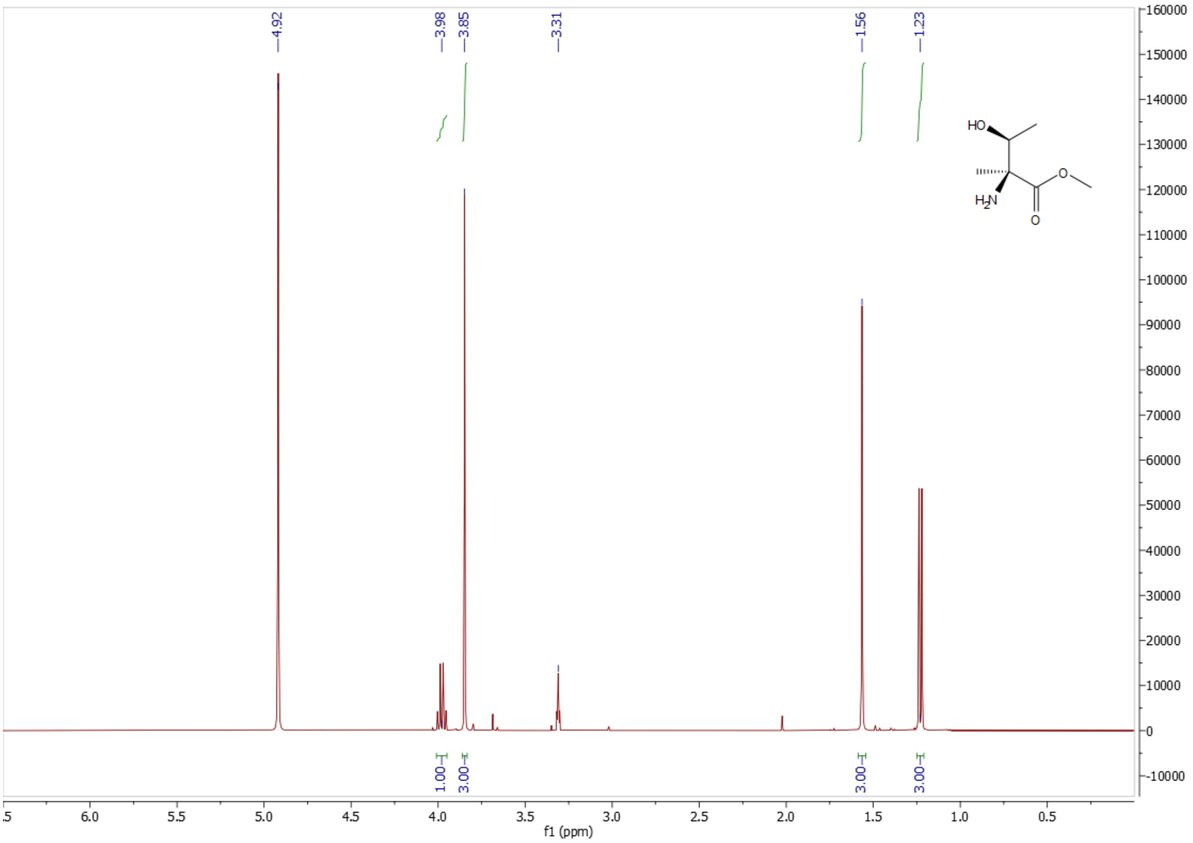

^{13}C NMR of **7** in MeOD

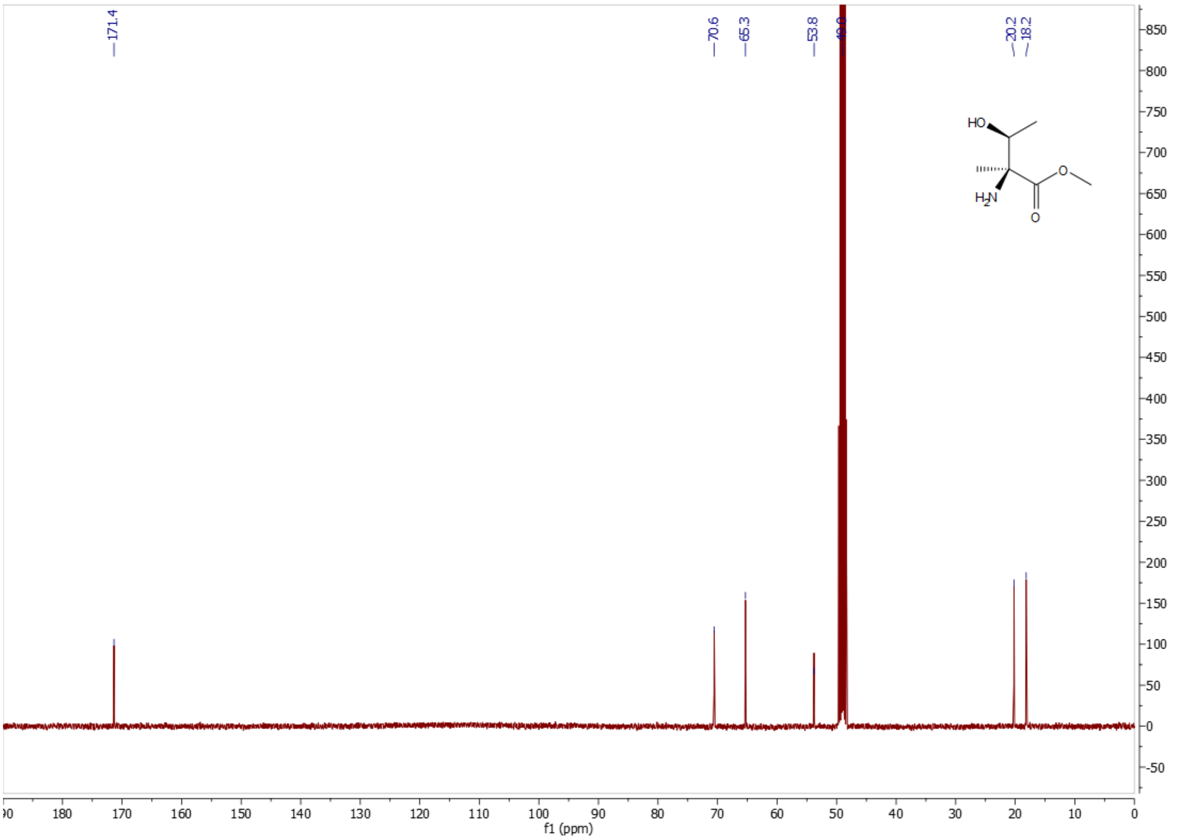

^{1}H NMR of **SI-17** in $CDCl_3$

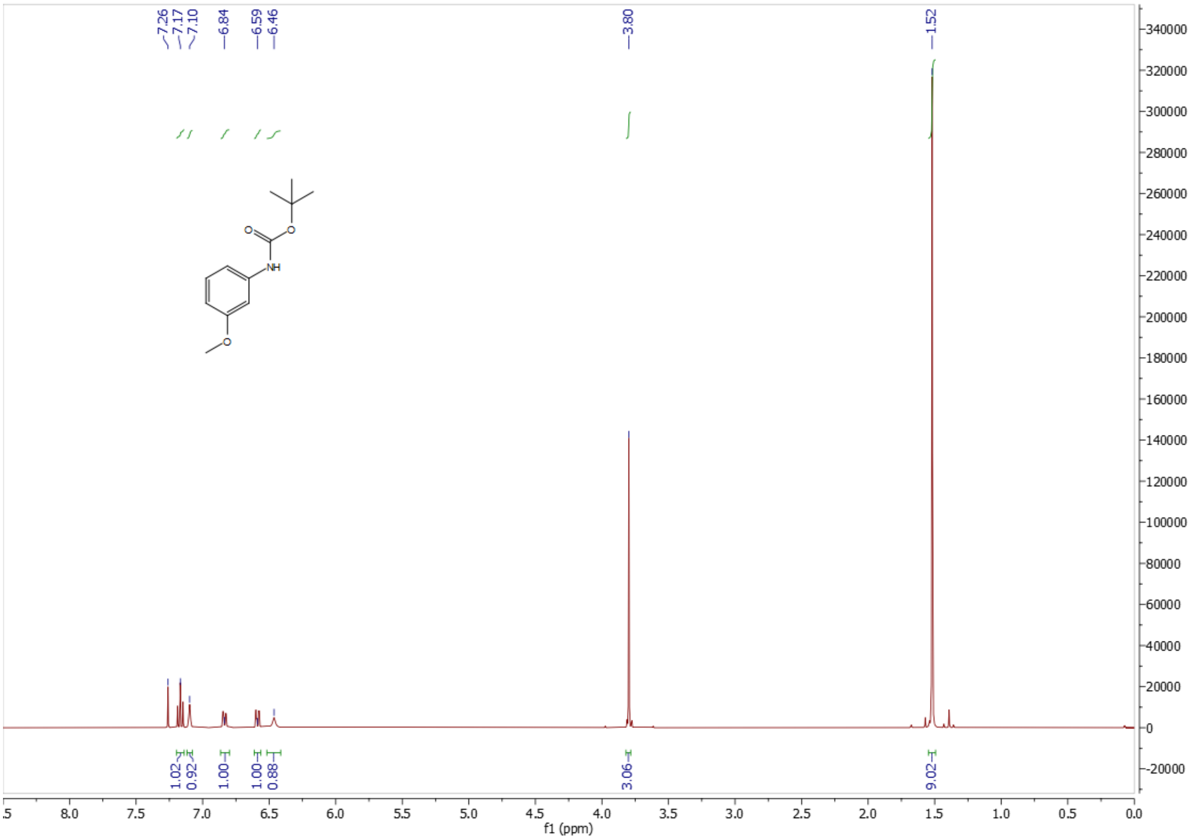

^{13}C NMR of **SI-17** in $CDCl_3$

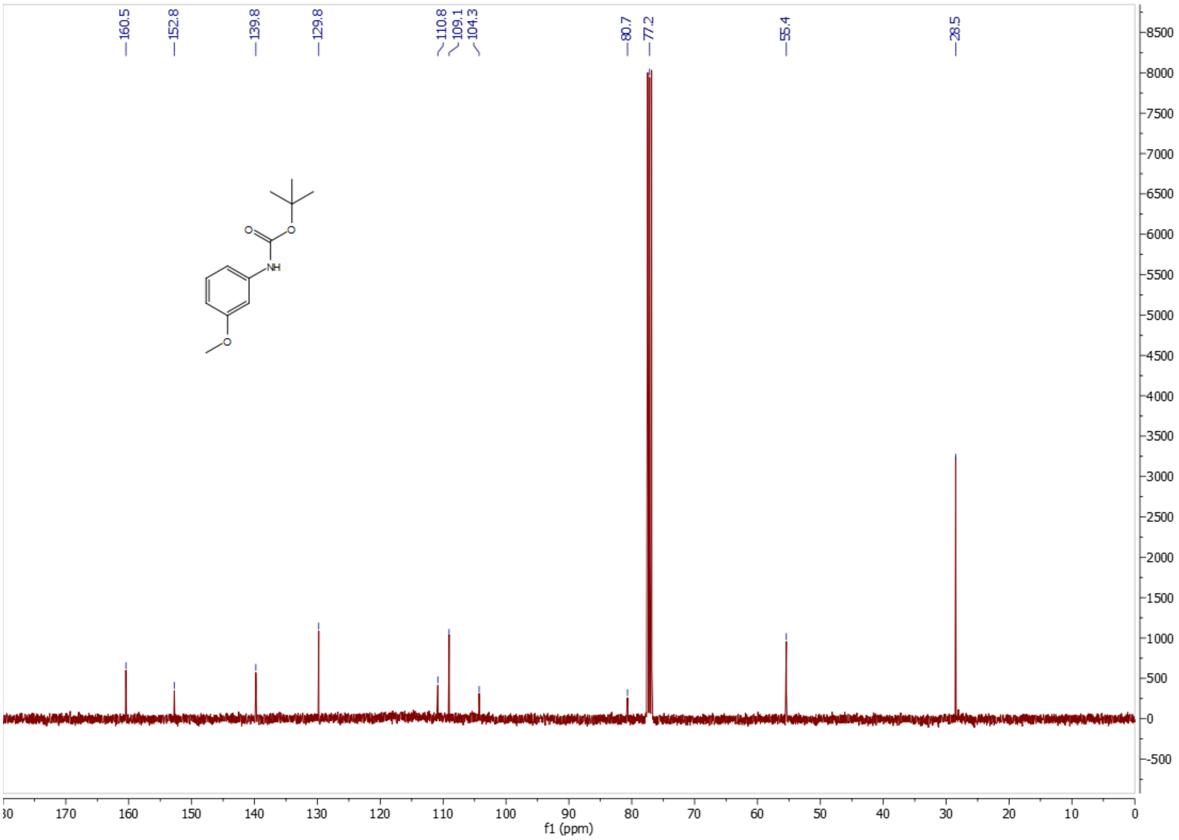

^1H NMR of **SI-18** in $CDCl_3$

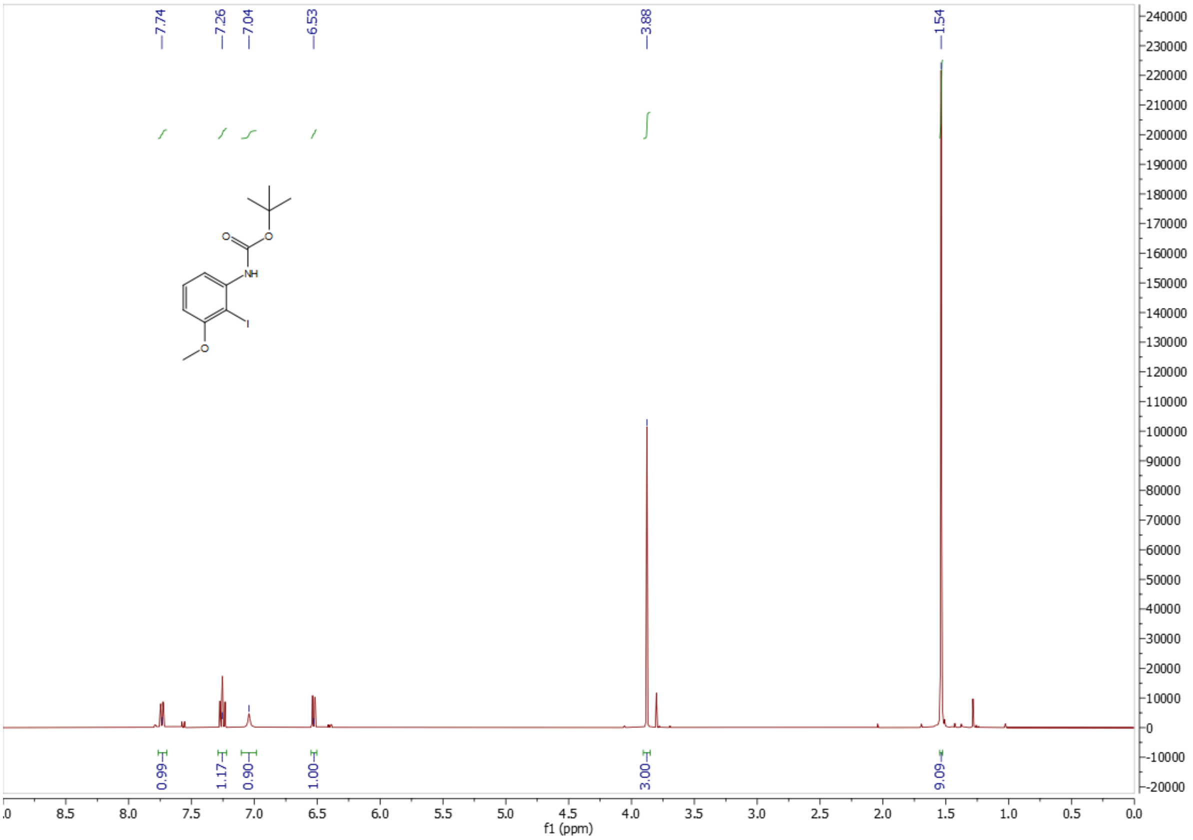

^{13}C NMR of **SI-18** in $CDCl_3$

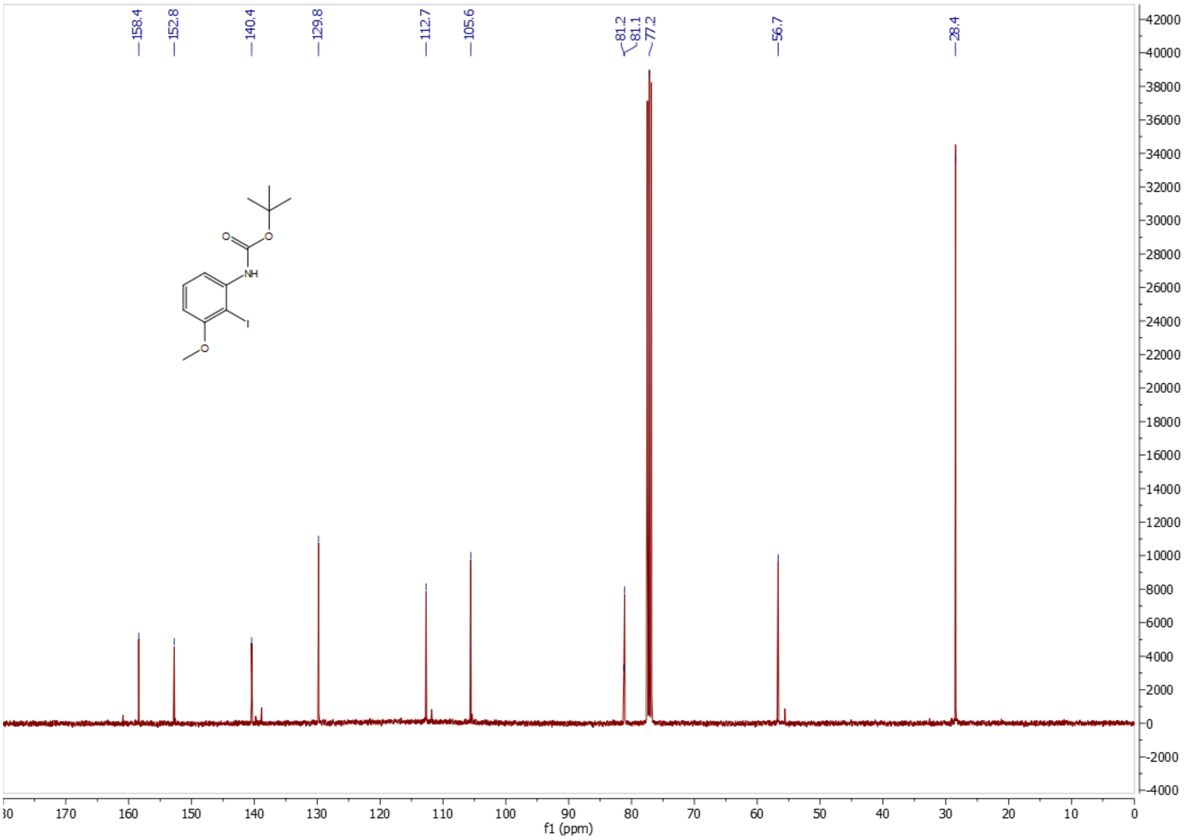

^{1}H NMR of **SI-19** in MeOD

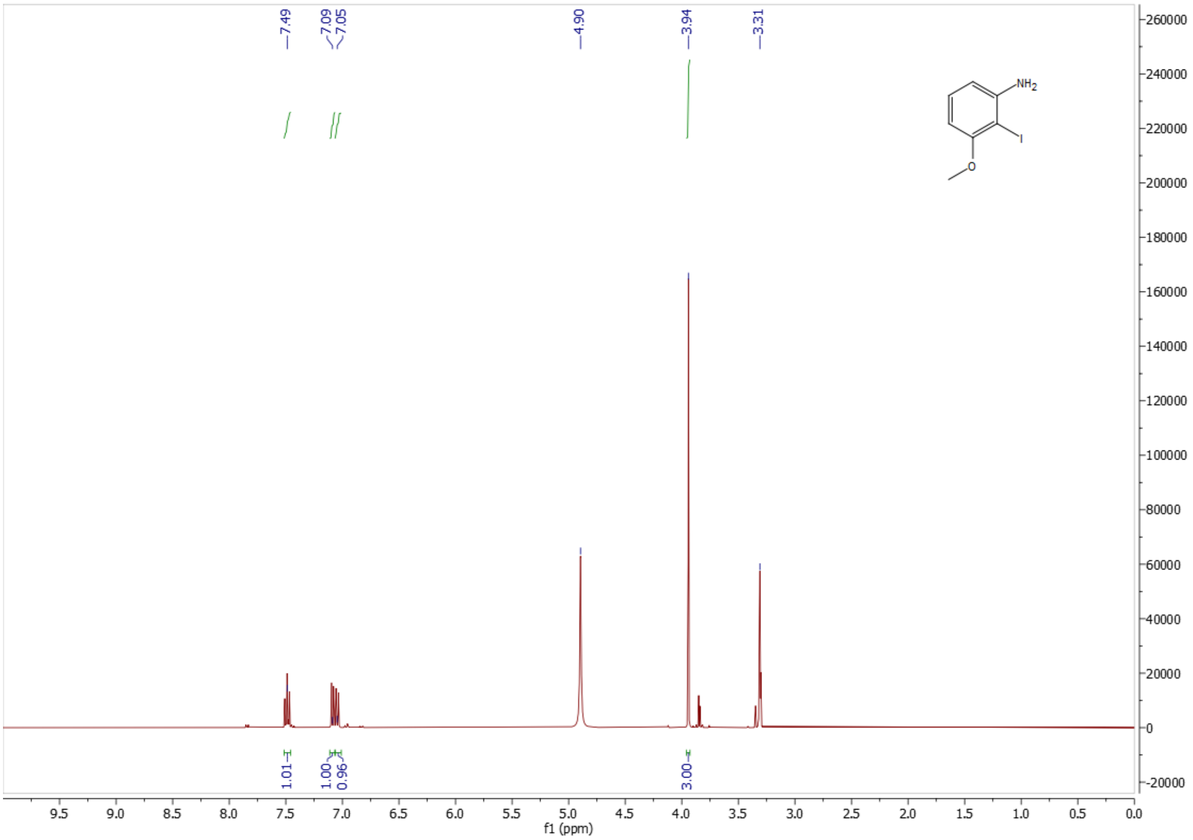

^{13}C NMR of **SI-19** in MeOD

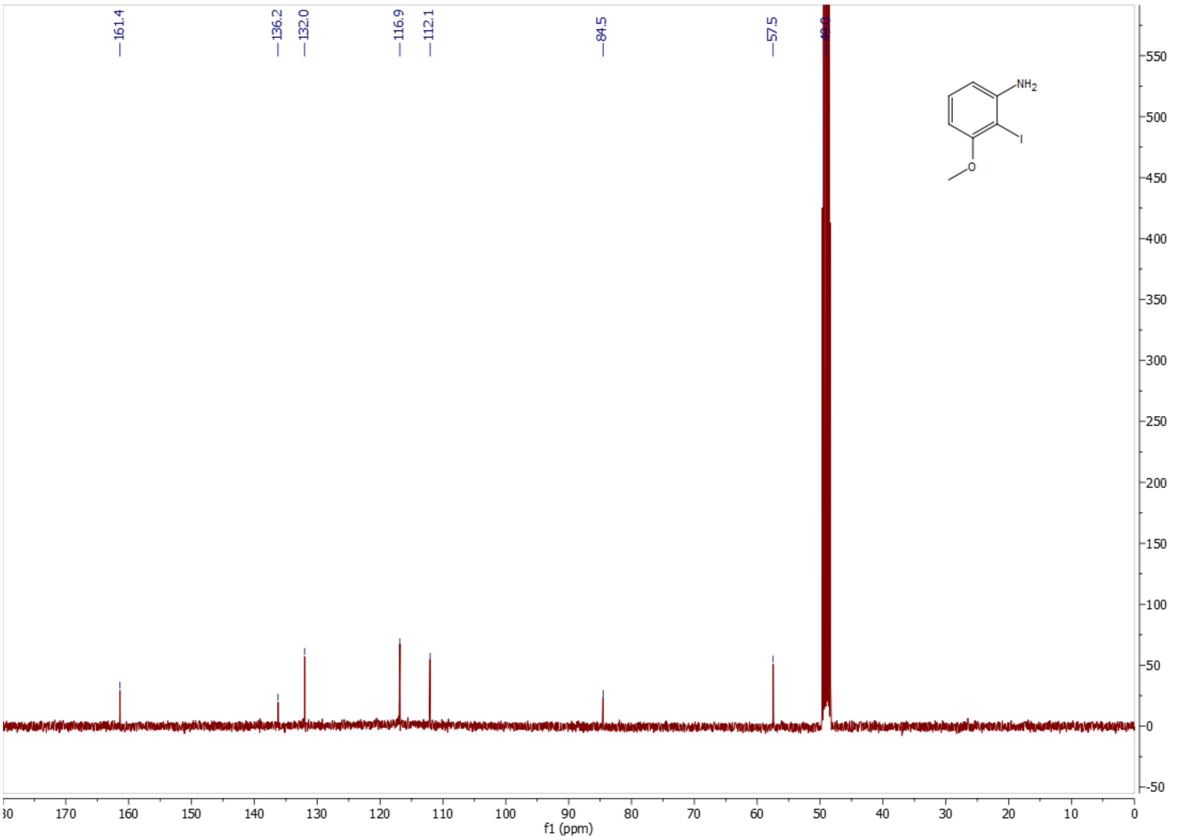

^{1}H NMR of **SI-21** in $CDCl_3$

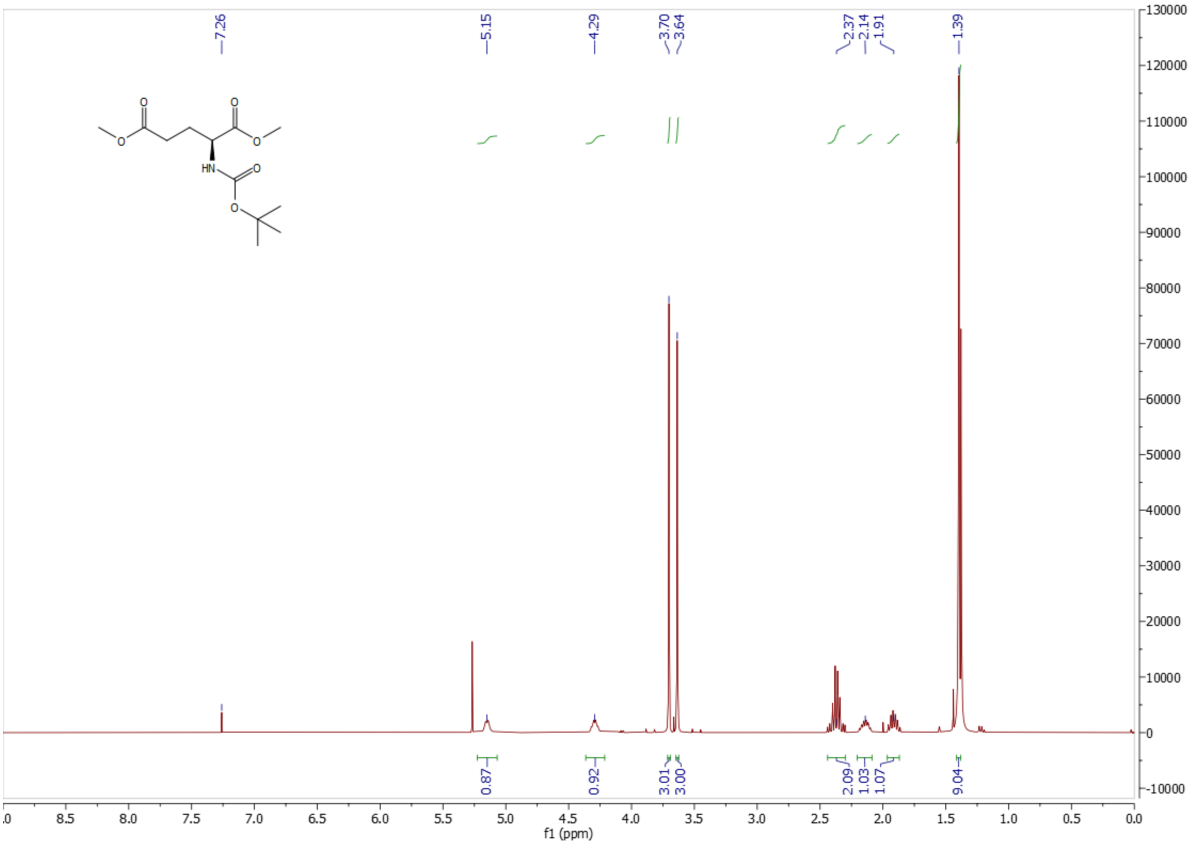

^{13}C NMR of **SI-21** in $CDCl_3$

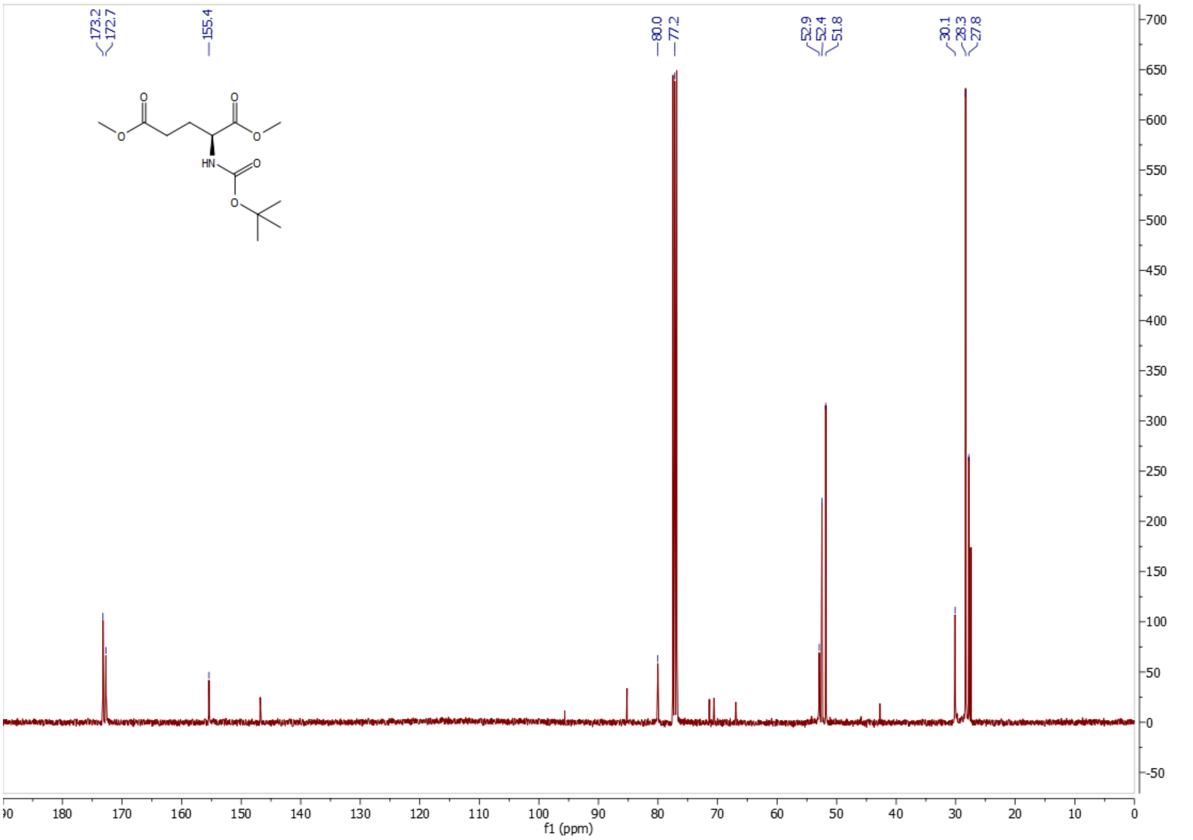

^{1}H NMR of **SI-22** in $CDCl_3$

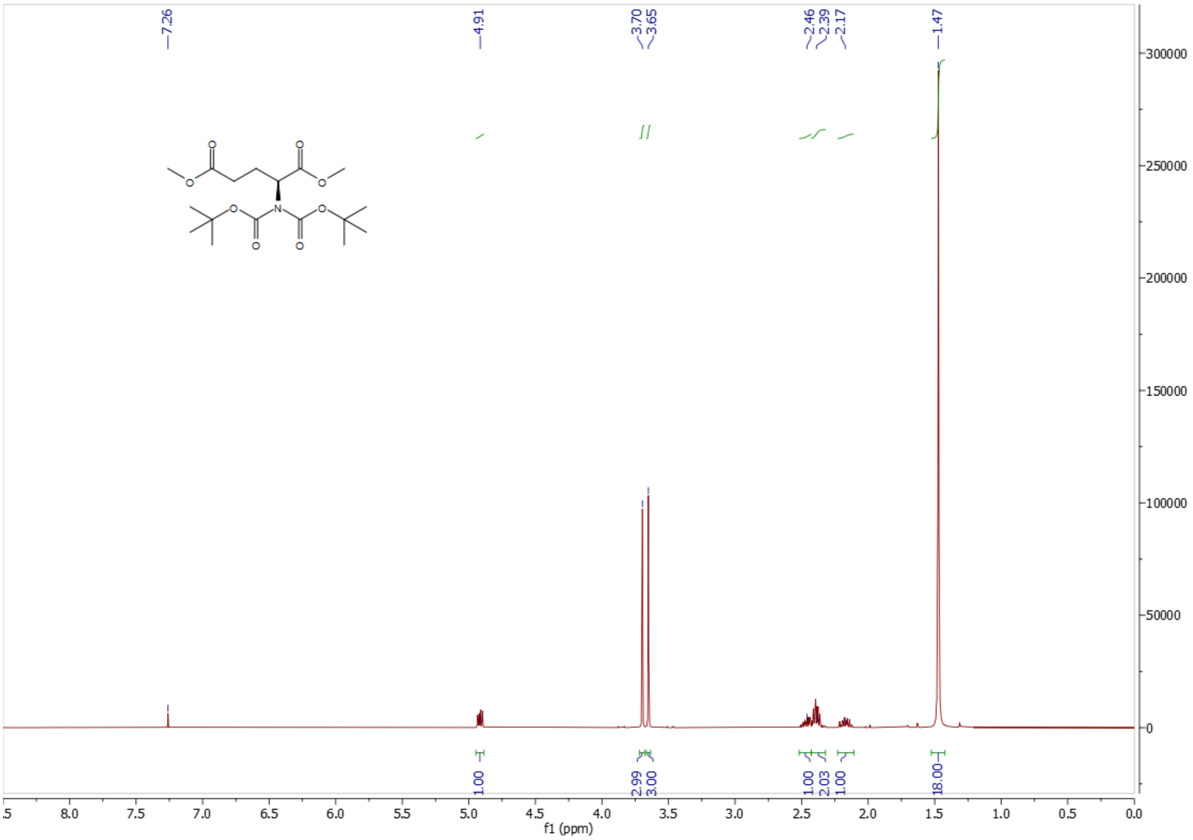

^{13}C NMR of **SI-22** in $CDCl_3$

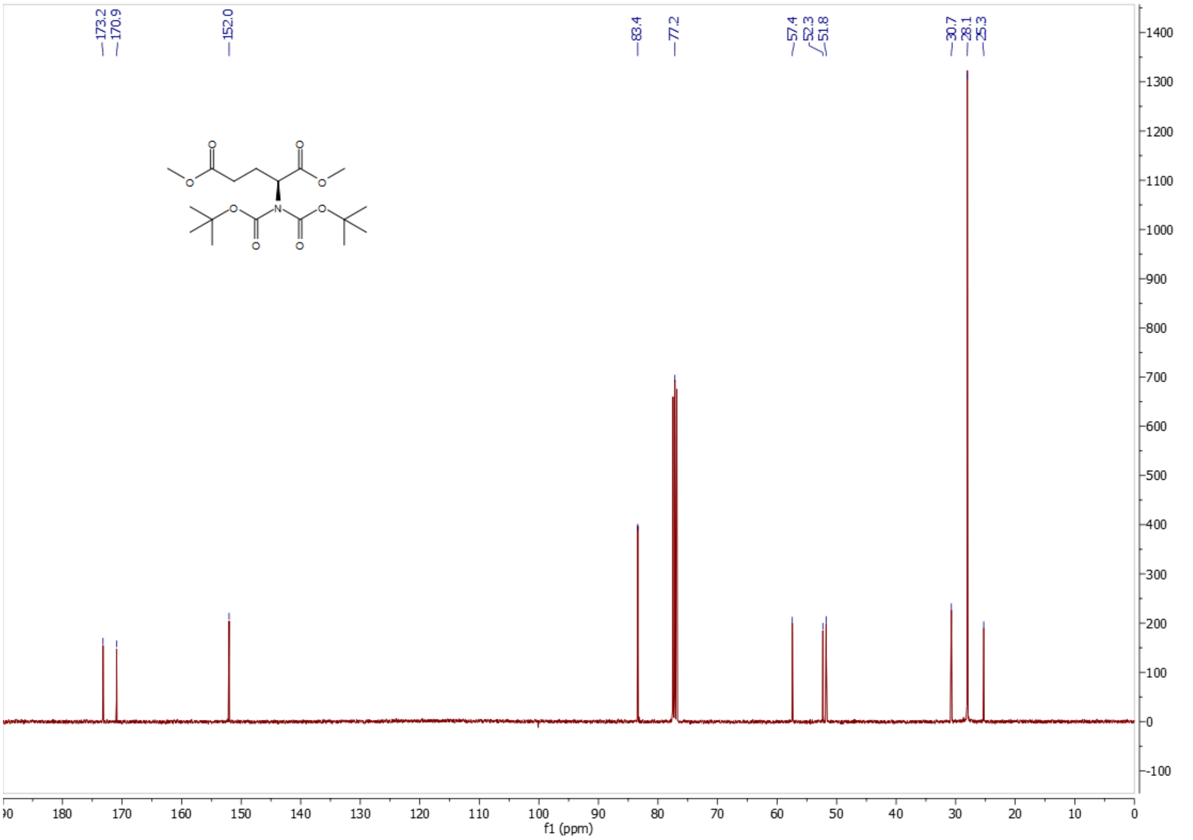

^1H NMR of **SI-23** in $CDCl_3$

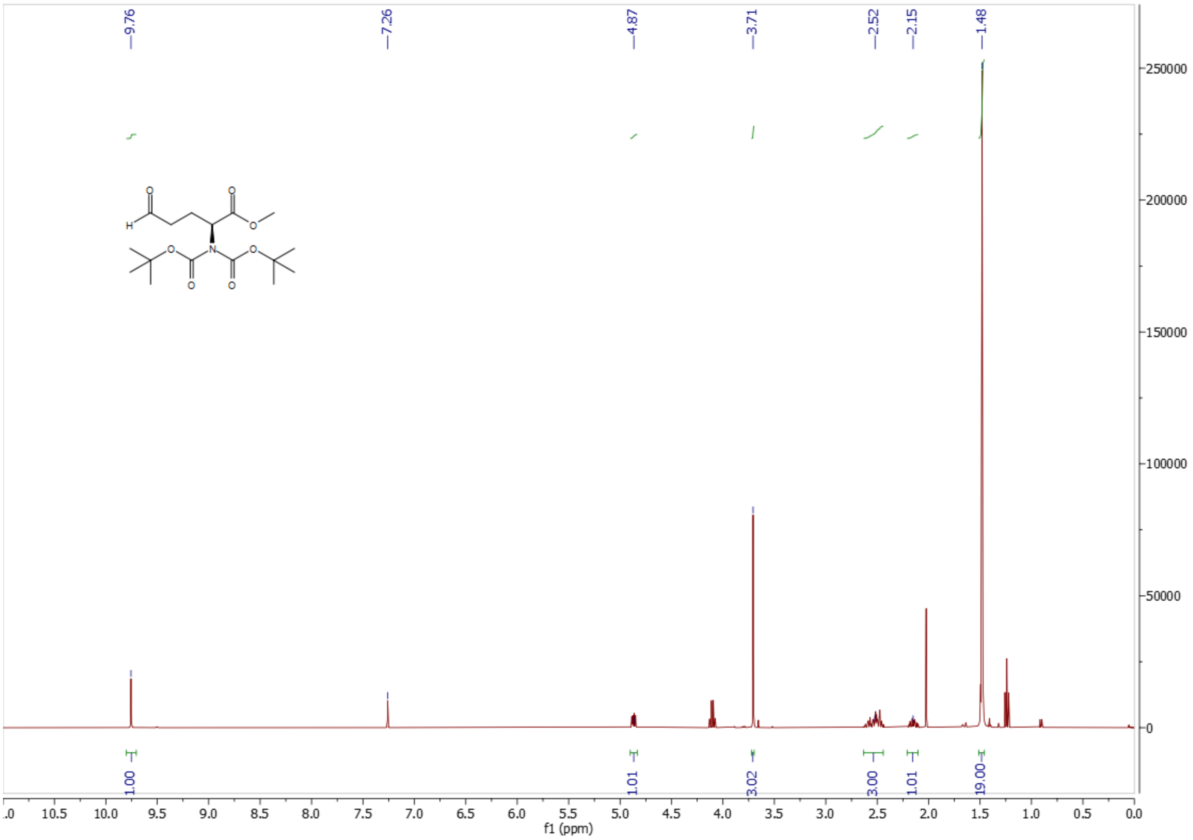

^{13}C NMR of **SI-23** in $CDCl_3$

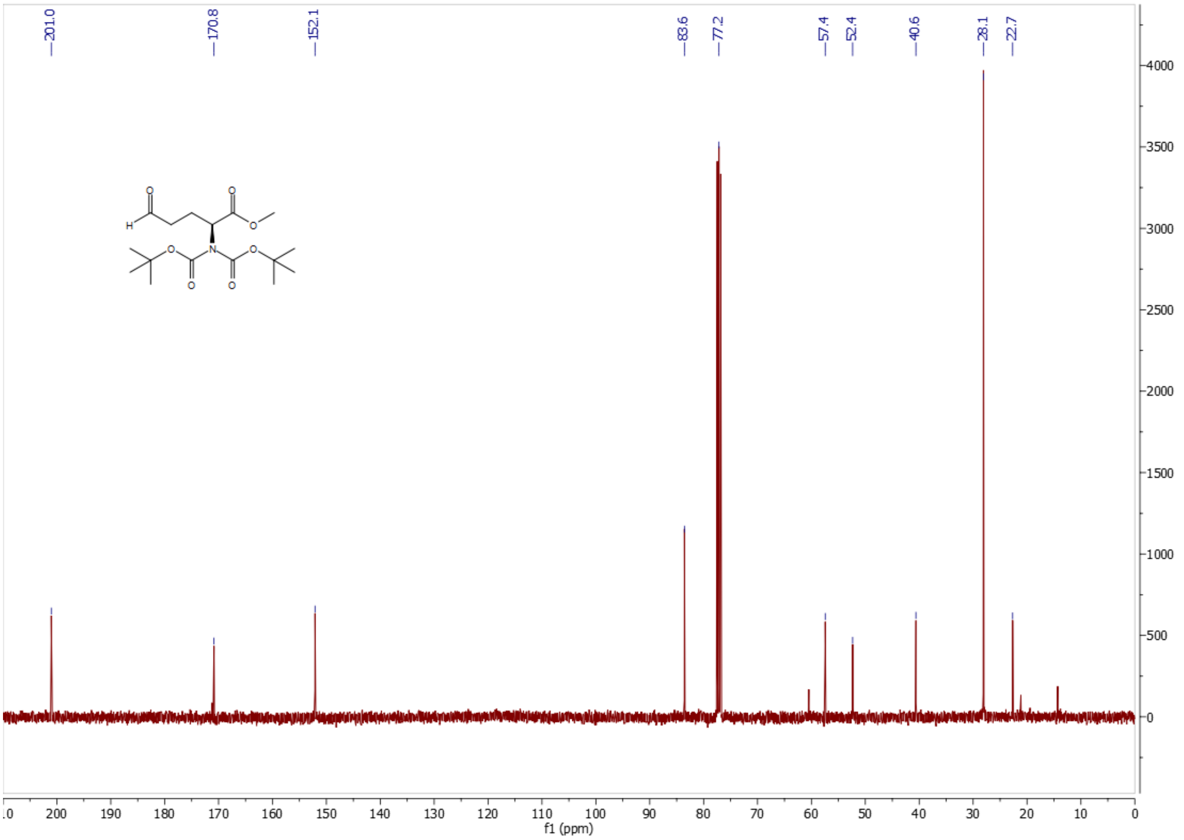

^{1}H NMR of **SI-24** in $CDCl_3$

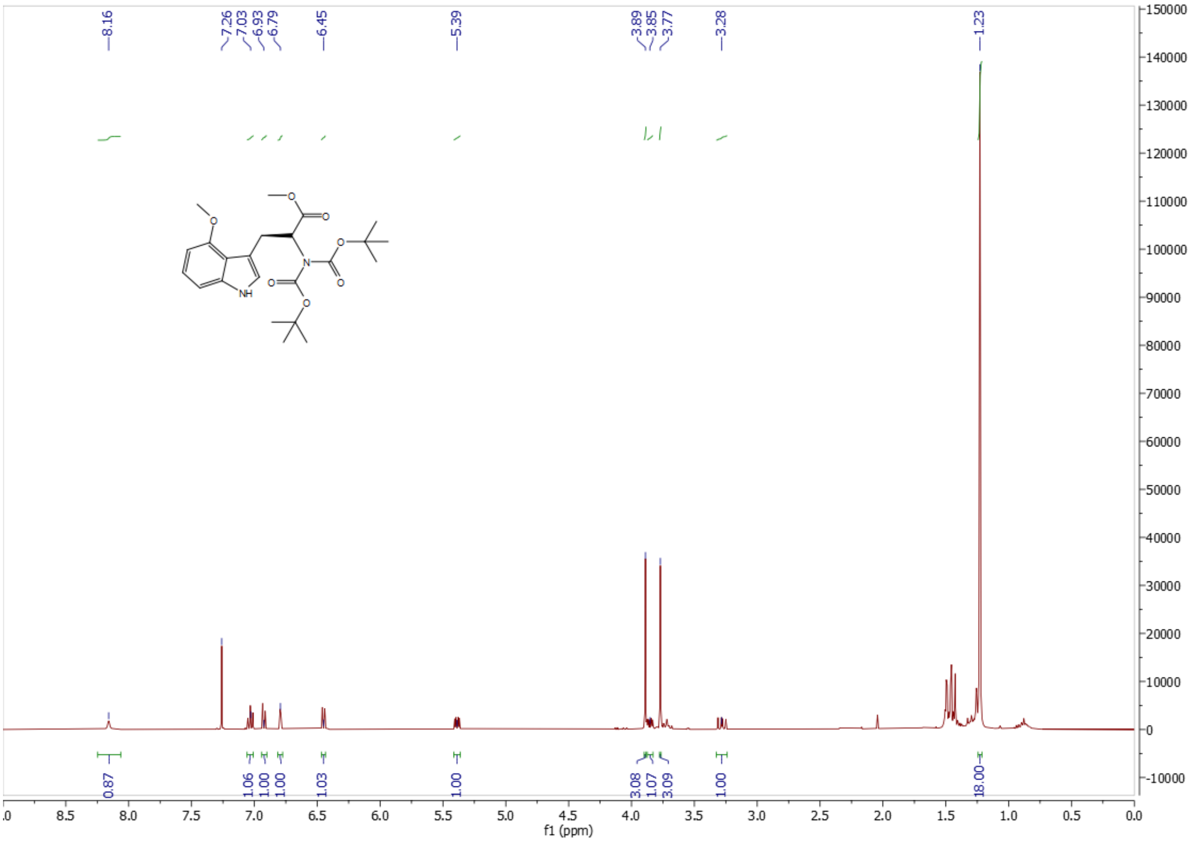

^{13}C NMR of **SI-24** in $CDCl_3$

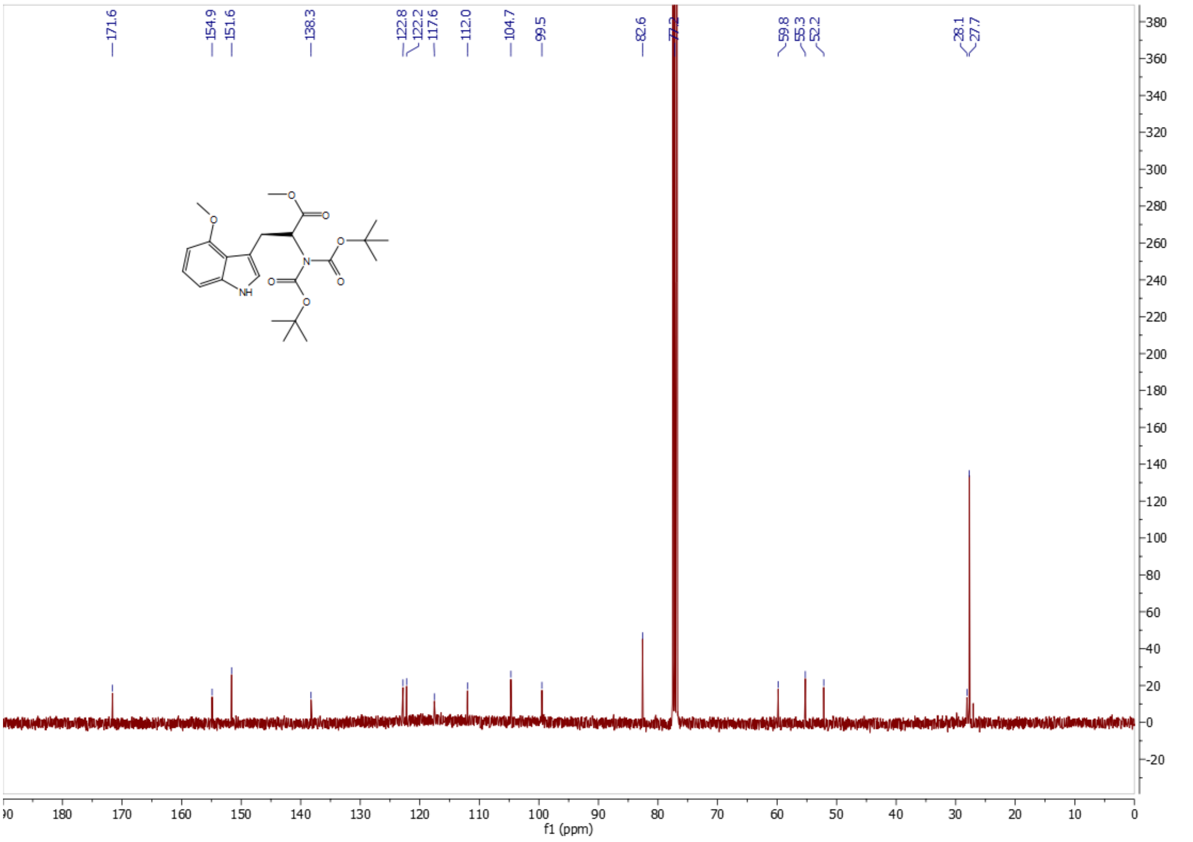

^1H NMR of **SI-25** in $CDCl_3$

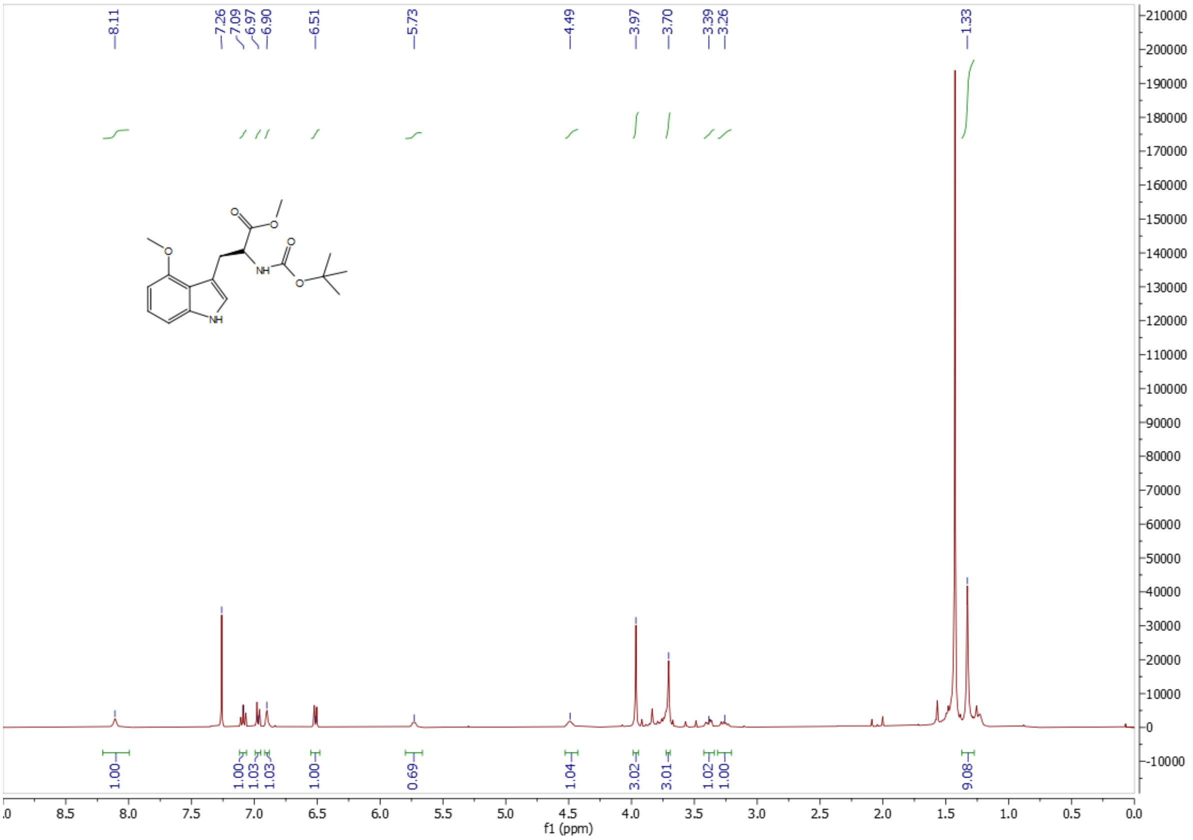

^{13}C NMR of **SI-25** in $CDCl_3$

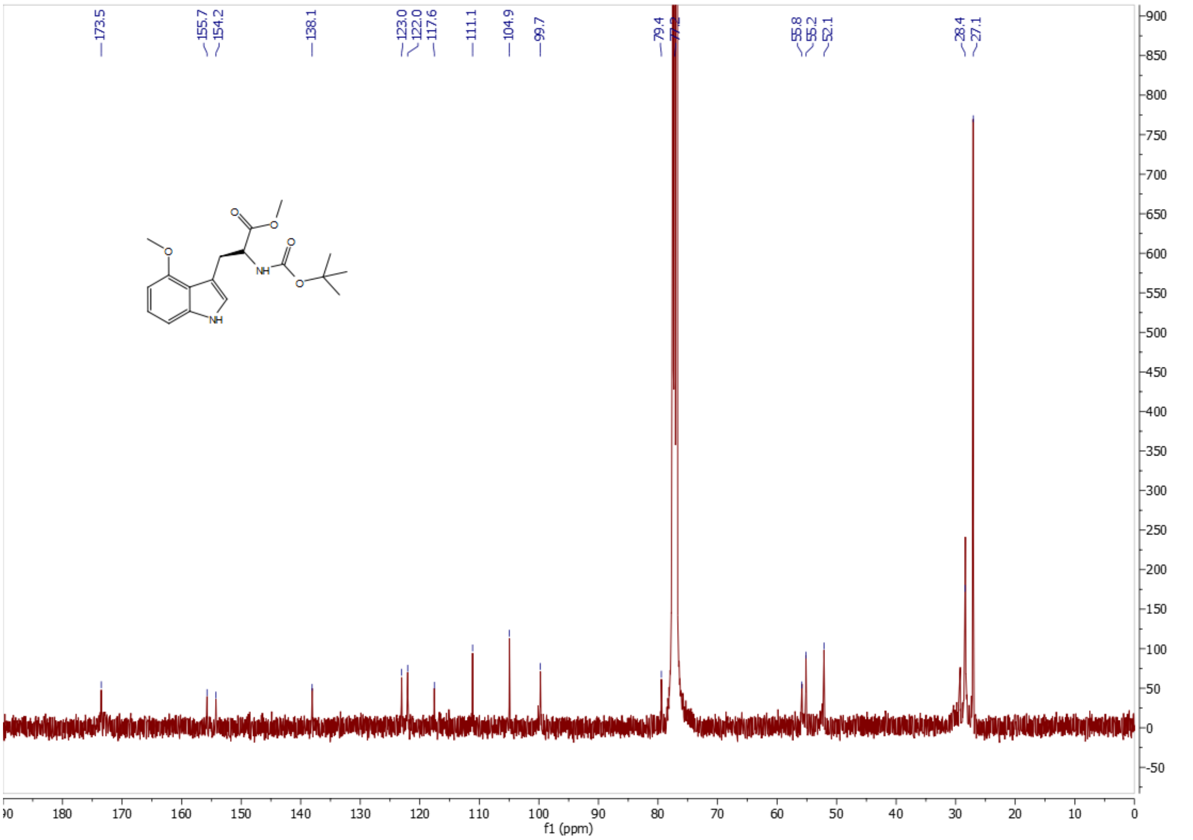

^{1}H NMR of **8** in $CDCl_3$

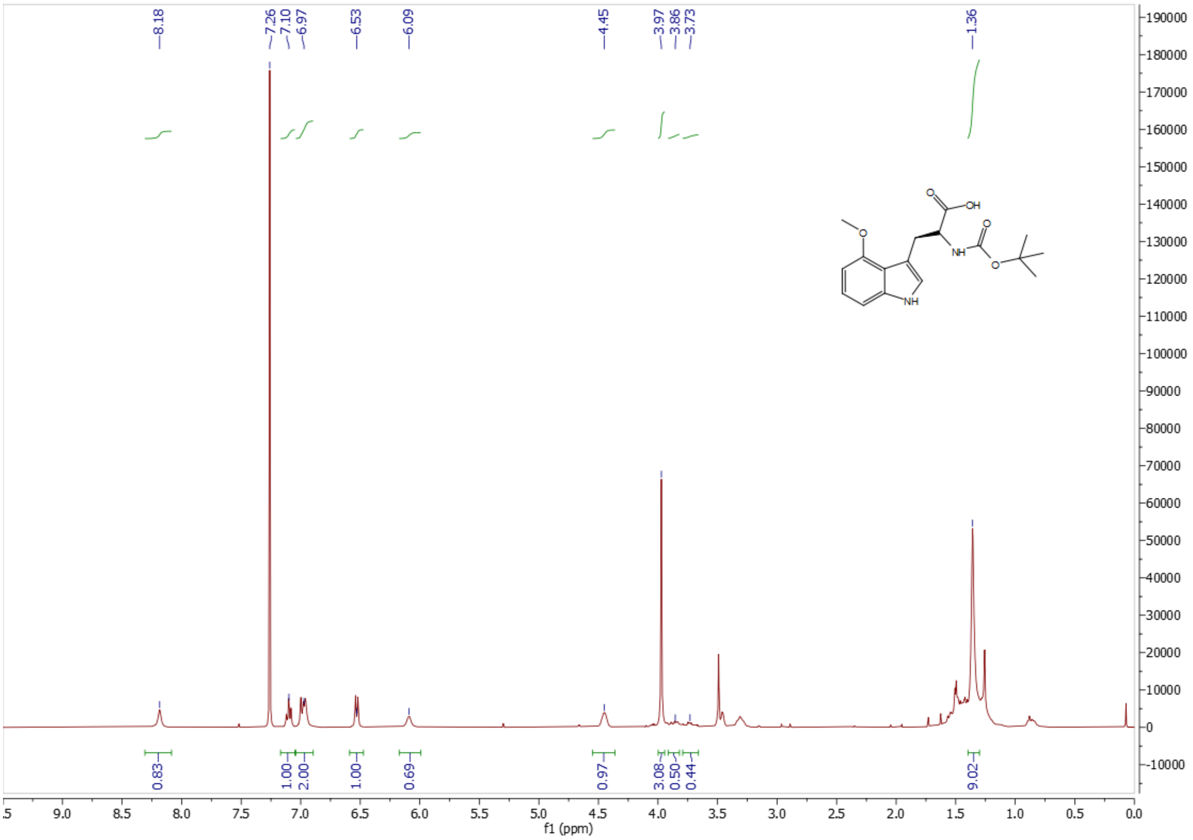

^{13}C NMR of **8** in $CDCl_3$

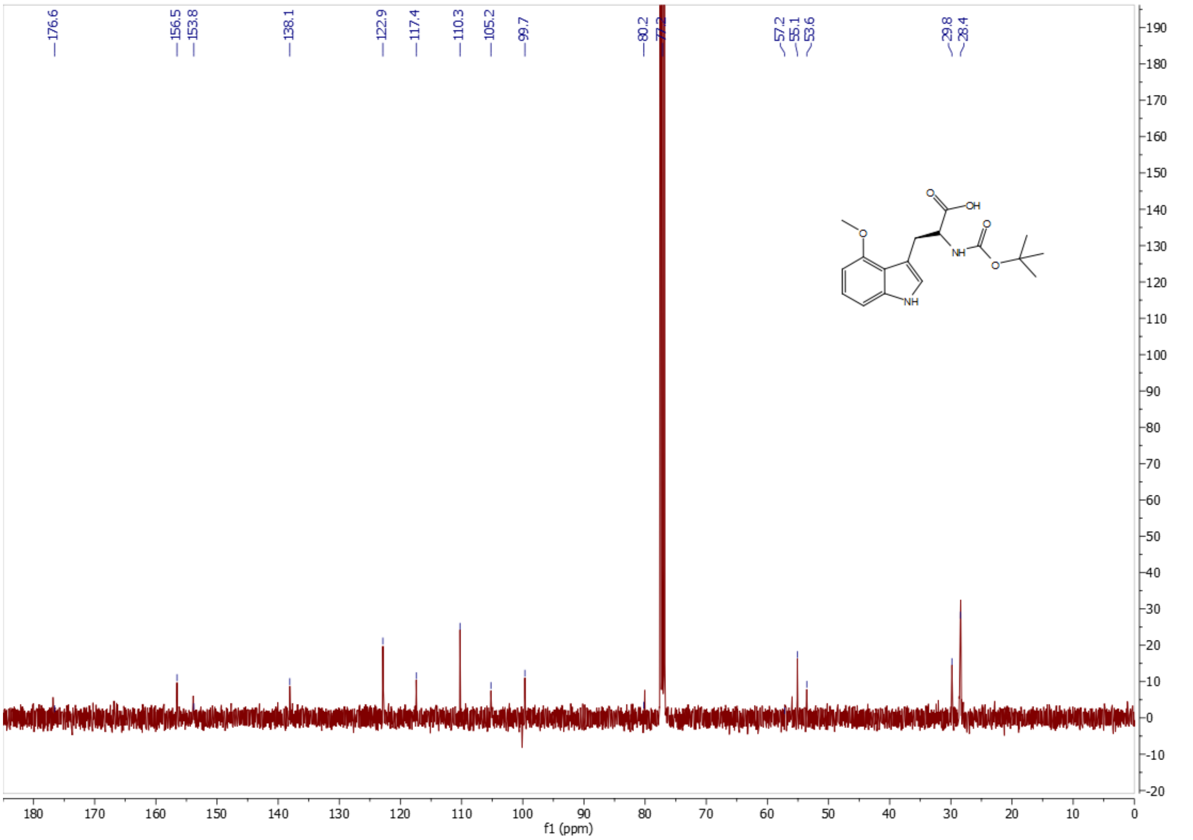

^{1}H NMR of **9** in $CDCl_3$

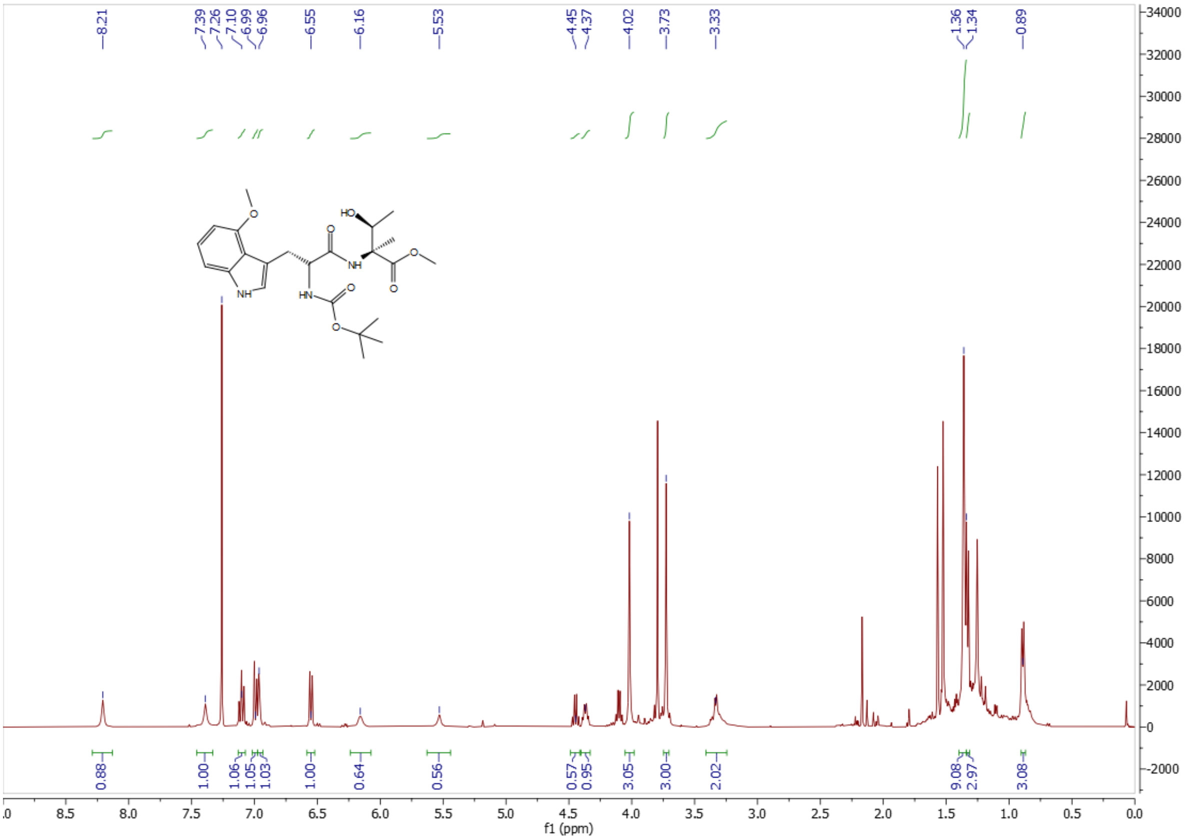

^{13}C NMR of **9** in $CDCl_3$

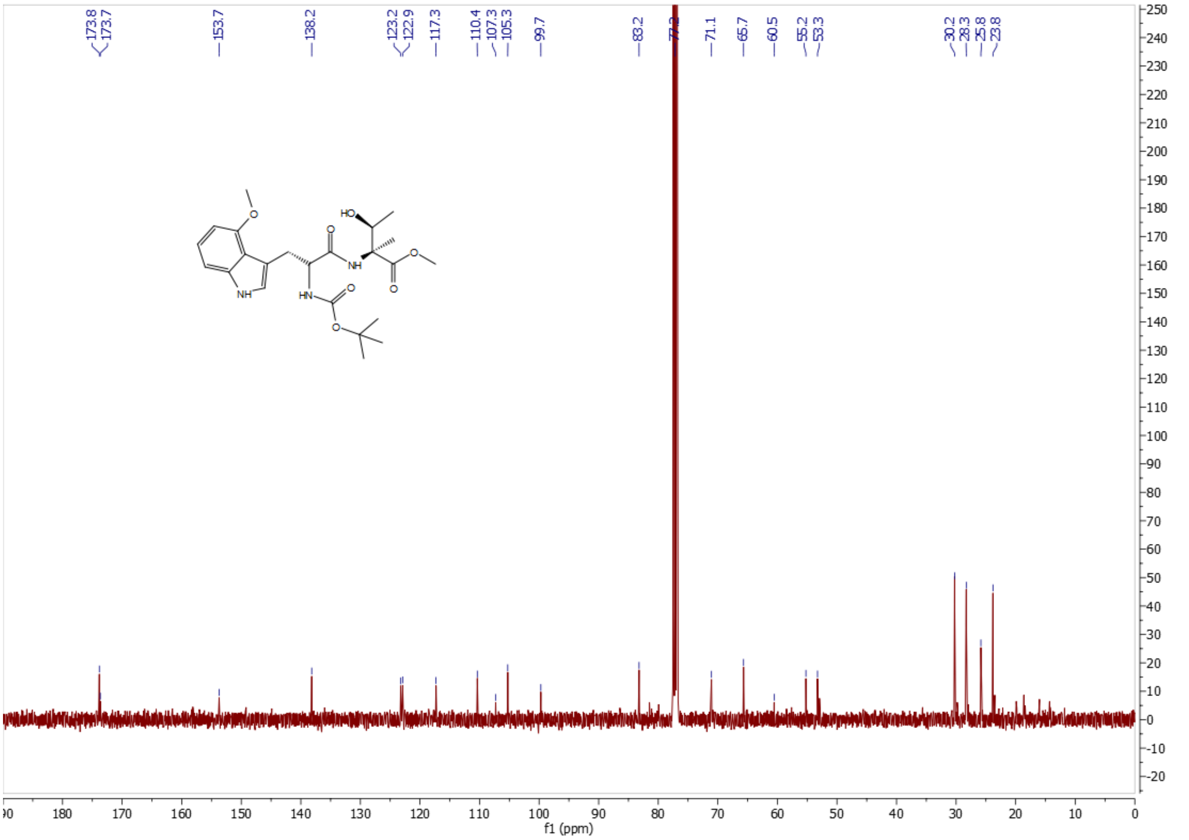

^{1}H NMR of **SI-26** in $CDCl_3$

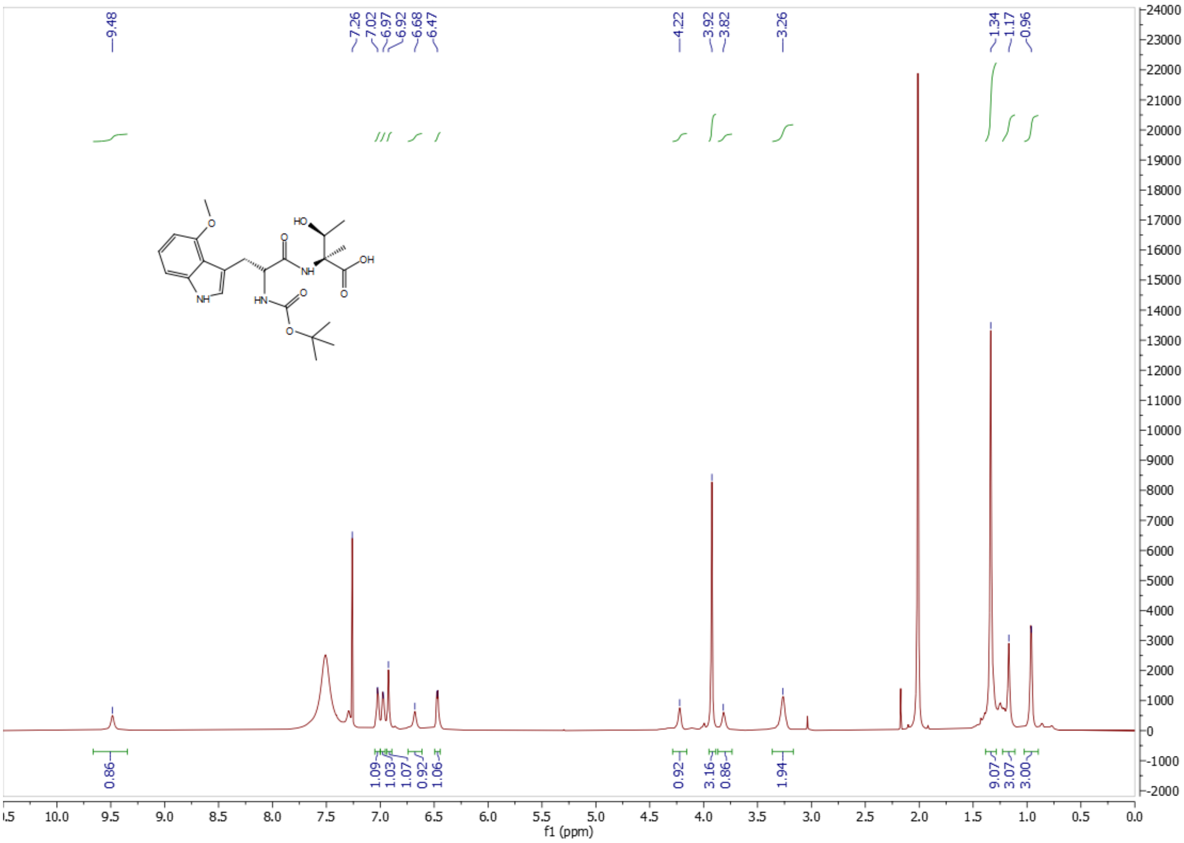

^{13}C NMR of **SI-26** in $CDCl_3$

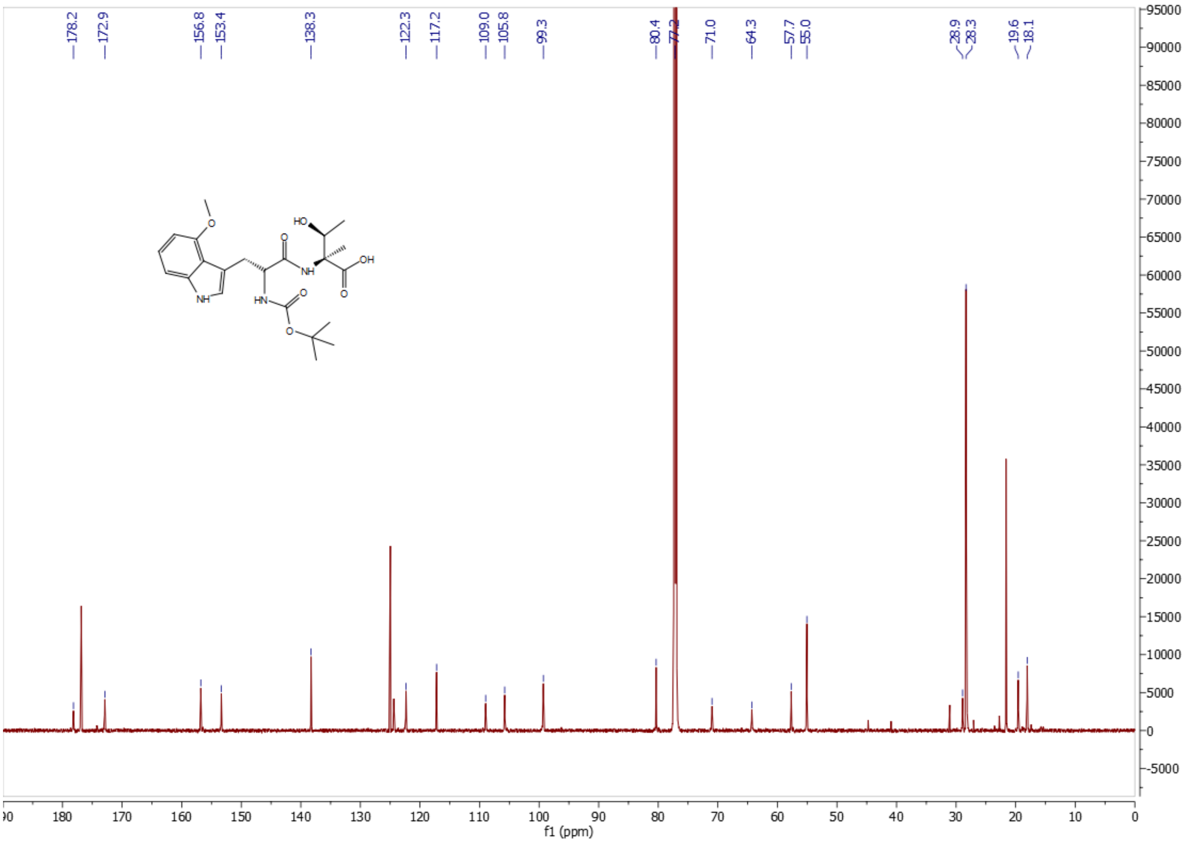

^{1}H NMR of **SI-27** in $CDCl_3$

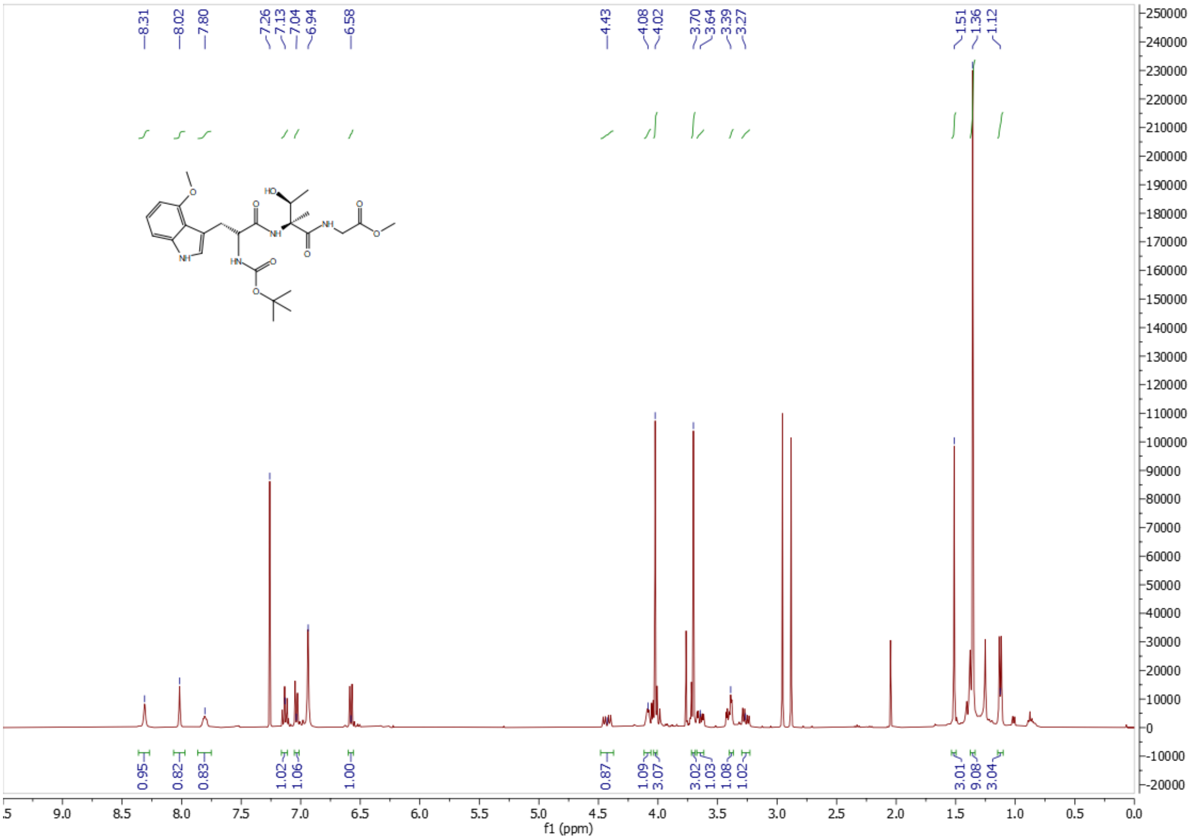

^{13}C NMR of **SI-27** in $CDCl_3$

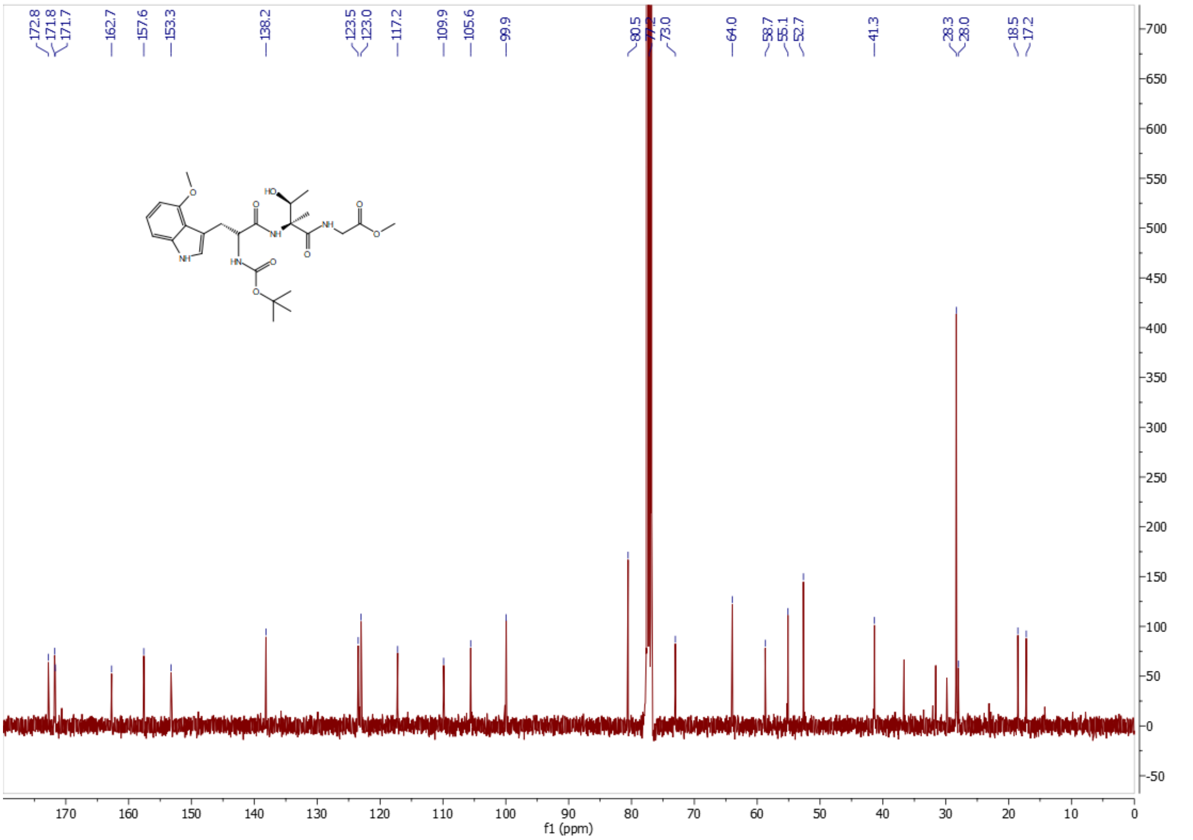

^{1}H NMR of **10** in MeOD

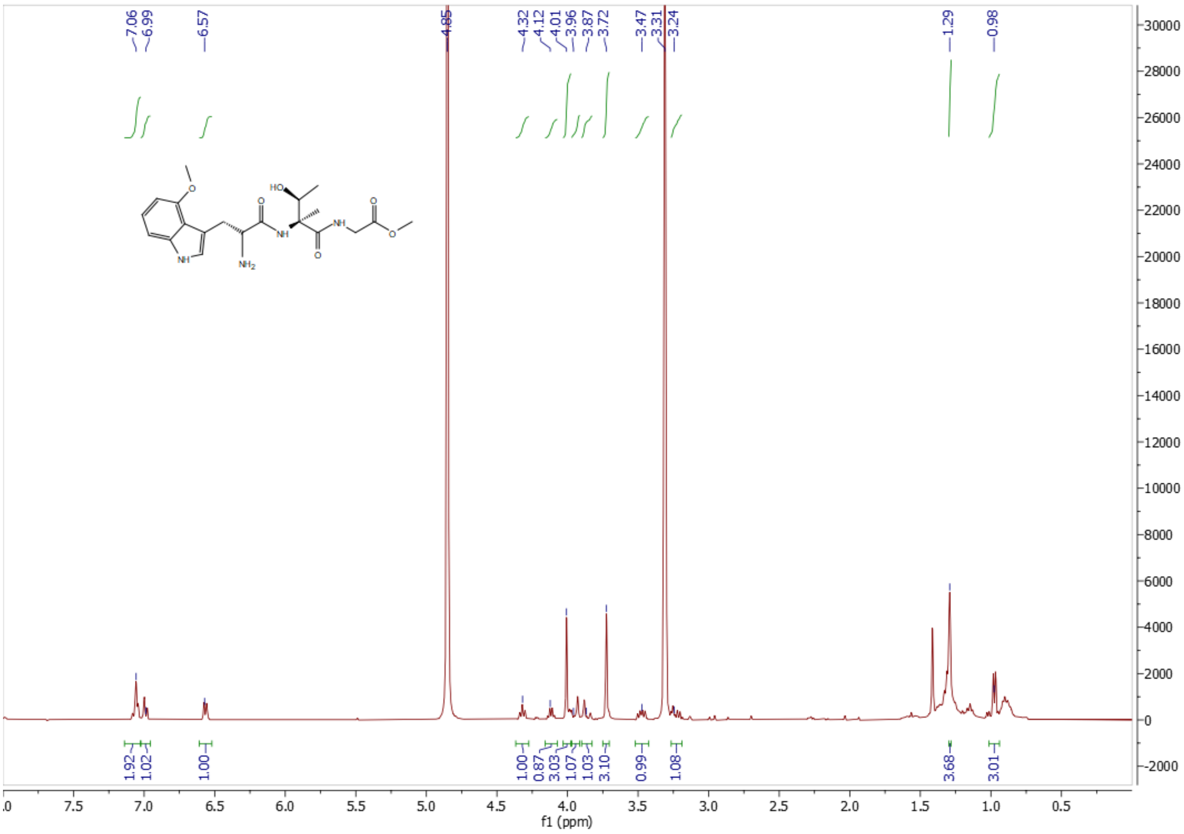

^{13}C NMR of **10** in MeOD

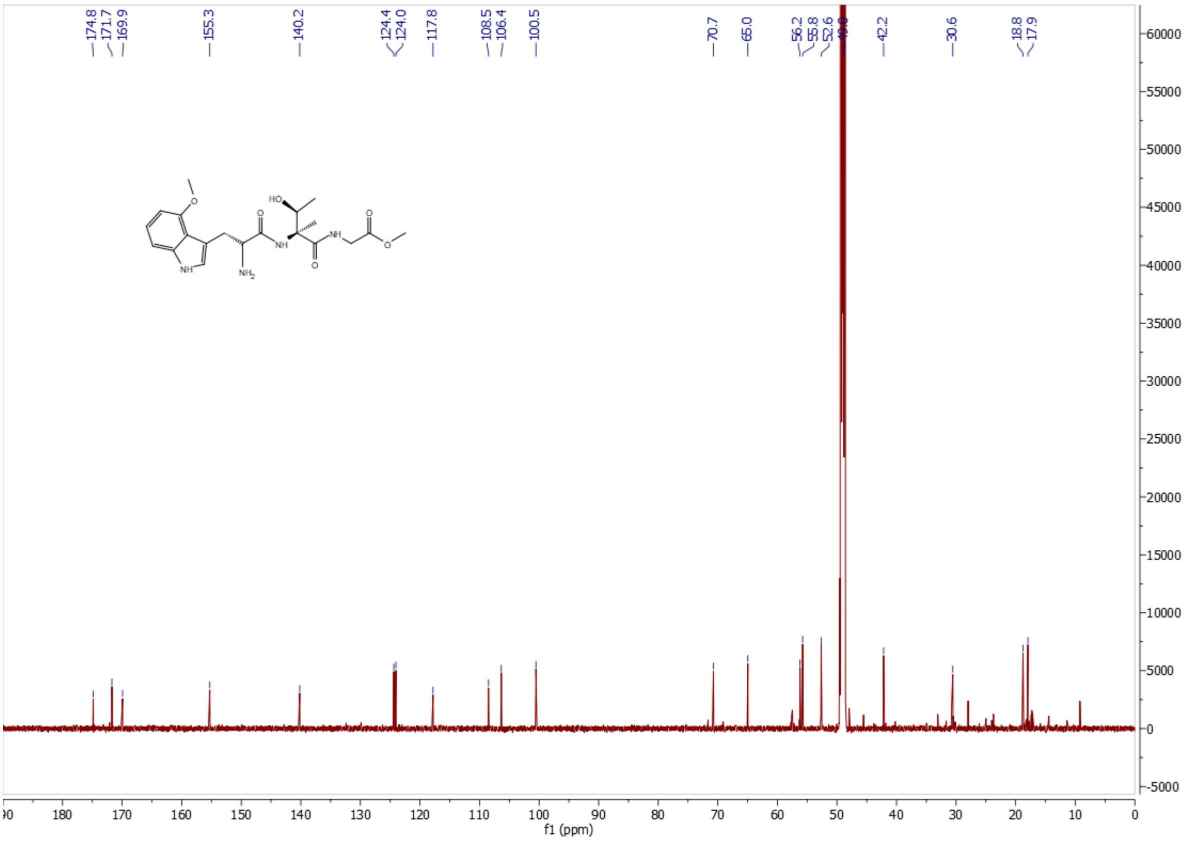

^{1}H NMR of **SI-28** in $CDCl_3$

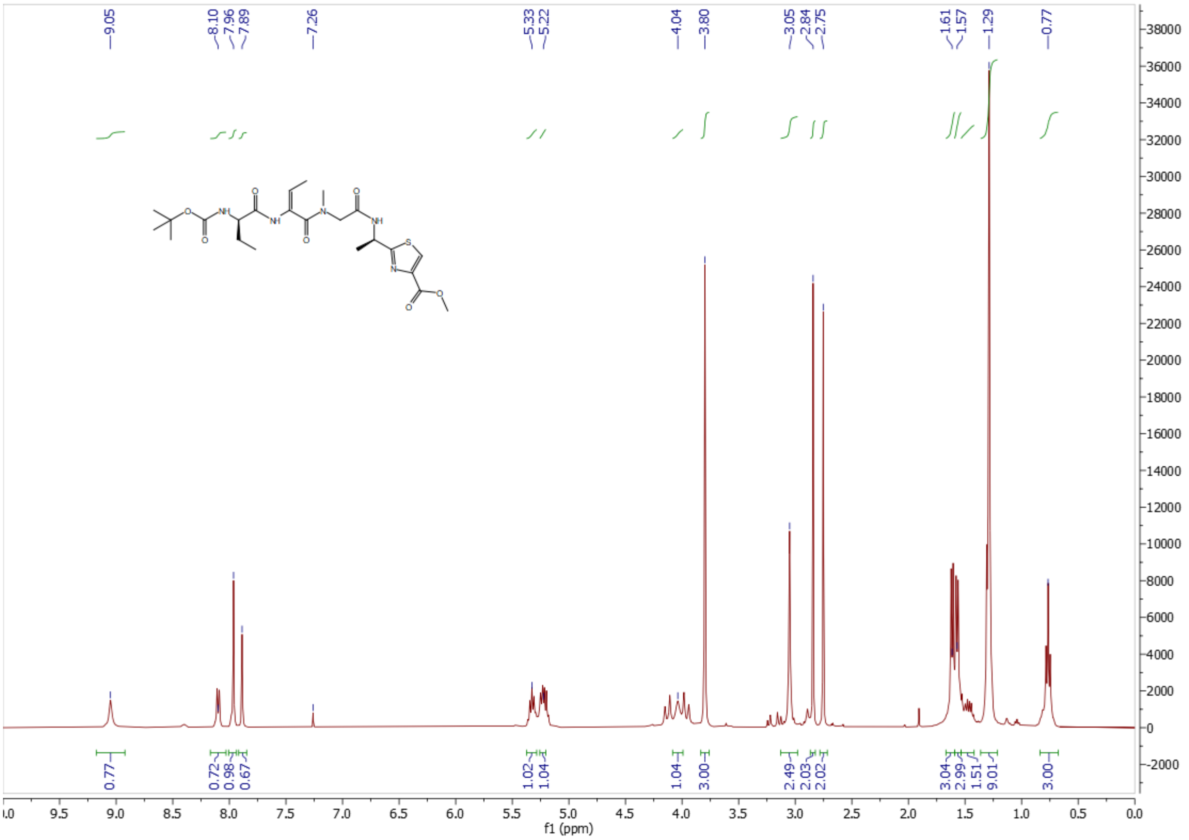

^{13}C NMR of **SI-28** in $CDCl_3$

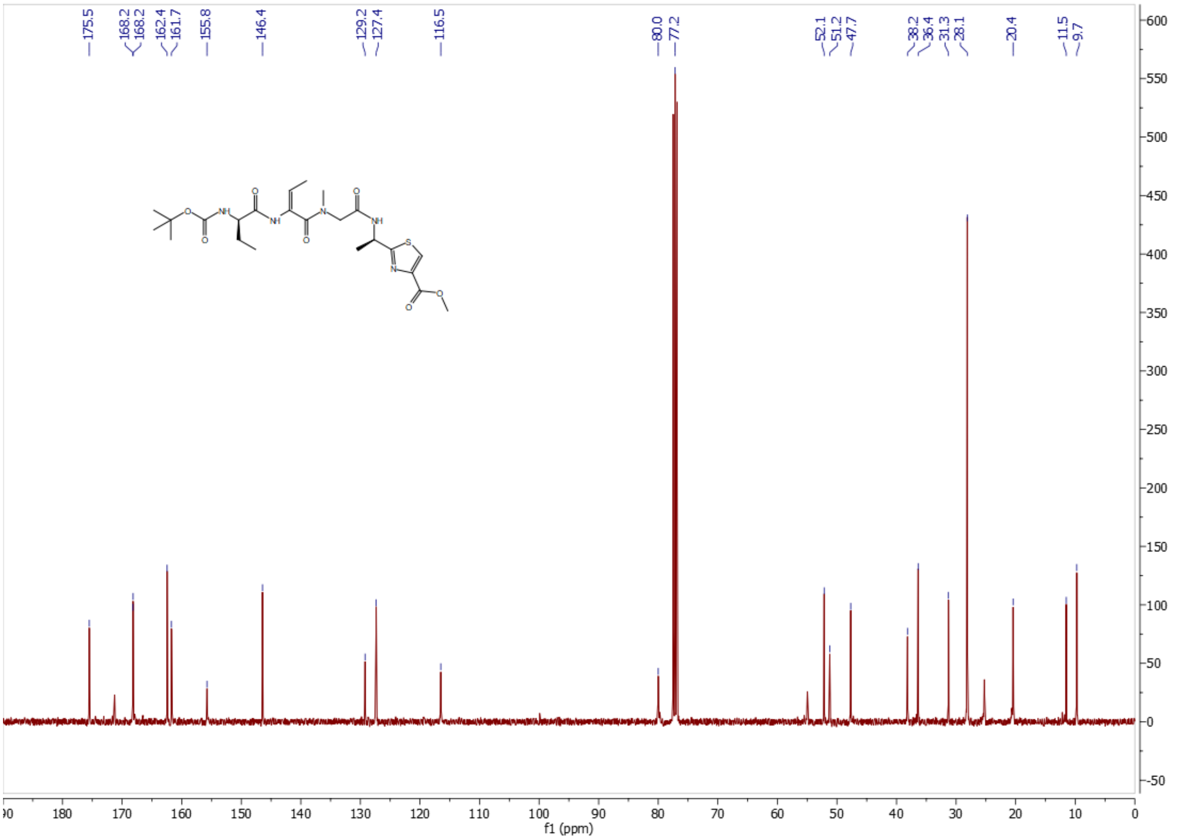

^{1}H NMR of **11** in MeOD

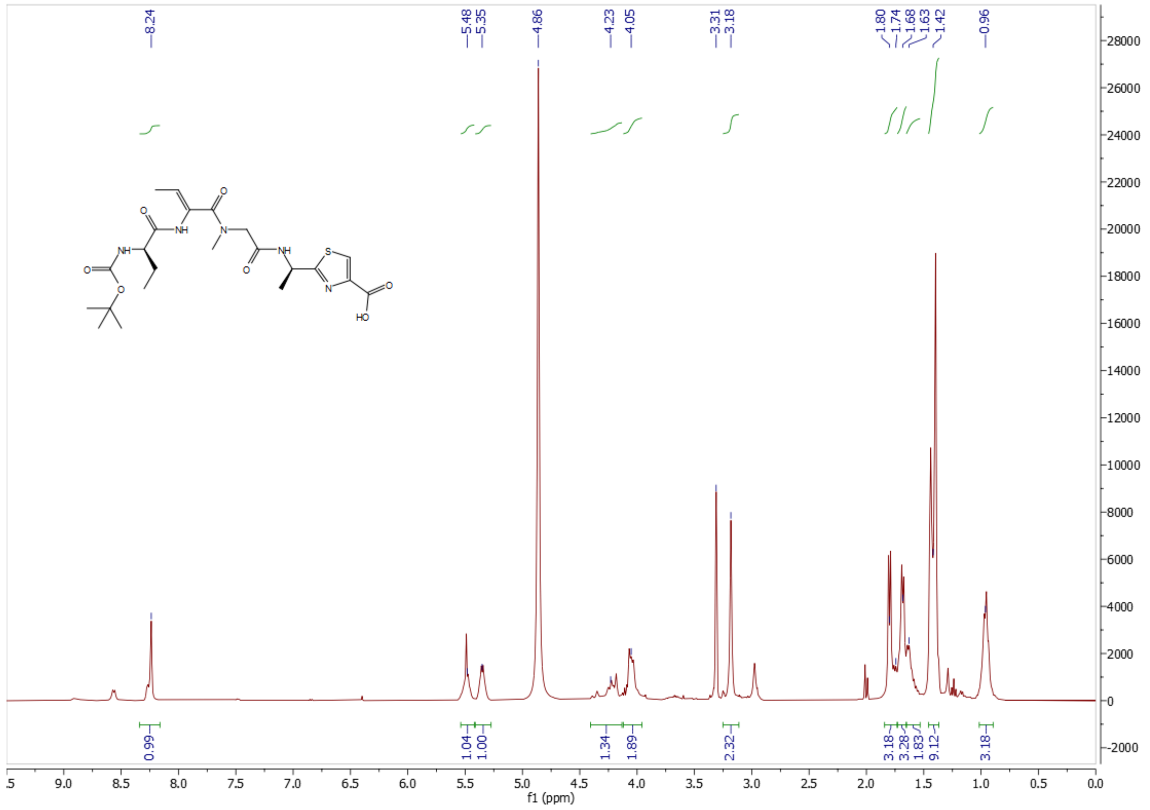

^{13}C NMR of **11** in MeOD

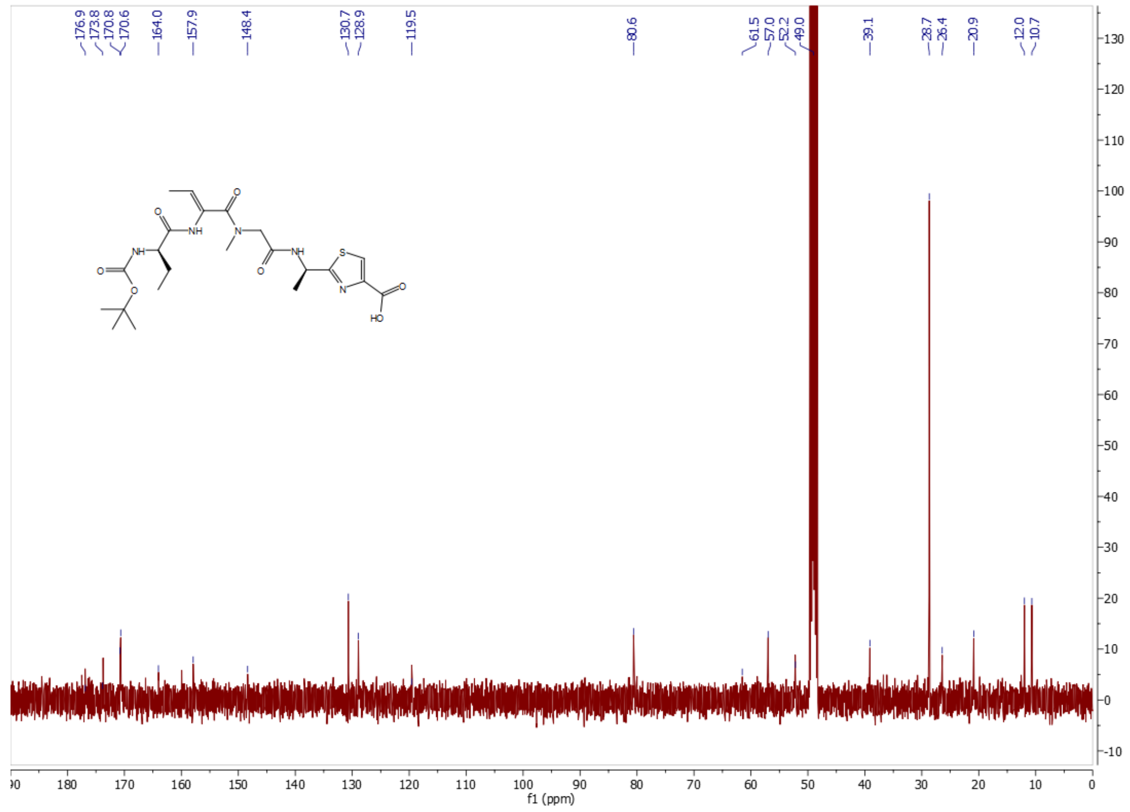

^{1}H NMR of **12** in MeOD

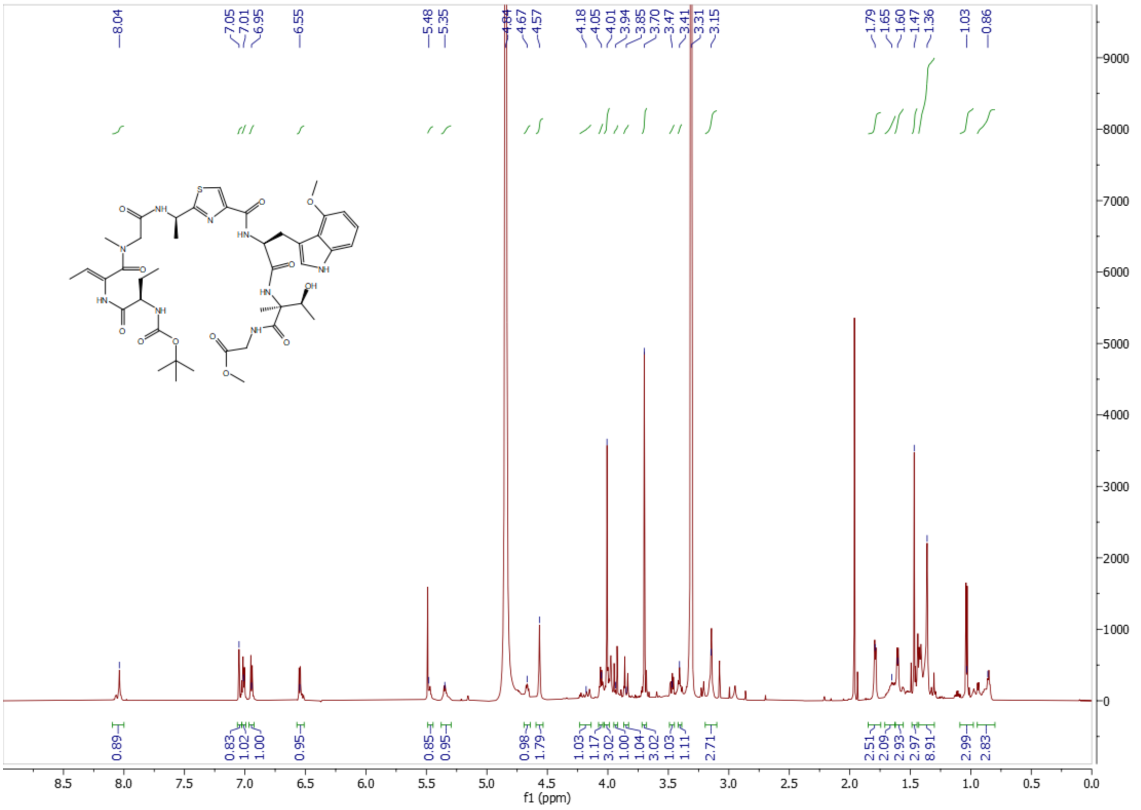

^{13}C NMR of **12** in $CDCl_3$

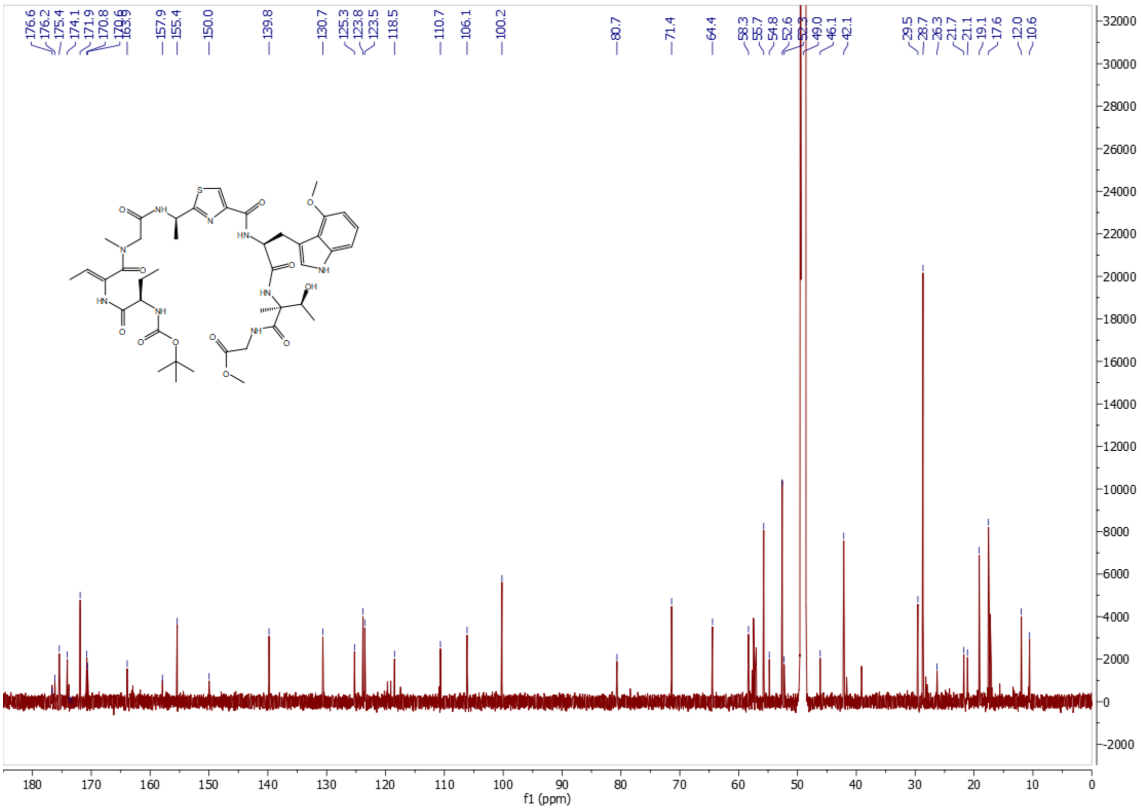

^{1}H NMR of **13** in $CDCl_3$

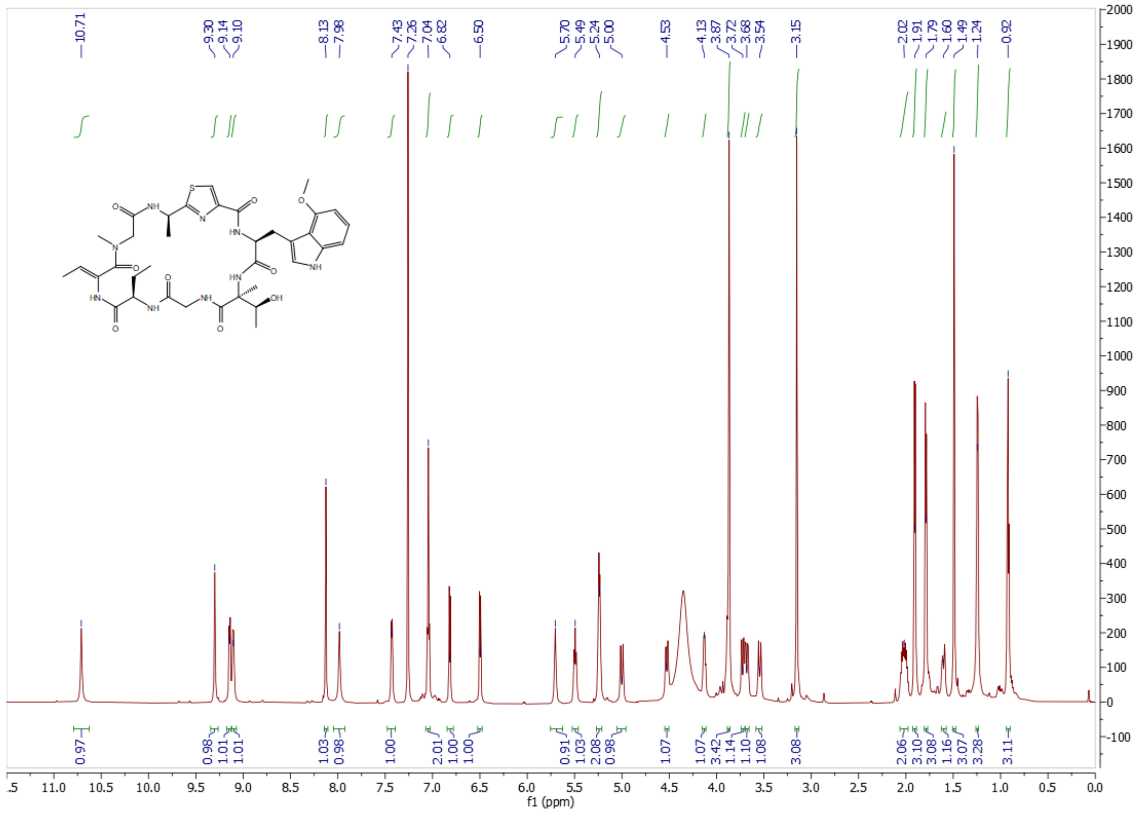

^{13}C NMR of **13** in $CDCl_3$

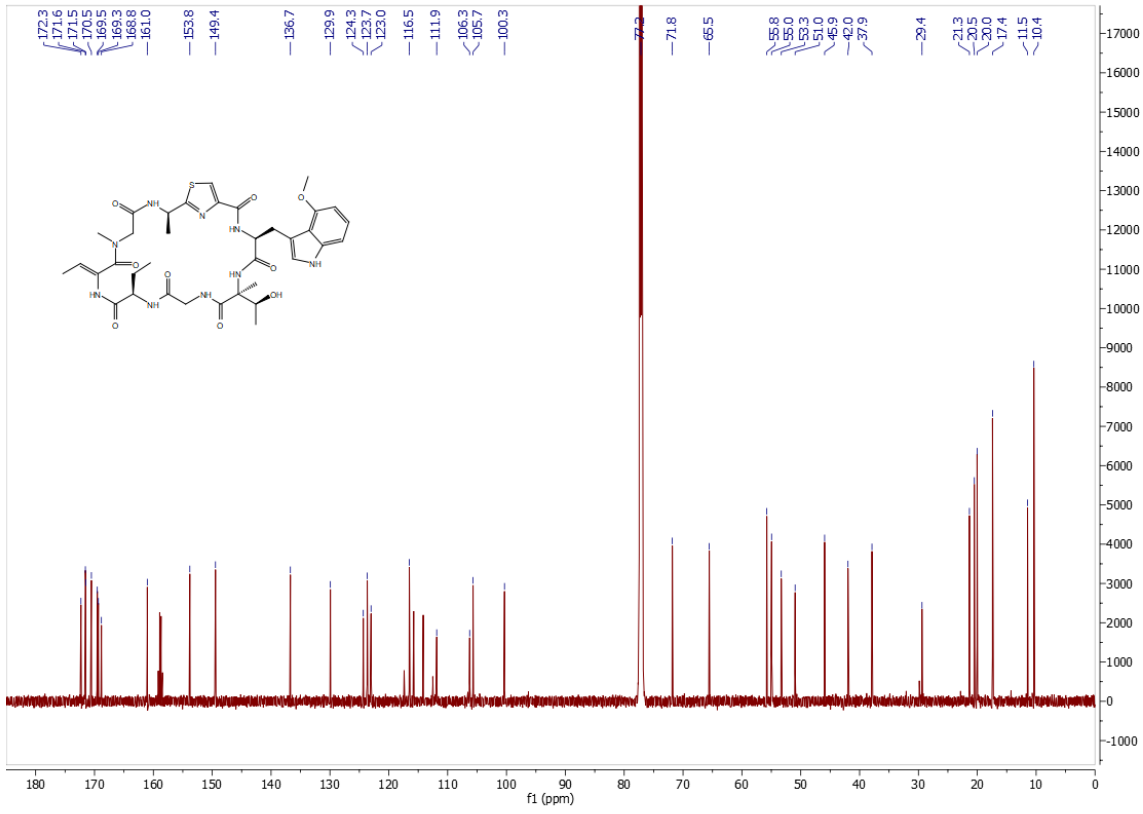

^{1}H NMR of **1** in $CDCl_3$

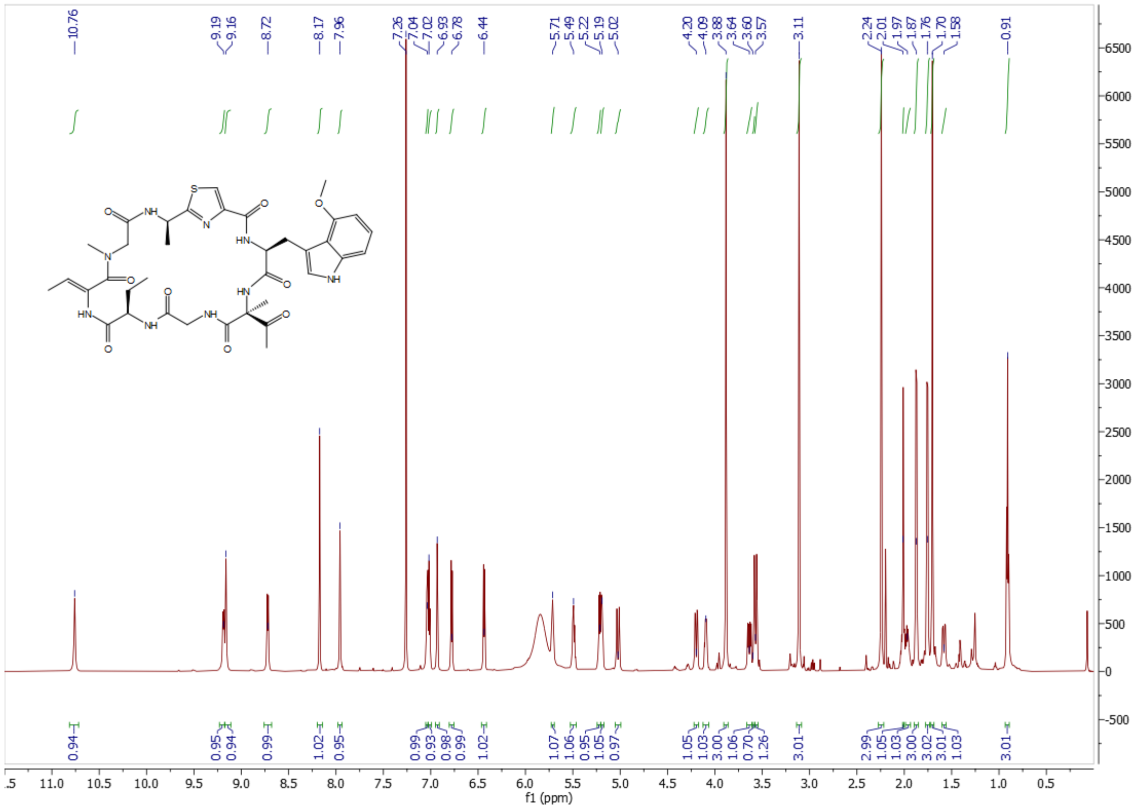

^{13}C NMR of **1** in $CDCl_3$

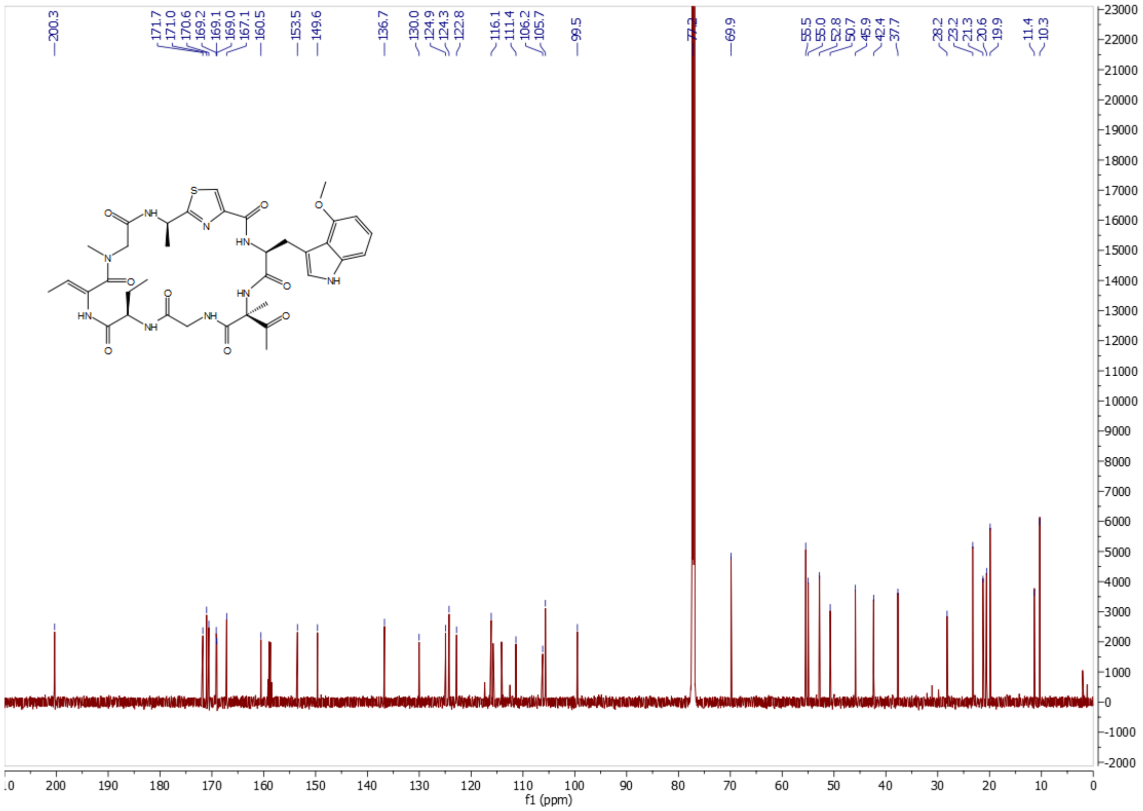

^{1}H NMR of **14** in $CDCl_3$

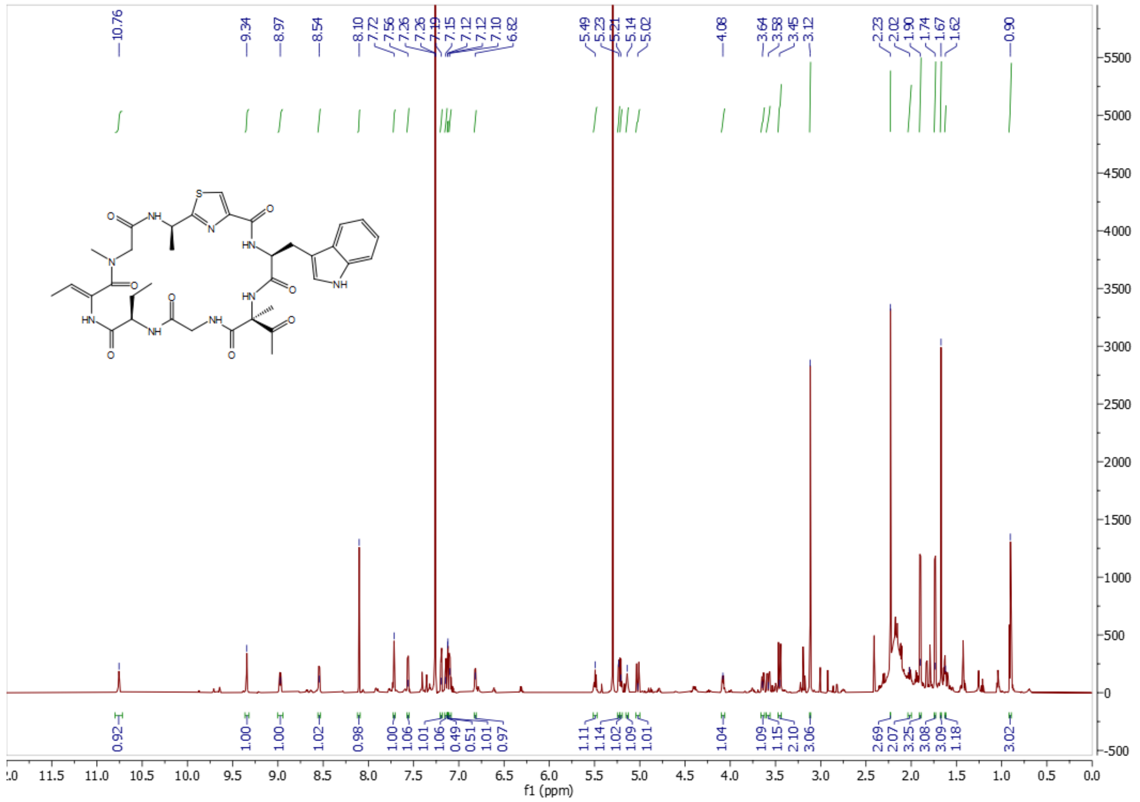

^{13}C NMR of **14** in $CDCl_3$

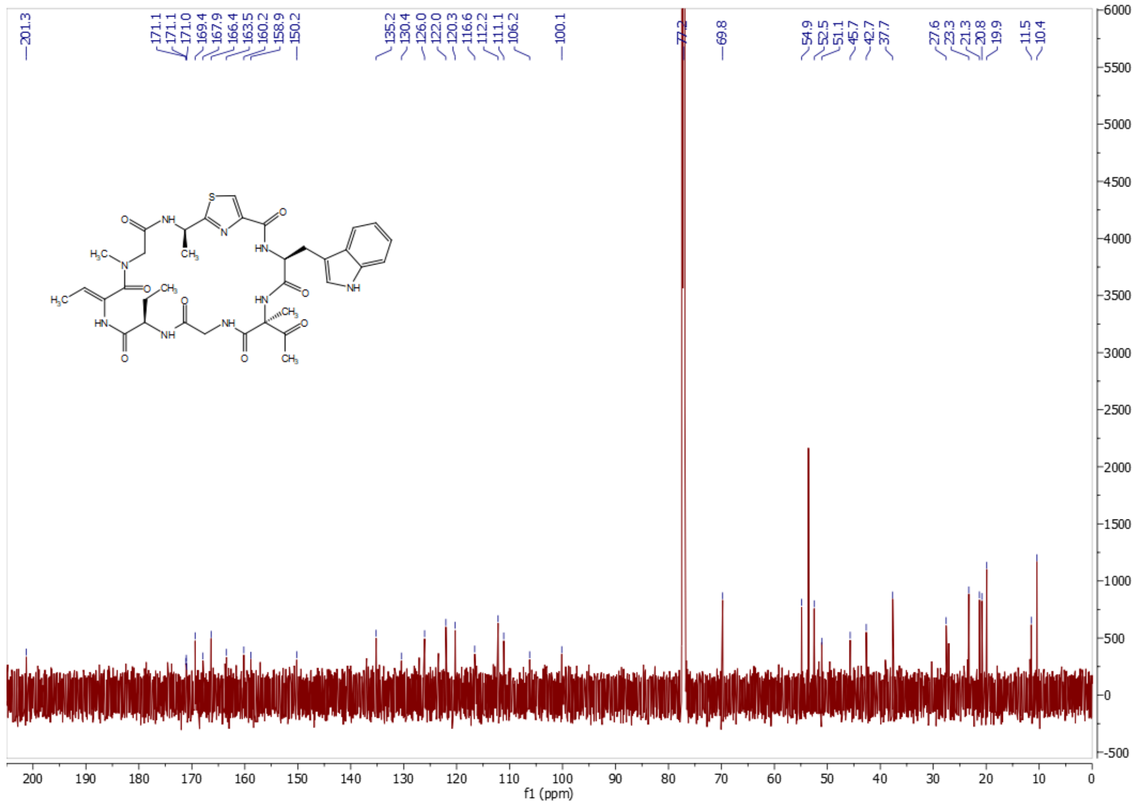

Supplement: Supplementary file 1 — Supplementary [file CHEM-26-8524-s001.zip › SuppInfos.pdf]
