# Supplementary material for: Zelkovamycin is an OXPHOS Inhibitory Member of the Argyrin Natural Product Family
Source: Chemistry. 2020 Jun 17;26(39):8524–31. doi: 10.1002/chem.202001577 (PMC7383741; doi:10.1002/chem.202001577)
Supplement: Supplementary file 1 — Supplementary [file CHEM-26-8524-s001.zip › Supporting File 1.pdf]

mMass Report: *MSn spectrum [568]*

**Date**	Fri Jul 19 06:14:04 2019	**Scan Number**	568
**Operator**		**Retention Time**	502.2816
**Contact**		**MS Level**	2
**Institution**		**Precursor m/z**	780.31
**Instrument**		**Polarity**	positive
		**Spectrum Points**	0
		**Peak List**	373

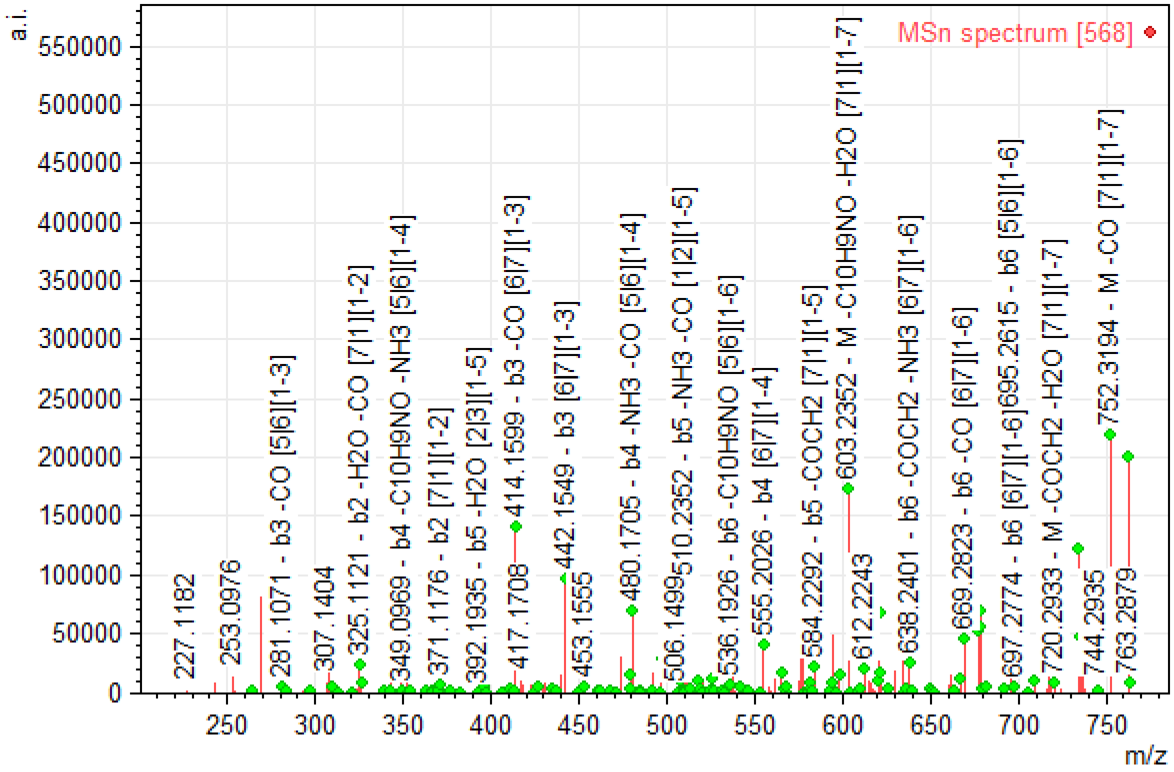

Sequence - *Zelkovamycin*

Accession	Length	Mo. Mass	Av. Mass	Coverage	Matched Int.
	7 (Cyclic)	779.3061	779.8652	100.0 %	66.2 %

`Ala-Thz | (OMe)Trp | 2Me-dhThr | Gly | Abu | Dhb | Sar`

Meas. m/z	Calc. m/z	δ (Da)	δ (ppm)	Rel. Int. (%)	z	Annotation	Formula
264.0803	264.0801	0.0001	0.5	0.57	1	b3 -NH3 -CO [5|6][1-3]	C12H13N3O2S
281.1071	281.1067	0.0004	1.4	2.19	1	b3 -CO [5|6][1-3]	C12H16N4O2S
283.0864	283.0859	0.0004	1.5	0.70	1	b3 -C10H9NO -COCH2 [7|1][1-3]	C11H14N4O3S
283.0864	283.0859	0.0004	1.5	0.70	1	b3 -C10H9NO [6|7][1-3]	C11H14N4O3S
297.1561	297.1557	0.0004	1.2	0.66	1	b4 -COCH2 [2|3][1-4]	C13H20N4O4
297.1561	297.1557	0.0004	1.2	0.66	1	b4 [3|4][1-4]	C13H20N4O4
309.1020	309.1016	0.0004	1.2	2.45	1	b3 [5|6][1-3]	C13H16N4O3S
313.1187	313.1183	0.0004	1.2	0.28	1	b2 -NH3 [1|2][1-2]	C17H16N2O4

Meas. m/z	Calc. m/z	δ (Da)	δ (ppm)	Rel. Int. (%)	z	Annotation	Formula
321.1562	321.1557	0.0004	1.3	0.27	1	b4 -H2O [2|3][1-4]	C15H20N4O4
325.1121	325.1118	0.0004	1.1	10.51	1	b2 -H2O -CO [7|1][1-2]	C17H16N4OS
326.0960	326.0958	0.0003	0.8	3.70	1	b2 -NH3 -CO [7|1][1-2]	C17H15N3O2S
339.1667	339.1663	0.0004	1.1	0.64	1	b4 [2|3][1-4]	C15H22N4O5
342.1450	342.1448	0.0001	0.4	1.08	1	b3 -NH3 -CO [1|2][1-3]	C18H19N3O4
343.1227	343.1223	0.0004	1.1	7.08	1	b2 -CO [7|1][1-2]	C17H18N4O2S
349.0969	349.0965	0.0004	1.0	0.53	1	b4 -C10H9NO -NH3 [5|6][1-4]	C15H16N4O4S
353.1071	353.1067	0.0005	1.3	0.14	1	b2 -H2O [7|1][1-2]	C18H16N4O2S
354.0909	354.0907	0.0002	0.5	0.91	1	b2 -NH3 [7|1][1-2]	C18H15N3O3S
363.1667	363.1663	0.0004	1.1	0.43	1	b3 +H2O -COCH2 [1|2][1-3]	C17H22N4O5
366.1235	366.1231	0.0005	1.3	0.39	1	b4 -C10H9NO [5|6][1-4]	C15H19N5O4S
366.1598	366.1594	0.0003	0.9	0.15	1	b4 -CO [4|5][1-4]	C16H23N5O3S
370.1400	370.1397	0.0003	0.8	0.86	1	b3 -NH3 [1|2][1-3]	C19H19N3O5
371.1176	371.1172	0.0003	0.9	3.14	1	b2 [7|1][1-2]	C18H18N4O3S
377.1283	377.1278	0.0005	1.2	0.57	1	b4 -NH3 [4|5][1-4]	C17H20N4O4S
382.2089	382.2085	0.0004	1.1	0.25	1	b5 -CO [2|3][1-5]	C17H27N5O5
392.1935	392.1928	0.0007	1.7	0.26	1	b5 -H2O [2|3][1-5]	C18H25N5O5
396.1341	396.1336	0.0005	1.2	2.09	1	b4 -C10H9NO [6|7][1-4]	C16H21N5O5S
396.1493	396.1489	0.0004	1.1	36.12	1	b3 -H2O -CO [6|7][1-3]	C20H21N5O2S
397.1335	397.1329	0.0007	1.7	0.25	1	b3 -NH3 -CO [6|7][1-3]	C20H20N4O3S
406.1544	406.1544	0.0001	0.2	0.25	1	b5 -NH3 -CO [3|4][1-5]	C18H23N5O4S
410.2037	410.2034	0.0003	0.7	1.47	1	b5 [2|3][1-5]	C18H27N5O6
413.1826	413.1819	0.0006	1.5	0.61	1	b4 -COCH2 -NH3 [1|2][1-4]	C21H24N4O5
414.1599	414.1594	0.0005	1.2	60.14	1	b3 -CO -COCH2 [7|1][1-3]	C20H23N5O3S
414.1599	414.1594	0.0005	1.2	60.14	1	b3 -CO [6|7][1-3]	C20H23N5O3S
424.1445	424.1438	0.0007	1.6	0.35	1	b3 -COCH2 -H2O [7|1][1-3]	C21H21N5O3S
424.1445	424.1438	0.0007	1.6	0.35	1	b3 -H2O [6|7][1-3]	C21H21N5O3S
427.1980	427.1976	0.0004	1.0	2.25	1	b4 -NH3 -CO [1|2][1-4]	C22H26N4O5
435.1448	435.1445	0.0003	0.7	1.28	1	b5 -C10H9NO -H2O [6|7][1-5]	C18H22N6O5S
437.1603	437.1602	0.0002	0.4	0.95	1	b5 -C10H9NO -COCH2 [5|6][1-5]	C18H24N6O5S
442.1549	442.1544	0.0005	1.2	41.13	1	b3 -COCH2 [7|1][1-3]	C21H23N5O4S
442.1549	442.1544	0.0005	1.2	41.13	1	b3 [6|7][1-3]	C21H23N5O4S
449.1596	449.1602	-0.0006	-1.2	0.13	1	b5 -C10H9NO -H2O [7|1][1-5]	C19H24N6O5S
453.1555	453.1551	0.0005	1.0	2.05	1	b5 -C10H9NO [6|7][1-5]	C18H24N6O6S
461.1608	461.1602	0.0006	1.3	0.47	1	b5 -C10H9NO -H2O [5|6][1-5]	C20H24N6O5S
462.1448	462.1442	0.0006	1.4	1.03	1	b5 -C10H9NO -NH3 [5|6][1-5]	C20H23N5O6S
467.1387	467.1384	0.0003	0.7	0.39	1	b3 -NH3 [7|1][1-3]	C23H22N4O5S
471.1803	471.1809	-0.0006	-1.2	0.14	1	b4 -CO -COCH2 [7|1][1-4]	C22H26N6O4S
479.1714	479.1707	0.0007	1.4	1.17	1	b5 -C10H9NO [5|6][1-5]	C20H26N6O6S
479.1865	479.1860	0.0005	1.0	6.68	1	b4 -H2O -CO [5|6][1-4]	C24H26N6O3S
480.1705	480.1700	0.0005	1.1	29.46	1	b4 -NH3 -CO [5|6][1-4]	C24H25N5O4S
484.1653	484.1649	0.0004	0.8	0.77	1	b3 [7|1][1-3]	C23H25N5O5S
485.1968	485.1966	0.0003	0.6	0.14	1	b4 -CO -COCH2 [6|7][1-4]	C23H28N6O4S
490.1873	490.1867	0.0005	1.1	0.28	1	b6 -C10H9NO -H2O [3|4][1-6]	C21H27N7O5S
491.1711	491.1707	0.0004	0.7	0.76	1	b6 -C10H9NO -NH3 [3|4][1-6]	C21H26N6O6S
496.1660	496.1649	0.0011	2.3	0.14	1	b4 -COCH2 -NH3 [6|7][1-4]	C24H25N5O5S
496.1660	496.1649	0.0011	2.3	0.14	1	b4 -NH3 -CO [7|1][1-4]	C24H25N5O5S
496.2204	496.2191	0.0013	2.7	0.28	1	b5 -COCH2 -NH3 [1|2][1-5]	C25H29N5O6

Meas. m/z	Calc. m/z	δ (Da)	δ (ppm)	Rel. Int. (%)	z	Annotation	Formula
497.1971	497.1966	0.0006	1.2	12.27	1	b4 -CO [5|6][1-4]	C24H28N6O4S
506.1499	506.1493	0.0006	1.2	0.36	1	b4 -H2O -NH3 [7|1][1-4]	C25H23N5O5S
507.1815	507.1809	0.0006	1.2	4.67	1	b4 -H2O [5|6][1-4]	C25H26N6O4S
508.1655	508.1649	0.0006	1.1	15.97	1	b4 -NH3 [5|6][1-4]	C25H25N5O5S
509.1969	509.1966	0.0003	0.6	0.81	1	b4 -H2O -CO [6|7][1-4]	C25H28N6O4S
510.1814	510.1806	0.0009	1.7	0.32	1	b4 -NH3 -CO [6|7][1-4]	C25H27N5O5S
510.2352	510.2347	0.0005	1.0	39.11	1	b5 -NH3 -CO [1|2][1-5]	C26H31N5O6
513.1923	513.1915	0.0009	1.7	1.47	1	b4 -COCH2 [6|7][1-4]	C24H28N6O5S
513.1923	513.1915	0.0009	1.7	1.47	1	b4 -CO [7|1][1-4]	C24H28N6O5S
518.1821	518.1816	0.0004	0.8	4.02	1	b6 -C10H9NO -H2O [5|6][1-6]	C22H27N7O6S
519.1663	519.1656	0.0006	1.2	1.02	1	b6 -C10H9NO -NH3 [5|6][1-6]	C22H26N6O7S
519.2025	519.2020	0.0005	0.9	1.42	1	b6 -NH3 -CO [2|3][1-6]	C23H30N6O6S
520.2206	520.2191	0.0016	3.0	0.17	1	b5 -H2O -NH3 [1|2][1-5]	C27H29N5O6
524.1603	524.1598	0.0005	1.0	1.01	1	b4 -NH3 [7|1][1-4]	C25H25N5O6S
525.1921	525.1915	0.0006	1.2	4.79	1	b4 [5|6][1-4]	C25H28N6O5S
527.2077	527.2071	0.0006	1.1	0.49	1	b4 -CO [6|7][1-4]	C25H30N6O5S
531.2034	531.2020	0.0013	2.5	0.90	1	b4 +H2O -COCH2 [6|7][1-4]	C24H30N6O6S
531.2034	531.2020	0.0013	2.5	0.90	1	b4 +H2O -CO [7|1][1-4]	C24H30N6O6S
532.1981	532.1973	0.0008	1.5	0.14	1	b6 -C10H9NO -H2O [7|1][1-6]	C23H29N7O6S
536.1926	536.1922	0.0004	0.7	2.73	1	b6 -C10H9NO [5|6][1-6]	C22H29N7O7S
541.1871	541.1864	0.0008	1.4	2.38	1	b4 [7|1][1-4]	C25H28N6O6S
546.2136	546.2129	0.0006	1.2	0.59	1	b6 -H2O [2|3][1-6]	C24H31N7O6S
546.2136	546.2129	0.0006	1.2	0.59	1	b6 -C10H9NO -H2O [4|5][1-6]	C24H31N7O6S
553.1874	553.1864	0.0011	1.9	0.34	1	b5 -COCH2 -NH3 [6|7][1-5]	C26H28N6O6S
555.2026	555.2020	0.0005	1.0	17.30	1	b4 [6|7][1-4]	C26H30N6O6S
565.2234	565.2228	0.0007	1.2	7.60	1	b5 -NH3 -CO [4|5][1-5]	C28H32N6O5S
566.2186	566.2180	0.0006	1.0	1.68	1	b5 -H2O -CO [6|7][1-5]	C27H31N7O5S
566.2186	566.2180	0.0006	1.0	1.68	1	b5 -COCH2 -H2O [7|1][1-5]	C27H31N7O5S
567.2023	567.2020	0.0003	0.5	0.25	1	b5 -NH3 -CO [6|7][1-5]	C27H30N6O6S
567.2023	567.2020	0.0003	0.5	0.25	1	b5 -COCH2 -NH3 [7|1][1-5]	C27H30N6O6S
568.2343	568.2337	0.0006	1.1	2.00	1	b5 -CO -COCH2 [5|6][1-5]	C27H33N7O5S
578.2199	578.2180	0.0019	3.3	0.29	1	b5 -COCH2 -H2O [5|6][1-5]	C28H31N7O5S
580.2346	580.2337	0.0010	1.6	0.79	1	b5 -H2O -CO [7|1][1-5]	C28H33N7O5S
581.2186	581.2177	0.0009	1.5	1.43	1	b5 -NH3 -CO [7|1][1-5]	C28H32N6O6S
581.2725	581.2718	0.0006	1.1	3.87	1	b6 -NH3 -CO [1|2][1-6]	C29H36N6O7
582.2503	582.2493	0.0010	1.7	0.94	1	b5 -CO [4|5][1-5]	C28H35N7O5S
584.2292	584.2286	0.0007	1.1	9.45	1	b5 -CO [6|7][1-5]	C27H33N7O6S
584.2292	584.2286	0.0007	1.1	9.45	1	b5 -COCH2 [7|1][1-5]	C27H33N7O6S
592.2352	592.2337	0.0015	2.5	0.30	1	b5 -H2O [4|5][1-5]	C29H33N7O5S
592.2352	592.2337	0.0015	2.5	0.30	1	b5 -H2O -CO [5|6][1-5]	C29H33N7O5S
593.2184	593.2177	0.0008	1.3	0.28	1	b5 -NH3 [4|5][1-5]	C29H32N6O6S
593.2184	593.2177	0.0008	1.3	0.28	1	b5 -NH3 -CO [5|6][1-5]	C29H32N6O6S
593.2505	593.2500	0.0004	0.8	0.51	1	M -C10H9NO -CO [7|1][1-7]	C25H36N8O7S
594.2139	594.2129	0.0010	1.7	3.35	1	b5 -H2O [6|7][1-5]	C28H31N7O6S
596.2294	596.2286	0.0008	1.3	0.32	1	b5 -COCH2 [5|6][1-5]	C28H33N7O6S
598.2449	598.2442	0.0007	1.2	6.72	1	b5 -CO [7|1][1-5]	C28H35N7O6S
603.2352	603.2344	0.0008	1.4	74.03	1	M -C10H9NO -H2O [7|1][1-7]	C26H34N8O7S
604.2192	604.2184	0.0008	1.4	0.17	1	M -C10H9NO -NH3 [7|1][1-7]	C26H33N7O8S

Meas. m/z	Calc. m/z	δ (Da)	δ (ppm)	Rel. Int. (%)	z	Annotation	Formula
608.2296	608.2286	0.0010	1.7	0.87	1	b5 -H2O [7|1][1-5]	C29H33N7O6S
609.2132	609.2126	0.0006	1.0	1.02	1	b5 -NH3 [7|1][1-5]	C29H32N6O7S
610.2448	610.2442	0.0006	1.0	1.36	1	b5 [4|5][1-5]	C29H35N7O6S
610.2448	610.2442	0.0006	1.0	1.36	1	b5 -CO [5|6][1-5]	C29H35N7O6S
612.2243	612.2235	0.0008	1.3	8.68	1	b5 [6|7][1-5]	C28H33N7O7S
620.2291	620.2286	0.0005	0.9	4.27	1	b5 -H2O [5|6][1-5]	C30H33N7O6S
621.2134	621.2126	0.0008	1.3	28.79	1	b5 -NH3 [5|6][1-5]	C30H32N6O7S
621.2458	621.2450	0.0009	1.4	7.30	1	M -C10H9NO [7|1][1-7]	C26H36N8O8S
626.2401	626.2391	0.0009	1.4	1.60	1	b5 [7|1][1-5]	C29H35N7O7S
635.2404	635.2395	0.0009	1.5	0.28	1	b6 -COCH2 -H2O [5|6][1-6]	C30H34N8O6S
636.2239	636.2235	0.0004	0.7	1.16	1	b6 -COCH2 -NH3 [5|6][1-6]	C30H33N7O7S
638.2401	638.2391	0.0009	1.4	10.71	1	b6 -COCH2 -NH3 [6|7][1-6]	C30H35N7O7S
638.2401	638.2391	0.0009	1.4	10.71	1	b5 [5|6][1-5]	C30H35N7O7S
639.2718	639.2708	0.0010	1.6	0.43	1	b6 -CO -COCH2 [7|1][1-6]	C30H38N8O6S
639.2718	639.2708	0.0010	1.6	0.43	1	b6 -CO [3|4][1-6]	C30H38N8O6S
649.2563	649.2551	0.0012	1.9	1.31	1	b6 -COCH2 -H2O [7|1][1-6]	C31H36N8O6S
649.2563	649.2551	0.0012	1.9	1.31	1	b6 -H2O [3|4][1-6]	C31H36N8O6S
649.2563	649.2551	0.0012	1.9	1.31	1	b6 -H2O -CO [5|6][1-6]	C31H36N8O6S
650.2399	650.2391	0.0008	1.2	0.65	1	b6 -NH3 -CO [5|6][1-6]	C31H35N7O7S
650.2399	650.2391	0.0008	1.2	0.65	1	b6 -COCH2 -NH3 [7|1][1-6]	C31H35N7O7S
650.2399	650.2391	0.0008	1.2	0.65	1	b6 -NH3 [3|4][1-6]	C31H35N7O7S
651.2717	651.2708	0.0010	1.5	0.99	1	b6 -H2O -CO [6|7][1-6]	C31H38N8O6S
652.2559	652.2548	0.0011	1.7	0.25	1	b6 -NH3 -CO [6|7][1-6]	C31H37N7O7S
663.2714	663.2708	0.0006	1.0	0.38	1	b6 -H2O -CO [7|1][1-6]	C32H38N8O6S
663.2714	663.2708	0.0006	1.0	0.38	1	b6 -COCH2 -H2O [4|5][1-6]	C32H38N8O6S
667.2666	667.2657	0.0009	1.4	5.34	1	b6 [3|4][1-6]	C31H38N8O7S
667.2666	667.2657	0.0009	1.4	5.34	1	b6 -CO [5|6][1-6]	C31H38N8O7S
667.2666	667.2657	0.0009	1.4	5.34	1	b6 -COCH2 [7|1][1-6]	C31H38N8O7S
669.2823	669.2813	0.0010	1.5	19.53	1	b6 -CO [6|7][1-6]	C31H40N8O7S
677.2510	677.2500	0.0009	1.4	22.61	1	b6 -H2O [5|6][1-6]	C32H36N8O7S
678.2350	678.2341	0.0010	1.5	29.51	1	b6 -NH3 [5|6][1-6]	C32H35N7O8S
678.2712	678.2704	0.0007	1.1	24.25	1	b6 -NH3 -CO [4|5][1-6]	C33H39N7O7S
679.2664	679.2657	0.0007	1.1	1.73	1	b6 -H2O [6|7][1-6]	C32H38N8O7S
681.2823	681.2813	0.0009	1.4	1.89	1	b6 -CO [7|1][1-6]	C32H40N8O7S
681.2823	681.2813	0.0009	1.4	1.89	1	b6 -COCH2 [4|5][1-6]	C32H40N8O7S
691.2669	691.2657	0.0012	1.8	1.38	1	b6 -H2O [7|1][1-6]	C33H38N8O7S
695.2615	695.2606	0.0009	1.3	100.00	1	b6 [5|6][1-6]	C32H38N8O8S
697.2774	697.2763	0.0011	1.6	2.38	1	b6 [6|7][1-6]	C32H40N8O8S
705.2815	705.2813	0.0002	0.2	0.24	1	b6 -H2O [4|5][1-6]	C34H40N8O7S
709.2772	709.2763	0.0010	1.4	4.31	1	b6 [7|1][1-6]	C33H40N8O8S
720.2933	720.2922	0.0011	1.5	3.28	1	M -COCH2 -H2O [7|1][1-7]	C34H41N9O7S
734.3090	734.3079	0.0011	1.5	52.13	1	M -H2O -CO [7|1][1-7]	C35H43N9O7S
735.2930	735.2919	0.0011	1.4	20.51	1	M -NH3 -CO [7|1][1-7]	C35H42N8O8S
745.2768	745.2763	0.0005	0.7	0.63	1	M -H2O -NH3 [7|1][1-7]	C36H40N8O8S
752.3194	752.3185	0.0009	1.2	93.81	1	M -CO [7|1][1-7]	C35H45N9O8S
762.3037	762.3028	0.0009	1.2	85.56	1	M -H2O [7|1][1-7]	C36H43N9O8S
763.2879	763.2868	0.0011	1.4	3.38	1	M -NH3 [7|1][1-7]	C36H42N8O9S

Generated by mMass • Open Source Mass Spectrometry Tool • www.mmass.org

mMass Report: *MSn spectrum [568]*

**Date**	Fri Jul 19 06:14:04 2019	**Scan Number**	568
**Operator**		**Retention Time**	502.2816
**Contact**		**MS Level**	2
**Institution**		**Precursor m/z**	780.31
**Instrument**		**Polarity**	positive
		**Spectrum Points**	0
		**Peak List**	373

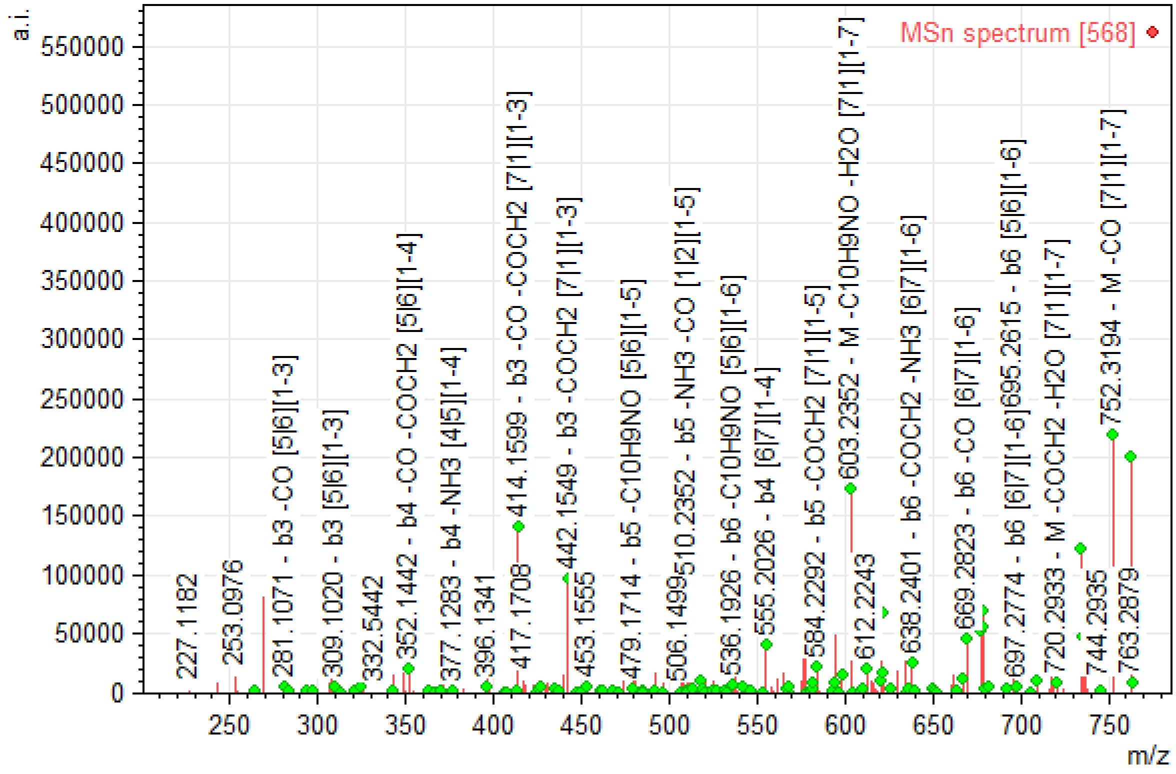

Sequence - *Argyrin-like*

Accession	Length	Mo. Mass	Av. Mass	Coverage	Matched Int.
	7 (Cyclic)	779.3061	779.8652	100.0 %	57.3 %

Ala-Thz | 2Me-dhThr | (OMe)Trp | Gly | Abu | Dhb | Sar

Meas. m/z	Calc. m/z	δ (Da)	δ (ppm)	Rel. Int. (%)	z	Annotation	Formula
264.0803	264.0801	0.0001	0.5	0.57	1	b3 -NH3 -CO [5|6][1-3]	C12H13N3O2S
281.1071	281.1067	0.0004	1.4	2.19	1	b3 -CO [5|6][1-3]	C12H16N4O2S
283.0864	283.0859	0.0004	1.5	0.70	1	b3 -C10H9NO -COCH2 [7|1][1-3]	C11H14N4O3S
293.1072	293.1067	0.0006	1.9	0.64	1	b3 -H2O -CO [6|7][1-3]	C13H16N4O2S
297.1561	297.1557	0.0004	1.2	0.66	1	b4 [3|4][1-4]	C13H20N4O4
309.1020	309.1016	0.0004	1.2	2.45	1	b3 [5|6][1-3]	C13H16N4O3S
313.1187	313.1183	0.0004	1.2	0.28	1	b2 -NH3 [1|2][1-2]	C17H16N2O4
321.1018	321.1016	0.0002	0.7	0.44	1	b3 -H2O [6|7][1-3]	C14H16N4O3S

Meas. m/z	Calc. m/z	δ (Da)	δ (ppm)	Rel. Int. (%)	z	Annotation	Formula
324.1347	324.1343	0.0005	1.4	1.92	1	b3 -H2O -NH3 [2|3][1-3]	C18H17N3O3
342.1450	342.1448	0.0001	0.4	1.08	1	b3 -NH3 [2|3][1-3]	C18H19N3O4
342.1450	342.1448	0.0001	0.4	1.08	1	b3 -NH3 -CO [1|2][1-3]	C18H19N3O4
352.1442	352.1438	0.0004	1.1	8.68	1	b4 -CO -COCH2 [5|6][1-4]	C15H21N5O3S
363.1667	363.1663	0.0004	1.1	0.43	1	b3 +H2O -COCH2 [1|2][1-3]	C17H22N4O5
366.1598	366.1594	0.0003	0.9	0.15	1	b4 -CO [4|5][1-4]	C16H23N5O3S
370.1400	370.1397	0.0003	0.8	0.86	1	b3 -NH3 [1|2][1-3]	C19H19N3O5
377.1283	377.1278	0.0005	1.2	0.57	1	b4 -NH3 [4|5][1-4]	C17H20N4O4S
377.1283	377.1278	0.0005	1.2	0.57	1	b4 -NH3 -CO [5|6][1-4]	C17H20N4O4S
396.1341	396.1336	0.0005	1.2	2.09	1	b4 -C10H9NO [6|7][1-4]	C16H21N5O5S
406.1544	406.1544	0.0001	0.2	0.25	1	b5 -NH3 -CO [3|4][1-5]	C18H23N5O4S
407.1721	407.1714	0.0007	1.7	0.28	1	b4 -H2O -NH3 [2|3][1-4]	C22H22N4O4
413.1826	413.1819	0.0006	1.5	0.61	1	b4 -COCH2 -NH3 [1|2][1-4]	C21H24N4O5
414.1599	414.1594	0.0005	1.2	60.14	1	b3 -CO -COCH2 [7|1][1-3]	C20H23N5O3S
424.1445	424.1438	0.0007	1.6	0.35	1	b3 -COCH2 -H2O [7|1][1-3]	C21H21N5O3S
425.1824	425.1819	0.0005	1.1	1.63	1	b4 -NH3 [2|3][1-4]	C22H24N4O5
427.1980	427.1976	0.0004	1.0	2.25	1	b4 -NH3 -CO [1|2][1-4]	C22H26N4O5
435.1448	435.1445	0.0003	0.7	1.28	1	b5 -C10H9NO -H2O [6|7][1-5]	C18H22N6O5S
437.1603	437.1602	0.0002	0.4	0.95	1	b5 -C10H9NO -COCH2 [5|6][1-5]	C18H24N6O5S
442.1549	442.1544	0.0005	1.2	41.13	1	b3 -COCH2 [7|1][1-3]	C21H23N5O4S
447.1814	447.1809	0.0005	1.0	0.21	1	b5 -COCH2 -H2O [4|5][1-5]	C20H26N6O4S
449.1596	449.1602	-0.0006	-1.2	0.13	1	b5 -C10H9NO -H2O [7|1][1-5]	C19H24N6O5S
453.1555	453.1551	0.0005	1.0	2.05	1	b5 -C10H9NO [6|7][1-5]	C18H24N6O6S
461.1608	461.1602	0.0006	1.3	0.47	1	b5 -C10H9NO -H2O [5|6][1-5]	C20H24N6O5S
462.1448	462.1442	0.0006	1.4	1.03	1	b5 -C10H9NO -NH3 [5|6][1-5]	C20H23N5O6S
467.1387	467.1384	0.0003	0.7	0.39	1	b3 -NH3 [7|1][1-3]	C23H22N4O5S
471.1803	471.1809	-0.0006	-1.2	0.14	1	b4 -CO -COCH2 [7|1][1-4]	C22H26N6O4S
479.1714	479.1707	0.0007	1.4	1.17	1	b5 -C10H9NO [5|6][1-5]	C20H26N6O6S
484.1653	484.1649	0.0004	0.8	0.77	1	b3 [7|1][1-3]	C23H25N5O5S
485.1968	485.1966	0.0003	0.6	0.14	1	b4 -CO -COCH2 [6|7][1-4]	C23H28N6O4S
490.1873	490.1867	0.0005	1.1	0.28	1	b6 -C10H9NO -H2O [2|3][1-6]	C21H27N7O5S
491.1711	491.1707	0.0004	0.7	0.76	1	b6 -C10H9NO -NH3 [2|3][1-6]	C21H26N6O6S
496.1660	496.1649	0.0011	2.3	0.14	1	b4 -COCH2 -NH3 [6|7][1-4]	C24H25N5O5S
496.1660	496.1649	0.0011	2.3	0.14	1	b4 -NH3 -CO [7|1][1-4]	C24H25N5O5S
496.2204	496.2191	0.0013	2.7	0.28	1	b5 -COCH2 -NH3 [1|2][1-5]	C25H29N5O6
496.2204	496.2191	0.0013	2.7	0.28	1	b5 -NH3 [2|3][1-5]	C25H29N5O6
506.1499	506.1493	0.0006	1.2	0.36	1	b4 -H2O -NH3 [7|1][1-4]	C25H23N5O5S
509.1969	509.1966	0.0003	0.6	0.81	1	b4 -H2O -CO [6|7][1-4]	C25H28N6O4S
510.1814	510.1806	0.0009	1.7	0.32	1	b4 -NH3 -CO [6|7][1-4]	C25H27N5O5S
510.2352	510.2347	0.0005	1.0	39.11	1	b5 -NH3 -CO [1|2][1-5]	C26H31N5O6
513.1923	513.1915	0.0009	1.7	1.47	1	b4 -CO [7|1][1-4]	C24H28N6O5S
513.1923	513.1915	0.0009	1.7	1.47	1	b4 -COCH2 [6|7][1-4]	C24H28N6O5S
518.1821	518.1816	0.0004	0.8	4.02	1	b6 -C10H9NO -H2O [5|6][1-6]	C22H27N7O6S
519.1663	519.1656	0.0006	1.2	1.02	1	b6 -C10H9NO -NH3 [5|6][1-6]	C22H26N6O7S
519.2025	519.2020	0.0005	0.9	1.42	1	b6 -NH3 -CO [3|4][1-6]	C23H30N6O6S
520.2206	520.2191	0.0016	3.0	0.17	1	b5 -H2O -NH3 [1|2][1-5]	C27H29N5O6
524.1603	524.1598	0.0005	1.0	1.01	1	b4 -NH3 [7|1][1-4]	C25H25N5O6S
527.2077	527.2071	0.0006	1.1	0.49	1	b4 -CO [6|7][1-4]	C25H30N6O5S

Meas. m/z	Calc. m/z	δ (Da)	δ (ppm)	Rel. Int. (%)	z	Annotation	Formula
531.2034	531.2020	0.0013	2.5	0.90	1	b4 +H2O -CO [7|1][1-4]	C24H30N6O6S
531.2034	531.2020	0.0013	2.5	0.90	1	b4 +H2O -COCH2 [6|7][1-4]	C24H30N6O6S
532.1981	532.1973	0.0008	1.5	0.14	1	b6 -C10H9NO -H2O [7|1][1-6]	C23H29N7O6S
536.1926	536.1922	0.0004	0.7	2.73	1	b6 -C10H9NO [5|6][1-6]	C22H29N7O7S
541.1871	541.1864	0.0008	1.4	2.38	1	b4 [7|1][1-4]	C25H28N6O6S
546.2136	546.2129	0.0006	1.2	0.59	1	b6 -C10H9NO -H2O [4|5][1-6]	C24H31N7O6S
546.2136	546.2129	0.0006	1.2	0.59	1	b6 -H2O [3|4][1-6]	C24H31N7O6S
553.1874	553.1864	0.0011	1.9	0.34	1	b5 -COCH2 -NH3 [6|7][1-5]	C26H28N6O6S
555.2026	555.2020	0.0005	1.0	17.30	1	b4 [6|7][1-4]	C26H30N6O6S
566.2186	566.2180	0.0006	1.0	1.68	1	b5 -H2O -CO [6|7][1-5]	C27H31N7O5S
566.2186	566.2180	0.0006	1.0	1.68	1	b5 -COCH2 -H2O [7|1][1-5]	C27H31N7O5S
567.2023	567.2020	0.0003	0.5	0.25	1	b5 -NH3 -CO [6|7][1-5]	C27H30N6O6S
567.2023	567.2020	0.0003	0.5	0.25	1	b5 -COCH2 -NH3 [7|1][1-5]	C27H30N6O6S
568.2343	568.2337	0.0006	1.1	2.00	1	b5 -CO -COCH2 [5|6][1-5]	C27H33N7O5S
578.2199	578.2180	0.0019	3.3	0.29	1	b5 -COCH2 -H2O [5|6][1-5]	C28H31N7O5S
580.2346	580.2337	0.0010	1.6	0.79	1	b5 -H2O -CO [7|1][1-5]	C28H33N7O5S
581.2186	581.2177	0.0009	1.5	1.43	1	b5 -NH3 -CO [7|1][1-5]	C28H32N6O6S
581.2725	581.2718	0.0006	1.1	3.87	1	b6 -NH3 -CO [1|2][1-6]	C29H36N6O7
584.2292	584.2286	0.0007	1.1	9.45	1	b5 -COCH2 [7|1][1-5]	C27H33N7O6S
584.2292	584.2286	0.0007	1.1	9.45	1	b5 -CO [6|7][1-5]	C27H33N7O6S
592.2352	592.2337	0.0015	2.5	0.30	1	b5 -H2O -CO [5|6][1-5]	C29H33N7O5S
593.2184	593.2177	0.0008	1.3	0.28	1	b5 -NH3 -CO [5|6][1-5]	C29H32N6O6S
593.2505	593.2500	0.0004	0.8	0.51	1	M -C10H9NO -CO [7|1][1-7]	C25H36N8O7S
594.2139	594.2129	0.0010	1.7	3.35	1	b5 -H2O [6|7][1-5]	C28H31N7O6S
596.2294	596.2286	0.0008	1.3	0.32	1	b5 -COCH2 [5|6][1-5]	C28H33N7O6S
598.2449	598.2442	0.0007	1.2	6.72	1	b5 -CO [7|1][1-5]	C28H35N7O6S
603.2352	603.2344	0.0008	1.4	74.03	1	M -C10H9NO -H2O [7|1][1-7]	C26H34N8O7S
604.2192	604.2184	0.0008	1.4	0.17	1	M -C10H9NO -NH3 [7|1][1-7]	C26H33N7O8S
608.2296	608.2286	0.0010	1.7	0.87	1	b5 -H2O [7|1][1-5]	C29H33N7O6S
609.2132	609.2126	0.0006	1.0	1.02	1	b5 -NH3 [7|1][1-5]	C29H32N6O7S
610.2448	610.2442	0.0006	1.0	1.36	1	b5 -CO [5|6][1-5]	C29H35N7O6S
612.2243	612.2235	0.0008	1.3	8.68	1	b5 [6|7][1-5]	C28H33N7O7S
620.2291	620.2286	0.0005	0.9	4.27	1	b5 -H2O [5|6][1-5]	C30H33N7O6S
621.2134	621.2126	0.0008	1.3	28.79	1	b5 -NH3 [5|6][1-5]	C30H32N6O7S
621.2458	621.2450	0.0009	1.4	7.30	1	M -C10H9NO [7|1][1-7]	C26H36N8O8S
626.2401	626.2391	0.0009	1.4	1.60	1	b5 [7|1][1-5]	C29H35N7O7S
635.2404	635.2395	0.0009	1.5	0.28	1	b6 -COCH2 -H2O [5|6][1-6]	C30H34N8O6S
636.2239	636.2235	0.0004	0.7	1.16	1	b6 -COCH2 -NH3 [5|6][1-6]	C30H33N7O7S
638.2401	638.2391	0.0009	1.4	10.71	1	b5 [5|6][1-5]	C30H35N7O7S
638.2401	638.2391	0.0009	1.4	10.71	1	b6 -COCH2 -NH3 [6|7][1-6]	C30H35N7O7S
639.2718	639.2708	0.0010	1.6	0.43	1	b6 -CO [2|3][1-6]	C30H38N8O6S
639.2718	639.2708	0.0010	1.6	0.43	1	b6 -CO -COCH2 [7|1][1-6]	C30H38N8O6S
649.2563	649.2551	0.0012	1.9	1.31	1	b6 -H2O [2|3][1-6]	C31H36N8O6S
649.2563	649.2551	0.0012	1.9	1.31	1	b6 -H2O -CO [5|6][1-6]	C31H36N8O6S
649.2563	649.2551	0.0012	1.9	1.31	1	b6 -COCH2 -H2O [7|1][1-6]	C31H36N8O6S
650.2399	650.2391	0.0008	1.2	0.65	1	b6 -COCH2 -NH3 [7|1][1-6]	C31H35N7O7S
650.2399	650.2391	0.0008	1.2	0.65	1	b6 -NH3 [2|3][1-6]	C31H35N7O7S
650.2399	650.2391	0.0008	1.2	0.65	1	b6 -NH3 -CO [5|6][1-6]	C31H35N7O7S

Meas. m/z	Calc. m/z	δ (Da)	δ (ppm)	Rel. Int. (%)	z	Annotation	Formula
651.2717	651.2708	0.0010	1.5	0.99	1	b6 -H2O -CO [6|7][1-6]	C31H38N8O6S
652.2559	652.2548	0.0011	1.7	0.25	1	b6 -NH3 -CO [6|7][1-6]	C31H37N7O7S
663.2714	663.2708	0.0006	1.0	0.38	1	b6 -H2O -CO [7|1][1-6]	C32H38N8O6S
663.2714	663.2708	0.0006	1.0	0.38	1	b6 -COCH2 -H2O [4|5][1-6]	C32H38N8O6S
667.2666	667.2657	0.0009	1.4	5.34	1	b6 [2|3][1-6]	C31H38N8O7S
667.2666	667.2657	0.0009	1.4	5.34	1	b6 -COCH2 [7|1][1-6]	C31H38N8O7S
667.2666	667.2657	0.0009	1.4	5.34	1	b6 -CO [5|6][1-6]	C31H38N8O7S
669.2823	669.2813	0.0010	1.5	19.53	1	b6 -CO [6|7][1-6]	C31H40N8O7S
677.2510	677.2500	0.0009	1.4	22.61	1	b6 -H2O [5|6][1-6]	C32H36N8O7S
678.2350	678.2341	0.0010	1.5	29.51	1	b6 -NH3 [5|6][1-6]	C32H35N7O8S
678.2712	678.2704	0.0007	1.1	24.25	1	b6 -NH3 -CO [4|5][1-6]	C33H39N7O7S
679.2664	679.2657	0.0007	1.1	1.73	1	b6 -H2O [6|7][1-6]	C32H38N8O7S
681.2823	681.2813	0.0009	1.4	1.89	1	b6 -COCH2 [4|5][1-6]	C32H40N8O7S
681.2823	681.2813	0.0009	1.4	1.89	1	b6 -CO [7|1][1-6]	C32H40N8O7S
691.2669	691.2657	0.0012	1.8	1.38	1	b6 -H2O [7|1][1-6]	C33H38N8O7S
695.2615	695.2606	0.0009	1.3	100.00	1	b6 [5|6][1-6]	C32H38N8O8S
697.2774	697.2763	0.0011	1.6	2.38	1	b6 [6|7][1-6]	C32H40N8O8S
705.2815	705.2813	0.0002	0.2	0.24	1	b6 -H2O [4|5][1-6]	C34H40N8O7S
709.2772	709.2763	0.0010	1.4	4.31	1	b6 [7|1][1-6]	C33H40N8O8S
720.2933	720.2922	0.0011	1.5	3.28	1	M -COCH2 -H2O [7|1][1-7]	C34H41N9O7S
734.3090	734.3079	0.0011	1.5	52.13	1	M -H2O -CO [7|1][1-7]	C35H43N9O7S
735.2930	735.2919	0.0011	1.4	20.51	1	M -NH3 -CO [7|1][1-7]	C35H42N8O8S
745.2768	745.2763	0.0005	0.7	0.63	1	M -H2O -NH3 [7|1][1-7]	C36H40N8O8S
752.3194	752.3185	0.0009	1.2	93.81	1	M -CO [7|1][1-7]	C35H45N9O8S
762.3037	762.3028	0.0009	1.2	85.56	1	M -H2O [7|1][1-7]	C36H43N9O8S
763.2879	763.2868	0.0011	1.4	3.38	1	M -NH3 [7|1][1-7]	C36H42N8O9S

Generated by mMass • Open Source Mass Spectrometry Tool • www.mmass.org

mMass Report: *MSn spectrum [568]*

**Date**	Fri Jul 19 06:14:04 2019	**Scan Number**	568
**Operator**		**Retention Time**	502.2816
**Contact**		**MS Level**	2
**Institution**		**Precursor m/z**	780.31
**Instrument**		**Polarity**	positive
		**Spectrum Points**	0
		**Peak List**	373

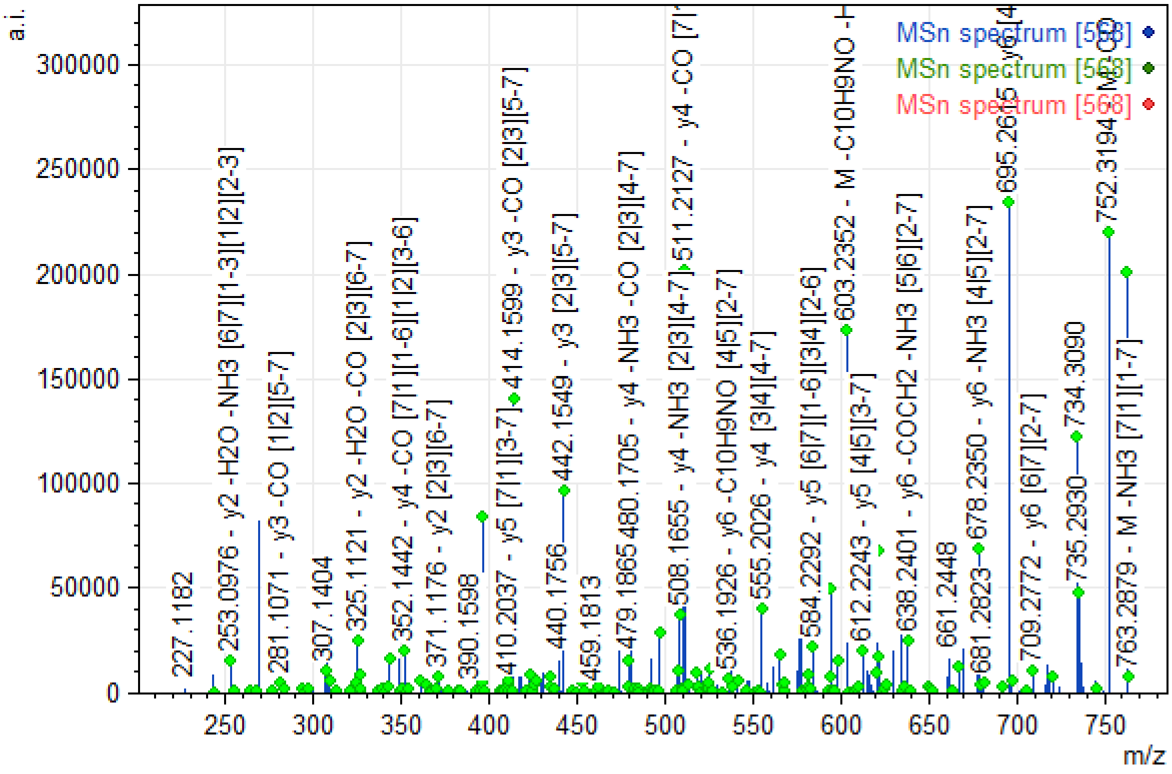

Sequence - *Zelkovamycin_scrambling*

Accession	Length	Mo. Mass	Av. Mass	Coverage	Matched Int.
	7 (Cyclic)	779.3061	779.8652	100.0 %	76.8 %

`Ala-Thz | (OMe)Trp | 2Me-dhThr | Gly | Abu | Dhb | Sar`

Meas. m/z	Calc. m/z	δ (Da)	δ (ppm)	Rel. Int. (%)	z	Annotation	Formula
243.1131	243.1128	0.0003	1.2	0.16	1	y2 -NH3 -CO [1|2][1-6][1|2][5-6]	C14H14N2O2
243.1131	243.1128	0.0003	1.2	0.16	1	y2 -NH3 -CO [6|7][1-3][1|2][2-3]	C14H14N2O2
243.1131	243.1128	0.0003	1.2	0.16	1	b2 -NH3 -CO [6|7][1-3][2|3][1-2]	C14H14N2O2
243.1131	243.1128	0.0003	1.2	0.16	1	b2 -NH3 -CO [1|2][1-6][5|6][1-2]	C14H14N2O2
253.0976	253.0972	0.0004	1.6	6.54	1	b2 -H2O -NH3 [6|7][1-3][2|3][1-2]	C15H12N2O2
253.0976	253.0972	0.0004	1.6	6.54	1	y2 -H2O -NH3 [6|7][1-3][1|2][2-3]	C15H12N2O2
253.0976	253.0972	0.0004	1.6	6.54	1	y2 -H2O -NH3 [1|2][1-6][1|2][5-6]	C15H12N2O2
253.0976	253.0972	0.0004	1.6	6.54	1	b2 -H2O -NH3 [1|2][1-6][5|6][1-2]	C15H12N2O2

Meas. m/z	Calc. m/z	δ (Da)	δ (ppm)	Rel. Int. (%)	z	Annotation	Formula
255.1133	255.1128	0.0005	1.9	0.38	1	b2 -NH3 -CO [5|6][1-4][3|4][1-2]	C15H14N2O2
255.1133	255.1128	0.0005	1.9	0.38	1	b2 -NH3 -CO [1|2][1-5][4|5][1-2]	C15H14N2O2
255.1133	255.1128	0.0005	1.9	0.38	1	y2 -NH3 -CO [1|2][1-5][1|2][4-5]	C15H14N2O2
255.1133	255.1128	0.0005	1.9	0.38	1	y2 -NH3 -CO [5|6][1-4][1|2][3-4]	C15H14N2O2
264.0803	264.0801	0.0001	0.5	0.57	1	b3 -NH3 -CO [5|6][1-3]	C12H13N3O2S
264.0803	264.0801	0.0001	0.5	0.57	1	y3 -NH3 -CO [1|2][5-7]	C12H13N3O2S
267.1134	267.1128	0.0006	2.2	0.54	1	b2 -H2O -NH3 [1|2][1-4][3|4][1-2]	C16H14N2O2
267.1134	267.1128	0.0006	2.2	0.54	1	y2 -H2O -NH3 [4|5][1-5][1|2][4-5]	C16H14N2O2
267.1134	267.1128	0.0006	2.2	0.54	1	y2 -H2O -NH3 [1|2][1-4][1|2][3-4]	C16H14N2O2
267.1134	267.1128	0.0006	2.2	0.54	1	b2 -H2O -NH3 [4|5][1-5][4|5][1-2]	C16H14N2O2
276.0804	276.0801	0.0002	0.8	0.31	1	b3 -H2O -NH3 [6|7][1-6][5|6][1-3]	C13H13N3O2S
276.0804	276.0801	0.0002	0.8	0.31	1	b3 -H2O -NH3 [4|5][1-4][2|3][1-3]	C13H13N3O2S
276.0804	276.0801	0.0002	0.8	0.31	1	y3 -H2O -NH3 [6|7][1-6][2|3][4-6]	C13H13N3O2S
276.0804	276.0801	0.0002	0.8	0.31	1	y3 -H2O -NH3 [4|5][1-4][1|2][2-4]	C13H13N3O2S
281.1071	281.1067	0.0004	1.4	2.19	1	b3 -CO [5|6][1-3]	C12H16N4O2S
281.1071	281.1067	0.0004	1.4	2.19	1	y3 -CO [1|2][5-7]	C12H16N4O2S
283.0864	283.0859	0.0004	1.5	0.70	1	b3 -C10H9NO -COCH2 [7|1][1-3]	C11H14N4O3S
283.0864	283.0859	0.0004	1.5	0.70	1	y3 -C10H9NO [2|3][5-7]	C11H14N4O3S
283.0864	283.0859	0.0004	1.5	0.70	1	b3 -COCH2 [7|1][1-4][2|3][1-3]	C11H14N4O3S
283.0864	283.0859	0.0004	1.5	0.70	1	b3 [3|4][1-5][3|4][1-3]	C11H14N4O3S
283.0864	283.0859	0.0004	1.5	0.70	1	y3 [6|7][1-5][2|3][3-5]	C11H14N4O3S
283.0864	283.0859	0.0004	1.5	0.70	1	y3 -COCH2 [7|1][1-4][1|2][2-4]	C11H14N4O3S
283.0864	283.0859	0.0004	1.5	0.70	1	y3 -C10H9NO -COCH2 [3|4][5-7]	C11H14N4O3S
283.0864	283.0859	0.0004	1.5	0.70	1	b3 -C10H9NO [6|7][1-3]	C11H14N4O3S
283.0864	283.0859	0.0004	1.5	0.70	1	y3 [3|4][1-5][1|2][3-5]	C11H14N4O3S
283.0864	283.0859	0.0004	1.5	0.70	1	y3 -COCH2 [2|3][1-6][2|3][4-6]	C11H14N4O3S
283.0864	283.0859	0.0004	1.5	0.70	1	b3 -COCH2 [2|3][1-6][5|6][1-3]	C11H14N4O3S
283.0864	283.0859	0.0004	1.5	0.70	1	b3 [6|7][1-5][4|5][1-3]	C11H14N4O3S
283.1082	283.1077	0.0005	1.6	0.84	1	b2 -NH3 [5|6][1-4][3|4][1-2]	C16H14N2O3
283.1082	283.1077	0.0005	1.6	0.84	1	y2 -NH3 [1|2][1-5][1|2][4-5]	C16H14N2O3
283.1082	283.1077	0.0005	1.6	0.84	1	b2 -NH3 [1|2][1-5][4|5][1-2]	C16H14N2O3
283.1082	283.1077	0.0005	1.6	0.84	1	y2 -NH3 [5|6][1-4][1|2][3-4]	C16H14N2O3
293.1072	293.1067	0.0006	1.9	0.64	1	y3 -H2O -CO [2|3][1-6][1|2][4-6]	C13H16N4O2S
293.1072	293.1067	0.0006	1.9	0.64	1	b3 -H2O -CO [6|7][1-4][3|4][1-3]	C13H16N4O2S
293.1072	293.1067	0.0006	1.9	0.64	1	b3 -H2O -CO [2|3][1-6][4|5][1-3]	C13H16N4O2S
293.1072	293.1067	0.0006	1.9	0.64	1	y3 -H2O [4|5][1-4][1|2][2-4]	C13H16N4O2S
293.1072	293.1067	0.0006	1.9	0.64	1	b3 -H2O [6|7][1-6][5|6][1-3]	C13H16N4O2S
293.1072	293.1067	0.0006	1.9	0.64	1	b3 -H2O [4|5][1-4][2|3][1-3]	C13H16N4O2S
293.1072	293.1067	0.0006	1.9	0.64	1	y3 -H2O -CO [6|7][1-4][2|3][2-4]	C13H16N4O2S
293.1072	293.1067	0.0006	1.9	0.64	1	y3 -H2O [6|7][1-6][2|3][4-6]	C13H16N4O2S
295.0868	295.0859	0.0009	3.0	0.13	1	y3 -C10H9NO [7|1][1-6][2|3][4-6]	C12H14N4O3S
295.0868	295.0859	0.0009	3.0	0.13	1	y3 -C10H9NO [5|6][1-4][1|2][2-4]	C12H14N4O3S
295.0868	295.0859	0.0009	3.0	0.13	1	b3 -C10H9NO [5|6][1-4][2|3][1-3]	C12H14N4O3S
295.0868	295.0859	0.0009	3.0	0.13	1	b3 -C10H9NO [7|1][1-6][5|6][1-3]	C12H14N4O3S
297.1561	297.1557	0.0004	1.2	0.66	1	b4 -CO [5|6][1-6][4|5][1-4]	C13H20N4O4
297.1561	297.1557	0.0004	1.2	0.66	1	b4 -C10H9NO -CO [5|6][1-5][3|4][1-4]	C13H20N4O4
297.1561	297.1557	0.0004	1.2	0.66	1	b4 -COCH2 [2|3][1-4]	C13H20N4O4
297.1561	297.1557	0.0004	1.2	0.66	1	b4 -C10H9NO [4|5][1-5][4|5][1-4]	C13H20N4O4

Meas. m/z	Calc. m/z	δ (Da)	δ (ppm)	Rel. Int. (%)	z	Annotation	Formula
297.1561	297.1557	0.0004	1.2	0.66	1	y4 [7|1][4-7]	C13H20N4O4
297.1561	297.1557	0.0004	1.2	0.66	1	b4 -C10H9NO -CO [1|2][1-6][4|5][1-4]	C13H20N4O4
297.1561	297.1557	0.0004	1.2	0.66	1	y4 -C10H9NO [4|5][1-5][3|4][2-5]	C13H20N4O4
297.1561	297.1557	0.0004	1.2	0.66	1	y4 -C10H9NO -COCH2 [4|5][1-6][2|3][3-6]	C13H20N4O4
297.1561	297.1557	0.0004	1.2	0.66	1	y4 -C10H9NO -CO [1|2][1-6][2|3][3-6]	C13H20N4O4
297.1561	297.1557	0.0004	1.2	0.66	1	b4 -C10H9NO -COCH2 [1|2][1-5][3|4][1-4]	C13H20N4O4
297.1561	297.1557	0.0004	1.2	0.66	1	y4 -C10H9NO [1|2][1-6][1|2][3-6]	C13H20N4O4
297.1561	297.1557	0.0004	1.2	0.66	1	y4 -C10H9NO -CO [5|6][1-5][2|3][2-5]	C13H20N4O4
297.1561	297.1557	0.0004	1.2	0.66	1	y4 -COCH2 [6|7][4-7]	C13H20N4O4
297.1561	297.1557	0.0004	1.2	0.66	1	b4 -C10H9NO [1|2][1-6][3|4][1-4]	C13H20N4O4
297.1561	297.1557	0.0004	1.2	0.66	1	y4 -CO [2|3][1-5][2|3][2-5]	C13H20N4O4
297.1561	297.1557	0.0004	1.2	0.66	1	b4 -CO [2|3][1-5][3|4][1-4]	C13H20N4O4
297.1561	297.1557	0.0004	1.2	0.66	1	b4 [3|4][1-4]	C13H20N4O4
297.1561	297.1557	0.0004	1.2	0.66	1	y4 -C10H9NO -COCH2 [1|2][1-5][2|3][2-5]	C13H20N4O4
297.1561	297.1557	0.0004	1.2	0.66	1	b4 -C10H9NO -COCH2 [4|5][1-6][4|5][1-4]	C13H20N4O4
297.1561	297.1557	0.0004	1.2	0.66	1	y4 -CO [5|6][1-6][2|3][3-6]	C13H20N4O4
307.1404	307.1401	0.0004	1.2	4.30	1	b4 -H2O [5|6][1-6][4|5][1-4]	C14H18N4O4
307.1404	307.1401	0.0004	1.2	4.30	1	b4 -C10H9NO -H2O [5|6][1-5][3|4][1-4]	C14H18N4O4
307.1404	307.1401	0.0004	1.2	4.30	1	y4 -C10H9NO -H2O [5|6][1-5][2|3][2-5]	C14H18N4O4
307.1404	307.1401	0.0004	1.2	4.30	1	b4 -C10H9NO -H2O [1|2][1-6][4|5][1-4]	C14H18N4O4
307.1404	307.1401	0.0004	1.2	4.30	1	b4 -H2O [2|3][1-5][3|4][1-4]	C14H18N4O4
307.1404	307.1401	0.0004	1.2	4.30	1	y4 -C10H9NO -H2O [1|2][1-6][2|3][3-6]	C14H18N4O4
307.1404	307.1401	0.0004	1.2	4.30	1	y4 -H2O [5|6][1-6][2|3][3-6]	C14H18N4O4
307.1404	307.1401	0.0004	1.2	4.30	1	y4 -H2O [2|3][1-5][2|3][2-5]	C14H18N4O4
309.1020	309.1016	0.0004	1.2	2.45	1	b3 [5|6][1-3]	C13H16N4O3S
309.1020	309.1016	0.0004	1.2	2.45	1	y3 [1|2][5-7]	C13H16N4O3S
313.1187	313.1183	0.0004	1.2	0.28	1	b2 -NH3 [1|2][1-2]	C17H16N2O4
313.1187	313.1183	0.0004	1.2	0.28	1	y2 -NH3 [3|4][6-7]	C17H16N2O4
321.1018	321.1016	0.0002	0.7	0.44	1	b3 -H2O [2|3][1-6][4|5][1-3]	C14H16N4O3S
321.1018	321.1016	0.0002	0.7	0.44	1	b3 -H2O [6|7][1-4][3|4][1-3]	C14H16N4O3S
321.1018	321.1016	0.0002	0.7	0.44	1	b4 -NH3 -CO [5|6][1-6][5|6][1-4]	C14H16N4O3S
321.1018	321.1016	0.0002	0.7	0.44	1	y3 -H2O [2|3][1-6][1|2][4-6]	C14H16N4O3S
321.1018	321.1016	0.0002	0.7	0.44	1	y4 -NH3 -CO [3|4][1-5][1|2][2-5]	C14H16N4O3S
321.1018	321.1016	0.0002	0.7	0.44	1	y3 -H2O [6|7][1-4][2|3][2-4]	C14H16N4O3S
321.1018	321.1016	0.0002	0.7	0.44	1	y4 -NH3 -CO [5|6][1-6][3|4][3-6]	C14H16N4O3S
321.1018	321.1016	0.0002	0.7	0.44	1	b4 -NH3 -CO [3|4][1-5][2|3][1-4]	C14H16N4O3S
321.1562	321.1557	0.0004	1.3	0.27	1	y4 -C10H9NO -H2O [1|2][1-5][2|3][2-5]	C15H20N4O4
321.1562	321.1557	0.0004	1.3	0.27	1	b4 -C10H9NO -H2O [1|2][1-5][3|4][1-4]	C15H20N4O4
321.1562	321.1557	0.0004	1.3	0.27	1	y4 -C10H9NO -H2O [4|5][1-6][2|3][3-6]	C15H20N4O4
321.1562	321.1557	0.0004	1.3	0.27	1	b4 -C10H9NO -H2O [4|5][1-6][4|5][1-4]	C15H20N4O4
321.1562	321.1557	0.0004	1.3	0.27	1	y4 -H2O [6|7][4-7]	C15H20N4O4
321.1562	321.1557	0.0004	1.3	0.27	1	b4 -H2O [2|3][1-4]	C15H20N4O4
324.1347	324.1343	0.0005	1.4	1.92	1	y3 -H2O -NH3 [1|2][1-4][1|2][2-4]	C18H17N3O3
324.1347	324.1343	0.0005	1.4	1.92	1	b3 -H2O -NH3 [3|4][1-6][5|6][1-3]	C18H17N3O3
324.1347	324.1343	0.0005	1.4	1.92	1	b3 -H2O -NH3 [1|2][1-4][2|3][1-3]	C18H17N3O3
324.1347	324.1343	0.0005	1.4	1.92	1	y3 -H2O -NH3 [3|4][1-6][2|3][4-6]	C18H17N3O3
325.1121	325.1118	0.0004	1.1	10.51	1	b2 -H2O -CO [7|1][1-2]	C17H16N4OS
325.1121	325.1118	0.0004	1.1	10.51	1	y2 -H2O -CO [2|3][6-7]	C17H16N4OS

Meas. m/z	Calc. m/z	δ (Da)	δ (ppm)	Rel. Int. (%)	z	Annotation	Formula
326.0960	326.0958	0.0003	0.8	3.70	1	y2 -NH3 -CO [2|3][6-7]	C17H15N3O2S
326.0960	326.0958	0.0003	0.8	3.70	1	b2 -NH3 -CO [7|1][1-2]	C17H15N3O2S
326.1285	326.1281	0.0004	1.1	0.58	1	y4 -C10H9NO -CO [7|1][1-5][2|3][2-5]	C13H19N5O3S
326.1285	326.1281	0.0004	1.1	0.58	1	y4 -CO -COCH2 [2|3][1-6][2|3][3-6]	C13H19N5O3S
326.1285	326.1281	0.0004	1.1	0.58	1	b4 -CO -COCH2 [2|3][1-6][4|5][1-4]	C13H19N5O3S
326.1285	326.1281	0.0004	1.1	0.58	1	b4 -C10H9NO -CO [3|4][1-6][4|5][1-4]	C13H19N5O3S
326.1285	326.1281	0.0004	1.1	0.58	1	y4 -CO -COCH2 [6|7][1-5][2|3][2-5]	C13H19N5O3S
326.1285	326.1281	0.0004	1.1	0.58	1	b4 -C10H9NO -CO [7|1][1-5][3|4][1-4]	C13H19N5O3S
326.1285	326.1281	0.0004	1.1	0.58	1	y4 -C10H9NO -CO [3|4][1-6][2|3][3-6]	C13H19N5O3S
326.1285	326.1281	0.0004	1.1	0.58	1	b4 -CO -COCH2 [6|7][1-5][3|4][1-4]	C13H19N5O3S
326.1504	326.1499	0.0005	1.4	0.66	1	y3 -NH3 -CO [5|6][1-4][2|3][2-4]	C18H19N3O3
326.1504	326.1499	0.0005	1.4	0.66	1	b3 -NH3 -CO [1|2][1-6][4|5][1-3]	C18H19N3O3
326.1504	326.1499	0.0005	1.4	0.66	1	y3 -NH3 -CO [1|2][1-6][1|2][4-6]	C18H19N3O3
326.1504	326.1499	0.0005	1.4	0.66	1	b3 -NH3 -CO [5|6][1-4][3|4][1-3]	C18H19N3O3
337.0969	337.0965	0.0004	1.3	0.29	1	b4 -COCH2 -NH3 [6|7][1-5][3|4][1-4]	C14H16N4O4S
337.0969	337.0965	0.0004	1.3	0.29	1	b4 -C10H9NO -NH3 [3|4][1-6][4|5][1-4]	C14H16N4O4S
337.0969	337.0965	0.0004	1.3	0.29	1	y4 -COCH2 -NH3 [6|7][1-5][2|3][2-5]	C14H16N4O4S
337.0969	337.0965	0.0004	1.3	0.29	1	y4 -C10H9NO -NH3 [3|4][1-6][2|3][3-6]	C14H16N4O4S
337.0969	337.0965	0.0004	1.3	0.29	1	y4 -COCH2 -NH3 [2|3][1-6][2|3][3-6]	C14H16N4O4S
337.0969	337.0965	0.0004	1.3	0.29	1	y4 -C10H9NO -NH3 [7|1][1-5][2|3][2-5]	C14H16N4O4S
337.0969	337.0965	0.0004	1.3	0.29	1	b4 -C10H9NO -NH3 [7|1][1-5][3|4][1-4]	C14H16N4O4S
337.0969	337.0965	0.0004	1.3	0.29	1	b4 -COCH2 -NH3 [2|3][1-6][4|5][1-4]	C14H16N4O4S
339.1667	339.1663	0.0004	1.1	0.64	1	y4 -C10H9NO [4|5][1-6][2|3][3-6]	C15H22N4O5
339.1667	339.1663	0.0004	1.1	0.64	1	y4 -C10H9NO [1|2][1-5][2|3][2-5]	C15H22N4O5
339.1667	339.1663	0.0004	1.1	0.64	1	b4 -C10H9NO [1|2][1-5][3|4][1-4]	C15H22N4O5
339.1667	339.1663	0.0004	1.1	0.64	1	b4 [2|3][1-4]	C15H22N4O5
339.1667	339.1663	0.0004	1.1	0.64	1	b4 -C10H9NO [4|5][1-6][4|5][1-4]	C15H22N4O5
339.1667	339.1663	0.0004	1.1	0.64	1	y4 [6|7][4-7]	C15H22N4O5
340.1661	340.1656	0.0005	1.6	0.16	1	b3 -NH3 -CO [4|5][1-5][4|5][1-3]	C19H21N3O3
340.1661	340.1656	0.0005	1.6	0.16	1	y3 -NH3 -CO [1|2][1-5][1|2][3-5]	C19H21N3O3
340.1661	340.1656	0.0005	1.6	0.16	1	b3 -NH3 -CO [1|2][1-5][3|4][1-3]	C19H21N3O3
340.1661	340.1656	0.0005	1.6	0.16	1	y3 -NH3 -CO [4|5][1-5][2|3][3-5]	C19H21N3O3
342.1450	342.1448	0.0001	0.4	1.08	1	y3 -NH3 [3|4][1-6][2|3][4-6]	C18H19N3O4
342.1450	342.1448	0.0001	0.4	1.08	1	y3 -NH3 -CO [4|5][5-7]	C18H19N3O4
342.1450	342.1448	0.0001	0.4	1.08	1	y3 -COCH2 -NH3 [1|2][1-6][2|3][4-6]	C18H19N3O4
342.1450	342.1448	0.0001	0.4	1.08	1	b3 -COCH2 -NH3 [6|7][1-4][2|3][1-3]	C18H19N3O4
342.1450	342.1448	0.0001	0.4	1.08	1	b3 -COCH2 -NH3 [1|2][1-6][5|6][1-3]	C18H19N3O4
342.1450	342.1448	0.0001	0.4	1.08	1	y3 -COCH2 -NH3 [6|7][1-4][1|2][2-4]	C18H19N3O4
342.1450	342.1448	0.0001	0.4	1.08	1	b3 -NH3 [1|2][1-4][2|3][1-3]	C18H19N3O4
342.1450	342.1448	0.0001	0.4	1.08	1	b3 -NH3 [3|4][1-6][5|6][1-3]	C18H19N3O4
342.1450	342.1448	0.0001	0.4	1.08	1	b3 -NH3 -CO [1|2][1-3]	C18H19N3O4
342.1450	342.1448	0.0001	0.4	1.08	1	y3 -NH3 [1|2][1-4][1|2][2-4]	C18H19N3O4
343.1227	343.1223	0.0004	1.1	7.08	1	b2 -CO [7|1][1-2]	C17H18N4O2S
343.1227	343.1223	0.0004	1.1	7.08	1	y2 -CO [2|3][6-7]	C17H18N4O2S
349.0969	349.0965	0.0004	1.0	0.53	1	y4 -C10H9NO -NH3 [2|3][4-7]	C15H16N4O4S
349.0969	349.0965	0.0004	1.0	0.53	1	b4 -NH3 [3|4][1-5][2|3][1-4]	C15H16N4O4S
349.0969	349.0965	0.0004	1.0	0.53	1	y4 -NH3 [5|6][1-6][3|4][3-6]	C15H16N4O4S
349.0969	349.0965	0.0004	1.0	0.53	1	b4 -NH3 [5|6][1-6][5|6][1-4]	C15H16N4O4S

Meas. m/z	Calc. m/z	δ (Da)	δ (ppm)	Rel. Int. (%)	z	Annotation	Formula
349.0969	349.0965	0.0004	1.0	0.53	1	y4 -NH3 [3|4][1-5][1|2][2-5]	C15H16N4O4S
349.0969	349.0965	0.0004	1.0	0.53	1	b4 -C10H9NO -NH3 [5|6][1-4]	C15H16N4O4S
350.1284	350.1281	0.0003	0.9	0.92	1	y4 -H2O -CO [6|7][1-5][2|3][2-5]	C15H19N5O3S
350.1284	350.1281	0.0003	0.9	0.92	1	b4 -C10H9NO -H2O [4|5][1-5][2|3][1-4]	C15H19N5O3S
350.1284	350.1281	0.0003	0.9	0.92	1	b4 -H2O [6|7][1-6][4|5][1-4]	C15H19N5O3S
350.1284	350.1281	0.0003	0.9	0.92	1	b4 -C10H9NO -H2O [6|7][1-6][5|6][1-4]	C15H19N5O3S
350.1284	350.1281	0.0003	0.9	0.92	1	b4 -H2O [3|4][1-5][3|4][1-4]	C15H19N5O3S
350.1284	350.1281	0.0003	0.9	0.92	1	b4 -H2O -CO [6|7][1-5][3|4][1-4]	C15H19N5O3S
350.1284	350.1281	0.0003	0.9	0.92	1	y4 -H2O [3|4][1-5][2|3][2-5]	C15H19N5O3S
350.1284	350.1281	0.0003	0.9	0.92	1	b4 -COCH2 -H2O [7|1][1-5][2|3][1-4]	C15H19N5O3S
350.1284	350.1281	0.0003	0.9	0.92	1	y4 -H2O [6|7][1-6][2|3][3-6]	C15H19N5O3S
350.1284	350.1281	0.0003	0.9	0.92	1	y4 -COCH2 -H2O [2|3][1-6][3|4][3-6]	C15H19N5O3S
350.1284	350.1281	0.0003	0.9	0.92	1	y4 -C10H9NO -H2O [4|5][1-5][1|2][2-5]	C15H19N5O3S
350.1284	350.1281	0.0003	0.9	0.92	1	y4 -C10H9NO -H2O [6|7][1-6][3|4][3-6]	C15H19N5O3S
350.1284	350.1281	0.0003	0.9	0.92	1	y4 -COCH2 -H2O [7|1][1-5][1|2][2-5]	C15H19N5O3S
350.1284	350.1281	0.0003	0.9	0.92	1	y4 -H2O -CO [2|3][1-6][2|3][3-6]	C15H19N5O3S
350.1284	350.1281	0.0003	0.9	0.92	1	b4 -H2O -CO [2|3][1-6][4|5][1-4]	C15H19N5O3S
350.1284	350.1281	0.0003	0.9	0.92	1	b4 -COCH2 -H2O [2|3][1-6][5|6][1-4]	C15H19N5O3S
352.1442	352.1438	0.0004	1.1	8.68	1	b4 -CO [3|4][1-5][4|5][1-4]	C15H21N5O3S
352.1442	352.1438	0.0004	1.1	8.68	1	y4 -C10H9NO -CO [4|5][1-5][2|3][2-5]	C15H21N5O3S
352.1442	352.1438	0.0004	1.1	8.68	1	b4 -C10H9NO -CO [4|5][1-5][3|4][1-4]	C15H21N5O3S
352.1442	352.1438	0.0004	1.1	8.68	1	y4 -CO -COCH2 [5|6][1-5][3|4][2-5]	C15H21N5O3S
352.1442	352.1438	0.0004	1.1	8.68	1	b4 -CO -COCH2 [5|6][1-5][4|5][1-4]	C15H21N5O3S
352.1442	352.1438	0.0004	1.1	8.68	1	y4 -CO [7|1][1-6][1|2][3-6]	C15H21N5O3S
352.1442	352.1438	0.0004	1.1	8.68	1	b4 -C10H9NO -CO [7|1][1-6][4|5][1-4]	C15H21N5O3S
352.1442	352.1438	0.0004	1.1	8.68	1	b4 -CO [7|1][1-6][3|4][1-4]	C15H21N5O3S
352.1442	352.1438	0.0004	1.1	8.68	1	y4 -C10H9NO -CO [7|1][1-6][2|3][3-6]	C15H21N5O3S
352.1442	352.1438	0.0004	1.1	8.68	1	y4 -CO -COCH2 [2|3][1-6][1|2][3-6]	C15H21N5O3S
352.1442	352.1438	0.0004	1.1	8.68	1	b4 -CO -COCH2 [2|3][1-6][3|4][1-4]	C15H21N5O3S
352.1442	352.1438	0.0004	1.1	8.68	1	y4 -CO [3|4][1-5][3|4][2-5]	C15H21N5O3S
353.1071	353.1067	0.0005	1.3	0.14	1	y2 -H2O [2|3][6-7]	C18H16N4O2S
353.1071	353.1067	0.0005	1.3	0.14	1	b2 -H2O [7|1][1-2]	C18H16N4O2S
354.0909	354.0907	0.0002	0.5	0.91	1	y2 -NH3 [2|3][6-7]	C18H15N3O3S
354.0909	354.0907	0.0002	0.5	0.91	1	b2 -NH3 [7|1][1-2]	C18H15N3O3S
361.0970	361.0965	0.0005	1.3	2.47	1	y4 -H2O -NH3 [6|7][1-5][2|3][2-5]	C16H16N4O4S
361.0970	361.0965	0.0005	1.3	2.47	1	y4 -H2O -NH3 [2|3][1-6][2|3][3-6]	C16H16N4O4S
361.0970	361.0965	0.0005	1.3	2.47	1	b4 -H2O -NH3 [6|7][1-5][3|4][1-4]	C16H16N4O4S
361.0970	361.0965	0.0005	1.3	2.47	1	b4 -H2O -NH3 [2|3][1-6][4|5][1-4]	C16H16N4O4S
364.1443	364.1438	0.0005	1.5	1.44	1	y4 -H2O -CO [2|3][1-6][3|4][3-6]	C16H21N5O3S
364.1443	364.1438	0.0005	1.5	1.44	1	b4 -H2O -CO [7|1][1-5][2|3][1-4]	C16H21N5O3S
364.1443	364.1438	0.0005	1.5	1.44	1	b4 -H2O -CO [2|3][1-6][5|6][1-4]	C16H21N5O3S
364.1443	364.1438	0.0005	1.5	1.44	1	y4 -H2O -CO [7|1][1-5][1|2][2-5]	C16H21N5O3S
366.1235	366.1231	0.0005	1.3	0.39	1	y4 -C10H9NO -COCH2 [7|1][1-6][3|4][3-6]	C15H19N5O4S
366.1235	366.1231	0.0005	1.3	0.39	1	b4 -C10H9NO -COCH2 [7|1][1-6][5|6][1-4]	C15H19N5O4S
366.1235	366.1231	0.0005	1.3	0.39	1	y4 -C10H9NO [2|3][4-7]	C15H19N5O4S
366.1235	366.1231	0.0005	1.3	0.39	1	y4 [3|4][1-5][1|2][2-5]	C15H19N5O4S
366.1235	366.1231	0.0005	1.3	0.39	1	y4 [5|6][1-6][3|4][3-6]	C15H19N5O4S
366.1235	366.1231	0.0005	1.3	0.39	1	b4 [5|6][1-6][5|6][1-4]	C15H19N5O4S

Meas. m/z	Calc. m/z	δ (Da)	δ (ppm)	Rel. Int. (%)	z	Annotation	Formula
366.1235	366.1231	0.0005	1.3	0.39	1	b4 [3|4][1-5][2|3][1-4]	C15H19N5O4S
366.1235	366.1231	0.0005	1.3	0.39	1	b4 -C10H9NO [5|6][1-4]	C15H19N5O4S
366.1235	366.1231	0.0005	1.3	0.39	1	y4 -C10H9NO -COCH2 [5|6][1-5][1|2][2-5]	C15H19N5O4S
366.1235	366.1231	0.0005	1.3	0.39	1	b4 -C10H9NO -COCH2 [5|6][1-5][2|3][1-4]	C15H19N5O4S
366.1598	366.1594	0.0003	0.9	0.15	1	b4 -CO [4|5][1-4]	C16H23N5O3S
366.1598	366.1594	0.0003	0.9	0.15	1	y4 -CO [1|2][4-7]	C16H23N5O3S
370.1400	370.1397	0.0003	0.8	0.86	1	y3 -NH3 [4|5][5-7]	C19H19N3O5
370.1400	370.1397	0.0003	0.8	0.86	1	b3 -NH3 [1|2][1-3]	C19H19N3O5
371.1176	371.1172	0.0003	0.9	3.14	1	b2 [7|1][1-2]	C18H18N4O3S
371.1176	371.1172	0.0003	0.9	3.14	1	y2 [2|3][6-7]	C18H18N4O3S
377.1283	377.1278	0.0005	1.2	0.57	1	y4 -NH3 [1|2][4-7]	C17H20N4O4S
377.1283	377.1278	0.0005	1.2	0.57	1	b4 -NH3 [4|5][1-4]	C17H20N4O4S
377.1283	377.1278	0.0005	1.2	0.57	1	y4 -NH3 -CO [5|6][1-5][3|4][2-5]	C17H20N4O4S
377.1283	377.1278	0.0005	1.2	0.57	1	y4 -NH3 -CO [2|3][1-6][1|2][3-6]	C17H20N4O4S
377.1283	377.1278	0.0005	1.2	0.57	1	b4 -NH3 -CO [2|3][1-6][3|4][1-4]	C17H20N4O4S
377.1283	377.1278	0.0005	1.2	0.57	1	b4 -NH3 -CO [5|6][1-5][4|5][1-4]	C17H20N4O4S
382.2089	382.2085	0.0004	1.1	0.25	1	b5 -C10H9NO -CO [4|5][1-6][4|5][1-5]	C17H27N5O5
382.2089	382.2085	0.0004	1.1	0.25	1	y5 -C10H9NO -CO [4|5][1-6][3|4][2-6]	C17H27N5O5
382.2089	382.2085	0.0004	1.1	0.25	1	b5 -C10H9NO -CO [1|2][1-6][3|4][1-5]	C17H27N5O5
382.2089	382.2085	0.0004	1.1	0.25	1	y5 -C10H9NO -CO [1|2][1-6][2|3][2-6]	C17H27N5O5
382.2089	382.2085	0.0004	1.1	0.25	1	b5 -CO [2|3][1-5]	C17H27N5O5
382.2089	382.2085	0.0004	1.1	0.25	1	y5 -CO [7|1][3-7]	C17H27N5O5
383.1177	383.1172	0.0004	1.1	0.23	1	b3 -NH3 -CO [3|4][1-6][4|5][1-3]	C19H18N4O3S
383.1177	383.1172	0.0004	1.1	0.23	1	b3 -NH3 -CO [7|1][1-4][3|4][1-3]	C19H18N4O3S
383.1177	383.1172	0.0004	1.1	0.23	1	y3 -NH3 -CO [3|4][1-6][1|2][4-6]	C19H18N4O3S
383.1177	383.1172	0.0004	1.1	0.23	1	y3 -NH3 -CO [7|1][1-4][2|3][2-4]	C19H18N4O3S
392.1935	392.1928	0.0007	1.7	0.26	1	b5 -H2O [2|3][1-5]	C18H25N5O5
392.1935	392.1928	0.0007	1.7	0.26	1	y5 -C10H9NO -H2O [4|5][1-6][3|4][2-6]	C18H25N5O5
392.1935	392.1928	0.0007	1.7	0.26	1	y5 -C10H9NO -H2O [1|2][1-6][2|3][2-6]	C18H25N5O5
392.1935	392.1928	0.0007	1.7	0.26	1	y5 -H2O [7|1][3-7]	C18H25N5O5
392.1935	392.1928	0.0007	1.7	0.26	1	b5 -C10H9NO -H2O [4|5][1-6][4|5][1-5]	C18H25N5O5
392.1935	392.1928	0.0007	1.7	0.26	1	b5 -C10H9NO -H2O [1|2][1-6][3|4][1-5]	C18H25N5O5
395.1501	395.1496	0.0005	1.2	0.51	1	b5 -C10H9NO -CO [3|4][1-6][2|3][1-5]	C16H22N6O4S
395.1501	395.1496	0.0005	1.2	0.51	1	b5 -C10H9NO -CO [5|6][1-6][5|6][1-5]	C16H22N6O4S
395.1501	395.1496	0.0005	1.2	0.51	1	y5 -C10H9NO -CO [3|4][1-6][1|2][2-6]	C16H22N6O4S
395.1501	395.1496	0.0005	1.2	0.51	1	y5 -C10H9NO -CO [5|6][1-6][4|5][2-6]	C16H22N6O4S
396.1341	396.1336	0.0005	1.2	2.09	1	b4 -C10H9NO [6|7][1-4]	C16H21N5O5S
396.1341	396.1336	0.0005	1.2	2.09	1	y4 -C10H9NO [3|4][4-7]	C16H21N5O5S
396.1341	396.1336	0.0005	1.2	2.09	1	y4 [6|7][1-5][2|3][2-5]	C16H21N5O5S
396.1341	396.1336	0.0005	1.2	2.09	1	b4 [2|3][1-6][4|5][1-4]	C16H21N5O5S
396.1341	396.1336	0.0005	1.2	2.09	1	b4 [6|7][1-5][3|4][1-4]	C16H21N5O5S
396.1341	396.1336	0.0005	1.2	2.09	1	y4 [2|3][1-6][2|3][3-6]	C16H21N5O5S
396.1493	396.1489	0.0004	1.1	36.12	1	b3 -H2O -CO [6|7][1-3]	C20H21N5O2S
396.1493	396.1489	0.0004	1.1	36.12	1	y3 -H2O -CO [2|3][5-7]	C20H21N5O2S
397.1335	397.1329	0.0007	1.7	0.25	1	y3 -NH3 -CO [2|3][5-7]	C20H20N4O3S
397.1335	397.1329	0.0007	1.7	0.25	1	b3 -NH3 -CO [6|7][1-3]	C20H20N4O3S
406.1544	406.1544	0.0001	0.2	0.25	1	y5 -NH3 -CO [1|2][3-7]	C18H23N5O4S
406.1544	406.1544	0.0001	0.2	0.25	1	b5 -NH3 -CO [3|4][1-5]	C18H23N5O4S

Meas. m/z	Calc. m/z	δ (Da)	δ (ppm)	Rel. Int. (%)	z	Annotation	Formula
407.1505	407.1496	0.0009	2.3	0.14	1	b5 -C10H9NO -H2O [6|7][1-6][4|5][1-5]	C17H22N6O4S
407.1505	407.1496	0.0009	2.3	0.14	1	b5 -C10H9NO -H2O [3|4][1-6][3|4][1-5]	C17H22N6O4S
407.1505	407.1496	0.0009	2.3	0.14	1	y5 -C10H9NO -H2O [3|4][1-6][2|3][2-6]	C17H22N6O4S
407.1505	407.1496	0.0009	2.3	0.14	1	y5 -C10H9NO -H2O [6|7][1-6][3|4][2-6]	C17H22N6O4S
407.1721	407.1714	0.0007	1.7	0.28	1	b4 -H2O -NH3 [3|4][1-6][5|6][1-4]	C22H22N4O4
407.1721	407.1714	0.0007	1.7	0.28	1	y4 -H2O -NH3 [3|4][1-6][3|4][3-6]	C22H22N4O4
407.1721	407.1714	0.0007	1.7	0.28	1	y4 -H2O -NH3 [1|2][1-5][1|2][2-5]	C22H22N4O4
407.1721	407.1714	0.0007	1.7	0.28	1	b4 -H2O -NH3 [1|2][1-5][2|3][1-4]	C22H22N4O4
408.1343	408.1336	0.0007	1.7	0.58	1	y5 -C10H9NO -NH3 [3|4][1-6][2|3][2-6]	C17H21N5O5S
408.1343	408.1336	0.0007	1.7	0.58	1	y4 -C10H9NO [7|1][1-6][3|4][3-6]	C17H21N5O5S
408.1343	408.1336	0.0007	1.7	0.58	1	y5 -C10H9NO -NH3 [6|7][1-6][3|4][2-6]	C17H21N5O5S
408.1343	408.1336	0.0007	1.7	0.58	1	b4 -C10H9NO [7|1][1-6][5|6][1-4]	C17H21N5O5S
408.1343	408.1336	0.0007	1.7	0.58	1	b5 -C10H9NO -NH3 [3|4][1-6][3|4][1-5]	C17H21N5O5S
408.1343	408.1336	0.0007	1.7	0.58	1	b4 -C10H9NO [5|6][1-5][2|3][1-4]	C17H21N5O5S
408.1343	408.1336	0.0007	1.7	0.58	1	y4 -C10H9NO [5|6][1-5][1|2][2-5]	C17H21N5O5S
408.1343	408.1336	0.0007	1.7	0.58	1	b5 -C10H9NO -NH3 [6|7][1-6][4|5][1-5]	C17H21N5O5S
410.2037	410.2034	0.0003	0.7	1.47	1	y5 -C10H9NO [4|5][1-6][3|4][2-6]	C18H27N5O6
410.2037	410.2034	0.0003	0.7	1.47	1	b5 -C10H9NO [4|5][1-6][4|5][1-5]	C18H27N5O6
410.2037	410.2034	0.0003	0.7	1.47	1	y5 [7|1][3-7]	C18H27N5O6
410.2037	410.2034	0.0003	0.7	1.47	1	y5 -C10H9NO [1|2][1-6][2|3][2-6]	C18H27N5O6
410.2037	410.2034	0.0003	0.7	1.47	1	b5 [2|3][1-5]	C18H27N5O6
410.2037	410.2034	0.0003	0.7	1.47	1	b5 -C10H9NO [1|2][1-6][3|4][1-5]	C18H27N5O6
411.1122	411.1122	0.0001	0.1	0.25	1	b3 -NH3 [7|1][1-4][3|4][1-3]	C20H18N4O4S
411.1122	411.1122	0.0001	0.1	0.25	1	y3 -NH3 [7|1][1-4][2|3][2-4]	C20H18N4O4S
411.1122	411.1122	0.0001	0.1	0.25	1	y3 -NH3 [3|4][1-6][1|2][4-6]	C20H18N4O4S
411.1122	411.1122	0.0001	0.1	0.25	1	b3 -NH3 [3|4][1-6][4|5][1-3]	C20H18N4O4S
411.1492	411.1485	0.0006	1.5	2.40	1	y3 -NH3 -CO [4|5][1-5][1|2][3-5]	C21H22N4O3S
411.1492	411.1485	0.0006	1.5	2.40	1	b3 -NH3 -CO [4|5][1-5][3|4][1-3]	C21H22N4O3S
411.1492	411.1485	0.0006	1.5	2.40	1	y3 -NH3 -CO [7|1][1-5][2|3][3-5]	C21H22N4O3S
411.1492	411.1485	0.0006	1.5	2.40	1	b3 -NH3 -CO [7|1][1-5][4|5][1-3]	C21H22N4O3S
413.1826	413.1819	0.0006	1.5	0.61	1	b3 [1|2][1-5][4|5][1-3]	C21H24N4O5
413.1826	413.1819	0.0006	1.5	0.61	1	y3 [5|6][1-5][1|2][3-5]	C21H24N4O5
413.1826	413.1819	0.0006	1.5	0.61	1	b3 [5|6][1-5][3|4][1-3]	C21H24N4O5
413.1826	413.1819	0.0006	1.5	0.61	1	b4 -NH3 -CO [6|7][1-5][2|3][1-4]	C21H24N4O5
413.1826	413.1819	0.0006	1.5	0.61	1	y4 -NH3 -CO [6|7][1-5][1|2][2-5]	C21H24N4O5
413.1826	413.1819	0.0006	1.5	0.61	1	y3 [1|2][1-5][2|3][3-5]	C21H24N4O5
413.1826	413.1819	0.0006	1.5	0.61	1	b4 -NH3 -CO [1|2][1-6][5|6][1-4]	C21H24N4O5
413.1826	413.1819	0.0006	1.5	0.61	1	y4 -NH3 -CO [1|2][1-6][3|4][3-6]	C21H24N4O5
413.1826	413.1819	0.0006	1.5	0.61	1	y4 -COCH2 -NH3 [5|6][4-7]	C21H24N4O5
413.1826	413.1819	0.0006	1.5	0.61	1	b4 -COCH2 -NH3 [1|2][1-4]	C21H24N4O5
414.1599	414.1594	0.0005	1.2	60.14	1	y3 -CO [2|3][5-7]	C20H23N5O3S
414.1599	414.1594	0.0005	1.2	60.14	1	y3 -CO -COCH2 [3|4][5-7]	C20H23N5O3S
414.1599	414.1594	0.0005	1.2	60.14	1	b3 -CO [6|7][1-3]	C20H23N5O3S
414.1599	414.1594	0.0005	1.2	60.14	1	b3 -CO -COCH2 [7|1][1-3]	C20H23N5O3S
421.1655	421.1653	0.0002	0.5	0.26	1	y5 -COCH2 -H2O [2|3][1-6][3|4][2-6]	C18H24N6O4S
421.1655	421.1653	0.0002	0.5	0.26	1	y5 -COCH2 -H2O [6|7][1-6][2|3][2-6]	C18H24N6O4S
421.1655	421.1653	0.0002	0.5	0.26	1	b5 -COCH2 -H2O [2|3][1-6][4|5][1-5]	C18H24N6O4S
421.1655	421.1653	0.0002	0.5	0.26	1	b5 -COCH2 -H2O [6|7][1-6][3|4][1-5]	C18H24N6O4S

Meas. m/z	Calc. m/z	δ (Da)	δ (ppm)	Rel. Int. (%)	z	Annotation	Formula
423.1447	423.1445	0.0002	0.4	3.59	1	b5 -C10H9NO -COCH2 [7|1][1-6][5|6][1-5]	C17H22N6O5S
423.1447	423.1445	0.0002	0.4	3.59	1	y5 -C10H9NO [5|6][1-6][4|5][2-6]	C17H22N6O5S
423.1447	423.1445	0.0002	0.4	3.59	1	b5 -C10H9NO [3|4][1-6][2|3][1-5]	C17H22N6O5S
423.1447	423.1445	0.0002	0.4	3.59	1	y5 -C10H9NO -COCH2 [5|6][1-6][1|2][2-6]	C17H22N6O5S
423.1447	423.1445	0.0002	0.4	3.59	1	y5 -C10H9NO [3|4][1-6][1|2][2-6]	C17H22N6O5S
423.1447	423.1445	0.0002	0.4	3.59	1	b5 -C10H9NO [5|6][1-6][5|6][1-5]	C17H22N6O5S
423.1447	423.1445	0.0002	0.4	3.59	1	b5 -C10H9NO -COCH2 [5|6][1-6][2|3][1-5]	C17H22N6O5S
423.1447	423.1445	0.0002	0.4	3.59	1	y5 -C10H9NO -COCH2 [7|1][1-6][4|5][2-6]	C17H22N6O5S
424.1445	424.1438	0.0007	1.6	0.35	1	b3 -COCH2 -H2O [7|1][1-3]	C21H21N5O3S
424.1445	424.1438	0.0007	1.6	0.35	1	y3 -H2O [2|3][5-7]	C21H21N5O3S
424.1445	424.1438	0.0007	1.6	0.35	1	b3 -H2O [6|7][1-3]	C21H21N5O3S
424.1445	424.1438	0.0007	1.6	0.35	1	y3 -COCH2 -H2O [3|4][5-7]	C21H21N5O3S
425.1824	425.1819	0.0005	1.1	1.63	1	b4 -NH3 -CO [1|2][1-5][4|5][1-4]	C22H24N4O5
425.1824	425.1819	0.0005	1.1	1.63	1	b4 -NH3 [1|2][1-5][2|3][1-4]	C22H24N4O5
425.1824	425.1819	0.0005	1.1	1.63	1	b4 -NH3 [3|4][1-6][5|6][1-4]	C22H24N4O5
425.1824	425.1819	0.0005	1.1	1.63	1	y4 -NH3 -CO [1|2][1-5][3|4][2-5]	C22H24N4O5
425.1824	425.1819	0.0005	1.1	1.63	1	y4 -COCH2 -NH3 [1|2][1-6][2|3][3-6]	C22H24N4O5
425.1824	425.1819	0.0005	1.1	1.63	1	y4 -NH3 [3|4][1-6][3|4][3-6]	C22H24N4O5
425.1824	425.1819	0.0005	1.1	1.63	1	y4 -NH3 [1|2][1-5][1|2][2-5]	C22H24N4O5
425.1824	425.1819	0.0005	1.1	1.63	1	y4 -COCH2 -NH3 [5|6][1-5][2|3][2-5]	C22H24N4O5
425.1824	425.1819	0.0005	1.1	1.63	1	b4 -COCH2 -NH3 [1|2][1-6][4|5][1-4]	C22H24N4O5
425.1824	425.1819	0.0005	1.1	1.63	1	b4 -COCH2 -NH3 [5|6][1-5][3|4][1-4]	C22H24N4O5
425.1824	425.1819	0.0005	1.1	1.63	1	y4 -NH3 -CO [5|6][1-6][1|2][3-6]	C22H24N4O5
425.1824	425.1819	0.0005	1.1	1.63	1	b4 -NH3 -CO [5|6][1-6][3|4][1-4]	C22H24N4O5
427.1980	427.1976	0.0004	1.0	2.25	1	y4 -NH3 -CO [5|6][4-7]	C22H26N4O5
427.1980	427.1976	0.0004	1.0	2.25	1	b4 -NH3 -CO [1|2][1-4]	C22H26N4O5
435.1448	435.1445	0.0003	0.7	1.28	1	y5 -C10H9NO -H2O [4|5][3-7]	C18H22N6O5S
435.1448	435.1445	0.0003	0.7	1.28	1	b5 -C10H9NO -H2O [6|7][1-5]	C18H22N6O5S
435.1813	435.1809	0.0004	0.9	3.20	1	y5 -H2O -CO [6|7][1-6][2|3][2-6]	C19H26N6O4S
435.1813	435.1809	0.0004	0.9	3.20	1	b5 -H2O -CO [2|3][1-6][4|5][1-5]	C19H26N6O4S
435.1813	435.1809	0.0004	0.9	3.20	1	y5 -H2O -CO [2|3][1-6][3|4][2-6]	C19H26N6O4S
435.1813	435.1809	0.0004	0.9	3.20	1	b5 -H2O -CO [6|7][1-6][3|4][1-5]	C19H26N6O4S
437.1603	437.1602	0.0002	0.4	0.95	1	y5 -C10H9NO -COCH2 [3|4][3-7]	C18H24N6O5S
437.1603	437.1602	0.0002	0.4	0.95	1	b5 -C10H9NO -COCH2 [5|6][1-5]	C18H24N6O5S
437.1603	437.1602	0.0002	0.4	0.95	1	y5 -C10H9NO -CO [7|1][1-6][4|5][2-6]	C18H24N6O5S
437.1603	437.1602	0.0002	0.4	0.95	1	y5 -C10H9NO [7|1][1-6][2|3][2-6]	C18H24N6O5S
437.1603	437.1602	0.0002	0.4	0.95	1	b5 -C10H9NO -CO [7|1][1-6][5|6][1-5]	C18H24N6O5S
437.1603	437.1602	0.0002	0.4	0.95	1	y5 -C10H9NO [3|4][1-6][3|4][2-6]	C18H24N6O5S
437.1603	437.1602	0.0002	0.4	0.95	1	b5 -C10H9NO [3|4][1-6][4|5][1-5]	C18H24N6O5S
437.1603	437.1602	0.0002	0.4	0.95	1	b5 -COCH2 [2|3][1-6][3|4][1-5]	C18H24N6O5S
437.1603	437.1602	0.0002	0.4	0.95	1	b5 -C10H9NO -CO [5|6][1-6][2|3][1-5]	C18H24N6O5S
437.1603	437.1602	0.0002	0.4	0.95	1	y5 -C10H9NO -CO [5|6][1-6][1|2][2-6]	C18H24N6O5S
437.1603	437.1602	0.0002	0.4	0.95	1	y5 -COCH2 [2|3][1-6][2|3][2-6]	C18H24N6O5S
437.1603	437.1602	0.0002	0.4	0.95	1	b5 -C10H9NO [7|1][1-6][3|4][1-5]	C18H24N6O5S
437.1603	437.1602	0.0002	0.4	0.95	1	b5 -COCH2 [5|6][1-6][4|5][1-5]	C18H24N6O5S
437.1603	437.1602	0.0002	0.4	0.95	1	y5 -COCH2 [5|6][1-6][3|4][2-6]	C18H24N6O5S
442.1549	442.1544	0.0005	1.2	41.13	1	y3 -COCH2 [3|4][5-7]	C21H23N5O4S
442.1549	442.1544	0.0005	1.2	41.13	1	b3 -COCH2 [7|1][1-3]	C21H23N5O4S

Meas. m/z	Calc. m/z	δ (Da)	δ (ppm)	Rel. Int. (%)	z	Annotation	Formula
442.1549	442.1544	0.0005	1.2	41.13	1	b3 [6|7][1-3]	C21H23N5O4S
442.1549	442.1544	0.0005	1.2	41.13	1	y3 [2|3][5-7]	C21H23N5O4S
447.1451	447.1445	0.0005	1.2	0.58	1	y5 -C10H9NO -H2O [5|6][1-6][1|2][2-6]	C19H22N6O5S
447.1451	447.1445	0.0005	1.2	0.58	1	y5 -C10H9NO -H2O [7|1][1-6][4|5][2-6]	C19H22N6O5S
447.1451	447.1445	0.0005	1.2	0.58	1	b5 -C10H9NO -H2O [7|1][1-6][5|6][1-5]	C19H22N6O5S
447.1451	447.1445	0.0005	1.2	0.58	1	b5 -C10H9NO -H2O [5|6][1-6][2|3][1-5]	C19H22N6O5S
447.1814	447.1809	0.0005	1.0	0.21	1	y5 -COCH2 -H2O [4|5][1-6][4|5][2-6]	C20H26N6O4S
447.1814	447.1809	0.0005	1.0	0.21	1	b5 -H2O -CO [2|3][1-6][5|6][1-5]	C20H26N6O4S
447.1814	447.1809	0.0005	1.0	0.21	1	b5 -H2O -CO [7|1][1-6][2|3][1-5]	C20H26N6O4S
447.1814	447.1809	0.0005	1.0	0.21	1	b5 -COCH2 -H2O [4|5][1-6][5|6][1-5]	C20H26N6O4S
447.1814	447.1809	0.0005	1.0	0.21	1	y5 -H2O -CO [7|1][1-6][1|2][2-6]	C20H26N6O4S
447.1814	447.1809	0.0005	1.0	0.21	1	b5 -COCH2 -H2O [2|3][1-6][2|3][1-5]	C20H26N6O4S
447.1814	447.1809	0.0005	1.0	0.21	1	y5 -COCH2 -H2O [2|3][1-6][1|2][2-6]	C20H26N6O4S
447.1814	447.1809	0.0005	1.0	0.21	1	y5 -H2O -CO [2|3][1-6][4|5][2-6]	C20H26N6O4S
449.1596	449.1602	-0.0006	-1.2	0.13	1	y5 -C10H9NO -H2O [5|6][3-7]	C19H24N6O5S
449.1596	449.1602	-0.0006	-1.2	0.13	1	b5 -C10H9NO -H2O [7|1][1-5]	C19H24N6O5S
453.1555	453.1551	0.0005	1.0	2.05	1	b5 -C10H9NO [6|7][1-5]	C18H24N6O6S
453.1555	453.1551	0.0005	1.0	2.05	1	y5 -C10H9NO [4|5][3-7]	C18H24N6O6S
454.1550	454.1544	0.0006	1.4	0.26	1	b3 [5|6][1-4][2|3][1-3]	C22H23N5O4S
454.1550	454.1544	0.0006	1.4	0.26	1	b3 [7|1][1-6][5|6][1-3]	C22H23N5O4S
454.1550	454.1544	0.0006	1.4	0.26	1	y3 [5|6][1-4][1|2][2-4]	C22H23N5O4S
454.1550	454.1544	0.0006	1.4	0.26	1	y3 [7|1][1-6][2|3][4-6]	C22H23N5O4S
454.1550	454.1544	0.0006	1.4	0.26	1	b4 -NH3 -CO [3|4][1-6][3|4][1-4]	C22H23N5O4S
454.1550	454.1544	0.0006	1.4	0.26	1	y4 -NH3 -CO [6|7][1-5][3|4][2-5]	C22H23N5O4S
454.1550	454.1544	0.0006	1.4	0.26	1	b4 -NH3 -CO [6|7][1-5][4|5][1-4]	C22H23N5O4S
454.1550	454.1544	0.0006	1.4	0.26	1	y4 -NH3 -CO [3|4][1-6][1|2][3-6]	C22H23N5O4S
461.1608	461.1602	0.0006	1.3	0.47	1	b5 -C10H9NO -H2O [5|6][1-5]	C20H24N6O5S
461.1608	461.1602	0.0006	1.3	0.47	1	b5 -H2O [5|6][1-6][4|5][1-5]	C20H24N6O5S
461.1608	461.1602	0.0006	1.3	0.47	1	y5 -H2O [2|3][1-6][2|3][2-6]	C20H24N6O5S
461.1608	461.1602	0.0006	1.3	0.47	1	b5 -H2O [2|3][1-6][3|4][1-5]	C20H24N6O5S
461.1608	461.1602	0.0006	1.3	0.47	1	y5 -C10H9NO -H2O [3|4][3-7]	C20H24N6O5S
461.1608	461.1602	0.0006	1.3	0.47	1	y5 -H2O [5|6][1-6][3|4][2-6]	C20H24N6O5S
462.1448	462.1442	0.0006	1.4	1.03	1	b5 -C10H9NO -NH3 [5|6][1-5]	C20H23N5O6S
462.1448	462.1442	0.0006	1.4	1.03	1	y5 -NH3 [5|6][1-6][3|4][2-6]	C20H23N5O6S
462.1448	462.1442	0.0006	1.4	1.03	1	b5 -NH3 [2|3][1-6][3|4][1-5]	C20H23N5O6S
462.1448	462.1442	0.0006	1.4	1.03	1	b5 -NH3 [5|6][1-6][4|5][1-5]	C20H23N5O6S
462.1448	462.1442	0.0006	1.4	1.03	1	y5 -NH3 [2|3][1-6][2|3][2-6]	C20H23N5O6S
462.1448	462.1442	0.0006	1.4	1.03	1	y5 -C10H9NO -NH3 [3|4][3-7]	C20H23N5O6S
467.1387	467.1384	0.0003	0.7	0.39	1	b3 -NH3 [7|1][1-3]	C23H22N4O5S
467.1387	467.1384	0.0003	0.7	0.39	1	y3 -NH3 [3|4][5-7]	C23H22N4O5S
471.1803	471.1809	-0.0006	-1.2	0.14	1	b4 -CO [6|7][1-5][4|5][1-4]	C22H26N6O4S
471.1803	471.1809	-0.0006	-1.2	0.14	1	y4 -CO -COCH2 [4|5][4-7]	C22H26N6O4S
471.1803	471.1809	-0.0006	-1.2	0.14	1	b4 -CO -COCH2 [7|1][1-4]	C22H26N6O4S
471.1803	471.1809	-0.0006	-1.2	0.14	1	y4 -CO [3|4][1-6][1|2][3-6]	C22H26N6O4S
471.1803	471.1809	-0.0006	-1.2	0.14	1	y4 -CO [6|7][1-5][3|4][2-5]	C22H26N6O4S
471.1803	471.1809	-0.0006	-1.2	0.14	1	b4 -CO [3|4][1-6][3|4][1-4]	C22H26N6O4S
479.1714	479.1707	0.0007	1.4	1.17	1	b5 [5|6][1-6][4|5][1-5]	C20H26N6O6S
479.1714	479.1707	0.0007	1.4	1.17	1	b5 [2|3][1-6][3|4][1-5]	C20H26N6O6S

Meas. m/z	Calc. m/z	δ (Da)	δ (ppm)	Rel. Int. (%)	z	Annotation	Formula
479.1714	479.1707	0.0007	1.4	1.17	1	y5 -C10H9NO [3|4][3-7]	C20H26N6O6S
479.1714	479.1707	0.0007	1.4	1.17	1	y5 [5|6][1-6][3|4][2-6]	C20H26N6O6S
479.1714	479.1707	0.0007	1.4	1.17	1	y5 [2|3][1-6][2|3][2-6]	C20H26N6O6S
479.1714	479.1707	0.0007	1.4	1.17	1	b5 -C10H9NO [5|6][1-5]	C20H26N6O6S
479.1865	479.1860	0.0005	1.0	6.68	1	b4 -H2O -CO [5|6][1-4]	C24H26N6O3S
479.1865	479.1860	0.0005	1.0	6.68	1	y4 -H2O -CO [2|3][4-7]	C24H26N6O3S
480.1705	480.1700	0.0005	1.1	29.46	1	y4 -NH3 -CO [2|3][4-7]	C24H25N5O4S
480.1705	480.1700	0.0005	1.1	29.46	1	b4 -NH3 -CO [5|6][1-4]	C24H25N5O4S
482.1859	482.1857	0.0003	0.6	0.84	1	b4 -NH3 -CO [4|5][1-5][2|3][1-4]	C24H27N5O4S
482.1859	482.1857	0.0003	0.6	0.84	1	y4 -NH3 -CO [6|7][1-6][3|4][3-6]	C24H27N5O4S
482.1859	482.1857	0.0003	0.6	0.84	1	b4 -NH3 -CO [6|7][1-6][5|6][1-4]	C24H27N5O4S
482.1859	482.1857	0.0003	0.6	0.84	1	y4 -NH3 -CO [4|5][1-5][1|2][2-5]	C24H27N5O4S
484.1653	484.1649	0.0004	0.8	0.77	1	b3 [7|1][1-3]	C23H25N5O5S
484.1653	484.1649	0.0004	0.8	0.77	1	y3 [3|4][5-7]	C23H25N5O5S
485.1968	485.1966	0.0003	0.6	0.14	1	b4 -CO [7|1][1-5][3|4][1-4]	C23H28N6O4S
485.1968	485.1966	0.0003	0.6	0.14	1	b4 -CO [3|4][1-6][4|5][1-4]	C23H28N6O4S
485.1968	485.1966	0.0003	0.6	0.14	1	y4 -CO [3|4][1-6][2|3][3-6]	C23H28N6O4S
485.1968	485.1966	0.0003	0.6	0.14	1	y4 -CO -COCH2 [3|4][4-7]	C23H28N6O4S
485.1968	485.1966	0.0003	0.6	0.14	1	b4 -CO -COCH2 [6|7][1-4]	C23H28N6O4S
485.1968	485.1966	0.0003	0.6	0.14	1	y4 -CO [7|1][1-5][2|3][2-5]	C23H28N6O4S
490.1873	490.1867	0.0005	1.1	0.28	1	y6 -C10H9NO -H2O [2|3][2-7]	C21H27N7O5S
490.1873	490.1867	0.0005	1.1	0.28	1	b6 -C10H9NO -H2O [3|4][1-6]	C21H27N7O5S
491.1711	491.1707	0.0004	0.7	0.76	1	y6 -C10H9NO -NH3 [2|3][2-7]	C21H26N6O6S
491.1711	491.1707	0.0004	0.7	0.76	1	b6 -C10H9NO -NH3 [3|4][1-6]	C21H26N6O6S
493.2024	493.2016	0.0008	1.5	0.29	1	b4 -H2O -CO [4|5][1-5][3|4][1-4]	C25H28N6O3S
493.2024	493.2016	0.0008	1.5	0.29	1	b4 -H2O -CO [7|1][1-6][4|5][1-4]	C25H28N6O3S
493.2024	493.2016	0.0008	1.5	0.29	1	y4 -H2O -CO [7|1][1-6][2|3][3-6]	C25H28N6O3S
493.2024	493.2016	0.0008	1.5	0.29	1	y4 -H2O -CO [4|5][1-5][2|3][2-5]	C25H28N6O3S
494.1864	494.1857	0.0007	1.5	0.51	1	b4 -NH3 -CO [7|1][1-6][4|5][1-4]	C25H27N5O4S
494.1864	494.1857	0.0007	1.5	0.51	1	b4 -NH3 -CO [4|5][1-5][3|4][1-4]	C25H27N5O4S
494.1864	494.1857	0.0007	1.5	0.51	1	y4 -NH3 -CO [7|1][1-6][2|3][3-6]	C25H27N5O4S
494.1864	494.1857	0.0007	1.5	0.51	1	y4 -NH3 -CO [4|5][1-5][2|3][2-5]	C25H27N5O4S
496.1660	496.1649	0.0011	2.3	0.14	1	y4 -NH3 [3|4][1-6][2|3][3-6]	C24H25N5O5S
496.1660	496.1649	0.0011	2.3	0.14	1	y4 -NH3 [7|1][1-5][2|3][2-5]	C24H25N5O5S
496.1660	496.1649	0.0011	2.3	0.14	1	b4 -NH3 [7|1][1-5][3|4][1-4]	C24H25N5O5S
496.1660	496.1649	0.0011	2.3	0.14	1	b4 -NH3 [3|4][1-6][4|5][1-4]	C24H25N5O5S
496.1660	496.1649	0.0011	2.3	0.14	1	y4 -NH3 -CO [4|5][4-7]	C24H25N5O5S
496.1660	496.1649	0.0011	2.3	0.14	1	b4 -COCH2 -NH3 [6|7][1-4]	C24H25N5O5S
496.1660	496.1649	0.0011	2.3	0.14	1	y4 -COCH2 -NH3 [3|4][4-7]	C24H25N5O5S
496.1660	496.1649	0.0011	2.3	0.14	1	b4 -NH3 -CO [7|1][1-4]	C24H25N5O5S
496.2204	496.2191	0.0013	2.7	0.28	1	b5 -NH3 [1|2][1-6][2|3][1-5]	C25H29N5O6
496.2204	496.2191	0.0013	2.7	0.28	1	b5 -COCH2 -NH3 [1|2][1-5]	C25H29N5O6
496.2204	496.2191	0.0013	2.7	0.28	1	y5 -NH3 -CO [5|6][1-6][2|3][2-6]	C25H29N5O6
496.2204	496.2191	0.0013	2.7	0.28	1	y5 -NH3 -CO [1|2][1-6][3|4][2-6]	C25H29N5O6
496.2204	496.2191	0.0013	2.7	0.28	1	y5 -NH3 [3|4][1-6][4|5][2-6]	C25H29N5O6
496.2204	496.2191	0.0013	2.7	0.28	1	b5 -NH3 [3|4][1-6][5|6][1-5]	C25H29N5O6
496.2204	496.2191	0.0013	2.7	0.28	1	b5 -NH3 -CO [5|6][1-6][3|4][1-5]	C25H29N5O6
496.2204	496.2191	0.0013	2.7	0.28	1	y5 -NH3 [1|2][1-6][1|2][2-6]	C25H29N5O6

Meas. m/z	Calc. m/z	δ (Da)	δ (ppm)	Rel. Int. (%)	z	Annotation	Formula
496.2204	496.2191	0.0013	2.7	0.28	1	b5 -NH3 -CO [1|2][1-6][4|5][1-5]	C25H29N5O6
496.2204	496.2191	0.0013	2.7	0.28	1	y5 -COCH2 -NH3 [6|7][3-7]	C25H29N5O6
497.1971	497.1966	0.0006	1.2	12.27	1	b4 -CO -COCH2 [7|1][1-6][5|6][1-4]	C24H28N6O4S
497.1971	497.1966	0.0006	1.2	12.27	1	y4 -CO -COCH2 [7|1][1-6][3|4][3-6]	C24H28N6O4S
497.1971	497.1966	0.0006	1.2	12.27	1	y4 -CO -COCH2 [5|6][1-5][1|2][2-5]	C24H28N6O4S
497.1971	497.1966	0.0006	1.2	12.27	1	y4 -CO [2|3][4-7]	C24H28N6O4S
497.1971	497.1966	0.0006	1.2	12.27	1	b4 -CO [5|6][1-4]	C24H28N6O4S
497.1971	497.1966	0.0006	1.2	12.27	1	b4 -CO -COCH2 [5|6][1-5][2|3][1-4]	C24H28N6O4S
506.1499	506.1493	0.0006	1.2	0.36	1	y4 -H2O -NH3 [4|5][4-7]	C25H23N5O5S
506.1499	506.1493	0.0006	1.2	0.36	1	b4 -H2O -NH3 [7|1][1-4]	C25H23N5O5S
507.1815	507.1809	0.0006	1.2	4.67	1	b4 -H2O [5|6][1-4]	C25H26N6O4S
507.1815	507.1809	0.0006	1.2	4.67	1	y4 -COCH2 -H2O [7|1][1-6][3|4][3-6]	C25H26N6O4S
507.1815	507.1809	0.0006	1.2	4.67	1	y4 -COCH2 -H2O [5|6][1-5][1|2][2-5]	C25H26N6O4S
507.1815	507.1809	0.0006	1.2	4.67	1	y4 -H2O [2|3][4-7]	C25H26N6O4S
507.1815	507.1809	0.0006	1.2	4.67	1	b4 -COCH2 -H2O [5|6][1-5][2|3][1-4]	C25H26N6O4S
507.1815	507.1809	0.0006	1.2	4.67	1	b4 -COCH2 -H2O [7|1][1-6][5|6][1-4]	C25H26N6O4S
508.1655	508.1649	0.0006	1.1	15.97	1	b4 -NH3 [5|6][1-4]	C25H25N5O5S
508.1655	508.1649	0.0006	1.1	15.97	1	y4 -NH3 [2|3][4-7]	C25H25N5O5S
508.1655	508.1649	0.0006	1.1	15.97	1	y4 -COCH2 -NH3 [7|1][1-6][3|4][3-6]	C25H25N5O5S
508.1655	508.1649	0.0006	1.1	15.97	1	y4 -COCH2 -NH3 [5|6][1-5][1|2][2-5]	C25H25N5O5S
508.1655	508.1649	0.0006	1.1	15.97	1	b4 -COCH2 -NH3 [7|1][1-6][5|6][1-4]	C25H25N5O5S
508.1655	508.1649	0.0006	1.1	15.97	1	b4 -COCH2 -NH3 [5|6][1-5][2|3][1-4]	C25H25N5O5S
509.1969	509.1966	0.0003	0.6	0.81	1	b4 -COCH2 -H2O [4|5][1-6][3|4][1-4]	C25H28N6O4S
509.1969	509.1966	0.0003	0.6	0.81	1	y4 -H2O [6|7][1-6][3|4][3-6]	C25H28N6O4S
509.1969	509.1966	0.0003	0.6	0.81	1	y4 -H2O [4|5][1-5][1|2][2-5]	C25H28N6O4S
509.1969	509.1966	0.0003	0.6	0.81	1	y4 -COCH2 -H2O [4|5][1-6][1|2][3-6]	C25H28N6O4S
509.1969	509.1966	0.0003	0.6	0.81	1	b4 -H2O [6|7][1-6][5|6][1-4]	C25H28N6O4S
509.1969	509.1966	0.0003	0.6	0.81	1	b4 -H2O [4|5][1-5][2|3][1-4]	C25H28N6O4S
509.1969	509.1966	0.0003	0.6	0.81	1	y4 -H2O -CO [3|4][4-7]	C25H28N6O4S
509.1969	509.1966	0.0003	0.6	0.81	1	y4 -COCH2 -H2O [7|1][1-5][3|4][2-5]	C25H28N6O4S
509.1969	509.1966	0.0003	0.6	0.81	1	b4 -COCH2 -H2O [7|1][1-5][4|5][1-4]	C25H28N6O4S
509.1969	509.1966	0.0003	0.6	0.81	1	b4 -H2O -CO [6|7][1-4]	C25H28N6O4S
510.1814	510.1806	0.0009	1.7	0.32	1	b4 -NH3 [6|7][1-6][5|6][1-4]	C25H27N5O5S
510.1814	510.1806	0.0009	1.7	0.32	1	y4 -COCH2 -NH3 [4|5][1-6][1|2][3-6]	C25H27N5O5S
510.1814	510.1806	0.0009	1.7	0.32	1	y4 -NH3 -CO [3|4][4-7]	C25H27N5O5S
510.1814	510.1806	0.0009	1.7	0.32	1	y4 -COCH2 -NH3 [7|1][1-5][3|4][2-5]	C25H27N5O5S
510.1814	510.1806	0.0009	1.7	0.32	1	b4 -NH3 [4|5][1-5][2|3][1-4]	C25H27N5O5S
510.1814	510.1806	0.0009	1.7	0.32	1	y4 -NH3 [4|5][1-5][1|2][2-5]	C25H27N5O5S
510.1814	510.1806	0.0009	1.7	0.32	1	b4 -NH3 -CO [6|7][1-4]	C25H27N5O5S
510.1814	510.1806	0.0009	1.7	0.32	1	b4 -COCH2 -NH3 [4|5][1-6][3|4][1-4]	C25H27N5O5S
510.1814	510.1806	0.0009	1.7	0.32	1	y4 -NH3 [6|7][1-6][3|4][3-6]	C25H27N5O5S
510.1814	510.1806	0.0009	1.7	0.32	1	b4 -COCH2 -NH3 [7|1][1-5][4|5][1-4]	C25H27N5O5S
510.2352	510.2347	0.0005	1.0	39.11	1	b5 -NH3 -CO [1|2][1-5]	C26H31N5O6
510.2352	510.2347	0.0005	1.0	39.11	1	y5 -NH3 -CO [6|7][3-7]	C26H31N5O6
510.2352	510.2347	0.0005	1.0	39.11	1	y5 -COCH2 -NH3 [1|2][1-6][2|3][2-6]	C26H31N5O6
510.2352	510.2347	0.0005	1.0	39.11	1	b5 -COCH2 -NH3 [4|5][1-6][4|5][1-5]	C26H31N5O6
510.2352	510.2347	0.0005	1.0	39.11	1	y5 -COCH2 -NH3 [4|5][1-6][3|4][2-6]	C26H31N5O6
510.2352	510.2347	0.0005	1.0	39.11	1	b5 -COCH2 -NH3 [1|2][1-6][3|4][1-5]	C26H31N5O6

Meas. m/z	Calc. m/z	δ (Da)	δ (ppm)	Rel. Int. (%)	z	Annotation	Formula
511.2127	511.2122	0.0005	1.1	85.94	1	b4 -CO [4|5][1-5][3|4][1-4]	C25H30N6O4S
511.2127	511.2122	0.0005	1.1	85.94	1	b4 -CO [7|1][1-6][4|5][1-4]	C25H30N6O4S
511.2127	511.2122	0.0005	1.1	85.94	1	y4 -CO [7|1][1-6][2|3][3-6]	C25H30N6O4S
511.2127	511.2122	0.0005	1.1	85.94	1	y4 -CO [4|5][1-5][2|3][2-5]	C25H30N6O4S
513.1923	513.1915	0.0009	1.7	1.47	1	b4 [3|4][1-6][4|5][1-4]	C24H28N6O5S
513.1923	513.1915	0.0009	1.7	1.47	1	b4 -COCH2 [6|7][1-4]	C24H28N6O5S
513.1923	513.1915	0.0009	1.7	1.47	1	y4 -COCH2 [3|4][4-7]	C24H28N6O5S
513.1923	513.1915	0.0009	1.7	1.47	1	b4 -CO [7|1][1-4]	C24H28N6O5S
513.1923	513.1915	0.0009	1.7	1.47	1	b4 [7|1][1-5][3|4][1-4]	C24H28N6O5S
513.1923	513.1915	0.0009	1.7	1.47	1	y4 [3|4][1-6][2|3][3-6]	C24H28N6O5S
513.1923	513.1915	0.0009	1.7	1.47	1	y4 [7|1][1-5][2|3][2-5]	C24H28N6O5S
513.1923	513.1915	0.0009	1.7	1.47	1	y4 -CO [4|5][4-7]	C24H28N6O5S
518.1821	518.1816	0.0004	0.8	4.02	1	b6 -C10H9NO -H2O [5|6][1-6]	C22H27N7O6S
518.1821	518.1816	0.0004	0.8	4.02	1	y6 -C10H9NO -H2O [4|5][2-7]	C22H27N7O6S
519.1663	519.1656	0.0006	1.2	1.02	1	y6 -C10H9NO -NH3 [4|5][2-7]	C22H26N6O7S
519.1663	519.1656	0.0006	1.2	1.02	1	b6 -C10H9NO -NH3 [5|6][1-6]	C22H26N6O7S
519.2025	519.2020	0.0005	0.9	1.42	1	y6 -NH3 -CO [1|2][2-7]	C23H30N6O6S
519.2025	519.2020	0.0005	0.9	1.42	1	b6 -NH3 -CO [2|3][1-6]	C23H30N6O6S
520.2206	520.2191	0.0016	3.0	0.17	1	b5 -H2O -NH3 [1|2][1-5]	C27H29N5O6
520.2206	520.2191	0.0016	3.0	0.17	1	y5 -H2O -NH3 [6|7][3-7]	C27H29N5O6
523.2129	523.2122	0.0007	1.3	0.78	1	b4 -H2O -CO [4|5][1-6][3|4][1-4]	C26H30N6O4S
523.2129	523.2122	0.0007	1.3	0.78	1	y4 -H2O -CO [4|5][1-6][1|2][3-6]	C26H30N6O4S
523.2129	523.2122	0.0007	1.3	0.78	1	b4 -H2O -CO [7|1][1-5][4|5][1-4]	C26H30N6O4S
523.2129	523.2122	0.0007	1.3	0.78	1	y4 -H2O -CO [7|1][1-5][3|4][2-5]	C26H30N6O4S
524.1603	524.1598	0.0005	1.0	1.01	1	y4 -NH3 [4|5][4-7]	C25H25N5O6S
524.1603	524.1598	0.0005	1.0	1.01	1	b4 -NH3 [7|1][1-4]	C25H25N5O6S
524.1967	524.1962	0.0005	1.0	2.06	1	b4 -NH3 -CO [7|1][1-5][4|5][1-4]	C26H29N5O5S
524.1967	524.1962	0.0005	1.0	2.06	1	b4 -NH3 -CO [4|5][1-6][3|4][1-4]	C26H29N5O5S
524.1967	524.1962	0.0005	1.0	2.06	1	y4 -NH3 -CO [4|5][1-6][1|2][3-6]	C26H29N5O5S
524.1967	524.1962	0.0005	1.0	2.06	1	y4 -NH3 -CO [7|1][1-5][3|4][2-5]	C26H29N5O5S
525.1921	525.1915	0.0006	1.2	4.79	1	y4 -COCH2 [5|6][1-5][1|2][2-5]	C25H28N6O5S
525.1921	525.1915	0.0006	1.2	4.79	1	b4 -COCH2 [5|6][1-5][2|3][1-4]	C25H28N6O5S
525.1921	525.1915	0.0006	1.2	4.79	1	b4 -COCH2 [7|1][1-6][5|6][1-4]	C25H28N6O5S
525.1921	525.1915	0.0006	1.2	4.79	1	y4 -COCH2 [7|1][1-6][3|4][3-6]	C25H28N6O5S
525.1921	525.1915	0.0006	1.2	4.79	1	y4 [2|3][4-7]	C25H28N6O5S
525.1921	525.1915	0.0006	1.2	4.79	1	b4 [5|6][1-4]	C25H28N6O5S
527.2077	527.2071	0.0006	1.1	0.49	1	y4 -COCH2 [4|5][1-6][1|2][3-6]	C25H30N6O5S
527.2077	527.2071	0.0006	1.1	0.49	1	b4 -COCH2 [4|5][1-6][3|4][1-4]	C25H30N6O5S
527.2077	527.2071	0.0006	1.1	0.49	1	b4 -CO [6|7][1-4]	C25H30N6O5S
527.2077	527.2071	0.0006	1.1	0.49	1	b4 [4|5][1-5][2|3][1-4]	C25H30N6O5S
527.2077	527.2071	0.0006	1.1	0.49	1	b4 -COCH2 [7|1][1-5][4|5][1-4]	C25H30N6O5S
527.2077	527.2071	0.0006	1.1	0.49	1	y4 -COCH2 [7|1][1-5][3|4][2-5]	C25H30N6O5S
527.2077	527.2071	0.0006	1.1	0.49	1	y4 [4|5][1-5][1|2][2-5]	C25H30N6O5S
527.2077	527.2071	0.0006	1.1	0.49	1	y4 -CO [3|4][4-7]	C25H30N6O5S
527.2077	527.2071	0.0006	1.1	0.49	1	b4 [6|7][1-6][5|6][1-4]	C25H30N6O5S
527.2077	527.2071	0.0006	1.1	0.49	1	y4 [6|7][1-6][3|4][3-6]	C25H30N6O5S
532.1981	532.1973	0.0008	1.5	0.14	1	b6 -C10H9NO -H2O [7|1][1-6]	C23H29N7O6S
532.1981	532.1973	0.0008	1.5	0.14	1	y6 -C10H9NO -H2O [6|7][2-7]	C23H29N7O6S

Meas. m/z	Calc. m/z	δ (Da)	δ (ppm)	Rel. Int. (%)	z	Annotation	Formula
536.1926	536.1922	0.0004	0.7	2.73	1	b6 -C10H9NO [5|6][1-6]	C22H29N7O7S
536.1926	536.1922	0.0004	0.7	2.73	1	y6 -C10H9NO [4|5][2-7]	C22H29N7O7S
538.2252	538.2231	0.0021	3.8	1.15	1	b5 -H2O -CO [3|4][1-6][3|4][1-5]	C26H31N7O4S
538.2252	538.2231	0.0021	3.8	1.15	1	b5 -H2O -CO [6|7][1-6][4|5][1-5]	C26H31N7O4S
538.2252	538.2231	0.0021	3.8	1.15	1	y5 -H2O -CO [3|4][1-6][2|3][2-6]	C26H31N7O4S
538.2252	538.2231	0.0021	3.8	1.15	1	y5 -H2O -CO [6|7][1-6][3|4][2-6]	C26H31N7O4S
541.1871	541.1864	0.0008	1.4	2.38	1	b4 [7|1][1-4]	C25H28N6O6S
541.1871	541.1864	0.0008	1.4	2.38	1	y4 [4|5][4-7]	C25H28N6O6S
546.2136	546.2129	0.0006	1.2	0.59	1	y6 -H2O [1|2][2-7]	C24H31N7O6S
546.2136	546.2129	0.0006	1.2	0.59	1	b6 -H2O [2|3][1-6]	C24H31N7O6S
546.2136	546.2129	0.0006	1.2	0.59	1	y6 -C10H9NO -H2O [3|4][2-7]	C24H31N7O6S
546.2136	546.2129	0.0006	1.2	0.59	1	b6 -C10H9NO -H2O [4|5][1-6]	C24H31N7O6S
550.2225	550.2231	-0.0006	-1.0	0.14	1	y5 -H2O -CO [3|4][1-6][3|4][2-6]	C27H31N7O4S
550.2225	550.2231	-0.0006	-1.0	0.14	1	y5 -H2O -CO [7|1][1-6][2|3][2-6]	C27H31N7O4S
550.2225	550.2231	-0.0006	-1.0	0.14	1	b5 -H2O -CO [7|1][1-6][3|4][1-5]	C27H31N7O4S
550.2225	550.2231	-0.0006	-1.0	0.14	1	b5 -H2O -CO [3|4][1-6][4|5][1-5]	C27H31N7O4S
553.1874	553.1864	0.0011	1.9	0.34	1	y5 -COCH2 -NH3 [4|5][3-7]	C26H28N6O6S
553.1874	553.1864	0.0011	1.9	0.34	1	b5 -COCH2 -NH3 [6|7][1-5]	C26H28N6O6S
554.2198	554.2180	0.0018	3.2	0.16	1	b5 -CO -COCH2 [7|1][1-6][5|6][1-5]	C26H31N7O5S
554.2198	554.2180	0.0018	3.2	0.16	1	y5 -CO -COCH2 [7|1][1-6][4|5][2-6]	C26H31N7O5S
554.2198	554.2180	0.0018	3.2	0.16	1	y5 -CO [3|4][1-6][1|2][2-6]	C26H31N7O5S
554.2198	554.2180	0.0018	3.2	0.16	1	b5 -CO [3|4][1-6][2|3][1-5]	C26H31N7O5S
554.2198	554.2180	0.0018	3.2	0.16	1	y5 -CO -COCH2 [5|6][1-6][1|2][2-6]	C26H31N7O5S
554.2198	554.2180	0.0018	3.2	0.16	1	b5 -CO -COCH2 [5|6][1-6][2|3][1-5]	C26H31N7O5S
554.2198	554.2180	0.0018	3.2	0.16	1	y5 -CO [5|6][1-6][4|5][2-6]	C26H31N7O5S
554.2198	554.2180	0.0018	3.2	0.16	1	b5 -CO [5|6][1-6][5|6][1-5]	C26H31N7O5S
555.2026	555.2020	0.0005	1.0	17.30	1	y4 [3|4][4-7]	C26H30N6O6S
555.2026	555.2020	0.0005	1.0	17.30	1	b4 [6|7][1-4]	C26H30N6O6S
565.2234	565.2228	0.0007	1.2	7.60	1	b5 -NH3 -CO [4|5][1-5]	C28H32N6O5S
565.2234	565.2228	0.0007	1.2	7.60	1	y5 -NH3 -CO [2|3][3-7]	C28H32N6O5S
566.2186	566.2180	0.0006	1.0	1.68	1	y5 -COCH2 -H2O [5|6][3-7]	C27H31N7O5S
566.2186	566.2180	0.0006	1.0	1.68	1	b5 -COCH2 -H2O [7|1][1-5]	C27H31N7O5S
566.2186	566.2180	0.0006	1.0	1.68	1	y5 -H2O -CO [4|5][3-7]	C27H31N7O5S
566.2186	566.2180	0.0006	1.0	1.68	1	b5 -H2O -CO [6|7][1-5]	C27H31N7O5S
566.2186	566.2180	0.0006	1.0	1.68	1	y5 -H2O [3|4][1-6][2|3][2-6]	C27H31N7O5S
566.2186	566.2180	0.0006	1.0	1.68	1	b5 -H2O [3|4][1-6][3|4][1-5]	C27H31N7O5S
566.2186	566.2180	0.0006	1.0	1.68	1	y5 -H2O [6|7][1-6][3|4][2-6]	C27H31N7O5S
566.2186	566.2180	0.0006	1.0	1.68	1	b5 -H2O [6|7][1-6][4|5][1-5]	C27H31N7O5S
567.2023	567.2020	0.0003	0.5	0.25	1	y4 [5|6][1-5][1|2][2-5]	C27H30N6O6S
567.2023	567.2020	0.0003	0.5	0.25	1	y4 [7|1][1-6][3|4][3-6]	C27H30N6O6S
567.2023	567.2020	0.0003	0.5	0.25	1	b4 [7|1][1-6][5|6][1-4]	C27H30N6O6S
567.2023	567.2020	0.0003	0.5	0.25	1	b4 [5|6][1-5][2|3][1-4]	C27H30N6O6S
567.2023	567.2020	0.0003	0.5	0.25	1	b5 -COCH2 -NH3 [7|1][1-5]	C27H30N6O6S
567.2023	567.2020	0.0003	0.5	0.25	1	y5 -NH3 -CO [4|5][3-7]	C27H30N6O6S
567.2023	567.2020	0.0003	0.5	0.25	1	b5 -NH3 [3|4][1-6][3|4][1-5]	C27H30N6O6S
567.2023	567.2020	0.0003	0.5	0.25	1	y5 -COCH2 -NH3 [5|6][3-7]	C27H30N6O6S
567.2023	567.2020	0.0003	0.5	0.25	1	b5 -NH3 -CO [6|7][1-5]	C27H30N6O6S
567.2023	567.2020	0.0003	0.5	0.25	1	y5 -NH3 [3|4][1-6][2|3][2-6]	C27H30N6O6S

Meas. m/z	Calc. m/z	δ (Da)	δ (ppm)	Rel. Int. (%)	z	Annotation	Formula
567.2023	567.2020	0.0003	0.5	0.25	1	y5 -NH3 [6|7][1-6][3|4][2-6]	C27H30N6O6S
567.2023	567.2020	0.0003	0.5	0.25	1	b5 -NH3 [6|7][1-6][4|5][1-5]	C27H30N6O6S
568.2343	568.2337	0.0006	1.1	2.00	1	y5 -CO -COCH2 [3|4][3-7]	C27H33N7O5S
568.2343	568.2337	0.0006	1.1	2.00	1	b5 -CO -COCH2 [5|6][1-5]	C27H33N7O5S
568.2343	568.2337	0.0006	1.1	2.00	1	b5 -CO [3|4][1-6][4|5][1-5]	C27H33N7O5S
568.2343	568.2337	0.0006	1.1	2.00	1	y5 -CO [7|1][1-6][2|3][2-6]	C27H33N7O5S
568.2343	568.2337	0.0006	1.1	2.00	1	b5 -CO [7|1][1-6][3|4][1-5]	C27H33N7O5S
568.2343	568.2337	0.0006	1.1	2.00	1	y5 -CO [3|4][1-6][3|4][2-6]	C27H33N7O5S
578.2199	578.2180	0.0019	3.3	0.29	1	y5 -H2O [7|1][1-6][2|3][2-6]	C28H31N7O5S
578.2199	578.2180	0.0019	3.3	0.29	1	y5 -H2O [3|4][1-6][3|4][2-6]	C28H31N7O5S
578.2199	578.2180	0.0019	3.3	0.29	1	b5 -H2O [7|1][1-6][3|4][1-5]	C28H31N7O5S
578.2199	578.2180	0.0019	3.3	0.29	1	y5 -H2O -CO [7|1][1-6][4|5][2-6]	C28H31N7O5S
578.2199	578.2180	0.0019	3.3	0.29	1	b5 -H2O -CO [7|1][1-6][5|6][1-5]	C28H31N7O5S
578.2199	578.2180	0.0019	3.3	0.29	1	b5 -H2O [3|4][1-6][4|5][1-5]	C28H31N7O5S
578.2199	578.2180	0.0019	3.3	0.29	1	y5 -H2O -CO [5|6][1-6][1|2][2-6]	C28H31N7O5S
578.2199	578.2180	0.0019	3.3	0.29	1	b5 -H2O -CO [5|6][1-6][2|3][1-5]	C28H31N7O5S
578.2199	578.2180	0.0019	3.3	0.29	1	y5 -COCH2 -H2O [3|4][3-7]	C28H31N7O5S
578.2199	578.2180	0.0019	3.3	0.29	1	b5 -COCH2 -H2O [5|6][1-5]	C28H31N7O5S
580.2346	580.2337	0.0010	1.6	0.79	1	b5 -H2O -CO [7|1][1-5]	C28H33N7O5S
580.2346	580.2337	0.0010	1.6	0.79	1	y5 -H2O -CO [5|6][3-7]	C28H33N7O5S
580.2346	580.2337	0.0010	1.6	0.79	1	y5 -COCH2 -H2O [4|5][1-6][1|2][2-6]	C28H33N7O5S
580.2346	580.2337	0.0010	1.6	0.79	1	b5 -COCH2 -H2O [4|5][1-6][2|3][1-5]	C28H33N7O5S
580.2346	580.2337	0.0010	1.6	0.79	1	y5 -COCH2 -H2O [6|7][1-6][4|5][2-6]	C28H33N7O5S
580.2346	580.2337	0.0010	1.6	0.79	1	b5 -COCH2 -H2O [6|7][1-6][5|6][1-5]	C28H33N7O5S
581.2186	581.2177	0.0009	1.5	1.43	1	y5 -COCH2 -NH3 [4|5][1-6][1|2][2-6]	C28H32N6O6S
581.2186	581.2177	0.0009	1.5	1.43	1	b5 -NH3 -CO [7|1][1-5]	C28H32N6O6S
581.2186	581.2177	0.0009	1.5	1.43	1	y5 -NH3 -CO [5|6][3-7]	C28H32N6O6S
581.2186	581.2177	0.0009	1.5	1.43	1	b5 -COCH2 -NH3 [4|5][1-6][2|3][1-5]	C28H32N6O6S
581.2186	581.2177	0.0009	1.5	1.43	1	y5 -COCH2 -NH3 [6|7][1-6][4|5][2-6]	C28H32N6O6S
581.2186	581.2177	0.0009	1.5	1.43	1	b5 -COCH2 -NH3 [6|7][1-6][5|6][1-5]	C28H32N6O6S
581.2725	581.2718	0.0006	1.1	3.87	1	y6 -NH3 -CO [7|1][2-7]	C29H36N6O7
581.2725	581.2718	0.0006	1.1	3.87	1	b6 -NH3 -CO [1|2][1-6]	C29H36N6O7
582.2503	582.2493	0.0010	1.7	0.94	1	b5 -CO -COCH2 [4|5][1-6][3|4][1-5]	C28H35N7O5S
582.2503	582.2493	0.0010	1.7	0.94	1	b5 -CO -COCH2 [7|1][1-6][4|5][1-5]	C28H35N7O5S
582.2503	582.2493	0.0010	1.7	0.94	1	y5 -CO -COCH2 [4|5][1-6][2|3][2-6]	C28H35N7O5S
582.2503	582.2493	0.0010	1.7	0.94	1	y5 -CO -COCH2 [7|1][1-6][3|4][2-6]	C28H35N7O5S
582.2503	582.2493	0.0010	1.7	0.94	1	b5 -CO [4|5][1-5]	C28H35N7O5S
582.2503	582.2493	0.0010	1.7	0.94	1	y5 -CO [2|3][3-7]	C28H35N7O5S
584.2292	584.2286	0.0007	1.1	9.45	1	b5 -CO [6|7][1-5]	C27H33N7O6S
584.2292	584.2286	0.0007	1.1	9.45	1	y5 -COCH2 [5|6][3-7]	C27H33N7O6S
584.2292	584.2286	0.0007	1.1	9.45	1	y5 [3|4][1-6][2|3][2-6]	C27H33N7O6S
584.2292	584.2286	0.0007	1.1	9.45	1	y5 -CO [4|5][3-7]	C27H33N7O6S
584.2292	584.2286	0.0007	1.1	9.45	1	b5 -COCH2 [7|1][1-5]	C27H33N7O6S
584.2292	584.2286	0.0007	1.1	9.45	1	y5 [6|7][1-6][3|4][2-6]	C27H33N7O6S
584.2292	584.2286	0.0007	1.1	9.45	1	b5 [6|7][1-6][4|5][1-5]	C27H33N7O6S
584.2292	584.2286	0.0007	1.1	9.45	1	b5 [3|4][1-6][3|4][1-5]	C27H33N7O6S
592.2352	592.2337	0.0015	2.5	0.30	1	y5 -COCH2 -H2O [7|1][1-6][3|4][2-6]	C29H33N7O5S
592.2352	592.2337	0.0015	2.5	0.30	1	y5 -H2O [2|3][3-7]	C29H33N7O5S

Meas. m/z	Calc. m/z	δ (Da)	δ (ppm)	Rel. Int. (%)	z	Annotation	Formula
592.2352	592.2337	0.0015	2.5	0.30	1	b5 -H2O [4|5][1-5]	C29H33N7O5S
592.2352	592.2337	0.0015	2.5	0.30	1	y5 -H2O -CO [3|4][3-7]	C29H33N7O5S
592.2352	592.2337	0.0015	2.5	0.30	1	b5 -H2O -CO [5|6][1-5]	C29H33N7O5S
592.2352	592.2337	0.0015	2.5	0.30	1	b5 -COCH2 -H2O [7|1][1-6][4|5][1-5]	C29H33N7O5S
592.2352	592.2337	0.0015	2.5	0.30	1	b5 -COCH2 -H2O [4|5][1-6][3|4][1-5]	C29H33N7O5S
592.2352	592.2337	0.0015	2.5	0.30	1	y5 -COCH2 -H2O [4|5][1-6][2|3][2-6]	C29H33N7O5S
593.2184	593.2177	0.0008	1.3	0.28	1	b5 -NH3 -CO [5|6][1-5]	C29H32N6O6S
593.2184	593.2177	0.0008	1.3	0.28	1	b5 -COCH2 -NH3 [7|1][1-6][4|5][1-5]	C29H32N6O6S
593.2184	593.2177	0.0008	1.3	0.28	1	y5 -NH3 -CO [3|4][3-7]	C29H32N6O6S
593.2184	593.2177	0.0008	1.3	0.28	1	y5 -NH3 [2|3][3-7]	C29H32N6O6S
593.2184	593.2177	0.0008	1.3	0.28	1	y5 -COCH2 -NH3 [4|5][1-6][2|3][2-6]	C29H32N6O6S
593.2184	593.2177	0.0008	1.3	0.28	1	y5 -COCH2 -NH3 [7|1][1-6][3|4][2-6]	C29H32N6O6S
593.2184	593.2177	0.0008	1.3	0.28	1	b5 -NH3 [4|5][1-5]	C29H32N6O6S
593.2184	593.2177	0.0008	1.3	0.28	1	b5 -COCH2 -NH3 [4|5][1-6][3|4][1-5]	C29H32N6O6S
593.2505	593.2500	0.0004	0.8	0.51	1	M -C10H9NO -CO [7|1][1-7]	C25H36N8O7S
594.2139	594.2129	0.0010	1.7	3.35	1	y5 -H2O [4|5][3-7]	C28H31N7O6S
594.2139	594.2129	0.0010	1.7	3.35	1	b5 -H2O [6|7][1-5]	C28H31N7O6S
594.2501	594.2493	0.0008	1.4	21.42	1	y5 -H2O -CO [4|5][1-6][1|2][2-6]	C29H35N7O5S
594.2501	594.2493	0.0008	1.4	21.42	1	b5 -H2O -CO [4|5][1-6][2|3][1-5]	C29H35N7O5S
594.2501	594.2493	0.0008	1.4	21.42	1	y5 -H2O -CO [6|7][1-6][4|5][2-6]	C29H35N7O5S
594.2501	594.2493	0.0008	1.4	21.42	1	b5 -H2O -CO [6|7][1-6][5|6][1-5]	C29H35N7O5S
595.2338	595.2333	0.0005	0.8	0.61	1	y5 -NH3 -CO [4|5][1-6][1|2][2-6]	C29H34N6O6S
595.2338	595.2333	0.0005	0.8	0.61	1	b5 -NH3 -CO [4|5][1-6][2|3][1-5]	C29H34N6O6S
595.2338	595.2333	0.0005	0.8	0.61	1	y5 -NH3 -CO [6|7][1-6][4|5][2-6]	C29H34N6O6S
595.2338	595.2333	0.0005	0.8	0.61	1	b5 -NH3 -CO [6|7][1-6][5|6][1-5]	C29H34N6O6S
596.2294	596.2286	0.0008	1.3	0.32	1	y5 [7|1][1-6][2|3][2-6]	C28H33N7O6S
596.2294	596.2286	0.0008	1.3	0.32	1	b5 [7|1][1-6][3|4][1-5]	C28H33N7O6S
596.2294	596.2286	0.0008	1.3	0.32	1	y5 [3|4][1-6][3|4][2-6]	C28H33N7O6S
596.2294	596.2286	0.0008	1.3	0.32	1	b5 [3|4][1-6][4|5][1-5]	C28H33N7O6S
596.2294	596.2286	0.0008	1.3	0.32	1	y5 -COCH2 [3|4][3-7]	C28H33N7O6S
596.2294	596.2286	0.0008	1.3	0.32	1	b5 -COCH2 [5|6][1-5]	C28H33N7O6S
596.2294	596.2286	0.0008	1.3	0.32	1	y5 -CO [7|1][1-6][4|5][2-6]	C28H33N7O6S
596.2294	596.2286	0.0008	1.3	0.32	1	b5 -CO [7|1][1-6][5|6][1-5]	C28H33N7O6S
596.2294	596.2286	0.0008	1.3	0.32	1	y5 -CO [5|6][1-6][1|2][2-6]	C28H33N7O6S
596.2294	596.2286	0.0008	1.3	0.32	1	b5 -CO [5|6][1-6][2|3][1-5]	C28H33N7O6S
598.2449	598.2442	0.0007	1.2	6.72	1	b5 -CO [7|1][1-5]	C28H35N7O6S
598.2449	598.2442	0.0007	1.2	6.72	1	y5 -CO [5|6][3-7]	C28H35N7O6S
598.2449	598.2442	0.0007	1.2	6.72	1	y5 -COCH2 [4|5][1-6][1|2][2-6]	C28H35N7O6S
598.2449	598.2442	0.0007	1.2	6.72	1	b5 -COCH2 [4|5][1-6][2|3][1-5]	C28H35N7O6S
598.2449	598.2442	0.0007	1.2	6.72	1	y5 -COCH2 [6|7][1-6][4|5][2-6]	C28H35N7O6S
598.2449	598.2442	0.0007	1.2	6.72	1	b5 -COCH2 [6|7][1-6][5|6][1-5]	C28H35N7O6S
603.2352	603.2344	0.0008	1.4	74.03	1	M -C10H9NO -H2O [7|1][1-7]	C26H34N8O7S
604.2192	604.2184	0.0008	1.4	0.17	1	M -C10H9NO -NH3 [7|1][1-7]	C26H33N7O8S
607.2341	607.2333	0.0007	1.2	0.34	1	y5 -NH3 -CO [7|1][1-6][3|4][2-6]	C30H34N6O6S
607.2341	607.2333	0.0007	1.2	0.34	1	b5 -NH3 -CO [7|1][1-6][4|5][1-5]	C30H34N6O6S
607.2341	607.2333	0.0007	1.2	0.34	1	y5 -NH3 -CO [4|5][1-6][2|3][2-6]	C30H34N6O6S
607.2341	607.2333	0.0007	1.2	0.34	1	b5 -NH3 -CO [4|5][1-6][3|4][1-5]	C30H34N6O6S
608.2296	608.2286	0.0010	1.7	0.87	1	b5 -H2O [7|1][1-5]	C29H33N7O6S

Meas. m/z	Calc. m/z	δ (Da)	δ (ppm)	Rel. Int. (%)	z	Annotation	Formula
608.2296	608.2286	0.0010	1.7	0.87	1	y5 -H2O [5|6][3-7]	C29H33N7O6S
609.2132	609.2126	0.0006	1.0	1.02	1	b5 -NH3 [7|1][1-5]	C29H32N6O7S
609.2132	609.2126	0.0006	1.0	1.02	1	y5 -NH3 [5|6][3-7]	C29H32N6O7S
610.2448	610.2442	0.0006	1.0	1.36	1	y5 [2|3][3-7]	C29H35N7O6S
610.2448	610.2442	0.0006	1.0	1.36	1	b5 [4|5][1-5]	C29H35N7O6S
610.2448	610.2442	0.0006	1.0	1.36	1	y5 -CO [3|4][3-7]	C29H35N7O6S
610.2448	610.2442	0.0006	1.0	1.36	1	b5 -CO [5|6][1-5]	C29H35N7O6S
610.2448	610.2442	0.0006	1.0	1.36	1	y5 -COCH2 [7|1][1-6][3|4][2-6]	C29H35N7O6S
610.2448	610.2442	0.0006	1.0	1.36	1	b5 -COCH2 [7|1][1-6][4|5][1-5]	C29H35N7O6S
610.2448	610.2442	0.0006	1.0	1.36	1	y5 -COCH2 [4|5][1-6][2|3][2-6]	C29H35N7O6S
610.2448	610.2442	0.0006	1.0	1.36	1	b5 -COCH2 [4|5][1-6][3|4][1-5]	C29H35N7O6S
612.2243	612.2235	0.0008	1.3	8.68	1	y5 [4|5][3-7]	C28H33N7O7S
612.2243	612.2235	0.0008	1.3	8.68	1	b5 [6|7][1-5]	C28H33N7O7S
620.2291	620.2286	0.0005	0.9	4.27	1	y5 -H2O [3|4][3-7]	C30H33N7O6S
620.2291	620.2286	0.0005	0.9	4.27	1	b5 -H2O [5|6][1-5]	C30H33N7O6S
621.2134	621.2126	0.0008	1.3	28.79	1	y5 -NH3 [3|4][3-7]	C30H32N6O7S
621.2134	621.2126	0.0008	1.3	28.79	1	b5 -NH3 [5|6][1-5]	C30H32N6O7S
621.2458	621.2450	0.0009	1.4	7.30	1	M -C10H9NO [7|1][1-7]	C26H36N8O8S
623.2293	623.2282	0.0011	1.7	0.15	1	y5 -NH3 [4|5][1-6][1|2][2-6]	C30H34N6O7S
623.2293	623.2282	0.0011	1.7	0.15	1	b5 -NH3 [4|5][1-6][2|3][1-5]	C30H34N6O7S
623.2293	623.2282	0.0011	1.7	0.15	1	y5 -NH3 [6|7][1-6][4|5][2-6]	C30H34N6O7S
623.2293	623.2282	0.0011	1.7	0.15	1	b5 -NH3 [6|7][1-6][5|6][1-5]	C30H34N6O7S
624.2244	624.2235	0.0009	1.5	0.91	1	y5 [7|1][1-6][4|5][2-6]	C29H33N7O7S
624.2244	624.2235	0.0009	1.5	0.91	1	b5 [7|1][1-6][5|6][1-5]	C29H33N7O7S
624.2244	624.2235	0.0009	1.5	0.91	1	y5 [5|6][1-6][1|2][2-6]	C29H33N7O7S
624.2244	624.2235	0.0009	1.5	0.91	1	b5 [5|6][1-6][2|3][1-5]	C29H33N7O7S
626.2401	626.2391	0.0009	1.4	1.60	1	b5 [7|1][1-5]	C29H35N7O7S
626.2401	626.2391	0.0009	1.4	1.60	1	y5 [5|6][3-7]	C29H35N7O7S
635.2404	635.2395	0.0009	1.5	0.28	1	y6 -COCH2 -H2O [4|5][2-7]	C30H34N8O6S
635.2404	635.2395	0.0009	1.5	0.28	1	b6 -COCH2 -H2O [5|6][1-6]	C30H34N8O6S
636.2239	636.2235	0.0004	0.7	1.16	1	y6 -COCH2 -NH3 [4|5][2-7]	C30H33N7O7S
636.2239	636.2235	0.0004	0.7	1.16	1	b6 -COCH2 -NH3 [5|6][1-6]	C30H33N7O7S
638.2401	638.2391	0.0009	1.4	10.71	1	y5 [3|4][3-7]	C30H35N7O7S
638.2401	638.2391	0.0009	1.4	10.71	1	b5 [5|6][1-5]	C30H35N7O7S
638.2401	638.2391	0.0009	1.4	10.71	1	y6 -COCH2 -NH3 [5|6][2-7]	C30H35N7O7S
638.2401	638.2391	0.0009	1.4	10.71	1	b6 -COCH2 -NH3 [6|7][1-6]	C30H35N7O7S
639.2718	639.2708	0.0010	1.6	0.43	1	b6 -CO -COCH2 [7|1][1-6]	C30H38N8O6S
639.2718	639.2708	0.0010	1.6	0.43	1	y6 -CO [2|3][2-7]	C30H38N8O6S
639.2718	639.2708	0.0010	1.6	0.43	1	b6 -CO [3|4][1-6]	C30H38N8O6S
639.2718	639.2708	0.0010	1.6	0.43	1	y6 -CO -COCH2 [6|7][2-7]	C30H38N8O6S
649.2563	649.2551	0.0012	1.9	1.31	1	y6 -H2O [2|3][2-7]	C31H36N8O6S
649.2563	649.2551	0.0012	1.9	1.31	1	b6 -H2O [3|4][1-6]	C31H36N8O6S
649.2563	649.2551	0.0012	1.9	1.31	1	y6 -H2O -CO [4|5][2-7]	C31H36N8O6S
649.2563	649.2551	0.0012	1.9	1.31	1	b6 -H2O -CO [5|6][1-6]	C31H36N8O6S
649.2563	649.2551	0.0012	1.9	1.31	1	y6 -COCH2 -H2O [6|7][2-7]	C31H36N8O6S
649.2563	649.2551	0.0012	1.9	1.31	1	b6 -COCH2 -H2O [7|1][1-6]	C31H36N8O6S
650.2399	650.2391	0.0008	1.2	0.65	1	b6 -COCH2 -NH3 [7|1][1-6]	C31H35N7O7S
650.2399	650.2391	0.0008	1.2	0.65	1	y6 -NH3 [2|3][2-7]	C31H35N7O7S

Meas. m/z	Calc. m/z	δ (Da)	δ (ppm)	Rel. Int. (%)	z	Annotation	Formula
650.2399	650.2391	0.0008	1.2	0.65	1	b6 -NH3 [3|4][1-6]	C31H35N7O7S
650.2399	650.2391	0.0008	1.2	0.65	1	y6 -NH3 -CO [4|5][2-7]	C31H35N7O7S
650.2399	650.2391	0.0008	1.2	0.65	1	b6 -NH3 -CO [5|6][1-6]	C31H35N7O7S
650.2399	650.2391	0.0008	1.2	0.65	1	y6 -COCH2 -NH3 [6|7][2-7]	C31H35N7O7S
651.2717	651.2708	0.0010	1.5	0.99	1	y6 -H2O -CO [5|6][2-7]	C31H38N8O6S
651.2717	651.2708	0.0010	1.5	0.99	1	b6 -H2O -CO [6|7][1-6]	C31H38N8O6S
652.2559	652.2548	0.0011	1.7	0.25	1	y5 [7|1][1-6][3|4][2-6]	C31H37N7O7S
652.2559	652.2548	0.0011	1.7	0.25	1	b5 [7|1][1-6][4|5][1-5]	C31H37N7O7S
652.2559	652.2548	0.0011	1.7	0.25	1	y5 [4|5][1-6][2|3][2-6]	C31H37N7O7S
652.2559	652.2548	0.0011	1.7	0.25	1	b5 [4|5][1-6][3|4][1-5]	C31H37N7O7S
652.2559	652.2548	0.0011	1.7	0.25	1	y6 -NH3 -CO [5|6][2-7]	C31H37N7O7S
652.2559	652.2548	0.0011	1.7	0.25	1	b6 -NH3 -CO [6|7][1-6]	C31H37N7O7S
663.2714	663.2708	0.0006	1.0	0.38	1	b6 -H2O -CO [7|1][1-6]	C32H38N8O6S
663.2714	663.2708	0.0006	1.0	0.38	1	y6 -COCH2 -H2O [3|4][2-7]	C32H38N8O6S
663.2714	663.2708	0.0006	1.0	0.38	1	b6 -COCH2 -H2O [4|5][1-6]	C32H38N8O6S
663.2714	663.2708	0.0006	1.0	0.38	1	y6 -H2O -CO [6|7][2-7]	C32H38N8O6S
667.2666	667.2657	0.0009	1.4	5.34	1	y6 [2|3][2-7]	C31H38N8O7S
667.2666	667.2657	0.0009	1.4	5.34	1	b6 [3|4][1-6]	C31H38N8O7S
667.2666	667.2657	0.0009	1.4	5.34	1	b6 -COCH2 [7|1][1-6]	C31H38N8O7S
667.2666	667.2657	0.0009	1.4	5.34	1	y6 -CO [4|5][2-7]	C31H38N8O7S
667.2666	667.2657	0.0009	1.4	5.34	1	b6 -CO [5|6][1-6]	C31H38N8O7S
667.2666	667.2657	0.0009	1.4	5.34	1	y6 -COCH2 [6|7][2-7]	C31H38N8O7S
669.2823	669.2813	0.0010	1.5	19.53	1	y6 -CO [5|6][2-7]	C31H40N8O7S
669.2823	669.2813	0.0010	1.5	19.53	1	b6 -CO [6|7][1-6]	C31H40N8O7S
677.2510	677.2500	0.0009	1.4	22.61	1	y6 -H2O [4|5][2-7]	C32H36N8O7S
677.2510	677.2500	0.0009	1.4	22.61	1	b6 -H2O [5|6][1-6]	C32H36N8O7S
678.2350	678.2341	0.0010	1.5	29.51	1	y6 -NH3 [4|5][2-7]	C32H35N7O8S
678.2350	678.2341	0.0010	1.5	29.51	1	b6 -NH3 [5|6][1-6]	C32H35N7O8S
678.2712	678.2704	0.0007	1.1	24.25	1	y6 -NH3 -CO [3|4][2-7]	C33H39N7O7S
678.2712	678.2704	0.0007	1.1	24.25	1	b6 -NH3 -CO [4|5][1-6]	C33H39N7O7S
679.2664	679.2657	0.0007	1.1	1.73	1	y6 -H2O [5|6][2-7]	C32H38N8O7S
679.2664	679.2657	0.0007	1.1	1.73	1	b6 -H2O [6|7][1-6]	C32H38N8O7S
681.2823	681.2813	0.0009	1.4	1.89	1	b6 -CO [7|1][1-6]	C32H40N8O7S
681.2823	681.2813	0.0009	1.4	1.89	1	y6 -COCH2 [3|4][2-7]	C32H40N8O7S
681.2823	681.2813	0.0009	1.4	1.89	1	b6 -COCH2 [4|5][1-6]	C32H40N8O7S
681.2823	681.2813	0.0009	1.4	1.89	1	y6 -CO [6|7][2-7]	C32H40N8O7S
691.2669	691.2657	0.0012	1.8	1.38	1	b6 -H2O [7|1][1-6]	C33H38N8O7S
691.2669	691.2657	0.0012	1.8	1.38	1	y6 -H2O [6|7][2-7]	C33H38N8O7S
695.2615	695.2606	0.0009	1.3	100.00	1	y6 [4|5][2-7]	C32H38N8O8S
695.2615	695.2606	0.0009	1.3	100.00	1	b6 [5|6][1-6]	C32H38N8O8S
697.2774	697.2763	0.0011	1.6	2.38	1	y6 [5|6][2-7]	C32H40N8O8S
697.2774	697.2763	0.0011	1.6	2.38	1	b6 [6|7][1-6]	C32H40N8O8S
705.2815	705.2813	0.0002	0.2	0.24	1	b6 -H2O [4|5][1-6]	C34H40N8O7S
705.2815	705.2813	0.0002	0.2	0.24	1	y6 -H2O [3|4][2-7]	C34H40N8O7S
709.2772	709.2763	0.0010	1.4	4.31	1	b6 [7|1][1-6]	C33H40N8O8S
709.2772	709.2763	0.0010	1.4	4.31	1	y6 [6|7][2-7]	C33H40N8O8S
720.2933	720.2922	0.0011	1.5	3.28	1	M -COCH2 -H2O [7|1][1-7]	C34H41N9O7S
734.3090	734.3079	0.0011	1.5	52.13	1	M -H2O -CO [7|1][1-7]	C35H43N9O7S

Meas. m/z	Calc. m/z	δ (Da)	δ (ppm)	Rel. Int. (%)	z	Annotation	Formula
735.2930	735.2919	0.0011	1.4	20.51	1	M -NH3 -CO [7|1][1-7]	C35H42N8O8S
745.2768	745.2763	0.0005	0.7	0.63	1	M -H2O -NH3 [7|1][1-7]	C36H40N8O8S
752.3194	752.3185	0.0009	1.2	93.81	1	M -CO [7|1][1-7]	C35H45N9O8S
762.3037	762.3028	0.0009	1.2	85.56	1	M -H2O [7|1][1-7]	C36H43N9O8S
763.2879	763.2868	0.0011	1.4	3.38	1	M -NH3 [7|1][1-7]	C36H42N8O9S

Generated by mMass • Open Source Mass Spectrometry Tool • www.mmass.org

mMass Report: *MSn spectrum [568]*

**Date**	Fri Jul 19 06:14:04 2019	**Scan Number**	568
**Operator**		**Retention Time**	502.2816
**Contact**		**MS Level**	2
**Institution**		**Precursor m/z**	780.31
**Instrument**		**Polarity**	positive
		**Spectrum Points**	0
		**Peak List**	373

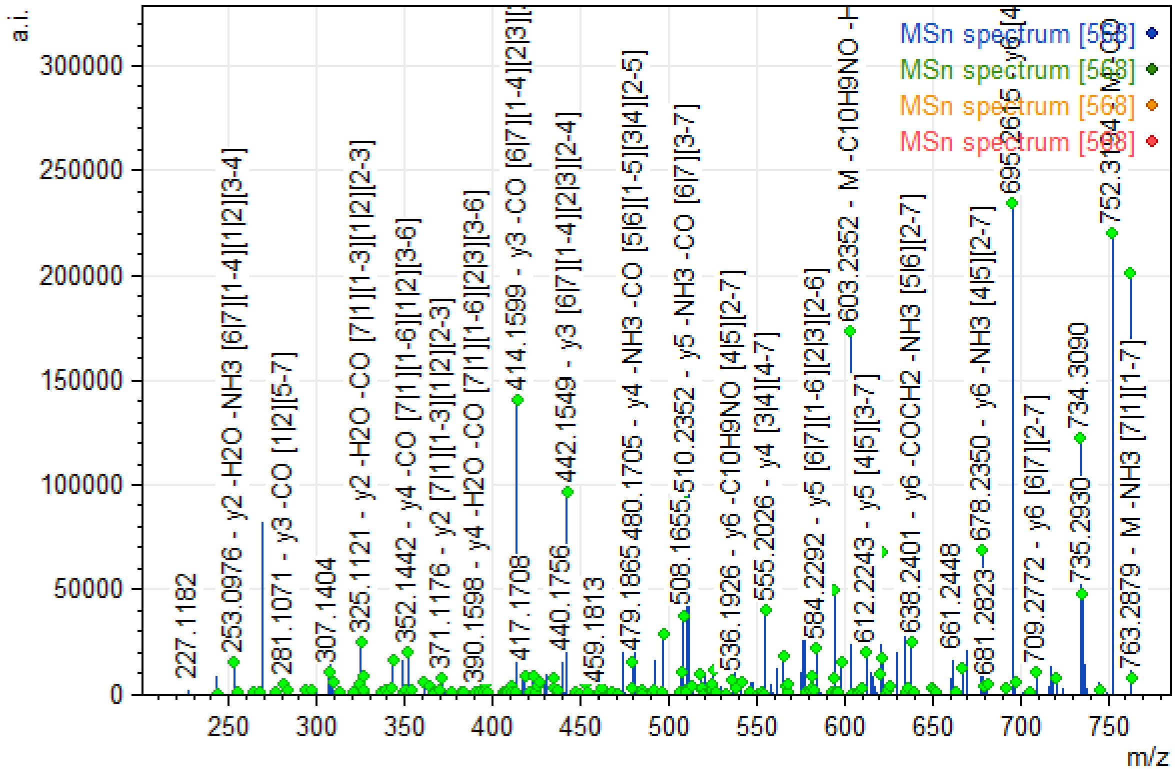

Sequence - *Aryrin-like scrambling*

Accession	Length	Mo. Mass	Av. Mass	Coverage	Matched Int.
	7 (Cyclic)	779.3061	779.8652	100.0 %	71.1 %

Ala-Thz | 2Me-dhThr | (OMe)Trp | Gly | Abu | Dhb | Sar

Meas. m/z	Calc. m/z	δ (Da)	δ (ppm)	Rel. Int. (%)	z	Annotation	Formula
243.1131	243.1128	0.0003	1.2	0.16	1	y2 -NH3 -CO [2|3][1-5][1|2][4-5]	C14H14N2O2
243.1131	243.1128	0.0003	1.2	0.16	1	b2 -NH3 -CO [2|3][1-5][4|5][1-2]	C14H14N2O2
243.1131	243.1128	0.0003	1.2	0.16	1	b2 -NH3 -CO [6|7][1-4][3|4][1-2]	C14H14N2O2
243.1131	243.1128	0.0003	1.2	0.16	1	y2 -NH3 -CO [6|7][1-4][1|2][3-4]	C14H14N2O2
253.0976	253.0972	0.0004	1.6	6.54	1	b2 -H2O -NH3 [2|3][1-5][4|5][1-2]	C15H12N2O2
253.0976	253.0972	0.0004	1.6	6.54	1	b2 -H2O -NH3 [6|7][1-4][3|4][1-2]	C15H12N2O2
253.0976	253.0972	0.0004	1.6	6.54	1	y2 -H2O -NH3 [2|3][1-5][1|2][4-5]	C15H12N2O2
253.0976	253.0972	0.0004	1.6	6.54	1	y2 -H2O -NH3 [6|7][1-4][1|2][3-4]	C15H12N2O2
255.1133	255.1128	0.0005	1.9	0.38	1	b2 -NH3 -CO [2|3][1-4][3|4][1-2]	C15H14N2O2
255.1133	255.1128	0.0005	1.9	0.38	1	y2 -NH3 -CO [2|3][1-4][1|2][3-4]	C15H14N2O2
255.1133	255.1128	0.0005	1.9	0.38	1	b2 -NH3 -CO [5|6][1-5][4|5][1-2]	C15H14N2O2

Meas. m/z	Calc. m/z	δ (Da)	δ (ppm)	Rel. Int. (%)	z	Annotation	Formula
255.1133	255.1128	0.0005	1.9	0.38	1	y2 -NH3 -CO [5|6][1-5][1|2][4-5]	C15H14N2O2
264.0803	264.0801	0.0001	0.5	0.57	1	y3 -NH3 -CO [1|2][5-7]	C12H13N3O2S
264.0803	264.0801	0.0001	0.5	0.57	1	b3 -NH3 -CO [5|6][1-3]	C12H13N3O2S
267.1134	267.1128	0.0006	2.2	0.54	1	y2 -H2O -NH3 [2|3][1-3][1|2][2-3]	C16H14N2O2
267.1134	267.1128	0.0006	2.2	0.54	1	y2 -H2O -NH3 [4|5][1-6][1|2][5-6]	C16H14N2O2
267.1134	267.1128	0.0006	2.2	0.54	1	b2 -H2O -NH3 [4|5][1-6][5|6][1-2]	C16H14N2O2
267.1134	267.1128	0.0006	2.2	0.54	1	b2 -H2O -NH3 [2|3][1-3][2|3][1-2]	C16H14N2O2
276.0804	276.0801	0.0002	0.8	0.31	1	y3 -H2O -NH3 [6|7][1-6][2|3][4-6]	C13H13N3O2S
276.0804	276.0801	0.0002	0.8	0.31	1	b3 -H2O -NH3 [4|5][1-4][2|3][1-3]	C13H13N3O2S
276.0804	276.0801	0.0002	0.8	0.31	1	b3 -H2O -NH3 [6|7][1-6][5|6][1-3]	C13H13N3O2S
276.0804	276.0801	0.0002	0.8	0.31	1	y3 -H2O -NH3 [4|5][1-4][1|2][2-4]	C13H13N3O2S
281.1071	281.1067	0.0004	1.4	2.19	1	y3 -CO [1|2][5-7]	C12H16N4O2S
281.1071	281.1067	0.0004	1.4	2.19	1	y3 -CO -COCH2 [5|6][1-4][1|2][2-4]	C12H16N4O2S
281.1071	281.1067	0.0004	1.4	2.19	1	b3 -CO [5|6][1-3]	C12H16N4O2S
281.1071	281.1067	0.0004	1.4	2.19	1	y3 -CO -COCH2 [7|1][1-6][2|3][4-6]	C12H16N4O2S
281.1071	281.1067	0.0004	1.4	2.19	1	b3 -CO -COCH2 [7|1][1-6][5|6][1-3]	C12H16N4O2S
281.1071	281.1067	0.0004	1.4	2.19	1	b3 -CO -COCH2 [5|6][1-4][2|3][1-3]	C12H16N4O2S
283.0864	283.0859	0.0004	1.5	0.70	1	b3 -C10H9NO [6|7][1-4][3|4][1-3]	C11H14N4O3S
283.0864	283.0859	0.0004	1.5	0.70	1	b3 -C10H9NO [2|3][1-6][4|5][1-3]	C11H14N4O3S
283.0864	283.0859	0.0004	1.5	0.70	1	b3 [6|7][1-5][4|5][1-3]	C11H14N4O3S
283.0864	283.0859	0.0004	1.5	0.70	1	y3 [6|7][1-5][2|3][3-5]	C11H14N4O3S
283.0864	283.0859	0.0004	1.5	0.70	1	b3 -COCH2 [3|4][1-6][4|5][1-3]	C11H14N4O3S
283.0864	283.0859	0.0004	1.5	0.70	1	y3 -C10H9NO -COCH2 [3|4][5-7]	C11H14N4O3S
283.0864	283.0859	0.0004	1.5	0.70	1	y3 -C10H9NO [2|3][1-6][1|2][4-6]	C11H14N4O3S
283.0864	283.0859	0.0004	1.5	0.70	1	b3 -COCH2 [7|1][1-4][3|4][1-3]	C11H14N4O3S
283.0864	283.0859	0.0004	1.5	0.70	1	y3 -COCH2 [7|1][1-4][2|3][2-4]	C11H14N4O3S
283.0864	283.0859	0.0004	1.5	0.70	1	b3 [3|4][1-5][3|4][1-3]	C11H14N4O3S
283.0864	283.0859	0.0004	1.5	0.70	1	y3 -COCH2 [3|4][1-6][1|2][4-6]	C11H14N4O3S
283.0864	283.0859	0.0004	1.5	0.70	1	y3 -C10H9NO [6|7][1-4][2|3][2-4]	C11H14N4O3S
283.0864	283.0859	0.0004	1.5	0.70	1	b3 -C10H9NO -COCH2 [7|1][1-3]	C11H14N4O3S
283.0864	283.0859	0.0004	1.5	0.70	1	y3 [3|4][1-5][1|2][3-5]	C11H14N4O3S
283.1082	283.1077	0.0005	1.6	0.84	1	y2 -NH3 [5|6][1-5][1|2][4-5]	C16H14N2O3
283.1082	283.1077	0.0005	1.6	0.84	1	b2 -NH3 [2|3][1-4][3|4][1-2]	C16H14N2O3
283.1082	283.1077	0.0005	1.6	0.84	1	y2 -NH3 [2|3][1-4][1|2][3-4]	C16H14N2O3
283.1082	283.1077	0.0005	1.6	0.84	1	b2 -NH3 [5|6][1-5][4|5][1-2]	C16H14N2O3
293.1072	293.1067	0.0006	1.9	0.64	1	y3 -H2O [4|5][1-4][1|2][2-4]	C13H16N4O2S
293.1072	293.1067	0.0006	1.9	0.64	1	y3 -COCH2 -H2O [7|1][1-5][2|3][3-5]	C13H16N4O2S
293.1072	293.1067	0.0006	1.9	0.64	1	y3 -H2O -CO [2|3][5-7]	C13H16N4O2S
293.1072	293.1067	0.0006	1.9	0.64	1	y3 -H2O [6|7][1-6][2|3][4-6]	C13H16N4O2S
293.1072	293.1067	0.0006	1.9	0.64	1	b3 -H2O [4|5][1-4][2|3][1-3]	C13H16N4O2S
293.1072	293.1067	0.0006	1.9	0.64	1	b3 -H2O [6|7][1-6][5|6][1-3]	C13H16N4O2S
293.1072	293.1067	0.0006	1.9	0.64	1	b3 -H2O -CO [6|7][1-3]	C13H16N4O2S
293.1072	293.1067	0.0006	1.9	0.64	1	b3 -COCH2 -H2O [4|5][1-5][3|4][1-3]	C13H16N4O2S
293.1072	293.1067	0.0006	1.9	0.64	1	y3 -COCH2 -H2O [4|5][1-5][1|2][3-5]	C13H16N4O2S
293.1072	293.1067	0.0006	1.9	0.64	1	b3 -COCH2 -H2O [7|1][1-5][4|5][1-3]	C13H16N4O2S
297.1561	297.1557	0.0004	1.2	0.66	1	b4 -C10H9NO -CO [5|6][1-5][3|4][1-4]	C13H20N4O4
297.1561	297.1557	0.0004	1.2	0.66	1	b4 -COCH2 [3|4][1-6][5|6][1-4]	C13H20N4O4
297.1561	297.1557	0.0004	1.2	0.66	1	y4 -C10H9NO -COCH2 [4|5][1-6][2|3][3-6]	C13H20N4O4
297.1561	297.1557	0.0004	1.2	0.66	1	b4 -COCH2 [1|2][1-5][2|3][1-4]	C13H20N4O4
297.1561	297.1557	0.0004	1.2	0.66	1	b4 -C10H9NO -CO [1|2][1-6][4|5][1-4]	C13H20N4O4
297.1561	297.1557	0.0004	1.2	0.66	1	b4 -C10H9NO -COCH2 [4|5][1-6][4|5][1-4]	C13H20N4O4

Meas. m/z	Calc. m/z	δ (Da)	δ (ppm)	Rel. Int. (%)	z	Annotation	Formula
297.1561	297.1557	0.0004	1.2	0.66	1	b4 -C10H9NO [4|5][1-6][5|6][1-4]	C13H20N4O4
297.1561	297.1557	0.0004	1.2	0.66	1	y4 -C10H9NO [2|3][1-5][1|2][2-5]	C13H20N4O4
297.1561	297.1557	0.0004	1.2	0.66	1	y4 -C10H9NO -COCH2 [1|2][1-5][2|3][2-5]	C13H20N4O4
297.1561	297.1557	0.0004	1.2	0.66	1	b4 -C10H9NO [2|3][1-5][2|3][1-4]	C13H20N4O4
297.1561	297.1557	0.0004	1.2	0.66	1	y4 -C10H9NO -CO [1|2][1-6][2|3][3-6]	C13H20N4O4
297.1561	297.1557	0.0004	1.2	0.66	1	y4 -C10H9NO [4|5][1-6][3|4][3-6]	C13H20N4O4
297.1561	297.1557	0.0004	1.2	0.66	1	y4 -C10H9NO -CO [5|6][1-5][2|3][2-5]	C13H20N4O4
297.1561	297.1557	0.0004	1.2	0.66	1	b4 -C10H9NO -COCH2 [1|2][1-5][3|4][1-4]	C13H20N4O4
297.1561	297.1557	0.0004	1.2	0.66	1	y4 -COCH2 [3|4][1-6][3|4][3-6]	C13H20N4O4
297.1561	297.1557	0.0004	1.2	0.66	1	y4 -COCH2 [1|2][1-5][1|2][2-5]	C13H20N4O4
297.1561	297.1557	0.0004	1.2	0.66	1	y4 [7|1][4-7]	C13H20N4O4
297.1561	297.1557	0.0004	1.2	0.66	1	b4 [3|4][1-4]	C13H20N4O4
307.1404	307.1401	0.0004	1.2	4.30	1	b4 -C10H9NO -H2O [1|2][1-6][4|5][1-4]	C14H18N4O4
307.1404	307.1401	0.0004	1.2	4.30	1	y4 -C10H9NO -H2O [5|6][1-5][2|3][2-5]	C14H18N4O4
307.1404	307.1401	0.0004	1.2	4.30	1	y4 -C10H9NO -H2O [1|2][1-6][2|3][3-6]	C14H18N4O4
307.1404	307.1401	0.0004	1.2	4.30	1	b4 -C10H9NO -H2O [5|6][1-5][3|4][1-4]	C14H18N4O4
309.1020	309.1016	0.0004	1.2	2.45	1	b3 [5|6][1-3]	C13H16N4O3S
309.1020	309.1016	0.0004	1.2	2.45	1	y3 [1|2][5-7]	C13H16N4O3S
309.1020	309.1016	0.0004	1.2	2.45	1	y3 -COCH2 [7|1][1-6][2|3][4-6]	C13H16N4O3S
309.1020	309.1016	0.0004	1.2	2.45	1	b3 -COCH2 [7|1][1-6][5|6][1-3]	C13H16N4O3S
309.1020	309.1016	0.0004	1.2	2.45	1	y3 -COCH2 [5|6][1-4][1|2][2-4]	C13H16N4O3S
309.1020	309.1016	0.0004	1.2	2.45	1	b3 -COCH2 [5|6][1-4][2|3][1-3]	C13H16N4O3S
313.1187	313.1183	0.0004	1.2	0.28	1	y2 -NH3 [3|4][6-7]	C17H16N2O4
313.1187	313.1183	0.0004	1.2	0.28	1	b2 -NH3 [1|2][1-2]	C17H16N2O4
321.1018	321.1016	0.0002	0.7	0.44	1	b3 -H2O [6|7][1-3]	C14H16N4O3S
321.1018	321.1016	0.0002	0.7	0.44	1	y3 -H2O [2|3][5-7]	C14H16N4O3S
321.1018	321.1016	0.0002	0.7	0.44	1	b4 -NH3 -CO [3|4][1-5][2|3][1-4]	C14H16N4O3S
321.1018	321.1016	0.0002	0.7	0.44	1	y4 -NH3 -CO [5|6][1-6][3|4][3-6]	C14H16N4O3S
321.1018	321.1016	0.0002	0.7	0.44	1	y4 -NH3 -CO [3|4][1-5][1|2][2-5]	C14H16N4O3S
321.1018	321.1016	0.0002	0.7	0.44	1	b4 -NH3 -CO [5|6][1-6][5|6][1-4]	C14H16N4O3S
321.1562	321.1557	0.0004	1.3	0.27	1	y4 -H2O [1|2][1-5][1|2][2-5]	C15H20N4O4
321.1562	321.1557	0.0004	1.3	0.27	1	b4 -C10H9NO -H2O [1|2][1-5][3|4][1-4]	C15H20N4O4
321.1562	321.1557	0.0004	1.3	0.27	1	y4 -H2O [3|4][1-6][3|4][3-6]	C15H20N4O4
321.1562	321.1557	0.0004	1.3	0.27	1	y4 -C10H9NO -H2O [4|5][1-6][2|3][3-6]	C15H20N4O4
321.1562	321.1557	0.0004	1.3	0.27	1	y4 -C10H9NO -H2O [1|2][1-5][2|3][2-5]	C15H20N4O4
321.1562	321.1557	0.0004	1.3	0.27	1	b4 -H2O [1|2][1-5][2|3][1-4]	C15H20N4O4
321.1562	321.1557	0.0004	1.3	0.27	1	b4 -H2O [3|4][1-6][5|6][1-4]	C15H20N4O4
321.1562	321.1557	0.0004	1.3	0.27	1	b4 -C10H9NO -H2O [4|5][1-6][4|5][1-4]	C15H20N4O4
324.1347	324.1343	0.0005	1.4	1.92	1	y3 -H2O -NH3 [5|6][5-7]	C18H17N3O3
324.1347	324.1343	0.0005	1.4	1.92	1	b3 -H2O -NH3 [2|3][1-3]	C18H17N3O3
325.1121	325.1118	0.0004	1.1	10.51	1	y2 -H2O -CO [7|1][1-3][1|2][2-3]	C17H16N4OS
325.1121	325.1118	0.0004	1.1	10.51	1	b2 -H2O -CO [7|1][1-3][2|3][1-2]	C17H16N4OS
325.1121	325.1118	0.0004	1.1	10.51	1	y2 -H2O -CO [2|3][1-6][1|2][5-6]	C17H16N4OS
325.1121	325.1118	0.0004	1.1	10.51	1	b2 -H2O -CO [2|3][1-6][5|6][1-2]	C17H16N4OS
326.0960	326.0958	0.0003	0.8	3.70	1	b2 -NH3 -CO [2|3][1-6][5|6][1-2]	C17H15N3O2S
326.0960	326.0958	0.0003	0.8	3.70	1	b2 -NH3 -CO [7|1][1-3][2|3][1-2]	C17H15N3O2S
326.0960	326.0958	0.0003	0.8	3.70	1	y2 -NH3 -CO [2|3][1-6][1|2][5-6]	C17H15N3O2S
326.0960	326.0958	0.0003	0.8	3.70	1	y2 -NH3 -CO [7|1][1-3][1|2][2-3]	C17H15N3O2S
326.1285	326.1281	0.0004	1.1	0.58	1	y4 -C10H9NO -CO [7|1][1-5][1|2][2-5]	C13H19N5O3S
326.1285	326.1281	0.0004	1.1	0.58	1	b4 -CO -COCH2 [6|7][1-5][4|5][1-4]	C13H19N5O3S
326.1285	326.1281	0.0004	1.1	0.58	1	y4 -CO -COCH2 [6|7][1-5][3|4][2-5]	C13H19N5O3S

Meas. m/z	Calc. m/z	δ (Da)	δ (ppm)	Rel. Int. (%)	z	Annotation	Formula
326.1285	326.1281	0.0004	1.1	0.58	1	b4 -CO -COCH2 [3|4][1-6][3|4][1-4]	C13H19N5O3S
326.1285	326.1281	0.0004	1.1	0.58	1	y4 -CO -COCH2 [3|4][1-6][1|2][3-6]	C13H19N5O3S
326.1285	326.1281	0.0004	1.1	0.58	1	b4 -C10H9NO -CO [2|3][1-6][5|6][1-4]	C13H19N5O3S
326.1285	326.1281	0.0004	1.1	0.58	1	y4 -C10H9NO -CO [2|3][1-6][3|4][3-6]	C13H19N5O3S
326.1285	326.1281	0.0004	1.1	0.58	1	b4 -C10H9NO -CO [7|1][1-5][2|3][1-4]	C13H19N5O3S
326.1504	326.1499	0.0005	1.4	0.66	1	b3 -NH3 -CO [5|6][1-5][4|5][1-3]	C18H19N3O3
326.1504	326.1499	0.0005	1.4	0.66	1	y3 -NH3 -CO [2|3][1-5][1|2][3-5]	C18H19N3O3
326.1504	326.1499	0.0005	1.4	0.66	1	b3 -NH3 -CO [2|3][1-5][3|4][1-3]	C18H19N3O3
326.1504	326.1499	0.0005	1.4	0.66	1	y3 -NH3 -CO [5|6][1-5][2|3][3-5]	C18H19N3O3
337.0969	337.0965	0.0004	1.3	0.29	1	b4 -C10H9NO -NH3 [2|3][1-6][5|6][1-4]	C14H16N4O4S
337.0969	337.0965	0.0004	1.3	0.29	1	y4 -C10H9NO -NH3 [7|1][1-5][1|2][2-5]	C14H16N4O4S
337.0969	337.0965	0.0004	1.3	0.29	1	b4 -C10H9NO -NH3 [7|1][1-5][2|3][1-4]	C14H16N4O4S
337.0969	337.0965	0.0004	1.3	0.29	1	y4 -COCH2 -NH3 [6|7][1-5][3|4][2-5]	C14H16N4O4S
337.0969	337.0965	0.0004	1.3	0.29	1	b4 -COCH2 -NH3 [6|7][1-5][4|5][1-4]	C14H16N4O4S
337.0969	337.0965	0.0004	1.3	0.29	1	y4 -COCH2 -NH3 [3|4][1-6][1|2][3-6]	C14H16N4O4S
337.0969	337.0965	0.0004	1.3	0.29	1	b4 -COCH2 -NH3 [3|4][1-6][3|4][1-4]	C14H16N4O4S
337.0969	337.0965	0.0004	1.3	0.29	1	y4 -C10H9NO -NH3 [2|3][1-6][3|4][3-6]	C14H16N4O4S
339.1667	339.1663	0.0004	1.1	0.64	1	y4 [3|4][1-6][3|4][3-6]	C15H22N4O5
339.1667	339.1663	0.0004	1.1	0.64	1	b4 [1|2][1-5][2|3][1-4]	C15H22N4O5
339.1667	339.1663	0.0004	1.1	0.64	1	y4 [1|2][1-5][1|2][2-5]	C15H22N4O5
339.1667	339.1663	0.0004	1.1	0.64	1	y4 -C10H9NO [1|2][1-5][2|3][2-5]	C15H22N4O5
339.1667	339.1663	0.0004	1.1	0.64	1	y4 -C10H9NO [4|5][1-6][2|3][3-6]	C15H22N4O5
339.1667	339.1663	0.0004	1.1	0.64	1	b4 -C10H9NO [1|2][1-5][3|4][1-4]	C15H22N4O5
339.1667	339.1663	0.0004	1.1	0.64	1	b4 [3|4][1-6][5|6][1-4]	C15H22N4O5
339.1667	339.1663	0.0004	1.1	0.64	1	b4 -C10H9NO [4|5][1-6][4|5][1-4]	C15H22N4O5
340.1661	340.1656	0.0005	1.6	0.16	1	b3 -NH3 -CO [4|5][1-6][5|6][1-3]	C19H21N3O3
340.1661	340.1656	0.0005	1.6	0.16	1	b3 -NH3 -CO [2|3][1-4][2|3][1-3]	C19H21N3O3
340.1661	340.1656	0.0005	1.6	0.16	1	y3 -NH3 -CO [2|3][1-4][1|2][2-4]	C19H21N3O3
340.1661	340.1656	0.0005	1.6	0.16	1	y3 -NH3 -CO [4|5][1-6][2|3][4-6]	C19H21N3O3
342.1450	342.1448	0.0001	0.4	1.08	1	b3 -COCH2 -NH3 [6|7][1-4][2|3][1-3]	C18H19N3O4
342.1450	342.1448	0.0001	0.4	1.08	1	b3 -COCH2 -NH3 [1|2][1-6][5|6][1-3]	C18H19N3O4
342.1450	342.1448	0.0001	0.4	1.08	1	b3 -NH3 -CO [1|2][1-3]	C18H19N3O4
342.1450	342.1448	0.0001	0.4	1.08	1	y3 -NH3 [5|6][5-7]	C18H19N3O4
342.1450	342.1448	0.0001	0.4	1.08	1	y3 -COCH2 -NH3 [1|2][1-6][2|3][4-6]	C18H19N3O4
342.1450	342.1448	0.0001	0.4	1.08	1	y3 -COCH2 -NH3 [6|7][1-4][1|2][2-4]	C18H19N3O4
342.1450	342.1448	0.0001	0.4	1.08	1	b3 -NH3 [2|3][1-3]	C18H19N3O4
342.1450	342.1448	0.0001	0.4	1.08	1	y3 -NH3 -CO [4|5][5-7]	C18H19N3O4
343.1227	343.1223	0.0004	1.1	7.08	1	b2 -CO [7|1][1-3][2|3][1-2]	C17H18N4O2S
343.1227	343.1223	0.0004	1.1	7.08	1	y2 -CO [2|3][1-6][1|2][5-6]	C17H18N4O2S
343.1227	343.1223	0.0004	1.1	7.08	1	y2 -CO [7|1][1-3][1|2][2-3]	C17H18N4O2S
343.1227	343.1223	0.0004	1.1	7.08	1	b2 -CO [2|3][1-6][5|6][1-2]	C17H18N4O2S
349.0969	349.0965	0.0004	1.0	0.53	1	y4 -NH3 [5|6][1-6][3|4][3-6]	C15H16N4O4S
349.0969	349.0965	0.0004	1.0	0.53	1	y4 -C10H9NO -NH3 [5|6][1-5][3|4][2-5]	C15H16N4O4S
349.0969	349.0965	0.0004	1.0	0.53	1	b4 -C10H9NO -NH3 [5|6][1-5][4|5][1-4]	C15H16N4O4S
349.0969	349.0965	0.0004	1.0	0.53	1	b4 -NH3 [3|4][1-5][2|3][1-4]	C15H16N4O4S
349.0969	349.0965	0.0004	1.0	0.53	1	b4 -NH3 [5|6][1-6][5|6][1-4]	C15H16N4O4S
349.0969	349.0965	0.0004	1.0	0.53	1	y4 -C10H9NO -NH3 [2|3][1-6][1|2][3-6]	C15H16N4O4S
349.0969	349.0965	0.0004	1.0	0.53	1	b4 -C10H9NO -NH3 [2|3][1-6][3|4][1-4]	C15H16N4O4S
349.0969	349.0965	0.0004	1.0	0.53	1	y4 -NH3 [3|4][1-5][1|2][2-5]	C15H16N4O4S
350.1284	350.1281	0.0003	0.9	0.92	1	b4 -H2O [6|7][1-6][4|5][1-4]	C15H19N5O3S
350.1284	350.1281	0.0003	0.9	0.92	1	y4 -H2O [6|7][1-6][2|3][3-6]	C15H19N5O3S

Meas. m/z	Calc. m/z	δ (Da)	δ (ppm)	Rel. Int. (%)	z	Annotation	Formula
350.1284	350.1281	0.0003	0.9	0.92	1	y4 -H2O -CO [3|4][1-6][1|2][3-6]	C15H19N5O3S
350.1284	350.1281	0.0003	0.9	0.92	1	b4 -COCH2 -H2O [7|1][1-5][3|4][1-4]	C15H19N5O3S
350.1284	350.1281	0.0003	0.9	0.92	1	b4 -H2O [3|4][1-5][3|4][1-4]	C15H19N5O3S
350.1284	350.1281	0.0003	0.9	0.92	1	y4 -H2O -CO [6|7][1-5][3|4][2-5]	C15H19N5O3S
350.1284	350.1281	0.0003	0.9	0.92	1	b4 -H2O -CO [3|4][1-6][3|4][1-4]	C15H19N5O3S
350.1284	350.1281	0.0003	0.9	0.92	1	y4 -COCH2 -H2O [7|1][1-5][2|3][2-5]	C15H19N5O3S
350.1284	350.1281	0.0003	0.9	0.92	1	y4 -H2O [3|4][1-5][2|3][2-5]	C15H19N5O3S
350.1284	350.1281	0.0003	0.9	0.92	1	b4 -H2O -CO [6|7][1-5][4|5][1-4]	C15H19N5O3S
350.1284	350.1281	0.0003	0.9	0.92	1	y4 -COCH2 -H2O [3|4][1-6][2|3][3-6]	C15H19N5O3S
350.1284	350.1281	0.0003	0.9	0.92	1	b4 -COCH2 -H2O [3|4][1-6][4|5][1-4]	C15H19N5O3S
352.1442	352.1438	0.0004	1.1	8.68	1	b4 -CO [7|1][1-6][3|4][1-4]	C15H21N5O3S
352.1442	352.1438	0.0004	1.1	8.68	1	b4 -CO [3|4][1-5][4|5][1-4]	C15H21N5O3S
352.1442	352.1438	0.0004	1.1	8.68	1	b4 -CO -COCH2 [5|6][1-4]	C15H21N5O3S
352.1442	352.1438	0.0004	1.1	8.68	1	y4 -CO [7|1][1-6][1|2][3-6]	C15H21N5O3S
352.1442	352.1438	0.0004	1.1	8.68	1	y4 -CO [3|4][1-5][3|4][2-5]	C15H21N5O3S
352.1442	352.1438	0.0004	1.1	8.68	1	y4 -CO -COCH2 [2|3][4-7]	C15H21N5O3S
353.1071	353.1067	0.0005	1.3	0.14	1	y2 -H2O [7|1][1-3][1|2][2-3]	C18H16N4O2S
353.1071	353.1067	0.0005	1.3	0.14	1	b2 -H2O [7|1][1-3][2|3][1-2]	C18H16N4O2S
353.1071	353.1067	0.0005	1.3	0.14	1	y2 -H2O [2|3][1-6][1|2][5-6]	C18H16N4O2S
353.1071	353.1067	0.0005	1.3	0.14	1	b2 -H2O [2|3][1-6][5|6][1-2]	C18H16N4O2S
354.0909	354.0907	0.0002	0.5	0.91	1	b2 -NH3 [2|3][1-6][5|6][1-2]	C18H15N3O3S
354.0909	354.0907	0.0002	0.5	0.91	1	b2 -NH3 [7|1][1-3][2|3][1-2]	C18H15N3O3S
354.0909	354.0907	0.0002	0.5	0.91	1	y2 -NH3 [2|3][1-6][1|2][5-6]	C18H15N3O3S
354.0909	354.0907	0.0002	0.5	0.91	1	y2 -NH3 [7|1][1-3][1|2][2-3]	C18H15N3O3S
361.0970	361.0965	0.0005	1.3	2.47	1	y4 -H2O -NH3 [3|4][1-6][1|2][3-6]	C16H16N4O4S
361.0970	361.0965	0.0005	1.3	2.47	1	y4 -H2O -NH3 [6|7][1-5][3|4][2-5]	C16H16N4O4S
361.0970	361.0965	0.0005	1.3	2.47	1	b4 -H2O -NH3 [6|7][1-5][4|5][1-4]	C16H16N4O4S
361.0970	361.0965	0.0005	1.3	2.47	1	b4 -H2O -NH3 [3|4][1-6][3|4][1-4]	C16H16N4O4S
364.1443	364.1438	0.0005	1.5	1.44	1	b4 -COCH2 -H2O [6|7][1-6][5|6][1-4]	C16H21N5O3S
364.1443	364.1438	0.0005	1.5	1.44	1	y4 -COCH2 -H2O [4|5][1-5][1|2][2-5]	C16H21N5O3S
364.1443	364.1438	0.0005	1.5	1.44	1	y4 -H2O -CO [7|1][1-5][2|3][2-5]	C16H21N5O3S
364.1443	364.1438	0.0005	1.5	1.44	1	b4 -COCH2 -H2O [4|5][1-5][2|3][1-4]	C16H21N5O3S
364.1443	364.1438	0.0005	1.5	1.44	1	b4 -H2O -CO [3|4][1-6][4|5][1-4]	C16H21N5O3S
364.1443	364.1438	0.0005	1.5	1.44	1	y4 -COCH2 -H2O [6|7][1-6][3|4][3-6]	C16H21N5O3S
364.1443	364.1438	0.0005	1.5	1.44	1	b4 -H2O -CO [7|1][1-5][3|4][1-4]	C16H21N5O3S
364.1443	364.1438	0.0005	1.5	1.44	1	y4 -H2O -CO [3|4][1-6][2|3][3-6]	C16H21N5O3S
366.1235	366.1231	0.0005	1.3	0.39	1	b4 -C10H9NO [5|6][1-5][4|5][1-4]	C15H19N5O4S
366.1235	366.1231	0.0005	1.3	0.39	1	b4 -C10H9NO -COCH2 [5|6][1-5][2|3][1-4]	C15H19N5O4S
366.1235	366.1231	0.0005	1.3	0.39	1	y4 [5|6][1-6][3|4][3-6]	C15H19N5O4S
366.1235	366.1231	0.0005	1.3	0.39	1	y4 -C10H9NO [5|6][1-5][3|4][2-5]	C15H19N5O4S
366.1235	366.1231	0.0005	1.3	0.39	1	y4 -C10H9NO [2|3][1-6][1|2][3-6]	C15H19N5O4S
366.1235	366.1231	0.0005	1.3	0.39	1	b4 [3|4][1-5][2|3][1-4]	C15H19N5O4S
366.1235	366.1231	0.0005	1.3	0.39	1	y4 -C10H9NO -COCH2 [5|6][1-5][1|2][2-5]	C15H19N5O4S
366.1235	366.1231	0.0005	1.3	0.39	1	b4 -C10H9NO [2|3][1-6][3|4][1-4]	C15H19N5O4S
366.1235	366.1231	0.0005	1.3	0.39	1	b4 -C10H9NO -COCH2 [7|1][1-6][5|6][1-4]	C15H19N5O4S
366.1235	366.1231	0.0005	1.3	0.39	1	y4 [3|4][1-5][1|2][2-5]	C15H19N5O4S
366.1235	366.1231	0.0005	1.3	0.39	1	b4 [5|6][1-6][5|6][1-4]	C15H19N5O4S
366.1235	366.1231	0.0005	1.3	0.39	1	y4 -C10H9NO -COCH2 [7|1][1-6][3|4][3-6]	C15H19N5O4S
366.1598	366.1594	0.0003	0.9	0.15	1	y4 -CO -COCH2 [7|1][1-6][2|3][3-6]	C16H23N5O3S
366.1598	366.1594	0.0003	0.9	0.15	1	b4 -CO -COCH2 [7|1][1-6][4|5][1-4]	C16H23N5O3S
366.1598	366.1594	0.0003	0.9	0.15	1	b4 -CO [4|5][1-4]	C16H23N5O3S

Meas. m/z	Calc. m/z	δ (Da)	δ (ppm)	Rel. Int. (%)	z	Annotation	Formula
366.1598	366.1594	0.0003	0.9	0.15	1	y4 -CO -COCH2 [4|5][1-5][2|3][2-5]	C16H23N5O3S
366.1598	366.1594	0.0003	0.9	0.15	1	b4 -CO -COCH2 [4|5][1-5][3|4][1-4]	C16H23N5O3S
366.1598	366.1594	0.0003	0.9	0.15	1	y4 -CO [1|2][4-7]	C16H23N5O3S
370.1400	370.1397	0.0003	0.8	0.86	1	y3 -NH3 [4|5][5-7]	C19H19N3O5
370.1400	370.1397	0.0003	0.8	0.86	1	b3 -NH3 [1|2][1-3]	C19H19N3O5
371.1176	371.1172	0.0003	0.9	3.14	1	y2 [7|1][1-3][1|2][2-3]	C18H18N4O3S
371.1176	371.1172	0.0003	0.9	3.14	1	b2 [2|3][1-6][5|6][1-2]	C18H18N4O3S
371.1176	371.1172	0.0003	0.9	3.14	1	y2 [2|3][1-6][1|2][5-6]	C18H18N4O3S
371.1176	371.1172	0.0003	0.9	3.14	1	b2 [7|1][1-3][2|3][1-2]	C18H18N4O3S
377.1283	377.1278	0.0005	1.2	0.57	1	b4 -COCH2 -NH3 [7|1][1-6][4|5][1-4]	C17H20N4O4S
377.1283	377.1278	0.0005	1.2	0.57	1	b4 -COCH2 -NH3 [4|5][1-5][3|4][1-4]	C17H20N4O4S
377.1283	377.1278	0.0005	1.2	0.57	1	y4 -COCH2 -NH3 [4|5][1-5][2|3][2-5]	C17H20N4O4S
377.1283	377.1278	0.0005	1.2	0.57	1	y4 -COCH2 -NH3 [7|1][1-6][2|3][3-6]	C17H20N4O4S
377.1283	377.1278	0.0005	1.2	0.57	1	b4 -NH3 [4|5][1-4]	C17H20N4O4S
377.1283	377.1278	0.0005	1.2	0.57	1	y4 -NH3 -CO [2|3][4-7]	C17H20N4O4S
377.1283	377.1278	0.0005	1.2	0.57	1	y4 -NH3 [1|2][4-7]	C17H20N4O4S
377.1283	377.1278	0.0005	1.2	0.57	1	b4 -NH3 -CO [5|6][1-4]	C17H20N4O4S
382.2089	382.2085	0.0004	1.1	0.25	1	b5 -C10H9NO -CO [4|5][1-6][4|5][1-5]	C17H27N5O5
382.2089	382.2085	0.0004	1.1	0.25	1	b5 -CO [3|4][1-6][5|6][1-5]	C17H27N5O5
382.2089	382.2085	0.0004	1.1	0.25	1	b5 -C10H9NO -CO [1|2][1-6][3|4][1-5]	C17H27N5O5
382.2089	382.2085	0.0004	1.1	0.25	1	y5 -CO [3|4][1-6][4|5][2-6]	C17H27N5O5
382.2089	382.2085	0.0004	1.1	0.25	1	y5 -C10H9NO -CO [1|2][1-6][2|3][2-6]	C17H27N5O5
382.2089	382.2085	0.0004	1.1	0.25	1	y5 -C10H9NO -CO [4|5][1-6][3|4][2-6]	C17H27N5O5
382.2089	382.2085	0.0004	1.1	0.25	1	y5 -CO [1|2][1-6][1|2][2-6]	C17H27N5O5
382.2089	382.2085	0.0004	1.1	0.25	1	b5 -CO [1|2][1-6][2|3][1-5]	C17H27N5O5
383.1177	383.1172	0.0004	1.1	0.23	1	y3 -NH3 -CO [2|3][1-6][2|3][4-6]	C19H18N4O3S
383.1177	383.1172	0.0004	1.1	0.23	1	b3 -NH3 -CO [2|3][1-6][5|6][1-3]	C19H18N4O3S
383.1177	383.1172	0.0004	1.1	0.23	1	y3 -NH3 -CO [7|1][1-4][1|2][2-4]	C19H18N4O3S
383.1177	383.1172	0.0004	1.1	0.23	1	b3 -NH3 -CO [7|1][1-4][2|3][1-3]	C19H18N4O3S
390.1598	390.1594	0.0003	0.8	0.59	1	y4 -H2O -CO [7|1][1-6][2|3][3-6]	C18H23N5O3S
390.1598	390.1594	0.0003	0.8	0.59	1	b4 -H2O -CO [4|5][1-5][3|4][1-4]	C18H23N5O3S
390.1598	390.1594	0.0003	0.8	0.59	1	b4 -H2O -CO [7|1][1-6][4|5][1-4]	C18H23N5O3S
390.1598	390.1594	0.0003	0.8	0.59	1	y4 -H2O -CO [4|5][1-5][2|3][2-5]	C18H23N5O3S
392.1935	392.1928	0.0007	1.7	0.26	1	y5 -H2O [1|2][1-6][1|2][2-6]	C18H25N5O5
392.1935	392.1928	0.0007	1.7	0.26	1	y5 -H2O [3|4][1-6][4|5][2-6]	C18H25N5O5
392.1935	392.1928	0.0007	1.7	0.26	1	b5 -C10H9NO -H2O [1|2][1-6][3|4][1-5]	C18H25N5O5
392.1935	392.1928	0.0007	1.7	0.26	1	b5 -H2O [1|2][1-6][2|3][1-5]	C18H25N5O5
392.1935	392.1928	0.0007	1.7	0.26	1	b5 -C10H9NO -H2O [4|5][1-6][4|5][1-5]	C18H25N5O5
392.1935	392.1928	0.0007	1.7	0.26	1	y5 -C10H9NO -H2O [1|2][1-6][2|3][2-6]	C18H25N5O5
392.1935	392.1928	0.0007	1.7	0.26	1	b5 -H2O [3|4][1-6][5|6][1-5]	C18H25N5O5
392.1935	392.1928	0.0007	1.7	0.26	1	y5 -C10H9NO -H2O [4|5][1-6][3|4][2-6]	C18H25N5O5
395.1501	395.1496	0.0005	1.2	0.51	1	b5 -C10H9NO -CO [2|3][1-6][3|4][1-5]	C16H22N6O4S
395.1501	395.1496	0.0005	1.2	0.51	1	y5 -C10H9NO -CO [2|3][1-6][2|3][2-6]	C16H22N6O4S
395.1501	395.1496	0.0005	1.2	0.51	1	y5 -C10H9NO -CO [5|6][1-6][3|4][2-6]	C16H22N6O4S
395.1501	395.1496	0.0005	1.2	0.51	1	b5 -C10H9NO -CO [5|6][1-6][4|5][1-5]	C16H22N6O4S
396.1341	396.1336	0.0005	1.2	2.09	1	b4 -C10H9NO [6|7][1-4]	C16H21N5O5S
396.1341	396.1336	0.0005	1.2	2.09	1	b4 [6|7][1-5][4|5][1-4]	C16H21N5O5S
396.1341	396.1336	0.0005	1.2	2.09	1	y4 -C10H9NO [3|4][4-7]	C16H21N5O5S
396.1341	396.1336	0.0005	1.2	2.09	1	b4 [3|4][1-6][3|4][1-4]	C16H21N5O5S
396.1341	396.1336	0.0005	1.2	2.09	1	y4 [6|7][1-5][3|4][2-5]	C16H21N5O5S
396.1341	396.1336	0.0005	1.2	2.09	1	y4 [3|4][1-6][1|2][3-6]	C16H21N5O5S

Meas. m/z	Calc. m/z	δ (Da)	δ (ppm)	Rel. Int. (%)	z	Annotation	Formula
396.1493	396.1489	0.0004	1.1	36.12	1	b3 -H2O -CO [6|7][1-4][3|4][1-3]	C20H21N5O2S
396.1493	396.1489	0.0004	1.1	36.12	1	b3 -H2O -CO [2|3][1-6][4|5][1-3]	C20H21N5O2S
396.1493	396.1489	0.0004	1.1	36.12	1	y3 -H2O -CO [6|7][1-4][2|3][2-4]	C20H21N5O2S
396.1493	396.1489	0.0004	1.1	36.12	1	y3 -H2O -CO [2|3][1-6][1|2][4-6]	C20H21N5O2S
397.1335	397.1329	0.0007	1.7	0.25	1	y3 -NH3 -CO [2|3][1-6][1|2][4-6]	C20H20N4O3S
397.1335	397.1329	0.0007	1.7	0.25	1	b3 -NH3 -CO [2|3][1-6][4|5][1-3]	C20H20N4O3S
397.1335	397.1329	0.0007	1.7	0.25	1	b3 -NH3 -CO [6|7][1-4][3|4][1-3]	C20H20N4O3S
397.1335	397.1329	0.0007	1.7	0.25	1	y3 -NH3 -CO [6|7][1-4][2|3][2-4]	C20H20N4O3S
406.1544	406.1544	0.0001	0.2	0.25	1	b4 -H2O [4|5][1-5][2|3][1-4]	C18H23N5O4S
406.1544	406.1544	0.0001	0.2	0.25	1	b4 -H2O [6|7][1-6][5|6][1-4]	C18H23N5O4S
406.1544	406.1544	0.0001	0.2	0.25	1	y4 -H2O [6|7][1-6][3|4][3-6]	C18H23N5O4S
406.1544	406.1544	0.0001	0.2	0.25	1	y5 -NH3 -CO [1|2][3-7]	C18H23N5O4S
406.1544	406.1544	0.0001	0.2	0.25	1	b5 -NH3 -CO [3|4][1-5]	C18H23N5O4S
406.1544	406.1544	0.0001	0.2	0.25	1	y4 -H2O [4|5][1-5][1|2][2-5]	C18H23N5O4S
407.1505	407.1496	0.0009	2.3	0.14	1	y5 -C10H9NO -H2O [6|7][1-6][2|3][2-6]	C17H22N6O4S
407.1505	407.1496	0.0009	2.3	0.14	1	b5 -C10H9NO -H2O [6|7][1-6][3|4][1-5]	C17H22N6O4S
407.1505	407.1496	0.0009	2.3	0.14	1	b5 -C10H9NO -H2O [2|3][1-6][4|5][1-5]	C17H22N6O4S
407.1505	407.1496	0.0009	2.3	0.14	1	y5 -C10H9NO -H2O [2|3][1-6][3|4][2-6]	C17H22N6O4S
407.1721	407.1714	0.0007	1.7	0.28	1	y4 -H2O -NH3 [6|7][4-7]	C22H22N4O4
407.1721	407.1714	0.0007	1.7	0.28	1	b4 -H2O -NH3 [2|3][1-4]	C22H22N4O4
408.1343	408.1336	0.0007	1.7	0.58	1	y4 -C10H9NO [7|1][1-6][3|4][3-6]	C17H21N5O5S
408.1343	408.1336	0.0007	1.7	0.58	1	y5 -C10H9NO -NH3 [2|3][1-6][3|4][2-6]	C17H21N5O5S
408.1343	408.1336	0.0007	1.7	0.58	1	b4 -C10H9NO [7|1][1-6][5|6][1-4]	C17H21N5O5S
408.1343	408.1336	0.0007	1.7	0.58	1	b5 -C10H9NO -NH3 [6|7][1-6][3|4][1-5]	C17H21N5O5S
408.1343	408.1336	0.0007	1.7	0.58	1	y4 -C10H9NO [5|6][1-5][1|2][2-5]	C17H21N5O5S
408.1343	408.1336	0.0007	1.7	0.58	1	y5 -C10H9NO -NH3 [6|7][1-6][2|3][2-6]	C17H21N5O5S
408.1343	408.1336	0.0007	1.7	0.58	1	b5 -C10H9NO -NH3 [2|3][1-6][4|5][1-5]	C17H21N5O5S
408.1343	408.1336	0.0007	1.7	0.58	1	b4 -C10H9NO [5|6][1-5][2|3][1-4]	C17H21N5O5S
410.2037	410.2034	0.0003	0.7	1.47	1	b5 [1|2][1-6][2|3][1-5]	C18H27N5O6
410.2037	410.2034	0.0003	0.7	1.47	1	y5 [1|2][1-6][1|2][2-6]	C18H27N5O6
410.2037	410.2034	0.0003	0.7	1.47	1	y5 [3|4][1-6][4|5][2-6]	C18H27N5O6
410.2037	410.2034	0.0003	0.7	1.47	1	b5 -C10H9NO [1|2][1-6][3|4][1-5]	C18H27N5O6
410.2037	410.2034	0.0003	0.7	1.47	1	y5 -C10H9NO [1|2][1-6][2|3][2-6]	C18H27N5O6
410.2037	410.2034	0.0003	0.7	1.47	1	y5 -C10H9NO [4|5][1-6][3|4][2-6]	C18H27N5O6
410.2037	410.2034	0.0003	0.7	1.47	1	b5 [3|4][1-6][5|6][1-5]	C18H27N5O6
410.2037	410.2034	0.0003	0.7	1.47	1	b5 -C10H9NO [4|5][1-6][4|5][1-5]	C18H27N5O6
411.1122	411.1122	0.0001	0.1	0.25	1	b3 -NH3 [7|1][1-4][2|3][1-3]	C20H18N4O4S
411.1122	411.1122	0.0001	0.1	0.25	1	y3 -NH3 [7|1][1-4][1|2][2-4]	C20H18N4O4S
411.1122	411.1122	0.0001	0.1	0.25	1	y3 -NH3 [2|3][1-6][2|3][4-6]	C20H18N4O4S
411.1122	411.1122	0.0001	0.1	0.25	1	b3 -NH3 [2|3][1-6][5|6][1-3]	C20H18N4O4S
413.1826	413.1819	0.0006	1.5	0.61	1	b4 -NH3 -CO [6|7][1-5][2|3][1-4]	C21H24N4O5
413.1826	413.1819	0.0006	1.5	0.61	1	y4 -NH3 [2|3][1-5][3|4][2-5]	C21H24N4O5
413.1826	413.1819	0.0006	1.5	0.61	1	b4 -COCH2 -NH3 [1|2][1-4]	C21H24N4O5
413.1826	413.1819	0.0006	1.5	0.61	1	b4 -NH3 -CO [1|2][1-6][5|6][1-4]	C21H24N4O5
413.1826	413.1819	0.0006	1.5	0.61	1	y4 -NH3 -CO [1|2][1-6][3|4][3-6]	C21H24N4O5
413.1826	413.1819	0.0006	1.5	0.61	1	b3 [5|6][1-5][3|4][1-3]	C21H24N4O5
413.1826	413.1819	0.0006	1.5	0.61	1	y3 [5|6][1-5][1|2][3-5]	C21H24N4O5
413.1826	413.1819	0.0006	1.5	0.61	1	b4 -NH3 [6|7][1-6][3|4][1-4]	C21H24N4O5
413.1826	413.1819	0.0006	1.5	0.61	1	y4 -NH3 -CO [6|7][1-5][1|2][2-5]	C21H24N4O5
413.1826	413.1819	0.0006	1.5	0.61	1	b4 -NH3 [2|3][1-5][4|5][1-4]	C21H24N4O5
413.1826	413.1819	0.0006	1.5	0.61	1	y4 -COCH2 -NH3 [5|6][4-7]	C21H24N4O5

Meas. m/z	Calc. m/z	δ (Da)	δ (ppm)	Rel. Int. (%)	z	Annotation	Formula
413.1826	413.1819	0.0006	1.5	0.61	1	b3 [1|2][1-5][4|5][1-3]	C21H24N4O5
413.1826	413.1819	0.0006	1.5	0.61	1	y4 -NH3 [6|7][1-6][1|2][3-6]	C21H24N4O5
413.1826	413.1819	0.0006	1.5	0.61	1	y3 [1|2][1-5][2|3][3-5]	C21H24N4O5
414.1599	414.1594	0.0005	1.2	60.14	1	y3 -CO -COCH2 [3|4][5-7]	C20H23N5O3S
414.1599	414.1594	0.0005	1.2	60.14	1	b3 -CO -COCH2 [7|1][1-3]	C20H23N5O3S
414.1599	414.1594	0.0005	1.2	60.14	1	b3 -CO [2|3][1-6][4|5][1-3]	C20H23N5O3S
414.1599	414.1594	0.0005	1.2	60.14	1	y3 -CO [2|3][1-6][1|2][4-6]	C20H23N5O3S
414.1599	414.1594	0.0005	1.2	60.14	1	y3 -CO [6|7][1-4][2|3][2-4]	C20H23N5O3S
414.1599	414.1594	0.0005	1.2	60.14	1	b3 -CO [6|7][1-4][3|4][1-3]	C20H23N5O3S
418.1548	418.1544	0.0005	1.2	3.48	1	y4 -H2O [7|1][1-6][2|3][3-6]	C19H23N5O4S
418.1548	418.1544	0.0005	1.2	3.48	1	b4 -H2O [4|5][1-5][3|4][1-4]	C19H23N5O4S
418.1548	418.1544	0.0005	1.2	3.48	1	b4 -H2O [7|1][1-6][4|5][1-4]	C19H23N5O4S
418.1548	418.1544	0.0005	1.2	3.48	1	y4 -H2O [4|5][1-5][2|3][2-5]	C19H23N5O4S
421.1655	421.1653	0.0002	0.5	0.26	1	y5 -COCH2 -H2O [6|7][1-6][3|4][2-6]	C18H24N6O4S
421.1655	421.1653	0.0002	0.5	0.26	1	b5 -COCH2 -H2O [6|7][1-6][4|5][1-5]	C18H24N6O4S
421.1655	421.1653	0.0002	0.5	0.26	1	b5 -COCH2 -H2O [3|4][1-6][3|4][1-5]	C18H24N6O4S
421.1655	421.1653	0.0002	0.5	0.26	1	y5 -COCH2 -H2O [3|4][1-6][2|3][2-6]	C18H24N6O4S
423.1447	423.1445	0.0002	0.4	3.59	1	b5 -C10H9NO -COCH2 [5|6][1-6][2|3][1-5]	C17H22N6O5S
423.1447	423.1445	0.0002	0.4	3.59	1	y5 -C10H9NO [5|6][1-6][3|4][2-6]	C17H22N6O5S
423.1447	423.1445	0.0002	0.4	3.59	1	b5 -C10H9NO [5|6][1-6][4|5][1-5]	C17H22N6O5S
423.1447	423.1445	0.0002	0.4	3.59	1	b5 -C10H9NO [2|3][1-6][3|4][1-5]	C17H22N6O5S
423.1447	423.1445	0.0002	0.4	3.59	1	y5 -C10H9NO -COCH2 [7|1][1-6][4|5][2-6]	C17H22N6O5S
423.1447	423.1445	0.0002	0.4	3.59	1	y5 -C10H9NO -COCH2 [5|6][1-6][1|2][2-6]	C17H22N6O5S
423.1447	423.1445	0.0002	0.4	3.59	1	y5 -C10H9NO [2|3][1-6][2|3][2-6]	C17H22N6O5S
423.1447	423.1445	0.0002	0.4	3.59	1	b5 -C10H9NO -COCH2 [7|1][1-6][5|6][1-5]	C17H22N6O5S
424.1445	424.1438	0.0007	1.6	0.35	1	b3 -COCH2 -H2O [7|1][1-3]	C21H21N5O3S
424.1445	424.1438	0.0007	1.6	0.35	1	y3 -COCH2 -H2O [3|4][5-7]	C21H21N5O3S
424.1445	424.1438	0.0007	1.6	0.35	1	y3 -H2O [2|3][1-6][1|2][4-6]	C21H21N5O3S
424.1445	424.1438	0.0007	1.6	0.35	1	b3 -H2O [2|3][1-6][4|5][1-3]	C21H21N5O3S
424.1445	424.1438	0.0007	1.6	0.35	1	y3 -H2O [6|7][1-4][2|3][2-4]	C21H21N5O3S
424.1445	424.1438	0.0007	1.6	0.35	1	b3 -H2O [6|7][1-4][3|4][1-3]	C21H21N5O3S
425.1824	425.1819	0.0005	1.1	1.63	1	b4 -NH3 -CO [1|2][1-5][4|5][1-4]	C22H24N4O5
425.1824	425.1819	0.0005	1.1	1.63	1	y4 -NH3 [6|7][4-7]	C22H24N4O5
425.1824	425.1819	0.0005	1.1	1.63	1	b4 -NH3 [2|3][1-4]	C22H24N4O5
425.1824	425.1819	0.0005	1.1	1.63	1	b4 -COCH2 -NH3 [1|2][1-6][4|5][1-4]	C22H24N4O5
425.1824	425.1819	0.0005	1.1	1.63	1	y4 -COCH2 -NH3 [5|6][1-5][2|3][2-5]	C22H24N4O5
425.1824	425.1819	0.0005	1.1	1.63	1	y4 -NH3 -CO [1|2][1-5][3|4][2-5]	C22H24N4O5
425.1824	425.1819	0.0005	1.1	1.63	1	y4 -COCH2 -NH3 [1|2][1-6][2|3][3-6]	C22H24N4O5
425.1824	425.1819	0.0005	1.1	1.63	1	y4 -NH3 -CO [5|6][1-6][1|2][3-6]	C22H24N4O5
425.1824	425.1819	0.0005	1.1	1.63	1	b4 -COCH2 -NH3 [5|6][1-5][3|4][1-4]	C22H24N4O5
425.1824	425.1819	0.0005	1.1	1.63	1	b4 -NH3 -CO [5|6][1-6][3|4][1-4]	C22H24N4O5
427.1980	427.1976	0.0004	1.0	2.25	1	b4 -NH3 -CO [1|2][1-4]	C22H26N4O5
427.1980	427.1976	0.0004	1.0	2.25	1	y4 -NH3 -CO [5|6][4-7]	C22H26N4O5
435.1448	435.1445	0.0003	0.7	1.28	1	y5 -C10H9NO -H2O [4|5][3-7]	C18H22N6O5S
435.1448	435.1445	0.0003	0.7	1.28	1	b5 -C10H9NO -H2O [6|7][1-5]	C18H22N6O5S
435.1813	435.1809	0.0004	0.9	3.20	1	y5 -H2O -CO [3|4][1-6][2|3][2-6]	C19H26N6O4S
435.1813	435.1809	0.0004	0.9	3.20	1	b5 -H2O -CO [3|4][1-6][3|4][1-5]	C19H26N6O4S
435.1813	435.1809	0.0004	0.9	3.20	1	y5 -H2O -CO [6|7][1-6][3|4][2-6]	C19H26N6O4S
435.1813	435.1809	0.0004	0.9	3.20	1	b5 -H2O -CO [6|7][1-6][4|5][1-5]	C19H26N6O4S
437.1603	437.1602	0.0002	0.4	0.95	1	y5 -C10H9NO [2|3][1-6][4|5][2-6]	C18H24N6O5S
437.1603	437.1602	0.0002	0.4	0.95	1	y5 -C10H9NO -CO [5|6][1-6][1|2][2-6]	C18H24N6O5S

Meas. m/z	Calc. m/z	δ (Da)	δ (ppm)	Rel. Int. (%)	z	Annotation	Formula
437.1603	437.1602	0.0002	0.4	0.95	1	b5 -C10H9NO -CO [7|1][1-6][5|6][1-5]	C18H24N6O5S
437.1603	437.1602	0.0002	0.4	0.95	1	b5 -C10H9NO [2|3][1-6][5|6][1-5]	C18H24N6O5S
437.1603	437.1602	0.0002	0.4	0.95	1	b5 -COCH2 [3|4][1-6][2|3][1-5]	C18H24N6O5S
437.1603	437.1602	0.0002	0.4	0.95	1	y5 -COCH2 [5|6][1-6][4|5][2-6]	C18H24N6O5S
437.1603	437.1602	0.0002	0.4	0.95	1	y5 -COCH2 [3|4][1-6][1|2][2-6]	C18H24N6O5S
437.1603	437.1602	0.0002	0.4	0.95	1	b5 -C10H9NO -CO [5|6][1-6][2|3][1-5]	C18H24N6O5S
437.1603	437.1602	0.0002	0.4	0.95	1	b5 -COCH2 [5|6][1-6][5|6][1-5]	C18H24N6O5S
437.1603	437.1602	0.0002	0.4	0.95	1	y5 -C10H9NO -CO [7|1][1-6][4|5][2-6]	C18H24N6O5S
437.1603	437.1602	0.0002	0.4	0.95	1	y5 -C10H9NO -COCH2 [3|4][3-7]	C18H24N6O5S
437.1603	437.1602	0.0002	0.4	0.95	1	b5 -C10H9NO -COCH2 [5|6][1-5]	C18H24N6O5S
437.1603	437.1602	0.0002	0.4	0.95	1	y5 -C10H9NO [7|1][1-6][1|2][2-6]	C18H24N6O5S
437.1603	437.1602	0.0002	0.4	0.95	1	b5 -C10H9NO [7|1][1-6][2|3][1-5]	C18H24N6O5S
442.1549	442.1544	0.0005	1.2	41.13	1	b3 [6|7][1-4][3|4][1-3]	C21H23N5O4S
442.1549	442.1544	0.0005	1.2	41.13	1	y3 -COCH2 [3|4][5-7]	C21H23N5O4S
442.1549	442.1544	0.0005	1.2	41.13	1	y3 [2|3][1-6][1|2][4-6]	C21H23N5O4S
442.1549	442.1544	0.0005	1.2	41.13	1	b3 -COCH2 [7|1][1-3]	C21H23N5O4S
442.1549	442.1544	0.0005	1.2	41.13	1	b3 [2|3][1-6][4|5][1-3]	C21H23N5O4S
442.1549	442.1544	0.0005	1.2	41.13	1	y3 [6|7][1-4][2|3][2-4]	C21H23N5O4S
447.1451	447.1445	0.0005	1.2	0.58	1	y5 -C10H9NO -H2O [7|1][1-6][4|5][2-6]	C19H22N6O5S
447.1451	447.1445	0.0005	1.2	0.58	1	y5 -C10H9NO -H2O [5|6][1-6][1|2][2-6]	C19H22N6O5S
447.1451	447.1445	0.0005	1.2	0.58	1	b5 -C10H9NO -H2O [7|1][1-6][5|6][1-5]	C19H22N6O5S
447.1451	447.1445	0.0005	1.2	0.58	1	b5 -C10H9NO -H2O [5|6][1-6][2|3][1-5]	C19H22N6O5S
447.1814	447.1809	0.0005	1.0	0.21	1	y5 -H2O -CO [3|4][1-6][3|4][2-6]	C20H26N6O4S
447.1814	447.1809	0.0005	1.0	0.21	1	b5 -H2O -CO [3|4][1-6][4|5][1-5]	C20H26N6O4S
447.1814	447.1809	0.0005	1.0	0.21	1	b5 -COCH2 -H2O [4|5][1-5]	C20H26N6O4S
447.1814	447.1809	0.0005	1.0	0.21	1	b5 -H2O -CO [7|1][1-6][3|4][1-5]	C20H26N6O4S
447.1814	447.1809	0.0005	1.0	0.21	1	y5 -COCH2 -H2O [2|3][3-7]	C20H26N6O4S
447.1814	447.1809	0.0005	1.0	0.21	1	y5 -H2O -CO [7|1][1-6][2|3][2-6]	C20H26N6O4S
449.1596	449.1602	-0.0006	-1.2	0.13	1	b5 -C10H9NO -H2O [7|1][1-5]	C19H24N6O5S
449.1596	449.1602	-0.0006	-1.2	0.13	1	y5 -C10H9NO -H2O [5|6][3-7]	C19H24N6O5S
453.1555	453.1551	0.0005	1.0	2.05	1	y5 -C10H9NO [4|5][3-7]	C18H24N6O6S
453.1555	453.1551	0.0005	1.0	2.05	1	b5 -C10H9NO [6|7][1-5]	C18H24N6O6S
454.1550	454.1544	0.0006	1.4	0.26	1	b4 -NH3 -CO [6|7][1-5][3|4][1-4]	C22H23N5O4S
454.1550	454.1544	0.0006	1.4	0.26	1	y4 -NH3 -CO [2|3][1-6][2|3][3-6]	C22H23N5O4S
454.1550	454.1544	0.0006	1.4	0.26	1	b4 -NH3 -CO [2|3][1-6][4|5][1-4]	C22H23N5O4S
454.1550	454.1544	0.0006	1.4	0.26	1	y4 -NH3 -CO [6|7][1-5][2|3][2-5]	C22H23N5O4S
461.1608	461.1602	0.0006	1.3	0.47	1	y5 -C10H9NO -H2O [3|4][3-7]	C20H24N6O5S
461.1608	461.1602	0.0006	1.3	0.47	1	b5 -C10H9NO -H2O [5|6][1-5]	C20H24N6O5S
461.1608	461.1602	0.0006	1.3	0.47	1	y5 -H2O [3|4][1-6][1|2][2-6]	C20H24N6O5S
461.1608	461.1602	0.0006	1.3	0.47	1	b5 -H2O [3|4][1-6][2|3][1-5]	C20H24N6O5S
461.1608	461.1602	0.0006	1.3	0.47	1	y5 -H2O [5|6][1-6][4|5][2-6]	C20H24N6O5S
461.1608	461.1602	0.0006	1.3	0.47	1	b5 -H2O [5|6][1-6][5|6][1-5]	C20H24N6O5S
462.1448	462.1442	0.0006	1.4	1.03	1	y5 -C10H9NO -NH3 [3|4][3-7]	C20H23N5O6S
462.1448	462.1442	0.0006	1.4	1.03	1	b5 -C10H9NO -NH3 [5|6][1-5]	C20H23N5O6S
462.1448	462.1442	0.0006	1.4	1.03	1	y5 -NH3 [3|4][1-6][1|2][2-6]	C20H23N5O6S
462.1448	462.1442	0.0006	1.4	1.03	1	b5 -NH3 [3|4][1-6][2|3][1-5]	C20H23N5O6S
462.1448	462.1442	0.0006	1.4	1.03	1	y5 -NH3 [5|6][1-6][4|5][2-6]	C20H23N5O6S
462.1448	462.1442	0.0006	1.4	1.03	1	b5 -NH3 [5|6][1-6][5|6][1-5]	C20H23N5O6S
467.1387	467.1384	0.0003	0.7	0.39	1	b3 -NH3 [7|1][1-3]	C23H22N4O5S
467.1387	467.1384	0.0003	0.7	0.39	1	y3 -NH3 [3|4][5-7]	C23H22N4O5S
471.1803	471.1809	-0.0006	-1.2	0.14	1	b4 -CO -COCH2 [7|1][1-4]	C22H26N6O4S

Meas. m/z	Calc. m/z	δ (Da)	δ (ppm)	Rel. Int. (%)	z	Annotation	Formula
471.1803	471.1809	-0.0006	-1.2	0.14	1	y4 -CO [6|7][1-5][2|3][2-5]	C22H26N6O4S
471.1803	471.1809	-0.0006	-1.2	0.14	1	y4 -CO -COCH2 [4|5][4-7]	C22H26N6O4S
471.1803	471.1809	-0.0006	-1.2	0.14	1	y4 -CO [2|3][1-6][2|3][3-6]	C22H26N6O4S
471.1803	471.1809	-0.0006	-1.2	0.14	1	b4 -CO [2|3][1-6][4|5][1-4]	C22H26N6O4S
471.1803	471.1809	-0.0006	-1.2	0.14	1	b4 -CO [6|7][1-5][3|4][1-4]	C22H26N6O4S
479.1714	479.1707	0.0007	1.4	1.17	1	y5 [3|4][1-6][1|2][2-6]	C20H26N6O6S
479.1714	479.1707	0.0007	1.4	1.17	1	b5 [3|4][1-6][2|3][1-5]	C20H26N6O6S
479.1714	479.1707	0.0007	1.4	1.17	1	y5 [5|6][1-6][4|5][2-6]	C20H26N6O6S
479.1714	479.1707	0.0007	1.4	1.17	1	b5 [5|6][1-6][5|6][1-5]	C20H26N6O6S
479.1714	479.1707	0.0007	1.4	1.17	1	y5 -C10H9NO [3|4][3-7]	C20H26N6O6S
479.1714	479.1707	0.0007	1.4	1.17	1	b5 -C10H9NO [5|6][1-5]	C20H26N6O6S
479.1865	479.1860	0.0005	1.0	6.68	1	y4 -H2O -CO [2|3][1-6][1|2][3-6]	C24H26N6O3S
479.1865	479.1860	0.0005	1.0	6.68	1	b4 -H2O -CO [2|3][1-6][3|4][1-4]	C24H26N6O3S
479.1865	479.1860	0.0005	1.0	6.68	1	y4 -H2O -CO [5|6][1-5][3|4][2-5]	C24H26N6O3S
479.1865	479.1860	0.0005	1.0	6.68	1	b4 -H2O -CO [5|6][1-5][4|5][1-4]	C24H26N6O3S
480.1705	480.1700	0.0005	1.1	29.46	1	y4 -NH3 -CO [2|3][1-6][1|2][3-6]	C24H25N5O4S
480.1705	480.1700	0.0005	1.1	29.46	1	b4 -NH3 -CO [2|3][1-6][3|4][1-4]	C24H25N5O4S
480.1705	480.1700	0.0005	1.1	29.46	1	y4 -NH3 -CO [5|6][1-5][3|4][2-5]	C24H25N5O4S
480.1705	480.1700	0.0005	1.1	29.46	1	b4 -NH3 -CO [5|6][1-5][4|5][1-4]	C24H25N5O4S
484.1653	484.1649	0.0004	0.8	0.77	1	y3 [3|4][5-7]	C23H25N5O5S
484.1653	484.1649	0.0004	0.8	0.77	1	b3 [7|1][1-3]	C23H25N5O5S
485.1968	485.1966	0.0003	0.6	0.14	1	b4 -CO [7|1][1-5][2|3][1-4]	C23H28N6O4S
485.1968	485.1966	0.0003	0.6	0.14	1	y4 -CO [2|3][1-6][3|4][3-6]	C23H28N6O4S
485.1968	485.1966	0.0003	0.6	0.14	1	b4 -CO [2|3][1-6][5|6][1-4]	C23H28N6O4S
485.1968	485.1966	0.0003	0.6	0.14	1	y4 -CO [7|1][1-5][1|2][2-5]	C23H28N6O4S
485.1968	485.1966	0.0003	0.6	0.14	1	b4 -CO -COCH2 [6|7][1-4]	C23H28N6O4S
485.1968	485.1966	0.0003	0.6	0.14	1	y4 -CO -COCH2 [3|4][4-7]	C23H28N6O4S
490.1873	490.1867	0.0005	1.1	0.28	1	y6 -C10H9NO -H2O [1|2][2-7]	C21H27N7O5S
490.1873	490.1867	0.0005	1.1	0.28	1	b6 -C10H9NO -H2O [2|3][1-6]	C21H27N7O5S
491.1711	491.1707	0.0004	0.7	0.76	1	y6 -C10H9NO -NH3 [1|2][2-7]	C21H26N6O6S
491.1711	491.1707	0.0004	0.7	0.76	1	b6 -C10H9NO -NH3 [2|3][1-6]	C21H26N6O6S
496.1660	496.1649	0.0011	2.3	0.14	1	b4 -NH3 -CO [7|1][1-4]	C24H25N5O5S
496.1660	496.1649	0.0011	2.3	0.14	1	y4 -COCH2 -NH3 [3|4][4-7]	C24H25N5O5S
496.1660	496.1649	0.0011	2.3	0.14	1	b4 -NH3 [7|1][1-5][2|3][1-4]	C24H25N5O5S
496.1660	496.1649	0.0011	2.3	0.14	1	y4 -NH3 -CO [4|5][4-7]	C24H25N5O5S
496.1660	496.1649	0.0011	2.3	0.14	1	b4 -COCH2 -NH3 [6|7][1-4]	C24H25N5O5S
496.1660	496.1649	0.0011	2.3	0.14	1	y4 -NH3 [7|1][1-5][1|2][2-5]	C24H25N5O5S
496.1660	496.1649	0.0011	2.3	0.14	1	y4 -NH3 [2|3][1-6][3|4][3-6]	C24H25N5O5S
496.1660	496.1649	0.0011	2.3	0.14	1	b4 -NH3 [2|3][1-6][5|6][1-4]	C24H25N5O5S
496.2204	496.2191	0.0013	2.7	0.28	1	y5 -NH3 [7|1][3-7]	C25H29N5O6
496.2204	496.2191	0.0013	2.7	0.28	1	b5 -COCH2 -NH3 [1|2][1-5]	C25H29N5O6
496.2204	496.2191	0.0013	2.7	0.28	1	b5 -NH3 [2|3][1-5]	C25H29N5O6
496.2204	496.2191	0.0013	2.7	0.28	1	b5 -NH3 -CO [1|2][1-6][4|5][1-5]	C25H29N5O6
496.2204	496.2191	0.0013	2.7	0.28	1	y5 -COCH2 -NH3 [6|7][3-7]	C25H29N5O6
496.2204	496.2191	0.0013	2.7	0.28	1	y5 -NH3 -CO [1|2][1-6][3|4][2-6]	C25H29N5O6
496.2204	496.2191	0.0013	2.7	0.28	1	y5 -NH3 -CO [5|6][1-6][2|3][2-6]	C25H29N5O6
496.2204	496.2191	0.0013	2.7	0.28	1	b5 -NH3 -CO [5|6][1-6][3|4][1-5]	C25H29N5O6
497.1971	497.1966	0.0006	1.2	12.27	1	b4 -CO -COCH2 [7|1][1-6][5|6][1-4]	C24H28N6O4S
497.1971	497.1966	0.0006	1.2	12.27	1	y4 -CO -COCH2 [7|1][1-6][3|4][3-6]	C24H28N6O4S
497.1971	497.1966	0.0006	1.2	12.27	1	y4 -CO [2|3][1-6][1|2][3-6]	C24H28N6O4S
497.1971	497.1966	0.0006	1.2	12.27	1	b4 -CO [2|3][1-6][3|4][1-4]	C24H28N6O4S

Meas. m/z	Calc. m/z	δ (Da)	δ (ppm)	Rel. Int. (%)	z	Annotation	Formula
497.1971	497.1966	0.0006	1.2	12.27	1	y4 -CO -COCH2 [5|6][1-5][1|2][2-5]	C24H28N6O4S
497.1971	497.1966	0.0006	1.2	12.27	1	b4 -CO -COCH2 [5|6][1-5][2|3][1-4]	C24H28N6O4S
497.1971	497.1966	0.0006	1.2	12.27	1	y4 -CO [5|6][1-5][3|4][2-5]	C24H28N6O4S
497.1971	497.1966	0.0006	1.2	12.27	1	b4 -CO [5|6][1-5][4|5][1-4]	C24H28N6O4S
506.1499	506.1493	0.0006	1.2	0.36	1	b4 -H2O -NH3 [7|1][1-4]	C25H23N5O5S
506.1499	506.1493	0.0006	1.2	0.36	1	y4 -H2O -NH3 [4|5][4-7]	C25H23N5O5S
507.1815	507.1809	0.0006	1.2	4.67	1	b4 -COCH2 -H2O [7|1][1-6][5|6][1-4]	C25H26N6O4S
507.1815	507.1809	0.0006	1.2	4.67	1	y4 -COCH2 -H2O [7|1][1-6][3|4][3-6]	C25H26N6O4S
507.1815	507.1809	0.0006	1.2	4.67	1	y4 -H2O [2|3][1-6][1|2][3-6]	C25H26N6O4S
507.1815	507.1809	0.0006	1.2	4.67	1	y4 -H2O [5|6][1-5][3|4][2-5]	C25H26N6O4S
507.1815	507.1809	0.0006	1.2	4.67	1	b4 -H2O [2|3][1-6][3|4][1-4]	C25H26N6O4S
507.1815	507.1809	0.0006	1.2	4.67	1	y4 -COCH2 -H2O [5|6][1-5][1|2][2-5]	C25H26N6O4S
507.1815	507.1809	0.0006	1.2	4.67	1	b4 -COCH2 -H2O [5|6][1-5][2|3][1-4]	C25H26N6O4S
507.1815	507.1809	0.0006	1.2	4.67	1	b4 -H2O [5|6][1-5][4|5][1-4]	C25H26N6O4S
508.1655	508.1649	0.0006	1.1	15.97	1	y4 -COCH2 -NH3 [7|1][1-6][3|4][3-6]	C25H25N5O5S
508.1655	508.1649	0.0006	1.1	15.97	1	b4 -COCH2 -NH3 [7|1][1-6][5|6][1-4]	C25H25N5O5S
508.1655	508.1649	0.0006	1.1	15.97	1	b4 -COCH2 -NH3 [5|6][1-5][2|3][1-4]	C25H25N5O5S
508.1655	508.1649	0.0006	1.1	15.97	1	y4 -NH3 [2|3][1-6][1|2][3-6]	C25H25N5O5S
508.1655	508.1649	0.0006	1.1	15.97	1	b4 -NH3 [2|3][1-6][3|4][1-4]	C25H25N5O5S
508.1655	508.1649	0.0006	1.1	15.97	1	y4 -COCH2 -NH3 [5|6][1-5][1|2][2-5]	C25H25N5O5S
508.1655	508.1649	0.0006	1.1	15.97	1	b4 -NH3 [5|6][1-5][4|5][1-4]	C25H25N5O5S
508.1655	508.1649	0.0006	1.1	15.97	1	y4 -NH3 [5|6][1-5][3|4][2-5]	C25H25N5O5S
509.1969	509.1966	0.0003	0.6	0.81	1	y4 -H2O -CO [3|4][4-7]	C25H28N6O4S
509.1969	509.1966	0.0003	0.6	0.81	1	b4 -H2O -CO [6|7][1-4]	C25H28N6O4S
509.1969	509.1966	0.0003	0.6	0.81	1	y4 -COCH2 -H2O [7|1][1-5][3|4][2-5]	C25H28N6O4S
509.1969	509.1966	0.0003	0.6	0.81	1	y4 -COCH2 -H2O [4|5][1-6][1|2][3-6]	C25H28N6O4S
509.1969	509.1966	0.0003	0.6	0.81	1	b4 -COCH2 -H2O [7|1][1-5][4|5][1-4]	C25H28N6O4S
509.1969	509.1966	0.0003	0.6	0.81	1	b4 -COCH2 -H2O [4|5][1-6][3|4][1-4]	C25H28N6O4S
510.1814	510.1806	0.0009	1.7	0.32	1	b4 -COCH2 -NH3 [4|5][1-6][3|4][1-4]	C25H27N5O5S
510.1814	510.1806	0.0009	1.7	0.32	1	y4 -COCH2 -NH3 [4|5][1-6][1|2][3-6]	C25H27N5O5S
510.1814	510.1806	0.0009	1.7	0.32	1	y4 -NH3 -CO [3|4][4-7]	C25H27N5O5S
510.1814	510.1806	0.0009	1.7	0.32	1	b4 -NH3 -CO [6|7][1-4]	C25H27N5O5S
510.1814	510.1806	0.0009	1.7	0.32	1	b4 -COCH2 -NH3 [7|1][1-5][4|5][1-4]	C25H27N5O5S
510.1814	510.1806	0.0009	1.7	0.32	1	y4 -COCH2 -NH3 [7|1][1-5][3|4][2-5]	C25H27N5O5S
510.2352	510.2347	0.0005	1.0	39.11	1	y5 -COCH2 -NH3 [1|2][1-6][2|3][2-6]	C26H31N5O6
510.2352	510.2347	0.0005	1.0	39.11	1	b5 -NH3 -CO [1|2][1-5]	C26H31N5O6
510.2352	510.2347	0.0005	1.0	39.11	1	y5 -NH3 -CO [6|7][3-7]	C26H31N5O6
510.2352	510.2347	0.0005	1.0	39.11	1	y5 -COCH2 -NH3 [4|5][1-6][3|4][2-6]	C26H31N5O6
510.2352	510.2347	0.0005	1.0	39.11	1	b5 -COCH2 -NH3 [1|2][1-6][3|4][1-5]	C26H31N5O6
510.2352	510.2347	0.0005	1.0	39.11	1	b5 -COCH2 -NH3 [4|5][1-6][4|5][1-5]	C26H31N5O6
513.1923	513.1915	0.0009	1.7	1.47	1	y4 [7|1][1-5][1|2][2-5]	C24H28N6O5S
513.1923	513.1915	0.0009	1.7	1.47	1	y4 -COCH2 [3|4][4-7]	C24H28N6O5S
513.1923	513.1915	0.0009	1.7	1.47	1	y4 -CO [4|5][4-7]	C24H28N6O5S
513.1923	513.1915	0.0009	1.7	1.47	1	b4 [7|1][1-5][2|3][1-4]	C24H28N6O5S
513.1923	513.1915	0.0009	1.7	1.47	1	b4 -COCH2 [6|7][1-4]	C24H28N6O5S
513.1923	513.1915	0.0009	1.7	1.47	1	b4 [2|3][1-6][5|6][1-4]	C24H28N6O5S
513.1923	513.1915	0.0009	1.7	1.47	1	y4 [2|3][1-6][3|4][3-6]	C24H28N6O5S
513.1923	513.1915	0.0009	1.7	1.47	1	b4 -CO [7|1][1-4]	C24H28N6O5S
518.1821	518.1816	0.0004	0.8	4.02	1	y6 -C10H9NO -H2O [4|5][2-7]	C22H27N7O6S
518.1821	518.1816	0.0004	0.8	4.02	1	b6 -C10H9NO -H2O [5|6][1-6]	C22H27N7O6S
519.1663	519.1656	0.0006	1.2	1.02	1	y6 -C10H9NO -NH3 [4|5][2-7]	C22H26N6O7S

Meas. m/z	Calc. m/z	δ (Da)	δ (ppm)	Rel. Int. (%)	z	Annotation	Formula
519.1663	519.1656	0.0006	1.2	1.02	1	b6 -C10H9NO -NH3 [5|6][1-6]	C22H26N6O7S
519.2025	519.2020	0.0005	0.9	1.42	1	y6 -NH3 -CO [2|3][2-7]	C23H30N6O6S
519.2025	519.2020	0.0005	0.9	1.42	1	b6 -NH3 -CO [3|4][1-6]	C23H30N6O6S
520.2206	520.2191	0.0016	3.0	0.17	1	b5 -H2O -NH3 [1|2][1-5]	C27H29N5O6
520.2206	520.2191	0.0016	3.0	0.17	1	y5 -H2O -NH3 [6|7][3-7]	C27H29N5O6
523.2129	523.2122	0.0007	1.3	0.78	1	y4 -H2O -CO [7|1][1-5][3|4][2-5]	C26H30N6O4S
523.2129	523.2122	0.0007	1.3	0.78	1	b4 -H2O -CO [7|1][1-5][4|5][1-4]	C26H30N6O4S
523.2129	523.2122	0.0007	1.3	0.78	1	y4 -H2O -CO [4|5][1-6][1|2][3-6]	C26H30N6O4S
523.2129	523.2122	0.0007	1.3	0.78	1	b4 -H2O -CO [4|5][1-6][3|4][1-4]	C26H30N6O4S
524.1603	524.1598	0.0005	1.0	1.01	1	b4 -NH3 [7|1][1-4]	C25H25N5O6S
524.1603	524.1598	0.0005	1.0	1.01	1	y4 -NH3 [4|5][4-7]	C25H25N5O6S
524.1967	524.1962	0.0005	1.0	2.06	1	y4 -NH3 -CO [7|1][1-5][3|4][2-5]	C26H29N5O5S
524.1967	524.1962	0.0005	1.0	2.06	1	b4 -NH3 -CO [7|1][1-5][4|5][1-4]	C26H29N5O5S
524.1967	524.1962	0.0005	1.0	2.06	1	y4 -NH3 -CO [4|5][1-6][1|2][3-6]	C26H29N5O5S
524.1967	524.1962	0.0005	1.0	2.06	1	b4 -NH3 -CO [4|5][1-6][3|4][1-4]	C26H29N5O5S
525.1921	525.1915	0.0006	1.2	4.79	1	b4 -COCH2 [5|6][1-5][2|3][1-4]	C25H28N6O5S
525.1921	525.1915	0.0006	1.2	4.79	1	y4 [2|3][1-6][1|2][3-6]	C25H28N6O5S
525.1921	525.1915	0.0006	1.2	4.79	1	b4 [2|3][1-6][3|4][1-4]	C25H28N6O5S
525.1921	525.1915	0.0006	1.2	4.79	1	y4 [5|6][1-5][3|4][2-5]	C25H28N6O5S
525.1921	525.1915	0.0006	1.2	4.79	1	b4 [5|6][1-5][4|5][1-4]	C25H28N6O5S
525.1921	525.1915	0.0006	1.2	4.79	1	y4 -COCH2 [7|1][1-6][3|4][3-6]	C25H28N6O5S
525.1921	525.1915	0.0006	1.2	4.79	1	b4 -COCH2 [7|1][1-6][5|6][1-4]	C25H28N6O5S
525.1921	525.1915	0.0006	1.2	4.79	1	y4 -COCH2 [5|6][1-5][1|2][2-5]	C25H28N6O5S
527.2077	527.2071	0.0006	1.1	0.49	1	y4 -CO [3|4][4-7]	C25H30N6O5S
527.2077	527.2071	0.0006	1.1	0.49	1	b4 -CO [6|7][1-4]	C25H30N6O5S
527.2077	527.2071	0.0006	1.1	0.49	1	y4 -COCH2 [7|1][1-5][3|4][2-5]	C25H30N6O5S
527.2077	527.2071	0.0006	1.1	0.49	1	b4 -COCH2 [7|1][1-5][4|5][1-4]	C25H30N6O5S
527.2077	527.2071	0.0006	1.1	0.49	1	y4 -COCH2 [4|5][1-6][1|2][3-6]	C25H30N6O5S
527.2077	527.2071	0.0006	1.1	0.49	1	b4 -COCH2 [4|5][1-6][3|4][1-4]	C25H30N6O5S
532.1981	532.1973	0.0008	1.5	0.14	1	b6 -C10H9NO -H2O [7|1][1-6]	C23H29N7O6S
532.1981	532.1973	0.0008	1.5	0.14	1	y6 -C10H9NO -H2O [6|7][2-7]	C23H29N7O6S
536.1926	536.1922	0.0004	0.7	2.73	1	y6 -C10H9NO [4|5][2-7]	C22H29N7O7S
536.1926	536.1922	0.0004	0.7	2.73	1	b6 -C10H9NO [5|6][1-6]	C22H29N7O7S
538.2252	538.2231	0.0021	3.8	1.15	1	y5 -H2O -CO [2|3][1-6][3|4][2-6]	C26H31N7O4S
538.2252	538.2231	0.0021	3.8	1.15	1	b5 -H2O -CO [2|3][1-6][4|5][1-5]	C26H31N7O4S
538.2252	538.2231	0.0021	3.8	1.15	1	y5 -H2O -CO [6|7][1-6][2|3][2-6]	C26H31N7O4S
538.2252	538.2231	0.0021	3.8	1.15	1	b5 -H2O -CO [6|7][1-6][3|4][1-5]	C26H31N7O4S
541.1871	541.1864	0.0008	1.4	2.38	1	b4 [7|1][1-4]	C25H28N6O6S
541.1871	541.1864	0.0008	1.4	2.38	1	y4 [4|5][4-7]	C25H28N6O6S
546.2136	546.2129	0.0006	1.2	0.59	1	y6 -H2O [2|3][2-7]	C24H31N7O6S
546.2136	546.2129	0.0006	1.2	0.59	1	b6 -H2O [3|4][1-6]	C24H31N7O6S
546.2136	546.2129	0.0006	1.2	0.59	1	y6 -C10H9NO -H2O [3|4][2-7]	C24H31N7O6S
546.2136	546.2129	0.0006	1.2	0.59	1	b6 -C10H9NO -H2O [4|5][1-6]	C24H31N7O6S
550.2225	550.2231	-0.0006	-1.0	0.14	1	y5 -H2O -CO [7|1][1-6][1|2][2-6]	C27H31N7O4S
550.2225	550.2231	-0.0006	-1.0	0.14	1	b5 -H2O -CO [7|1][1-6][2|3][1-5]	C27H31N7O4S
550.2225	550.2231	-0.0006	-1.0	0.14	1	y5 -H2O -CO [2|3][1-6][4|5][2-6]	C27H31N7O4S
550.2225	550.2231	-0.0006	-1.0	0.14	1	b5 -H2O -CO [2|3][1-6][5|6][1-5]	C27H31N7O4S
553.1874	553.1864	0.0011	1.9	0.34	1	b5 -COCH2 -NH3 [6|7][1-5]	C26H28N6O6S
553.1874	553.1864	0.0011	1.9	0.34	1	y5 -COCH2 -NH3 [4|5][3-7]	C26H28N6O6S
554.2198	554.2180	0.0018	3.2	0.16	1	y5 -CO -COCH2 [7|1][1-6][4|5][2-6]	C26H31N7O5S
554.2198	554.2180	0.0018	3.2	0.16	1	b5 -CO -COCH2 [7|1][1-6][5|6][1-5]	C26H31N7O5S

Meas. m/z	Calc. m/z	δ (Da)	δ (ppm)	Rel. Int. (%)	z	Annotation	Formula
554.2198	554.2180	0.0018	3.2	0.16	1	y5 -CO [2|3][1-6][2|3][2-6]	C26H31N7O5S
554.2198	554.2180	0.0018	3.2	0.16	1	b5 -CO [2|3][1-6][3|4][1-5]	C26H31N7O5S
554.2198	554.2180	0.0018	3.2	0.16	1	y5 -CO -COCH2 [5|6][1-6][1|2][2-6]	C26H31N7O5S
554.2198	554.2180	0.0018	3.2	0.16	1	b5 -CO -COCH2 [5|6][1-6][2|3][1-5]	C26H31N7O5S
554.2198	554.2180	0.0018	3.2	0.16	1	y5 -CO [5|6][1-6][3|4][2-6]	C26H31N7O5S
554.2198	554.2180	0.0018	3.2	0.16	1	b5 -CO [5|6][1-6][4|5][1-5]	C26H31N7O5S
555.2026	555.2020	0.0005	1.0	17.30	1	y4 [3|4][4-7]	C26H30N6O6S
555.2026	555.2020	0.0005	1.0	17.30	1	b4 [6|7][1-4]	C26H30N6O6S
565.2234	565.2228	0.0007	1.2	7.60	1	y5 -NH3 -CO [2|3][1-6][1|2][2-6]	C28H32N6O5S
565.2234	565.2228	0.0007	1.2	7.60	1	b5 -NH3 -CO [2|3][1-6][2|3][1-5]	C28H32N6O5S
565.2234	565.2228	0.0007	1.2	7.60	1	y5 -NH3 -CO [4|5][1-6][4|5][2-6]	C28H32N6O5S
565.2234	565.2228	0.0007	1.2	7.60	1	b5 -NH3 -CO [4|5][1-6][5|6][1-5]	C28H32N6O5S
566.2186	566.2180	0.0006	1.0	1.68	1	b5 -COCH2 -H2O [7|1][1-5]	C27H31N7O5S
566.2186	566.2180	0.0006	1.0	1.68	1	y5 -H2O -CO [4|5][3-7]	C27H31N7O5S
566.2186	566.2180	0.0006	1.0	1.68	1	y5 -COCH2 -H2O [5|6][3-7]	C27H31N7O5S
566.2186	566.2180	0.0006	1.0	1.68	1	b5 -H2O -CO [6|7][1-5]	C27H31N7O5S
566.2186	566.2180	0.0006	1.0	1.68	1	y5 -H2O [2|3][1-6][3|4][2-6]	C27H31N7O5S
566.2186	566.2180	0.0006	1.0	1.68	1	b5 -H2O [2|3][1-6][4|5][1-5]	C27H31N7O5S
566.2186	566.2180	0.0006	1.0	1.68	1	y5 -H2O [6|7][1-6][2|3][2-6]	C27H31N7O5S
566.2186	566.2180	0.0006	1.0	1.68	1	b5 -H2O [6|7][1-6][3|4][1-5]	C27H31N7O5S
567.2023	567.2020	0.0003	0.5	0.25	1	y4 [7|1][1-6][3|4][3-6]	C27H30N6O6S
567.2023	567.2020	0.0003	0.5	0.25	1	b4 [7|1][1-6][5|6][1-4]	C27H30N6O6S
567.2023	567.2020	0.0003	0.5	0.25	1	y4 [5|6][1-5][1|2][2-5]	C27H30N6O6S
567.2023	567.2020	0.0003	0.5	0.25	1	b4 [5|6][1-5][2|3][1-4]	C27H30N6O6S
567.2023	567.2020	0.0003	0.5	0.25	1	b5 -COCH2 -NH3 [7|1][1-5]	C27H30N6O6S
567.2023	567.2020	0.0003	0.5	0.25	1	y5 -NH3 -CO [4|5][3-7]	C27H30N6O6S
567.2023	567.2020	0.0003	0.5	0.25	1	y5 -COCH2 -NH3 [5|6][3-7]	C27H30N6O6S
567.2023	567.2020	0.0003	0.5	0.25	1	b5 -NH3 -CO [6|7][1-5]	C27H30N6O6S
567.2023	567.2020	0.0003	0.5	0.25	1	y5 -NH3 [2|3][1-6][3|4][2-6]	C27H30N6O6S
567.2023	567.2020	0.0003	0.5	0.25	1	b5 -NH3 [2|3][1-6][4|5][1-5]	C27H30N6O6S
567.2023	567.2020	0.0003	0.5	0.25	1	y5 -NH3 [6|7][1-6][2|3][2-6]	C27H30N6O6S
567.2023	567.2020	0.0003	0.5	0.25	1	b5 -NH3 [6|7][1-6][3|4][1-5]	C27H30N6O6S
568.2343	568.2337	0.0006	1.1	2.00	1	y5 -CO -COCH2 [3|4][3-7]	C27H33N7O5S
568.2343	568.2337	0.0006	1.1	2.00	1	b5 -CO -COCH2 [5|6][1-5]	C27H33N7O5S
568.2343	568.2337	0.0006	1.1	2.00	1	y5 -CO [7|1][1-6][1|2][2-6]	C27H33N7O5S
568.2343	568.2337	0.0006	1.1	2.00	1	b5 -CO [7|1][1-6][2|3][1-5]	C27H33N7O5S
568.2343	568.2337	0.0006	1.1	2.00	1	y5 -CO [2|3][1-6][4|5][2-6]	C27H33N7O5S
568.2343	568.2337	0.0006	1.1	2.00	1	b5 -CO [2|3][1-6][5|6][1-5]	C27H33N7O5S
578.2199	578.2180	0.0019	3.3	0.29	1	y5 -COCH2 -H2O [3|4][3-7]	C28H31N7O5S
578.2199	578.2180	0.0019	3.3	0.29	1	b5 -COCH2 -H2O [5|6][1-5]	C28H31N7O5S
578.2199	578.2180	0.0019	3.3	0.29	1	y5 -H2O [7|1][1-6][1|2][2-6]	C28H31N7O5S
578.2199	578.2180	0.0019	3.3	0.29	1	b5 -H2O [7|1][1-6][2|3][1-5]	C28H31N7O5S
578.2199	578.2180	0.0019	3.3	0.29	1	y5 -H2O -CO [7|1][1-6][4|5][2-6]	C28H31N7O5S
578.2199	578.2180	0.0019	3.3	0.29	1	b5 -H2O -CO [7|1][1-6][5|6][1-5]	C28H31N7O5S
578.2199	578.2180	0.0019	3.3	0.29	1	y5 -H2O [2|3][1-6][4|5][2-6]	C28H31N7O5S
578.2199	578.2180	0.0019	3.3	0.29	1	b5 -H2O [2|3][1-6][5|6][1-5]	C28H31N7O5S
578.2199	578.2180	0.0019	3.3	0.29	1	y5 -H2O -CO [5|6][1-6][1|2][2-6]	C28H31N7O5S
578.2199	578.2180	0.0019	3.3	0.29	1	b5 -H2O -CO [5|6][1-6][2|3][1-5]	C28H31N7O5S
580.2346	580.2337	0.0010	1.6	0.79	1	b5 -H2O -CO [7|1][1-5]	C28H33N7O5S
580.2346	580.2337	0.0010	1.6	0.79	1	y5 -H2O -CO [5|6][3-7]	C28H33N7O5S
580.2346	580.2337	0.0010	1.6	0.79	1	y5 -COCH2 -H2O [4|5][1-6][1|2][2-6]	C28H33N7O5S

Meas. m/z	Calc. m/z	δ (Da)	δ (ppm)	Rel. Int. (%)	z	Annotation	Formula
580.2346	580.2337	0.0010	1.6	0.79	1	b5 -COCH2 -H2O [4|5][1-6][2|3][1-5]	C28H33N7O5S
580.2346	580.2337	0.0010	1.6	0.79	1	y5 -COCH2 -H2O [6|7][1-6][4|5][2-6]	C28H33N7O5S
580.2346	580.2337	0.0010	1.6	0.79	1	b5 -COCH2 -H2O [6|7][1-6][5|6][1-5]	C28H33N7O5S
581.2186	581.2177	0.0009	1.5	1.43	1	b5 -NH3 -CO [7|1][1-5]	C28H32N6O6S
581.2186	581.2177	0.0009	1.5	1.43	1	y5 -NH3 -CO [5|6][3-7]	C28H32N6O6S
581.2186	581.2177	0.0009	1.5	1.43	1	y5 -COCH2 -NH3 [4|5][1-6][1|2][2-6]	C28H32N6O6S
581.2186	581.2177	0.0009	1.5	1.43	1	b5 -COCH2 -NH3 [4|5][1-6][2|3][1-5]	C28H32N6O6S
581.2186	581.2177	0.0009	1.5	1.43	1	y5 -COCH2 -NH3 [6|7][1-6][4|5][2-6]	C28H32N6O6S
581.2186	581.2177	0.0009	1.5	1.43	1	b5 -COCH2 -NH3 [6|7][1-6][5|6][1-5]	C28H32N6O6S
581.2725	581.2718	0.0006	1.1	3.87	1	y6 -NH3 -CO [7|1][2-7]	C29H36N6O7
581.2725	581.2718	0.0006	1.1	3.87	1	b6 -NH3 -CO [1|2][1-6]	C29H36N6O7
582.2503	582.2493	0.0010	1.7	0.94	1	y5 -CO -COCH2 [7|1][1-6][3|4][2-6]	C28H35N7O5S
582.2503	582.2493	0.0010	1.7	0.94	1	b5 -CO -COCH2 [7|1][1-6][4|5][1-5]	C28H35N7O5S
582.2503	582.2493	0.0010	1.7	0.94	1	y5 -CO [2|3][1-6][1|2][2-6]	C28H35N7O5S
582.2503	582.2493	0.0010	1.7	0.94	1	b5 -CO [2|3][1-6][2|3][1-5]	C28H35N7O5S
582.2503	582.2493	0.0010	1.7	0.94	1	y5 -CO -COCH2 [4|5][1-6][2|3][2-6]	C28H35N7O5S
582.2503	582.2493	0.0010	1.7	0.94	1	b5 -CO -COCH2 [4|5][1-6][3|4][1-5]	C28H35N7O5S
582.2503	582.2493	0.0010	1.7	0.94	1	y5 -CO [4|5][1-6][4|5][2-6]	C28H35N7O5S
582.2503	582.2493	0.0010	1.7	0.94	1	b5 -CO [4|5][1-6][5|6][1-5]	C28H35N7O5S
584.2292	584.2286	0.0007	1.1	9.45	1	y5 [6|7][1-6][2|3][2-6]	C27H33N7O6S
584.2292	584.2286	0.0007	1.1	9.45	1	y5 -CO [4|5][3-7]	C27H33N7O6S
584.2292	584.2286	0.0007	1.1	9.45	1	b5 [6|7][1-6][3|4][1-5]	C27H33N7O6S
584.2292	584.2286	0.0007	1.1	9.45	1	b5 -COCH2 [7|1][1-5]	C27H33N7O6S
584.2292	584.2286	0.0007	1.1	9.45	1	y5 -COCH2 [5|6][3-7]	C27H33N7O6S
584.2292	584.2286	0.0007	1.1	9.45	1	b5 -CO [6|7][1-5]	C27H33N7O6S
584.2292	584.2286	0.0007	1.1	9.45	1	y5 [2|3][1-6][3|4][2-6]	C27H33N7O6S
584.2292	584.2286	0.0007	1.1	9.45	1	b5 [2|3][1-6][4|5][1-5]	C27H33N7O6S
592.2352	592.2337	0.0015	2.5	0.30	1	y5 -H2O [4|5][1-6][4|5][2-6]	C29H33N7O5S
592.2352	592.2337	0.0015	2.5	0.30	1	y5 -H2O -CO [3|4][3-7]	C29H33N7O5S
592.2352	592.2337	0.0015	2.5	0.30	1	b5 -COCH2 -H2O [7|1][1-6][4|5][1-5]	C29H33N7O5S
592.2352	592.2337	0.0015	2.5	0.30	1	b5 -H2O -CO [5|6][1-5]	C29H33N7O5S
592.2352	592.2337	0.0015	2.5	0.30	1	y5 -COCH2 -H2O [7|1][1-6][3|4][2-6]	C29H33N7O5S
592.2352	592.2337	0.0015	2.5	0.30	1	y5 -COCH2 -H2O [4|5][1-6][2|3][2-6]	C29H33N7O5S
592.2352	592.2337	0.0015	2.5	0.30	1	y5 -H2O [2|3][1-6][1|2][2-6]	C29H33N7O5S
592.2352	592.2337	0.0015	2.5	0.30	1	b5 -H2O [2|3][1-6][2|3][1-5]	C29H33N7O5S
592.2352	592.2337	0.0015	2.5	0.30	1	b5 -COCH2 -H2O [4|5][1-6][3|4][1-5]	C29H33N7O5S
592.2352	592.2337	0.0015	2.5	0.30	1	b5 -H2O [4|5][1-6][5|6][1-5]	C29H33N7O5S
593.2184	593.2177	0.0008	1.3	0.28	1	b5 -NH3 [2|3][1-6][2|3][1-5]	C29H32N6O6S
593.2184	593.2177	0.0008	1.3	0.28	1	b5 -COCH2 -NH3 [4|5][1-6][3|4][1-5]	C29H32N6O6S
593.2184	593.2177	0.0008	1.3	0.28	1	y5 -NH3 [4|5][1-6][4|5][2-6]	C29H32N6O6S
593.2184	593.2177	0.0008	1.3	0.28	1	b5 -NH3 -CO [5|6][1-5]	C29H32N6O6S
593.2184	593.2177	0.0008	1.3	0.28	1	y5 -NH3 -CO [3|4][3-7]	C29H32N6O6S
593.2184	593.2177	0.0008	1.3	0.28	1	b5 -COCH2 -NH3 [7|1][1-6][4|5][1-5]	C29H32N6O6S
593.2184	593.2177	0.0008	1.3	0.28	1	y5 -COCH2 -NH3 [7|1][1-6][3|4][2-6]	C29H32N6O6S
593.2184	593.2177	0.0008	1.3	0.28	1	y5 -COCH2 -NH3 [4|5][1-6][2|3][2-6]	C29H32N6O6S
593.2184	593.2177	0.0008	1.3	0.28	1	y5 -NH3 [2|3][1-6][1|2][2-6]	C29H32N6O6S
593.2184	593.2177	0.0008	1.3	0.28	1	b5 -NH3 [4|5][1-6][5|6][1-5]	C29H32N6O6S
593.2505	593.2500	0.0004	0.8	0.51	1	M -C10H9NO -CO [7|1][1-7]	C25H36N8O7S
594.2139	594.2129	0.0010	1.7	3.35	1	y5 -H2O [4|5][3-7]	C28H31N7O6S
594.2139	594.2129	0.0010	1.7	3.35	1	b5 -H2O [6|7][1-5]	C28H31N7O6S
594.2501	594.2493	0.0008	1.4	21.42	1	y5 -H2O -CO [4|5][1-6][1|2][2-6]	C29H35N7O5S

Meas. m/z	Calc. m/z	δ (Da)	δ (ppm)	Rel. Int. (%)	z	Annotation	Formula
594.2501	594.2493	0.0008	1.4	21.42	1	b5 -H2O -CO [4|5][1-6][2|3][1-5]	C29H35N7O5S
594.2501	594.2493	0.0008	1.4	21.42	1	y5 -H2O -CO [6|7][1-6][4|5][2-6]	C29H35N7O5S
594.2501	594.2493	0.0008	1.4	21.42	1	b5 -H2O -CO [6|7][1-6][5|6][1-5]	C29H35N7O5S
595.2338	595.2333	0.0005	0.8	0.61	1	y5 -NH3 -CO [4|5][1-6][1|2][2-6]	C29H34N6O6S
595.2338	595.2333	0.0005	0.8	0.61	1	b5 -NH3 -CO [4|5][1-6][2|3][1-5]	C29H34N6O6S
595.2338	595.2333	0.0005	0.8	0.61	1	y5 -NH3 -CO [6|7][1-6][4|5][2-6]	C29H34N6O6S
595.2338	595.2333	0.0005	0.8	0.61	1	b5 -NH3 -CO [6|7][1-6][5|6][1-5]	C29H34N6O6S
596.2294	596.2286	0.0008	1.3	0.32	1	y5 [7|1][1-6][1|2][2-6]	C28H33N7O6S
596.2294	596.2286	0.0008	1.3	0.32	1	b5 [7|1][1-6][2|3][1-5]	C28H33N7O6S
596.2294	596.2286	0.0008	1.3	0.32	1	y5 [2|3][1-6][4|5][2-6]	C28H33N7O6S
596.2294	596.2286	0.0008	1.3	0.32	1	b5 [2|3][1-6][5|6][1-5]	C28H33N7O6S
596.2294	596.2286	0.0008	1.3	0.32	1	y5 -COCH2 [3|4][3-7]	C28H33N7O6S
596.2294	596.2286	0.0008	1.3	0.32	1	b5 -COCH2 [5|6][1-5]	C28H33N7O6S
596.2294	596.2286	0.0008	1.3	0.32	1	y5 -CO [7|1][1-6][4|5][2-6]	C28H33N7O6S
596.2294	596.2286	0.0008	1.3	0.32	1	b5 -CO [7|1][1-6][5|6][1-5]	C28H33N7O6S
596.2294	596.2286	0.0008	1.3	0.32	1	y5 -CO [5|6][1-6][1|2][2-6]	C28H33N7O6S
596.2294	596.2286	0.0008	1.3	0.32	1	b5 -CO [5|6][1-6][2|3][1-5]	C28H33N7O6S
598.2449	598.2442	0.0007	1.2	6.72	1	y5 -COCH2 [6|7][1-6][4|5][2-6]	C28H35N7O6S
598.2449	598.2442	0.0007	1.2	6.72	1	b5 -COCH2 [6|7][1-6][5|6][1-5]	C28H35N7O6S
598.2449	598.2442	0.0007	1.2	6.72	1	b5 -CO [7|1][1-5]	C28H35N7O6S
598.2449	598.2442	0.0007	1.2	6.72	1	y5 -CO [5|6][3-7]	C28H35N7O6S
598.2449	598.2442	0.0007	1.2	6.72	1	y5 -COCH2 [4|5][1-6][1|2][2-6]	C28H35N7O6S
598.2449	598.2442	0.0007	1.2	6.72	1	b5 -COCH2 [4|5][1-6][2|3][1-5]	C28H35N7O6S
603.2352	603.2344	0.0008	1.4	74.03	1	M -C10H9NO -H2O [7|1][1-7]	C26H34N8O7S
604.2192	604.2184	0.0008	1.4	0.17	1	M -C10H9NO -NH3 [7|1][1-7]	C26H33N7O8S
607.2341	607.2333	0.0007	1.2	0.34	1	b5 -NH3 -CO [7|1][1-6][4|5][1-5]	C30H34N6O6S
607.2341	607.2333	0.0007	1.2	0.34	1	y5 -NH3 -CO [7|1][1-6][3|4][2-6]	C30H34N6O6S
607.2341	607.2333	0.0007	1.2	0.34	1	y5 -NH3 -CO [4|5][1-6][2|3][2-6]	C30H34N6O6S
607.2341	607.2333	0.0007	1.2	0.34	1	b5 -NH3 -CO [4|5][1-6][3|4][1-5]	C30H34N6O6S
608.2296	608.2286	0.0010	1.7	0.87	1	b5 -H2O [7|1][1-5]	C29H33N7O6S
608.2296	608.2286	0.0010	1.7	0.87	1	y5 -H2O [5|6][3-7]	C29H33N7O6S
609.2132	609.2126	0.0006	1.0	1.02	1	y5 -NH3 [5|6][3-7]	C29H32N6O7S
609.2132	609.2126	0.0006	1.0	1.02	1	b5 -NH3 [7|1][1-5]	C29H32N6O7S
610.2448	610.2442	0.0006	1.0	1.36	1	y5 -COCH2 [7|1][1-6][3|4][2-6]	C29H35N7O6S
610.2448	610.2442	0.0006	1.0	1.36	1	y5 [4|5][1-6][4|5][2-6]	C29H35N7O6S
610.2448	610.2442	0.0006	1.0	1.36	1	y5 [2|3][1-6][1|2][2-6]	C29H35N7O6S
610.2448	610.2442	0.0006	1.0	1.36	1	b5 [2|3][1-6][2|3][1-5]	C29H35N7O6S
610.2448	610.2442	0.0006	1.0	1.36	1	y5 -CO [3|4][3-7]	C29H35N7O6S
610.2448	610.2442	0.0006	1.0	1.36	1	b5 [4|5][1-6][5|6][1-5]	C29H35N7O6S
610.2448	610.2442	0.0006	1.0	1.36	1	b5 -CO [5|6][1-5]	C29H35N7O6S
610.2448	610.2442	0.0006	1.0	1.36	1	b5 -COCH2 [7|1][1-6][4|5][1-5]	C29H35N7O6S
610.2448	610.2442	0.0006	1.0	1.36	1	b5 -COCH2 [4|5][1-6][3|4][1-5]	C29H35N7O6S
610.2448	610.2442	0.0006	1.0	1.36	1	y5 -COCH2 [4|5][1-6][2|3][2-6]	C29H35N7O6S
612.2243	612.2235	0.0008	1.3	8.68	1	b5 [6|7][1-5]	C28H33N7O7S
612.2243	612.2235	0.0008	1.3	8.68	1	y5 [4|5][3-7]	C28H33N7O7S
620.2291	620.2286	0.0005	0.9	4.27	1	y5 -H2O [3|4][3-7]	C30H33N7O6S
620.2291	620.2286	0.0005	0.9	4.27	1	b5 -H2O [5|6][1-5]	C30H33N7O6S
621.2134	621.2126	0.0008	1.3	28.79	1	y5 -NH3 [3|4][3-7]	C30H32N6O7S
621.2134	621.2126	0.0008	1.3	28.79	1	b5 -NH3 [5|6][1-5]	C30H32N6O7S
621.2458	621.2450	0.0009	1.4	7.30	1	M -C10H9NO [7|1][1-7]	C26H36N8O8S
623.2293	623.2282	0.0011	1.7	0.15	1	y5 -NH3 [4|5][1-6][1|2][2-6]	C30H34N6O7S

Meas. m/z	Calc. m/z	δ (Da)	δ (ppm)	Rel. Int. (%)	z	Annotation	Formula
623.2293	623.2282	0.0011	1.7	0.15	1	b5 -NH3 [4|5][1-6][2|3][1-5]	C30H34N6O7S
623.2293	623.2282	0.0011	1.7	0.15	1	y5 -NH3 [6|7][1-6][4|5][2-6]	C30H34N6O7S
623.2293	623.2282	0.0011	1.7	0.15	1	b5 -NH3 [6|7][1-6][5|6][1-5]	C30H34N6O7S
624.2244	624.2235	0.0009	1.5	0.91	1	y5 [7|1][1-6][4|5][2-6]	C29H33N7O7S
624.2244	624.2235	0.0009	1.5	0.91	1	b5 [7|1][1-6][5|6][1-5]	C29H33N7O7S
624.2244	624.2235	0.0009	1.5	0.91	1	y5 [5|6][1-6][1|2][2-6]	C29H33N7O7S
624.2244	624.2235	0.0009	1.5	0.91	1	b5 [5|6][1-6][2|3][1-5]	C29H33N7O7S
626.2401	626.2391	0.0009	1.4	1.60	1	b5 [7|1][1-5]	C29H35N7O7S
626.2401	626.2391	0.0009	1.4	1.60	1	y5 [5|6][3-7]	C29H35N7O7S
635.2404	635.2395	0.0009	1.5	0.28	1	y6 -COCH2 -H2O [4|5][2-7]	C30H34N8O6S
635.2404	635.2395	0.0009	1.5	0.28	1	b6 -COCH2 -H2O [5|6][1-6]	C30H34N8O6S
636.2239	636.2235	0.0004	0.7	1.16	1	b6 -COCH2 -NH3 [5|6][1-6]	C30H33N7O7S
636.2239	636.2235	0.0004	0.7	1.16	1	y6 -COCH2 -NH3 [4|5][2-7]	C30H33N7O7S
638.2401	638.2391	0.0009	1.4	10.71	1	b5 [5|6][1-5]	C30H35N7O7S
638.2401	638.2391	0.0009	1.4	10.71	1	b6 -COCH2 -NH3 [6|7][1-6]	C30H35N7O7S
638.2401	638.2391	0.0009	1.4	10.71	1	y6 -COCH2 -NH3 [5|6][2-7]	C30H35N7O7S
638.2401	638.2391	0.0009	1.4	10.71	1	y5 [3|4][3-7]	C30H35N7O7S
639.2718	639.2708	0.0010	1.6	0.43	1	b6 -CO -COCH2 [7|1][1-6]	C30H38N8O6S
639.2718	639.2708	0.0010	1.6	0.43	1	y6 -CO -COCH2 [6|7][2-7]	C30H38N8O6S
639.2718	639.2708	0.0010	1.6	0.43	1	b6 -CO [2|3][1-6]	C30H38N8O6S
639.2718	639.2708	0.0010	1.6	0.43	1	y6 -CO [1|2][2-7]	C30H38N8O6S
649.2563	649.2551	0.0012	1.9	1.31	1	y6 -H2O [1|2][2-7]	C31H36N8O6S
649.2563	649.2551	0.0012	1.9	1.31	1	b6 -COCH2 -H2O [7|1][1-6]	C31H36N8O6S
649.2563	649.2551	0.0012	1.9	1.31	1	y6 -COCH2 -H2O [6|7][2-7]	C31H36N8O6S
649.2563	649.2551	0.0012	1.9	1.31	1	y6 -H2O -CO [4|5][2-7]	C31H36N8O6S
649.2563	649.2551	0.0012	1.9	1.31	1	b6 -H2O -CO [5|6][1-6]	C31H36N8O6S
649.2563	649.2551	0.0012	1.9	1.31	1	b6 -H2O [2|3][1-6]	C31H36N8O6S
650.2399	650.2391	0.0008	1.2	0.65	1	b6 -NH3 -CO [5|6][1-6]	C31H35N7O7S
650.2399	650.2391	0.0008	1.2	0.65	1	y6 -COCH2 -NH3 [6|7][2-7]	C31H35N7O7S
650.2399	650.2391	0.0008	1.2	0.65	1	b6 -NH3 [2|3][1-6]	C31H35N7O7S
650.2399	650.2391	0.0008	1.2	0.65	1	y6 -NH3 -CO [4|5][2-7]	C31H35N7O7S
650.2399	650.2391	0.0008	1.2	0.65	1	y6 -NH3 [1|2][2-7]	C31H35N7O7S
650.2399	650.2391	0.0008	1.2	0.65	1	b6 -COCH2 -NH3 [7|1][1-6]	C31H35N7O7S
651.2717	651.2708	0.0010	1.5	0.99	1	y6 -H2O -CO [5|6][2-7]	C31H38N8O6S
651.2717	651.2708	0.0010	1.5	0.99	1	b6 -H2O -CO [6|7][1-6]	C31H38N8O6S
652.2559	652.2548	0.0011	1.7	0.25	1	y5 [7|1][1-6][3|4][2-6]	C31H37N7O7S
652.2559	652.2548	0.0011	1.7	0.25	1	b5 [4|5][1-6][3|4][1-5]	C31H37N7O7S
652.2559	652.2548	0.0011	1.7	0.25	1	b6 -NH3 -CO [6|7][1-6]	C31H37N7O7S
652.2559	652.2548	0.0011	1.7	0.25	1	b5 [7|1][1-6][4|5][1-5]	C31H37N7O7S
652.2559	652.2548	0.0011	1.7	0.25	1	y5 [4|5][1-6][2|3][2-6]	C31H37N7O7S
652.2559	652.2548	0.0011	1.7	0.25	1	y6 -NH3 -CO [5|6][2-7]	C31H37N7O7S
663.2714	663.2708	0.0006	1.0	0.38	1	b6 -H2O -CO [7|1][1-6]	C32H38N8O6S
663.2714	663.2708	0.0006	1.0	0.38	1	b6 -COCH2 -H2O [4|5][1-6]	C32H38N8O6S
663.2714	663.2708	0.0006	1.0	0.38	1	y6 -COCH2 -H2O [3|4][2-7]	C32H38N8O6S
663.2714	663.2708	0.0006	1.0	0.38	1	y6 -H2O -CO [6|7][2-7]	C32H38N8O6S
667.2666	667.2657	0.0009	1.4	5.34	1	y6 [1|2][2-7]	C31H38N8O7S
667.2666	667.2657	0.0009	1.4	5.34	1	y6 -CO [4|5][2-7]	C31H38N8O7S
667.2666	667.2657	0.0009	1.4	5.34	1	b6 [2|3][1-6]	C31H38N8O7S
667.2666	667.2657	0.0009	1.4	5.34	1	b6 -COCH2 [7|1][1-6]	C31H38N8O7S
667.2666	667.2657	0.0009	1.4	5.34	1	b6 -CO [5|6][1-6]	C31H38N8O7S
667.2666	667.2657	0.0009	1.4	5.34	1	y6 -COCH2 [6|7][2-7]	C31H38N8O7S

Meas. m/z	Calc. m/z	δ (Da)	δ (ppm)	Rel. Int. (%)	z	Annotation	Formula
669.2823	669.2813	0.0010	1.5	19.53	1	y6 -CO [5|6][2-7]	C31H40N8O7S
669.2823	669.2813	0.0010	1.5	19.53	1	b6 -CO [6|7][1-6]	C31H40N8O7S
677.2510	677.2500	0.0009	1.4	22.61	1	y6 -H2O [4|5][2-7]	C32H36N8O7S
677.2510	677.2500	0.0009	1.4	22.61	1	b6 -H2O [5|6][1-6]	C32H36N8O7S
678.2350	678.2341	0.0010	1.5	29.51	1	b6 -NH3 [5|6][1-6]	C32H35N7O8S
678.2350	678.2341	0.0010	1.5	29.51	1	y6 -NH3 [4|5][2-7]	C32H35N7O8S
678.2712	678.2704	0.0007	1.1	24.25	1	y6 -NH3 -CO [3|4][2-7]	C33H39N7O7S
678.2712	678.2704	0.0007	1.1	24.25	1	b6 -NH3 -CO [4|5][1-6]	C33H39N7O7S
679.2664	679.2657	0.0007	1.1	1.73	1	y6 -H2O [5|6][2-7]	C32H38N8O7S
679.2664	679.2657	0.0007	1.1	1.73	1	b6 -H2O [6|7][1-6]	C32H38N8O7S
681.2823	681.2813	0.0009	1.4	1.89	1	y6 -CO [6|7][2-7]	C32H40N8O7S
681.2823	681.2813	0.0009	1.4	1.89	1	b6 -CO [7|1][1-6]	C32H40N8O7S
681.2823	681.2813	0.0009	1.4	1.89	1	b6 -COCH2 [4|5][1-6]	C32H40N8O7S
681.2823	681.2813	0.0009	1.4	1.89	1	y6 -COCH2 [3|4][2-7]	C32H40N8O7S
691.2669	691.2657	0.0012	1.8	1.38	1	b6 -H2O [7|1][1-6]	C33H38N8O7S
691.2669	691.2657	0.0012	1.8	1.38	1	y6 -H2O [6|7][2-7]	C33H38N8O7S
695.2615	695.2606	0.0009	1.3	100.00	1	b6 [5|6][1-6]	C32H38N8O8S
695.2615	695.2606	0.0009	1.3	100.00	1	y6 [4|5][2-7]	C32H38N8O8S
697.2774	697.2763	0.0011	1.6	2.38	1	y6 [5|6][2-7]	C32H40N8O8S
697.2774	697.2763	0.0011	1.6	2.38	1	b6 [6|7][1-6]	C32H40N8O8S
705.2815	705.2813	0.0002	0.2	0.24	1	y6 -H2O [3|4][2-7]	C34H40N8O7S
705.2815	705.2813	0.0002	0.2	0.24	1	b6 -H2O [4|5][1-6]	C34H40N8O7S
709.2772	709.2763	0.0010	1.4	4.31	1	b6 [7|1][1-6]	C33H40N8O8S
709.2772	709.2763	0.0010	1.4	4.31	1	y6 [6|7][2-7]	C33H40N8O8S
720.2933	720.2922	0.0011	1.5	3.28	1	M -COCH2 -H2O [7|1][1-7]	C34H41N9O7S
734.3090	734.3079	0.0011	1.5	52.13	1	M -H2O -CO [7|1][1-7]	C35H43N9O7S
735.2930	735.2919	0.0011	1.4	20.51	1	M -NH3 -CO [7|1][1-7]	C35H42N8O8S
745.2768	745.2763	0.0005	0.7	0.63	1	M -H2O -NH3 [7|1][1-7]	C36H40N8O8S
752.3194	752.3185	0.0009	1.2	93.81	1	M -CO [7|1][1-7]	C35H45N9O8S
762.3037	762.3028	0.0009	1.2	85.56	1	M -H2O [7|1][1-7]	C36H43N9O8S
763.2879	763.2868	0.0011	1.4	3.38	1	M -NH3 [7|1][1-7]	C36H42N8O9S

Generated by mMass • Open Source Mass Spectrometry Tool • www.mmass.org
